# Profiling Serine Hydrolases in the *Leishmania* Host–Pathogen Interactome Using Cell‐Permeable Activity‐Based Fluorophosphonate Probes

**DOI:** 10.1002/cbic.202500160

**Published:** 2025-05-14

**Authors:** Jaime A. Isern, Exequiel O. J. Porta, Karunakaran Kalesh, Zisis Koutsogiannis, Davide Cazzola, Ehmke Pohl, Paul W. Denny, Patrick G. Steel

**Affiliations:** ^1^ Department of Chemistry Durham University Stockton Rd. Durham DH1 3LE UK; ^2^ School of Health and Life Sciences Teesside University Southfield Rd. Middlesbrough TS1 3BX UK; ^3^ National Horizons Centre Darlington DL1 1HG UK; ^4^ Department of Biosciences Durham University Durham DH1 3LE UK

**Keywords:** fluorophosphonate probes, host–pathogen interactomes, *Leishmania*, proteomics, serine hydrolases

## Abstract

Leishmaniasis, a vector‐borne neglected tropical disease, caused by the protozoan parasite *Leishmania*, is a major global public health challenge with millions of new cases annually. Treatment of leishmaniasis is difficult for many reasons including multiple lifecycle stages, involving both an infective insect vector form, the promastigote, and a disease‐causing intracellular mammalian host form, the amastigote, and increasing drug tolerance that are all linked by the interplay between parasite and host. Activity‐based protein profiling (ABPP) was employed using new cell‐permeable fluorophosphonate probes to explore serine hydrolases (SHs) in *Leishmania mexicana* with subsequent analysis enabled by secondary reaction with an affinity reagent. Importantly, these cell‐permeable probes are capable of accessing all lifecycle stages including the disease‐critical intramacrophage amastigote. Probe efficacy is a combination of both target engagement and subsequent accessibility to the affinity agent. Fourteen SHs, including peptidases and lipases, were identified in the *L. mexicana* proteome with comparative profiling of different parasite life‐stages revealing significant changes in SH activity across the lifecycle stages. This intracellular ABPP approach provides insights into the host–parasite interactome demonstrating that SHs function as important virulence factors with Z‐Pro‐Prolinal, a known prolyl‐oligopeptidase inhibitor, being able to reduce parasite infectivity in the macrophage by altering multiple SH targets.

## Graphical abstract

Leishmaniasis poses a global health challenge with limited treatment options and rising drug resistance. Activity‐based protein profiling using fluorophosphonate probes is employed to profile serine hydrolases, key virulence factors in *Leishmania mexicana*. Changes in SHs expression levels across the life stages highlight their critical role in the parasite–host interactome, providing valuable insights into infectivity mechanisms and potential therapeutic targets.

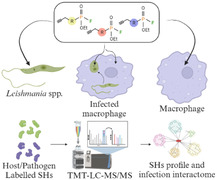

## 1 Introduction

Leishmaniasis, a group of vector‐borne parasitic diseases caused by species of the kinetoplastid parasite *Leishmania*, is a neglected tropical disease (NTD) of global importance. With over 12 million people infected, 0.9–1.6 million new cases annually, more than 30,000 deaths per year, and more than 350 million people considered at risk, leishmaniasis is a major public health concern. Currently, there are no effective vaccines and treatment relies solely on chemotherapy. This is challenged by diminishing efficacy, high toxicity, cost, lengthy and painful treatment regimens leading to poor patient adherence, and increasing drug resistance.^[^ ^1^, ^2^, ^3^, ^4^ ^]^ Consequently, new treatments are critically needed, as outlined in the World Health Organization's roadmap for NTDs.^[^ ^5^ ^]^

*Leishmania* has a digenetic lifecycle, alternating between an insect vector form (promastigote) and an intracellular mammalian host form (amastigote). The major host cell is the macrophage, where the parasites differentiate from promastigote into the amastigote form. Within the macrophage, where the parasites reside in the parasitophorous vacuole, the interaction between parasite and host is crucial for parasite survival, replication, and differentiation. This interaction involves a complex crosstalk between parasite survival mechanisms and the host immune response with changes in both species occurring concurrently in response to each other. Consequently, given this dynamic interplay between host and parasite, tools that can interrogate both simultaneously are essential to better understand infection, pathogenesis, and treatment.^[^ ^6^, ^7^ ^]^ This can be achieved using small cell‐permeable chemical probes that can access both parasite and host cells and report on the functional activity of their proteomes. While many probes are highly selective to their target proteins, exploring the interactome requires probes that are functionally selective but structurally agnostic, targeting families of enzymes. For this, activity‐based protein profiling (ABPP) has become a powerful strategy for the study of proteins in a wide variety of complex proteomes.^[^ ^8^, ^9^, ^10^ ^]^ While there have been reports of the application of this technique to the study of the host–pathogen interaction,^[^ ^11^, ^12^, ^13^ ^]^ these have largely been undertaken using cell lysate. Particularly for an intracellular parasite such as Leishmania, cell lysis perturbs both individual proteomes and the interactome and therefore impacts the outcome of the experiments.^[^ ^14^, ^15^ ^]^ The use of cell‐permeable probes directly avoids this, providing insights into the unperturbed interactions. In this study, we demonstrate this through an investigation into the role of serine hydrolases (SH) in the leishmania host–macrophage interaction.

The SH superfamily plays a critical role in the transition from promastigote to amastigote, acting as virulence factors and aiding parasite dissemination. SHs are vital throughout the parasite lifecycle, with in silico comparative genomic analysis showing that serine proteases constitute about 10–16% of *Leishmania's degradome*.^[^ ^16^, ^17^ ^]^ Despite extensive studies in other organisms such as humans,^[^ ^18^ ^]^ rice (*Oryza sativa L.*),^[^ ^19^ ^]^ and *Plasmodium falciparum*,^[^ ^20^ ^]^ the *Leishmania* serinome, the complete collection of SHs in an organism, remains surprisingly understudied and offers the potential for new drug targets.^[^ ^16^ ^]^

Activity‐based probes (ABPs) with a fluorophosphonate (FP) warhead are widely used for profiling SHs due to their specificity for active serine residues.^[^ ^18^, ^21^, ^22^ ^]^ Recently, we employed the well‐established tetramethylrhodamine fluorophosphonate (TAMRA‐FP) probe to investigate the serinome of *Leishmania mexicana* promastigotes and identify potential therapeutic targets.^[^ ^23^ ^]^ However, the limited cell permeability of TAMRA‐FP restricts its utility in an intracellular context. Moreover, the fluorescent tag may also contribute to the selectivity profile observed.^[^ ^20^ ^]^ In this report, we describe the synthesis of a set of broad‐spectrum cell‐permeable FP probes and their application to provide insights into the dynamic activity of SHs in *L. mexicana* proteomes across both promastigotes and intramacrophage amastigote lifecycle stages. The enzymes identified are involved in the infection process and represent promising targets for the development of new therapies to combat leishmaniasis.

## 2 Results and Discussion

**Figure 1:**
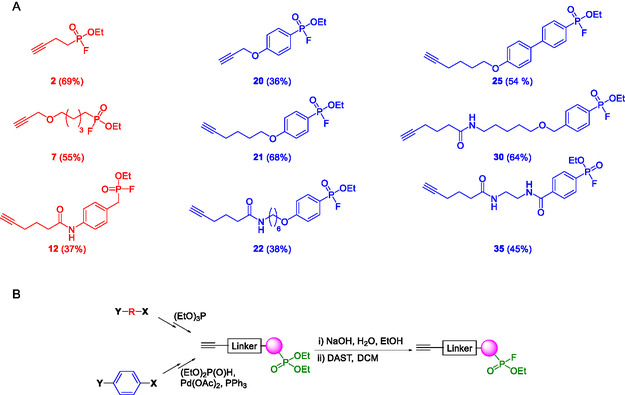
A) Structures of the activity‐based FP probes used in this study: red alkyl probes and blue aryl probes. B) General Synthetic Scheme for FP probe synthesis.

Recent studies have demonstrated that the reactivity and specificity of probes targeting SHs can be tuned by modifying the structure.^[^ ^18^, ^19^, ^22^ ^]^ In our previous study, the use of TAMRA‐FP required the use of cell lysate preparations, resulting in the identification of only a limited number of proteins. To address this limitation, we employed alkyne‐tagged FPs that could be coupled to a reporter after labeling and cell lysis. Whilst a number of related probes have been described, these, predominantly, are based around a design in which the FP warhead is linked to the alkyne (and/or tag) by a simple alkyl or pegylated tether.^[^ ^24^, ^25^, ^26^ ^]^ To assess the importance of this, we aimed to assess how varying the electrophilicity of the phosphorus center and the steric properties of the warhead affected labeling efficiency. These probes were categorized into two sets (**Figure** **1**A). Set one, built on the literature precedents, contained alkyl‐FPs, exploring different spacer lengths between warhead and alkyne, and a benzylic‐FP **12** to examine the impact of introducing steric bulk close to the FP group, while set two were aryl‐FPs. This also examined different tether lengths but, additionally, enabled the effect of changing the electrophilicity of the key phosphorus center to be explored. Each probe could be synthesized from readily available commercial precursors following a common strategy (Figure 1B) of linker construction and introduction of a phosphonate ester by either Arbuzov chemistry for alkyl probes (Scheme S1, Supporting Information) or Hirao palladium‐catalyzed coupling of an aryl bromide with diethyl phosphite for the aryl probes (Scheme S2, Supporting Information). Final deprotection and activation was achieved by selective monohydrolysis, using NaOH in aqueous ethanol, and fluorination with diethylaminosulfur trifluoride (DAST).

**Figure 2:**
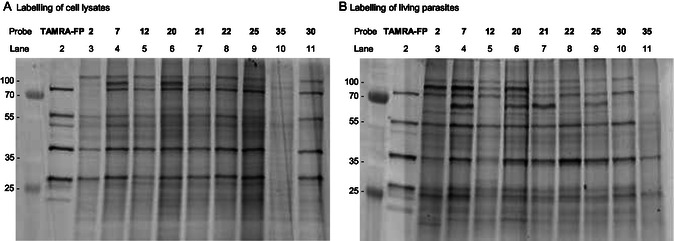
SDS‐PAGE comparison between the labeling capacity of the different probes: A) Labelling using cell lysates: *L. mexicana* lysates were incubated with the stated FP‐alkynes (lanes 3–11) and FP‐TAMRA (lane 2). (B) Labeling using living parasites: *L. mexicana* promastigote cultures were incubated with the stated FP‐alkynes followed by cell lysis. The FP‐TAMRA (lane 2) from gel B) was added as control and comparison, but labelling was conducted as in (A), on cell lysates. Subsequently, Rh–N_3_ was attached via click chemistry. Finally, the samples were separated on SDS‐PAGE and analyzed using fluorescent imaging. Each gel represents a representative output of triplicate experimental runs.

With the probes in hand, we then turned to the analysis of the Leishmania proteome (Figure S1, Supporting Information). After probe treatment of *L. mexicana* promastigote lysates and copper(I)‐catalyzed azide‐alkyne cycloaddition (CuAAC) click reaction with rhodamine‐azide (Rh‐N_3_), the tagged proteins were separated by sodium dodecyl sulfate‐polyacrylamide gel electrophoresis (SDS‐PAGE) and analyzed by in‐gel fluorescence (emission at 560 nm). Importantly, verifying the intrinsic selectivity of FP warheads for SHs, the proteins most intensely labeled by TAMRA‐FP (**Figure** **2**A, lane 2) were also identified by most of the ABPs, except for electron‐deficient probe **35** (Figure 2A, lane 10), which failed to label any significant enzymes. Further investigations revealed that probe **35** undergoes extensive and rapid hydrolysis (<5 min) in solvents such as dimethyl sulfoxide (DMSO), methanol (MeOH), phosphate‐buffered saline (PBS) solution, culture media, and H_2_O (data not shown). More bands were observed than with the TAMRA‐FP probe, consistent with the suggestion that the carboxytetramethylrhodamine (TAMRA) fluorophore may not only affect membrane permeability but also play a significant role in modulating the probe activity. This observation is particularly evident in comparison with probe **7** (Figure 2A, lane 4), as both probes share the same warhead and similar linker length.

To assess the impact of cell lysis on the read out of the probes, this analysis was repeated using living parasites. Here, promastigote cultures were incubated with the probes for 1 h at 26 °C before cell lysis, click reaction with Rh‐N_3_, and analysis as before. In contrast to TAMRA‐FP, these cell‐permeable probes labeled a broader enzyme set, particularly within the 55–100 and 20–30 kDa regions. These results suggest that experiments with live parasites offer a superior model for exploring the Leishmania serine hydrolase family when compared with the use of cell lysates as required in experiments with TAMRA‐FP. Comparing alkyl probes **2** and **7** revealed that probe **7** exhibited a higher intensity and produced more bands (Figure 2B, lanes 3 and 4), suggesting that linker length may influence labelling efficiency, potentially by enhancing alkyne handle accessibility and solvent exposure, thereby facilitating the click reaction. However, this trend was not observed with phenolic probes **20–22**, where labelling patterns were more consistent across the three probes evaluated with small but distinct differences between them (Figure 2B, lanes 5, 7, and 8). Overall, these results highlight the value of using cell‐permeable FP probes.

**Figure 4:**
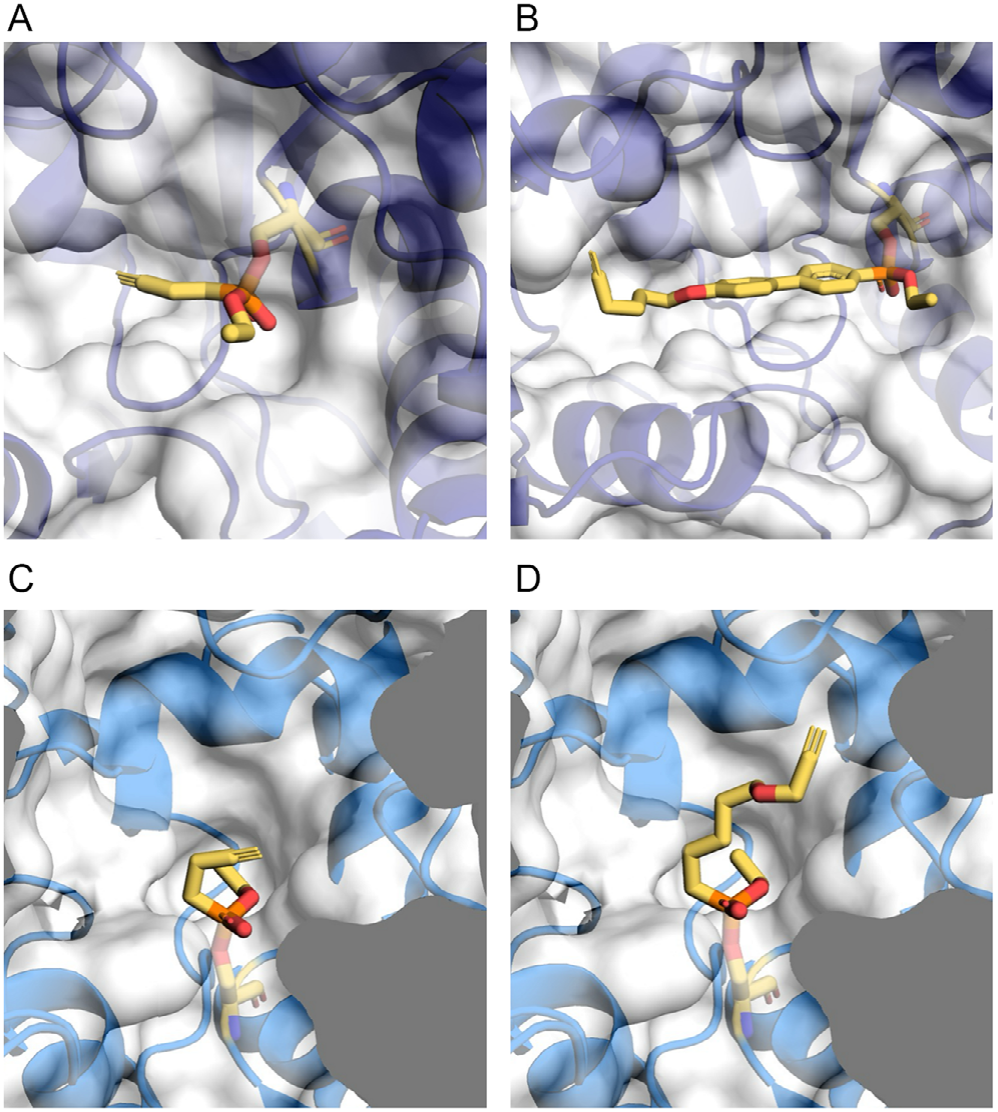
Close up of active sites of A,B) E9B6I1 and C,D) E9AUB0 showing docked probes covalently bound to the catalytic serine. Protein models are depicted as cartoons with transparent surfaces, docked ligands, and active serines in stick representation. Carbon: yellow; Nitrogen: blue; Oxygen: red; and Phosphorous: orange. Hydrogens omitted for clarity. (A) Probe **2**; (B) Probe **25**; (C) Probe **2**; and (D) Probe **7**.

To provide greater insight into the *Leishmania serinome* identified by the probes, isobaric stable isotope labelling, utilizing tandem mass tagging (TMT) and quantitative proteomic mass spectrometry (MS), was employed.^[^ ^27^, ^28^ ^]^ In brief, after probe incubation, cell lysis, and click coupling with commercial biotin‐N_3_, the samples were then enriched using streptavidin pull‐down, reduced, alkylated, digested, labeled with the TMT tags, and analyzed by LC‐MS/MS. All experiments were performed in biological replicates. A modified *t‐*test with permutation‐based FDR statistics (250 permutations, FDR = 0.05) was applied to compare probe treatments versus control groups as reported before.^[^ ^29^ ^]^ After data processing, 14 SHs were identified: 12 peptidases and 2 lipases (Figure S2, Supporting Information). As before, alkyl‐FP **7** exhibited the broadest scope, labelling all 14 SH identified, followed by alkyl‐FP **2**, which identified 12 but with lower affinity as depicted by the lower log2 fold‐change (FC) values obtained (**Figure** **3**). This is likely due to linker length and alkyne accessibility, a proposal supported by comparing labeling experiments using probes **2** and **30** with two samples of lysate with one being denatured prior to the click reaction. Consistent with accessibility to the alkyne being a critical factor, additional enzymes were labeled when the lysate was denatured by heating to 95 °C before the click reaction. (Figure S3, Supporting Information). Similar trends were observed with aryl‐FP probes **20**, **21,** and **22**, also pointing to an optimal probe structure for each protein. To further explore this observation, AlphaFold was used to predict the structure for each protein.^[^ ^30^ ^]^ Importantly, since serine hydrolase core structures are evolutionary well‐conserved, the structure predictions with AlphaFold had a very high confidence level and are therefore well‐suited to model the different probes into the respective active sites. Probes were then docked into the respective binding sites using the covalent docking protocol implemented in GOLD cambridge crystallographic data centre (CCDC) in which the key reactive serine residue was phosphonylated with each FP probe^[^ ^31^, ^32^, ^33^ ^]^ (Table S1, Supporting Information). In all cases, the docking was successful and produced acceptable docking scores and chemically reasonable poses, validating the overall probe design. The docking poses were then correlated with the labelling efficiency (Figure 3) of the different probes. In general, probes with high labelling efficiency showed excellent docking poses in which the alkyne was highly accessible for linking to the biotin‐N_3_ via a click reaction whereas the less efficient probes were found to have adopted conformations in which the alkyne was directed into a hydrophobic region of the protein and was not accessible to the azide reagent. For example, using putative 1‐alkyl‐2‐acetylglycerophosphocholine esterase (E9B6I1; LmxM.34.3020), the best docking pose of probe **2** shows that the alkyne group extends into a groove accessible to the click reagents (**Figure** **4**A), which is reflected by the high labelling efficiency. Conversely, the more rigid conformation adopted by biphenyl probe **25** directs the alkyne group into an inaccessible hydrophobic pocket (Figure 4B) and thus shows no labeling. Similarly, while the longer alkyl probe **7** extends into a large solvent‐exposed cavity in the putative prolyl oligopeptidase (POP) (E9AUB0, LmxM.36.6750) enabling alkyne accessibility for efficient click conjugation, the same is not true for the shorter probe **2**, which collapses into a hydrophobic pocket accounting for the loss of labelling (Figure 4C,D).

Figure 3A) Log_2_FC heat map of synthesized probes versus curated SHs identified. The Log_2_FC values, representing the ratio of protein abundance between the treated and untreated samples, range from 0.5 (black) to 1.5 (light green). Briefly, promastigote *L. mexicana* cultures were incubated with the stated FP‐alkynes followed by cell lysis and click attachment of biotin‐N_3_. The samples were then enriched, TMT‐labeled, and analyzed by LC‐MS/MS. B) Venn diagram showing SHs identified using the TMT‐LC‐MS/MS methodology in the promastigote (blue), intracellular amastigote (green), and in vitro axenic amastigote (yellow) forms. C) Venn diagram showing host SHs identified using the TMT‐LC‐MS/MS methodology in uninfected (red) versus infected (orange) murine macrophage. D) Infectivity assay of untreated and 100 μm ZPP‐treated *L. mexicana* metacyclic promastigotes. The assay was conducted at a multiplicity of infection (MOI) of 10 (25 × 10^4^), 5 (12.5 × 10^4^), and 2.5 (62.5 × 10^3^). Values are expressed as the mean ± SD of the relative fluorescence from triplicates of three independent experiments. The relative fluorescence after infection with 25 × 10^4^, untreated *L. mexicana* metacyclic promastigotes was set as 100%. Asterisks indicate *p*‐value significance and set at **p ≤ 0.005 and ***p ≤ 0.0005.

**Figure:**
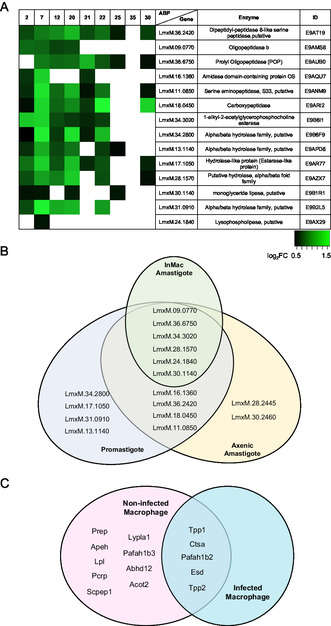

**Figure:**
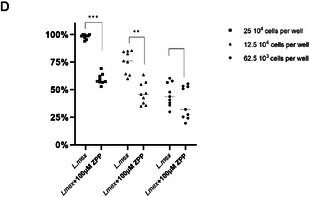

With these enzymes identified as functional in the promastigote parasite, it was of interest to contrast this with that found in the more disease‐relevant amastigotes. We initially used axenic amastigotes, which, although not fully reflective of the true intracellular parasite, serve as a validated model.^[^ ^34^, ^35^ ^]^ Using established protocols for *L. mexicana* axenic amastigote generation,^[^ ^36^ ^]^ parasites were treated with probe **7** as described above, leading to the identification of 16 proteins, two of which, esterase DUF676 (LmxM.28.2445) and putative phospholipase A1 (LmxM.30.2460),^[^ ^37^ ^]^ were unique to the axenic amastigote (Figure 3B).

While the axenic amastigote is simpler to handle, the intramacrophage amastigote is subject to the stresses triggered by the host immune response and the question remains as to how the functional proteome is impacted. Consequently, to analyze how the serinome transition from promastigotes to amastigotes and better understand these interactions with the host, we then explored the serinomes within infected macrophages. Initially, probe **7** was used to determine the host serinome of noninfected murine cells. Following the same TMT‐LC‐MS/MS protocol, 14 murine SHs were identified, comprising seven proteases and seven lipases) (Figure S4 and Table S2, Supporting Information). Subsequently, murine macrophages were infected with *L. mexicana* metacyclic promastigotes and, 4 h postinfection, treated with probe **7**.

Following TMT analysis as before, a total of 6 *Leishmania* SHs (three proteases and three lipases) were identified (**Table** **1**, Figure 3B, and S5, Supporting Information). Importantly, these enzymes were also identified in both the axenic and promastigote forms, suggesting important roles in parasite survival throughout the lifecycle. In parallel, analysis of the host serinome postinfection revealed that only 5 of the 14 enzymes (three proteases, a thioesterase and a lipase) were differentially labeled by probe **7** indicating increased activity due to the infection (**Table** **2**, Figure 3C, and S6, Supporting Information).

**Table 1:** Curated list of SHs identified in intracellular amastigotes after macrophage infection through treatment with probe 7, followed by TMT LC‐MS/MS analysis.

ID	Enzyme	Gene	Log_2_ FC
E9AMS8	Oligopeptidase B	LmxM.09.0770	1.28
E9AUB0	POP	LmxM.36.6750	1.08
E9B6I1	Phospholipase A2‐like protein	LmxM.34.3020	0.95
E9AZX7	Hydrolase, alpha/beta fold family	LmxM.28.1570	1.21
E9B1R1	Monoglyceride lipase	LmxM.30.1140	1.71
E9AX29	Lysophospholipase	LmxM.24.1840	0.88

**Table 2:** Curated list of macrophage SHs identified after infection through treatment with probe 7, followed by TMT LC‐MS/MS analysis.

Enzyme	Gene	Log_2_ FC
S‐formylglutathione hydrolase	Esd	0.75
Tripeptidyl‐peptidase 2	Tpp2	0.51
Peptidase S53 domain‐containing protein	Tpp1	0.40
Carboxypeptidase	Ctsa	0.29
1‐alkyl‐2‐acetylglycerophosphocholine esterase	Pafah1b2	0.29

To further explore the role of the serinome in parasite survival, we selected the prolyl oligopeptidase LmxM.36.6750 (POP), an enzyme active in both promastigotes and intracellular amastigotes for further study. Significantly, POP has been identified as having a role in macrophage invasion by *L. infantum* and that this process can be impacted by treatment with the protease inhibitor Z‐pro‐prolinal (ZP).^[^ ^38^ ^]^ Having previously verified that ZPP is a potent inhibitor of *L. mexicana* POP (IC_50_ < 20 pm),^[^ ^17^ ^]^ we used *L. mexicana* and RAW 264.7 macrophages to investigate the effect of ZPP on *L. mexicana* macrophage infection. As initial experiments, using pretreatment of the promastigotes with 50 μm ZPP prior to infection of the macrophage did not show any impact we used 100 μm ZPP for 2 h and, consistent with Lasse's report,^[^ ^38^ ^]^ ZPP treatment led to a reduction in the infection process (Figure 3D). Interestingly, prolonged infection periods (e.g., greater than 6 h) with ZPP‐treated parasites did not show any effect on the infection. To account for this, we suggest that, although ZPP is a covalent inhibitor, it still binds reversibly, so the longer incubation times potentially lead to loss of the inhibition, through decomposition pathways or binding to other enzymes and restoration of infection.^[^ ^39^ ^]^

Cell‐permeable probes allow real‐time study of proteomic processes, with quantitative chemoproteomics providing both qualitative and quantitative insights. Consequently, we conducted competitive ABPP (cABPP) using ZPP and probe **7** coupled with TMT‐LC‐MS/MS identifying 25 differentially enriched proteins. Among these, eight were SHs and whilst POP shows the highest fold change, these results indicate that multiple enzymes are impacted by ZPP treatment. This suggests that the observed reduction in *L. mexicana* infectivity may be linked to other effects of ZPP action rather than on POP alone (**Table** **3**, Figure S7, and Table S3, Supporting Information).

**Table 3:** (Promastigote) enzymes differentially labeled in promastigotes after cABPP between ZPP and probe 7, as identified using TMT LC‐MS/MS.

ID	Protein Name	Gene 3b)	Log_2_ FCa)
E9AUB0	POP	LmxM.36.6750*	2.78
E9B066	Putative serine esterase (DUF676)	LmxM.28.2445	1.85
E9AT19	Dipeptidyl‐peptidase 8‐like serine peptidase, putative	LmxM.36.2420	1.51
E9AZX7	Putative hydrolase, alpha/beta fold family	LmxM.28.1570*	1.45
E9AMS8	Oligopeptidase b	LmxM.09.0770*	1.42
E9B1R1	Monoglyceride lipase	LmxM.30.1140*	1.15
E9B6I1	1‐alkyl‐2‐acetylglycerophosphocholine esterase	LmxM.34.3020*	1.08
E9AX29	Lysophospholipase	LmxM.24.1840*	0.73

a) FC shows enzymes more highly labeled in samples not treated with.

b) *indicates enzyme that is also observed in the infected macrophage.

## 3 Discussion

*Leishmania* has a digenetic lifecycle involving an insect vector promastigote form and an intracellular mammalian host infective amastigote form, with transitions between the two being critical to disease transmission. As an obligate intracellular parasite, *Leishmania* relies on interactions with host immune responses to survive. As SHs play essential roles in these processes,^[^ ^16^ ^]^ understanding the mechanisms behind these interactions is crucial for drug development.^[^ ^40^, ^41^ ^]^

Proteomes are dynamic and highly regulated systems with the constituent proteins often existing in inactive forms until proteolytically processed or post‐translationally modified. Consequently, whilst global proteome analysis of Leishmania (and other systems) can give insights into the response to environmental stimuli, including that induced by the host immune response, it does not afford quantifiable measurement of the functional activity of observed proteins. On the other hand, ABPP is a powerful tool for studying host–pathogen interactions, as it measures protein activity rather than expression levels in complex proteomes.^[^ ^42^, ^43^ ^]^ As such these are complementary processes, although comparisons between the two outputs require the use of similar conditions and species.

SH‐targeting ABPs are well established and include warheads based on FPs, phosphonate esters, and sulfonyl fluorides.^[^ ^10^, ^44^, ^45^ ^]^ Here we have synthesized FP and ABPs varying in structure and electrophilicity. Although this concept has been explored with various other warheads, such as phosphonate esters^[^ ^46^, ^47^, ^48^ ^]^ or sulfonyl fluorides^[^ ^49^ ^]^ fluorophosphonates are largely conserved, typically based on a simple alkylfluorophosphonate as represented by the commercially available TAMRA‐FP. Although aryl fluorophosphonates are well‐precedented, applications as components of ABPs appear surprisingly limited.^[^ ^44^, ^50^, ^51^ ^]^

Reflecting the structure of many probes, which contain either a preassembled fluorophore or affinity handle, most ABPP workflows use cell lysate, removing proteins from their native environments. However, this can lead to enzyme inactivation and loss of labelling during cell lysis.^[^ ^52^, ^53^ ^]^ Consequently, *in vitro* (lysate) preparations only capture a fraction of the functional proteome. To address this, simpler probes conjugated post‐labelling to enrichment and analysis enabling tags have been advocated.^[^ ^14^, ^52^ ^]^ Our results using cell‐permeable probes align with this, as the gels revealed proteins not visible under *in vitro* conditions (Figure 2A,B).

Apart from probe **35**, which lacked stability, all the probes labeled a subset of the *Leishmania* serinome. The variations in labelling patterns with different probes validated our hypothesis that labelling is dependent on both probe structure and target protein. Unlike studies focusing on a single‐protein target, where selectivity is crucial, studying whole proteomes requires broad coverage. Due to the limitations of in‐gel fluorescence ABPP for identifying probe‐labeled proteins, we used a gel‐free approach with biotin‐streptavidin enrichment followed by TMT LC‐MS/MS analysis. In general, alkyl fluorophosphonates exhibited broader labelling than their aryl counterparts. This can be explained by the higher electrophilicity of the alkyl probes, as evidenced by the chemical shifts observed in ^31^P and ^19^F NMR spectra (Table S4, Supporting Information) and their ease of access to the active site serine residue. In addition, efficiency in the click reaction, with biotin‐N_3_, is equally critical, explaining why the longer linker in probe **7** enabled more effective labelling than the smaller and potentially less sterically demanding probe **2**. Significantly, these findings could be corroborated using covalent docking protocols in AlphaFold. Importantly, as serine hydrolase core structures are evolutionarily well conserved, these structure predictions have a very high confidence level and are thus well suited for modelling the different probes into the respective active sites.

Probe **7** was employed to label the serinome of promastigotes, axenic amastigotes, and intracellular amastigotes from infected murine macrophages. For the infection process, it is the differences in labelling between promastigotes and amastigotes that are most relevant. Alves et al. previously reported that the activity of serine (as well as aspartic‐, and metallo‐) proteinase activity is observed in promastigotes and early transformation stages, but is downregulated in axenic amastigotes.^[^ ^16^ ^]^ Our results are consistent with these findings, as only ten of the fourteen SH enzymes identified as active in promastigotes were observed in axenic amastigotes, and only six in the intramacrophage phase. However, we note the caveat that, due to the low parasite‐to‐host protein ratio in infected cell lines, identifying the complete set of SHs in the intracellular parasite may be challenging due to protein dilution within the host proteome.

Notably, with the potential exception of LmxM.28.1570, a putative hydrolase without a clearly defined function, all the amastigote SHs detected in the macrophage have reported roles in virulence and lipid metabolism, which are crucial for parasite survival. Proteases such as POP and oligopeptidase B (OPB) are known virulence factors, with orthologs involved in macrophage invasion during *T. cruzi* and *L. amazonensis* infections.^[^ ^38^, ^54^, ^55^, ^56^, ^57^ ^]^ Similarly, lipase activity in amastigotes is expected, as *Leishmania* are lipid‐scavenging pathogens with studies showing that lipid metabolism pathways are significantly enhanced in the amastigote form, where lipids become the primary energy source.^[^ ^58^, ^59^, ^60^, ^61^, ^62^ ^]^ For example, lipoprotein‐associated phospholipase A 2/platelet‐activating factor acetylhydrolase LmxM.34.3020 (PLA2/PAF‐AH) degrades platelet‐activating factor‐like lipids, which are crucial for mammalian host infection. Similarly, lysophospholipase (LyPLA, LmxM.24.1840) plays roles in the virulence and pathogenicity of organisms such as bacteria and fungi.^[^ ^63^, ^64^ ^]^ Although its function in trypanosomastids is not fully understood, homologs have been identified in *T. brucei*,^[^ ^65^ ^]^ and its inhibition has been shown to block the in vitro replication of *P. falciparum*.^[^ ^11^ ^]^

Macrophages are key phagocytic cells of the immune system specialized in detecting, engulfing, and destroying foreign pathogens. Recognition receptors and molecules differentiate between “local” and “threat,” which is crucial for balancing tolerance and threat response.^[^ ^66^ ^]^ Pathogens are engulfed as phagosomes form and fuse with lysosomes, creating phagolysosomes in which low pH, reactive oxygen and nitrogen species (ROS and RNS), and protease and lipase activity combine to degrade pathogens.^[^ ^67^, ^68^, ^69^ ^]^ Our analysis of uninfected and infected macrophages reveals multiple active SHs linked to the immune response, suggesting that proteolytic and lipolytic pathways also contribute to the host defense mechanisms.

However, neither macrophages nor parasites operate independently. The difference in response to probe **7** between axenic and intracellular amastigotes may indicate the host's impact on the parasite's functional serinome. Upon macrophage invasion, parasites secrete enzymes for survival, likely affecting the host proteome as well.^[^ ^40^, ^41^, ^70^, ^71^ ^]^ Significantly, many of the macrophage proteins that are no longer observed using probe **7** post‐infection are involved in the immune response. For example, PREP has been linked to inflammation, oxidative stress, and autophagy^[^ ^72^ ^]^ and found to coregulate macrophage function.^[^ ^73^ ^]^ Lysophosphatidylserine lipase (ABHD12) hydrolyses oxidized phosphatidylserine, a proapoptotic trigger induced by oxidative stress,^[^ ^74^ ^]^ and acylamino‐acid‐releasing enzyme degrades oxidatively damaged proteins.^[^ ^75^, ^76^ ^]^ In addition, PAF 1‐alkyl‐2‐acetylglycerophosphocholine esterase (Pafah1b3) is an important effector during inflammatory and immune responses^[^ ^77^, ^78^ ^]^ while exogenous PAF promotes macrophage‐mediated phagocytosis and ROS production, both essential for its leishmanicidal effect in humans.^[^ ^79^ ^]^ Finally, lysosomal prolyl carboxypeptidase has been associated with the kinin‐kallikrein system, which is involved in the inflammation process.^[^ ^80^ ^]^ While the mechanism of this “down regulation” remains to be fully established, this study shows that SHs, and specifically POP plays a role in this process. Although treatment of the promastigote or axenic amastigotes with ZPP, a highly specific inhibitor of POP, has no impact on parasite survival, this treatment significantly reduces the infection in the macrophage. Similar results have been described by Lasse et al.^[^ ^38^ ^]^ However, our results using cABPP suggest that, at the high concentrations required to observe this effect (approximately three orders of magnitude greater than the IC_50_ against the enzyme), there is significant loss of specificity and other elements of the parasite secretome are also involved in this effect

## 4 Conclusions

In conclusion, this study has successfully developed novel cell‐permeable FP probes, with varying warhead reactivities, expanding the toolbox for ABPP studies. These probes which target multiple enzymes have proven valuable for the profiling of serine hydrolases SHs in *Leishmania* spp across the lifecycle, providing insights into the host–parasite interactome and infection processes that would not be possible with noncell permeable probes. In contrast to, many probes that are designed to be specific to a single protein, the use of ABPs that target a broad range of an enzyme family is desirable in this context. Even so, the fact that the most successful probe only labeled 14 of the putative 28 serine‐proteases in *L. mexicana* suggests that further structural variation is required for a fully comprehensive study. For these probes, the outcome is a reflection on the efficiency of both reaction with the enzyme and also conjugation with fluorophore or biotin tag. Our results support the notion that a comprehensive profiling of the serinome requires the use of multiple probes with different reactivities and steric demands. The positive correlation between *in silico* docking and experimental outcomes suggests that the AlphaFold protocol employed here may be of value in identifying suitable probes in future endeavors to profile the complete serinome.

## Conflict of Interest

The authors declare no conflict of interest.

## Author Contributions

**Jaime A. Isern**: data curation (equal); formal analysis (equal); investigation (lead); methodology (equal); writing—original draft (equal); writing—review & editing (supporting). **Exequiel O. J. Porta**: data curation (equal); formal analysis (equal); investigation (equal); methodology (equal); writing—review & editing (equal). **Karunakaran Kalesh**: data curation (equal); formal analysis (equal); investigation (equal); methodology (equal); writing—review & editing (supporting). **Zisis Koutsogiannis**: data curation (equal); formal analysis (equal); investigation (equal); methodology (equal); writing—review & editing (supporting). **Davide Cazzola**: formal analysis (supporting); investigation (supporting); methodology (supporting); writing—review & editing (supporting). **Ehmke Pohl**: formal analysis (supporting); methodology (supporting); supervision (equal); writing—review & editing (supporting). **Paul W. Denny**: formal analysis (equal); supervision (equal); writing—review & editing (supporting). **Patrick G. Steel**: conceptualization (lead); funding acquisition (lead); methodology (lead); project administration (lead); supervision (equal); writing—original draft (equal); writing—review & editing (lead).

## Supporting information

Supplementary Material

## Data Availability

The data that support the findings of this study are available in the supplementary material of this article.
