# Supplementary material for: Profiling Serine Hydrolases in the Leishmania Host–Pathogen Interactome Using Cell‐Permeable Activity‐Based Fluorophosphonate Probes
Source: Chembiochem. 2025 May 14;26(10):e202500160. doi: 10.1002/cbic.202500160 (PMC12117450; doi:10.1002/cbic.202500160)
Supplement: Supplementary file 1 — Supplementary Material [file CBIC-26-e202500160-s001.pdf]

Supporting Information

Profiling Serine Hydrolases In The *Leishmania* Host-Pathogen Interactome Using Cell-Permeable Activity-Based Fluorophosphonate Probes

Jaime A. Isern,[a] Exequiel O. J. Porta,[a] Karunakaran Kalesh,[b,c] Zisis Koutsogiannis,[a] Davide Cazzola,[a] Ehmke Pohl,[a] Paul W. Denny [d] and Patrick G. Steel*[a]

[a] Department of Chemistry, Durham University, Stockton Rd, Durham, DH1 3LE, United Kingdom.

[b] School of Health and Life Sciences, Teesside University, Southfield Rd, TS1 3BX, Middlesbrough, United Kingdom.

[c] National Horizons Centre, Darlington, DL1 1HG, United Kingdom.

[d] Department of Biosciences, Durham University, Durham, DH1 3LE United Kingdom

Table of Contents

SUPPLEMENTARY SCHEMES S1-S2 2
SUPPLEMENTARY FIGURES S1-S7 5
SUPPLEMENTARY TABLES S1-S4 13
MATERIALS AND METHODS 19
GENERAL CONDITIONS 19
GENERAL METHODS 19
EXPERIMENTAL PROCEDURES 21
LEISHMANIA MEXICANA CELL CULTURE 40
RAW 264.7 CELL CULTURE 40
L. MEXICANA – RAW CELL INTERACTOME ASSAYS 40
PROTEIN EXTRACTION FROM *LEISHMANIA* PARASITES 41
LYSATE ABP LABELLING 41
LIVE PARASITES ABP LABELLING 41
BIOORTHOGONAL CU-CATALYSED CYCLOADDITION 41
L. MEXICANA INFECTIVITY ASSAY 42
L. MEXICANA COMPETITIVE ABPP WITH ZPP COUPLED WITH TMT-LC-MS/MS 42
PROTEIN PRECIPITATION 42
AFFINITY ENRICHMENT 43
ON-BEAD REDUCTION, ALKYLATION, AND TRYPTIC DIGESTION 43
TMT LABELLING 43
NANO LC-MS/MS DATA ACQUISITION. 44
PROTEOMICS MS DATA PROCESSING. 44
APPENDIX 46
REFERENCES 93

Supplementary Schemes S1-S2

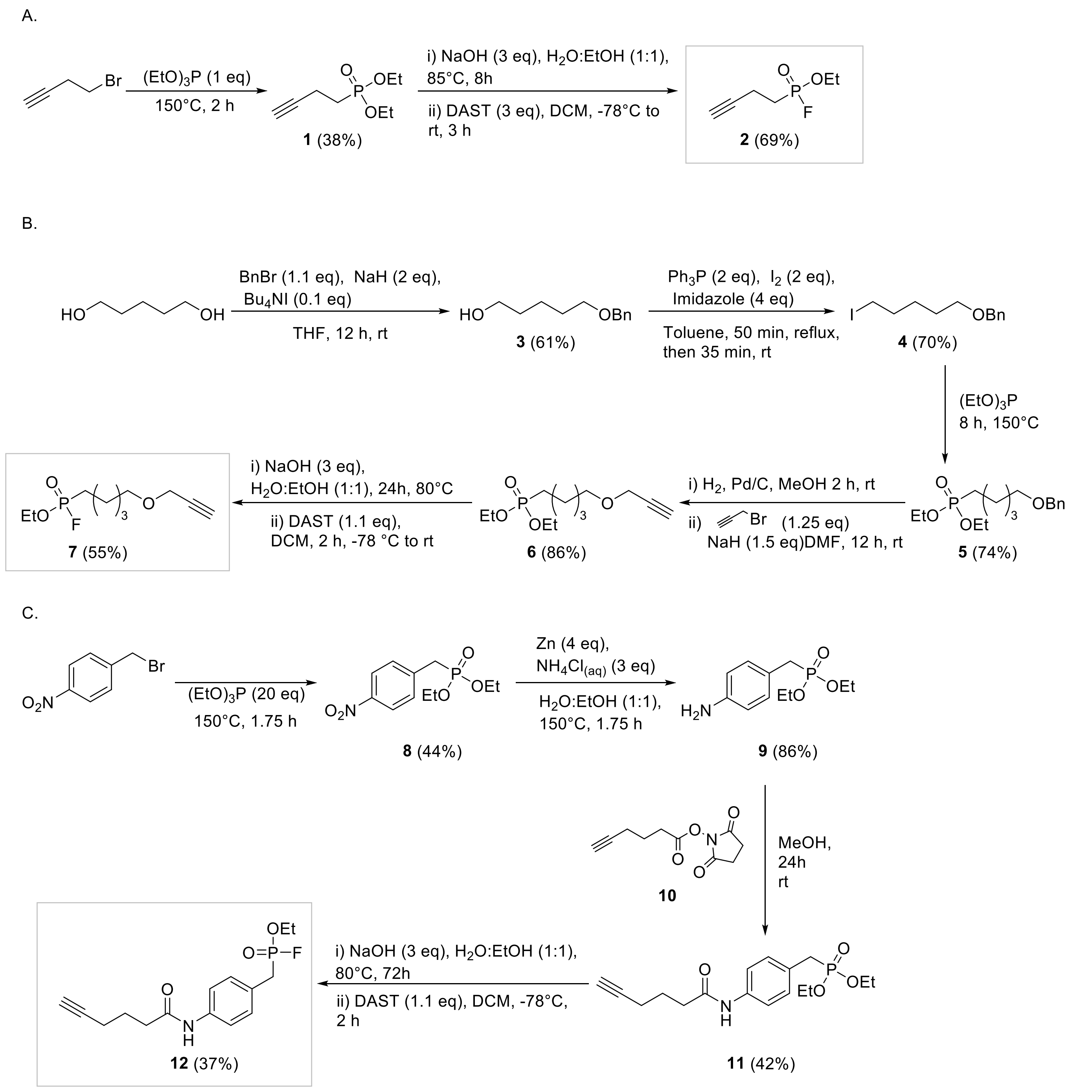

Scheme S1. Synthetic steps to obtain alkyl-FPs **2** (A) and **7** (B); and benzyl-FP **12** (C).

A.

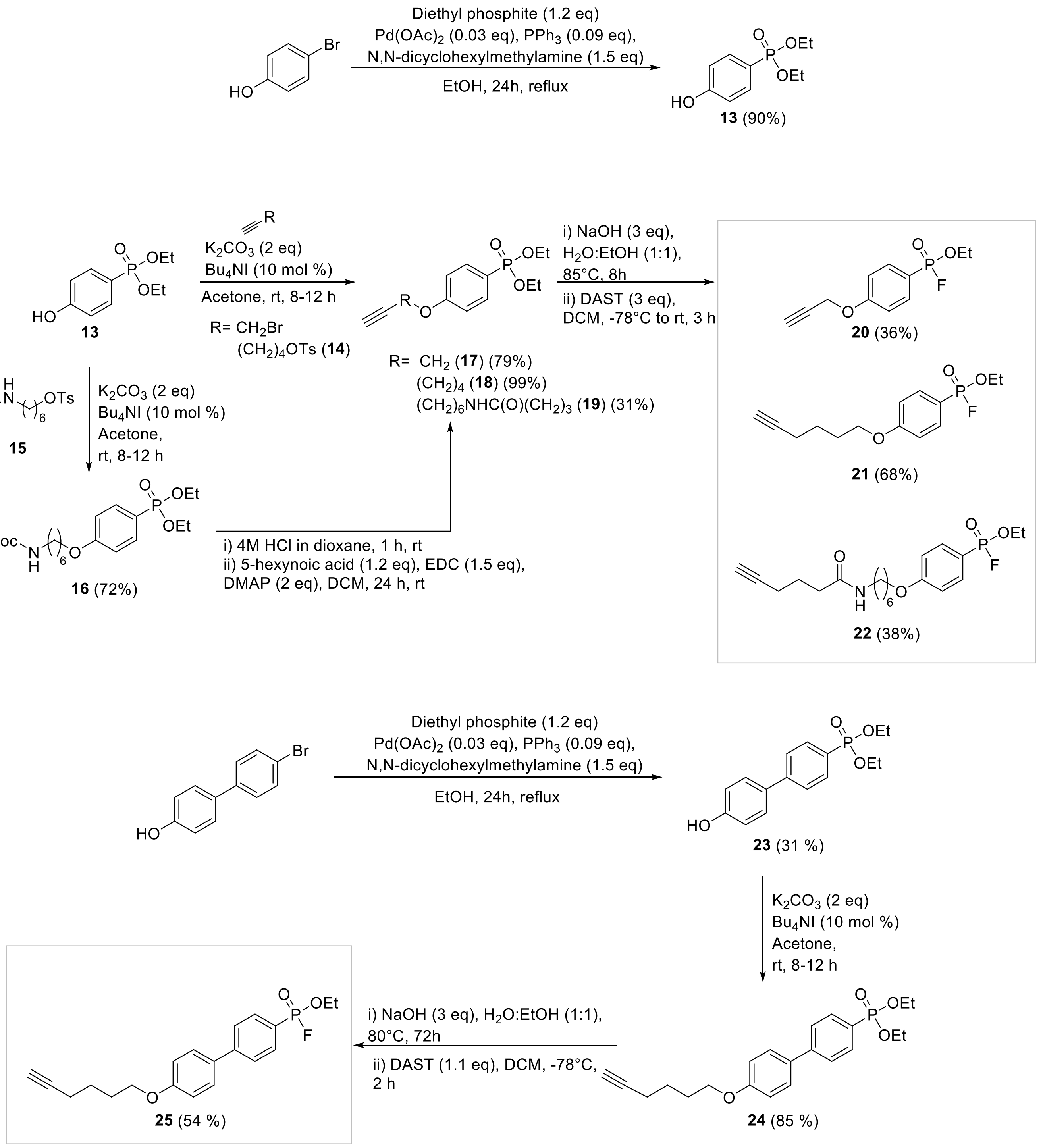

Diethyl phosphite (1.2 eq)
Pd(OAc)2 (0.03 eq), PPh3 (0.09 eq),
N,N-dicyclohexylmethylamine (1.5 eq)
EtOH, 24h, reflux
13 (90%)
K2CO3 (2 eq)
Bu4NI (10 mol %)
Acetone, rt, 8-12 h
R= CH2Br
(CH2)4OTs (14)
R= CH2 (17) (79%)
(CH2)4 (18) (99%)
(CH2)6NHC(O)(CH2)3 (19) (31%)
i) NaOH (3 eq),
H2O:EtOH (1:1),
85°C, 8h
ii) DAST (3 eq),
DCM, -78°C to rt, 3 h
20 (36%)
21 (68%)
22 (38%)
15
K2CO3 (2 eq)
Bu4NI (10 mol %)
Acetone,
rt, 8-12 h
16 (72%)
i) 4M HCl in dioxane, 1 h, rt
ii) 5-hexynoic acid (1.2 eq), EDC (1.5 eq),
DMAP (2 eq), DCM, 24 h, rt
Diethyl phosphite (1.2 eq)
Pd(OAc)2 (0.03 eq), PPh3 (0.09 eq),
N,N-dicyclohexylmethylamine (1.5 eq)
EtOH, 24h, reflux
23 (31 %)
K2CO3 (2 eq)
Bu4NI (10 mol %)
Acetone,
rt, 8-12 h
24 (85 %)
i) NaOH (3 eq), H2O:EtOH (1:1),
80°C, 72h
ii) DAST (1.1 eq), DCM, -78°C,
2 h
25 (54 %)

B.

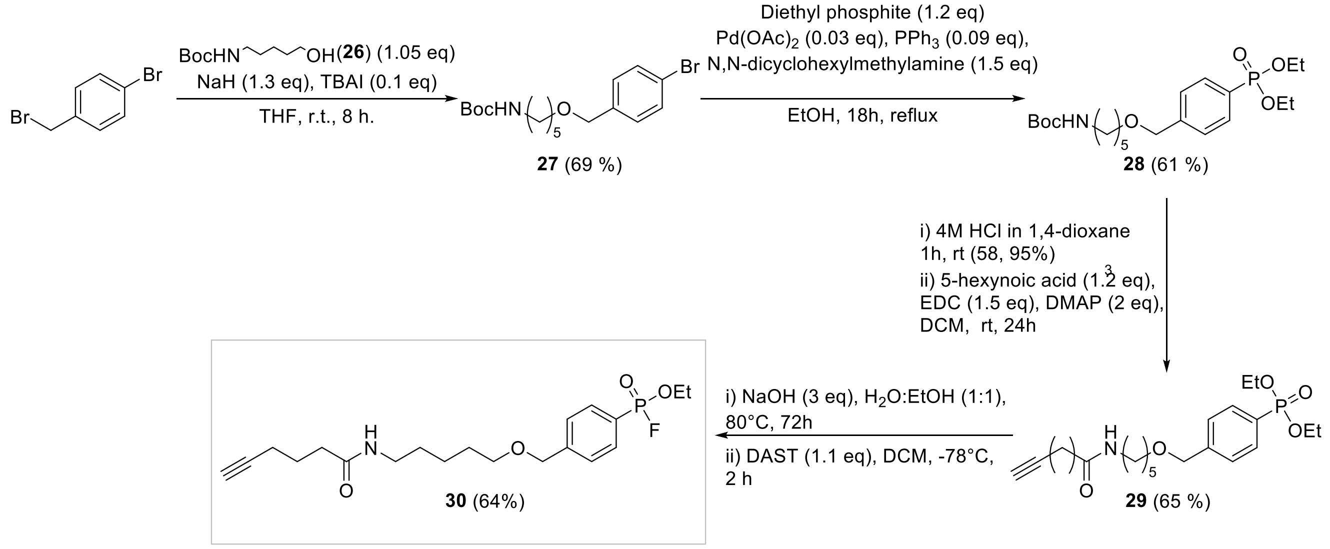

C.

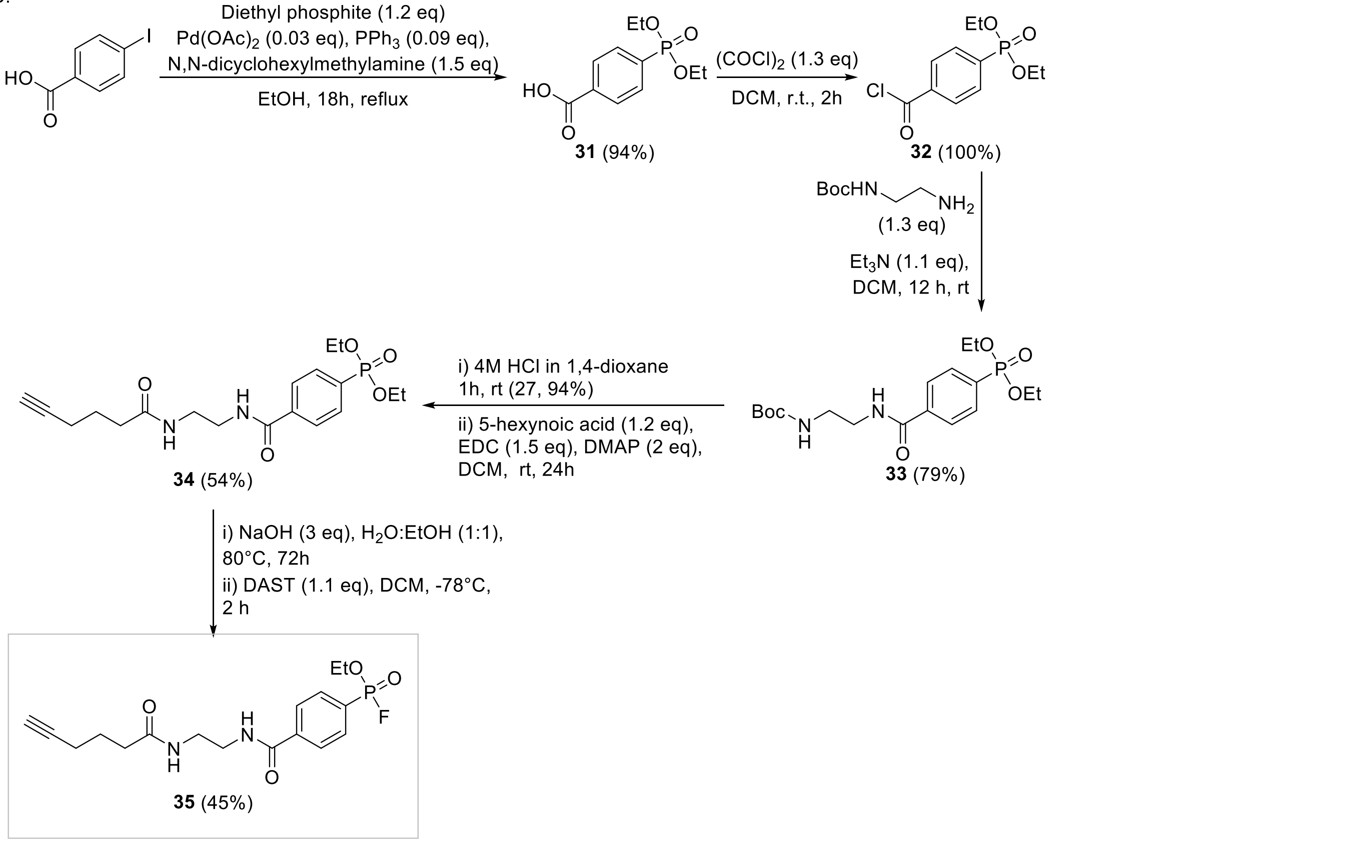

Scheme S2: Synthetic routes to aryl-FPs **20-22** and **25** (A); **30** (B) & **35**(C).

Supplementary Figures S1-S7

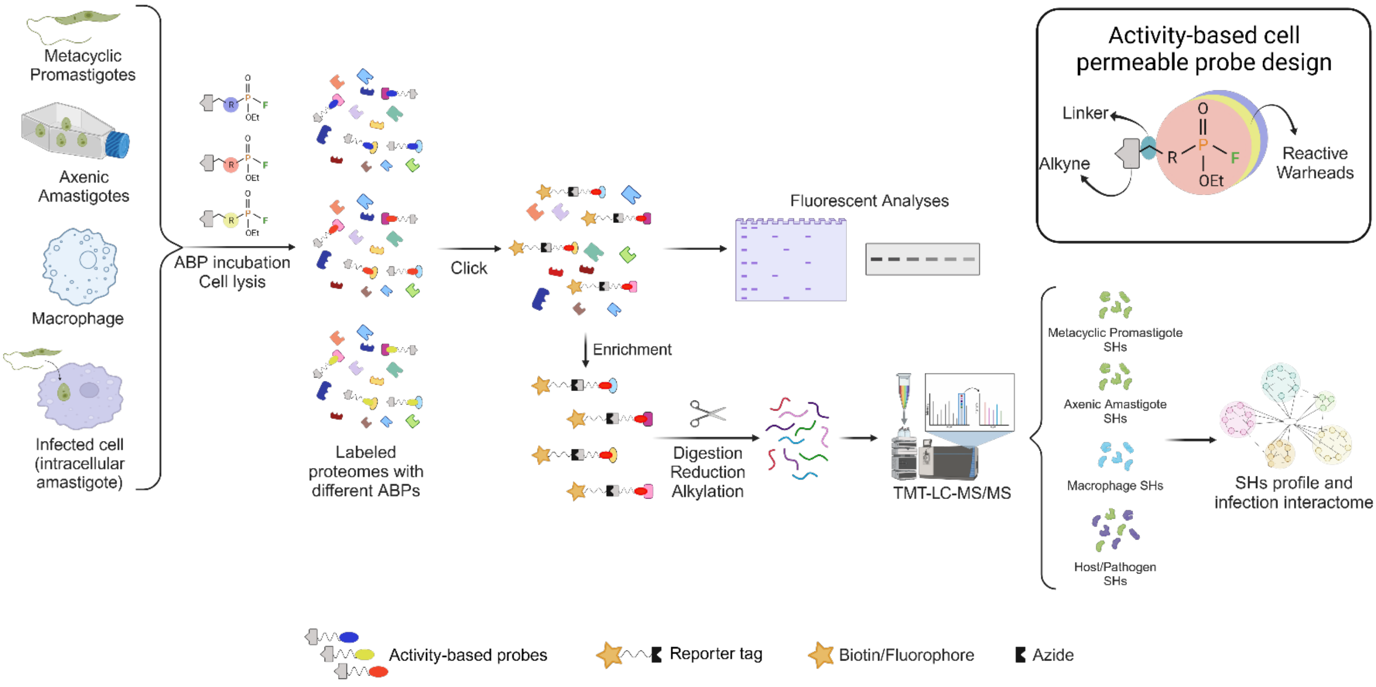

Figure S1 – General ABPP Workflow. Different cell culture systems (*Leishmania* metacyclic promastigotes, axenic amastigotes, uninfected RAW macrophages, and infected RAW macrophages) are incubated with cell permeable FP-probes. Following cell lysis, fluorescent and biotinylated tags are attached to the probes via click chemistry. The labelled proteomes are then either directly visualized by SDS-PAGE and in-gel fluorescence scanning or enriched through affinity purification using streptavidin beads for biotin-tagged probes. Enriched enzymes are digested into peptides, labelled with isobaric tags (TMT), and identified by liquid chromatography-tandem mass spectrometry (LC-MS/MS), enabling the characterization of active enzymes and their regulation under various biological conditions. *Created in BioRender. Isern, J. (2025) https://BioRender.com/9hwp3bk*

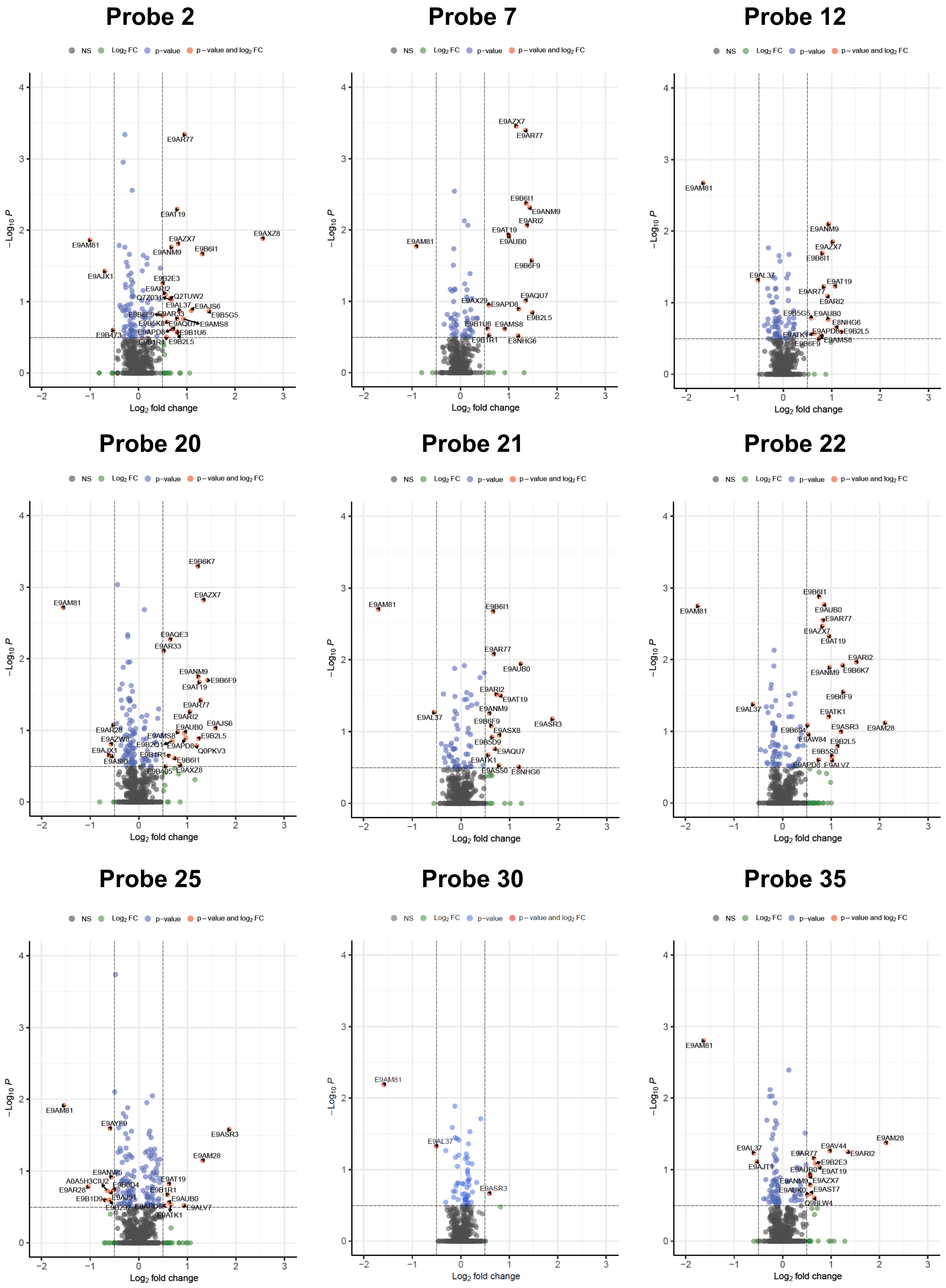
Probe 2
Probe 7
Probe 12
Probe 20
Probe 21
Probe 22
Probe 25
Probe 30
Probe 35
NS
Log_2 FC
p–value
p–value and log_2 FC
$-Log_{10}$ P
Log_2 fold change

Figure S2 - Volcano plots of ABPs. Labelling performance of 9 serine hydrolase (SH) activity-based probes (ABPs) at 15 μM in *L. mexicana* promastigotes (final protein concentration of 1.5 mg/mL) was quantitatively compared with no-probe treatments (DMSO) using activity-based protein profiling (ABPP) followed by tandem-mass-tagging (TMT)-labelling-based quantitative proteomic mass spectrometry (MS). All experiments were performed in biological replicate. A modified *t*-test with permutation-based FDR statistics (250 permutations, FDR = 0.05) was applied to compare probe treatments versus control groups. In the volcano plots, the x-axis represents enrichment on a $\log_2$ scale, while the y-axis shows the $-\log_{10} P$ value. The cut-off values selected were $\log_2$ FC > 0.5 and $-\log_{10} P$ value > 0.3. Positive targets of the probes that passed these cut-off values are shown on the right-hand side as red-filled circles, with the identified *L. mexicana* SHs annotated using their UniProt accession numbers. Green values correspond to targets with $-\log_{10} P$ value below the selected cut-off but a $\log_2$ FC > 0.5. Conversely, blue values represent targets with a $\log_2$ FC < 0.5 but a $-\log_{10} p$ value above the cut-off range. The curated SHs are listed in **Figure 3**.

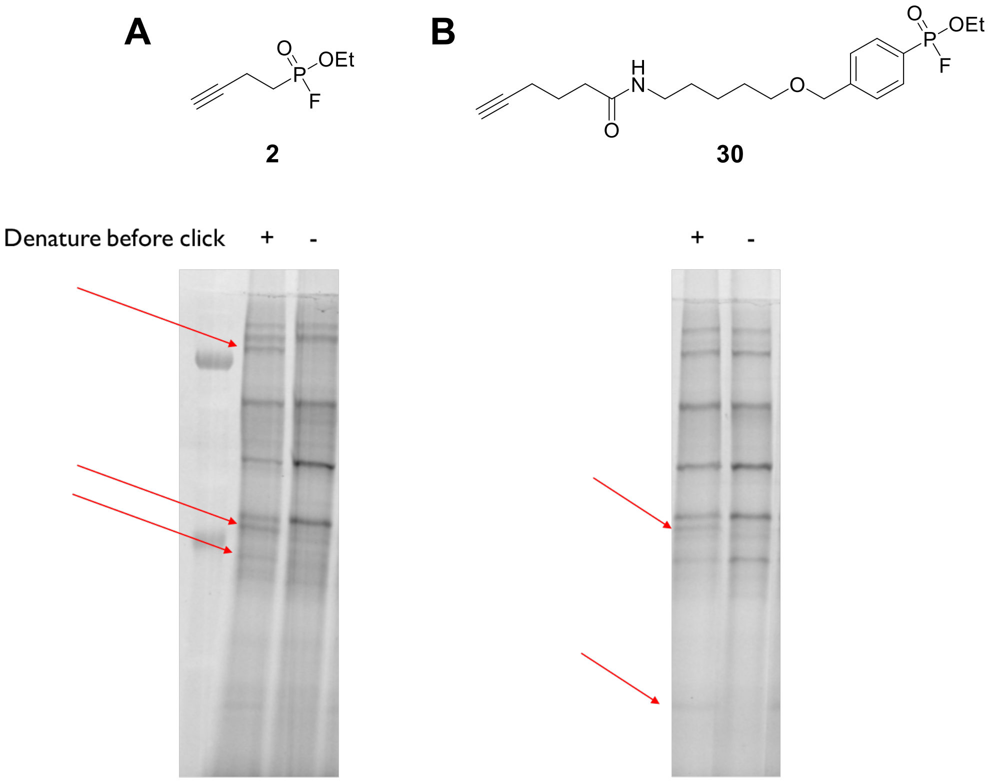

Figure S3 - Evaluation of alkyne accessibility for biorthogonal click reaction. This assay was performed to analyse the relationship between linker length and alkyne handle accessibility for subsequent click chemistry reactions with the N_3-tag. Briefly, *L. mexicana* promastigote lysates were incubated with probe **2** and **30** (15 µM) for 15 minutes at room temperature. Subsequently, one of the lysates was denatured (95 °C for 5 minutes) before performing the click reaction following the general procedure. The resulting fingerprints were analysed using SDS-PAGE followed by in-gel fluorescence detection. **A:** In-gel fluorescence analysis (emission at 560 nm) of the SH fingerprint from *L. mexicana* lysates (1.5 mg/mL) labelled with probe **2** (15 µM). Lane 2: denatured lysate before click chemistry; Lane 3: non-denatured lysate before click chemistry. **B:** In-gel fluorescence analysis (emission at 560 nm) of the SH fingerprint from *L. mexicana* lysates (1.5 mg/mL) labelled with probe **30** (15 µM). Lane 1: denatured lysate before click chemistry; Lane 2: non-denatured lysate before click chemistry. In both cases, the red arrows indicate bands present in the denatured lysate that are absent under non-denatured conditions.

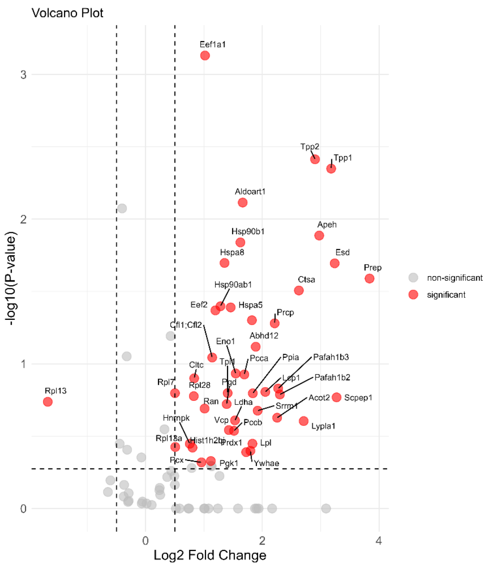

Figure S4 - SHs identified in murine RAW macrophage cells. Following the described general procedures (*vide infra* – see Material and Methods), the SH profile of macrophages was identified. A modified *t*-test with permutation-based FDR statistics (250 permutations, FDR = 0.05) was applied to compare probe treatments versus control groups. In the volcano plots, the x-axis represents enrichment on a $\log_2$ fold change scale, while the y-axis shows the $-\log_{10} P$ value. The selected cut-off values were $\log_2$ FC > 0.5 and $-\log_{10} P$ value > 0.3. Positive targets of probe **7** that passed the cut-off values are depicted on the right-hand side as red filled circles, and the identified murine RAW macrophage SHs are annotated with their gene names. Values in grey correspond to targets below the cut-off range. Volcano plots reveal a significant enrichment of 14 SHs in murine RAW macrophage cells treated with 10 µM of probe **7** compared to untreated cells. The curated SHs are listed in **Table S2**.

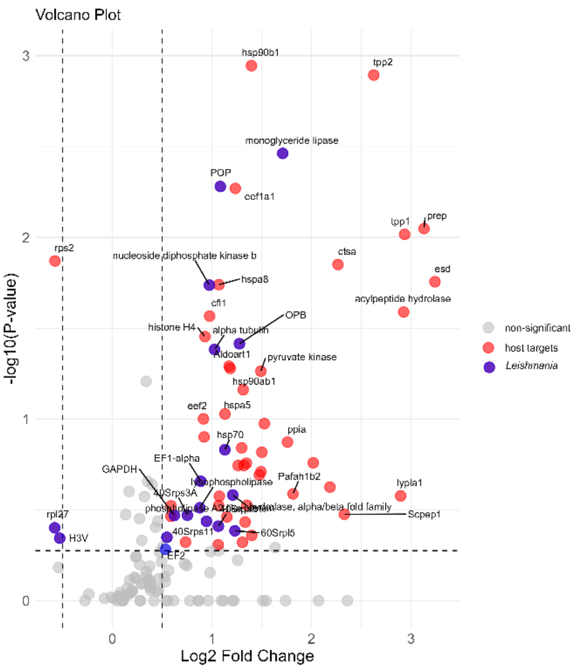

Figure S5 - Probe-oriented interactome (RAW cell + *L. mexicana* + Probe) vs Control (RAW cell + *L. mexicana* + DMSO). Following the described general procedures (*vide infra* – see Material and Methods), the infection interactome was determined using probe **7**, and the SHs where identified. A modified *t*-test with permutation-based FDR statistics (250 permutations, FDR = 0.05) was applied to compare the probe treatments versus control groups. In the volcano plots, the x-axis represents enrichment on a $\log_2$ fold change scale, while the y-axis represents the $-\log_{10}$ *P* value. The selected cut-off values were $\log_2$ FC > 0.5 and $-\log_{10}$ *P* value > 0.3. Positive targets that passed these cut-off values are depicted on the right-hand side. Blue circles represent *L. mexicana* targets. Red circles represent murine host cell targets. Values in grey correspond to targets below the cut-off range. The volcano plots reveal significant enrichment of SHs in the host-parasite interactome between *L. mexicana* and murine RAW macrophage cells treated with 10 µM of probe **7**, compared to the untreated host-parasite system. The curated SHs identified are listed in **Tables 1** and **2**.

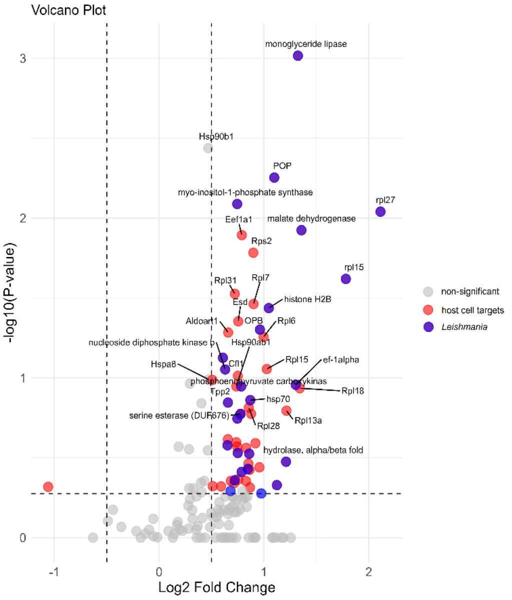

Figure S6 - Infection-specific Interactome [(RAW cell + *L. mexicana* + Probe) vs. (RAW cell + Probe)]. The infection-specific SHs of macrophages were determined by comparing the SHs of infected macrophages (RAW cell + *L. mexicana* + Probe **7**) versus non-infected macrophages (RAW cell + Probe **7**). After following described procedures (*vide infra* – see Material and Methods), the volcano plots reveal significant enrichment of SHs in the host-parasite interactome between *L. mexicana* and murine RAW macrophage cells treated with 10 μM of probe **7**, compared to non-infected RAW macrophage cells. Positive targets that passed the cut-off values are depicted on the right-hand side. Blue circles represent *L. mexicana* targets. Red circles represent host cell targets. Values in grey correspond to targets below the cut-off range. The curated SHs identified are listed in **Tables 1** and **2**.

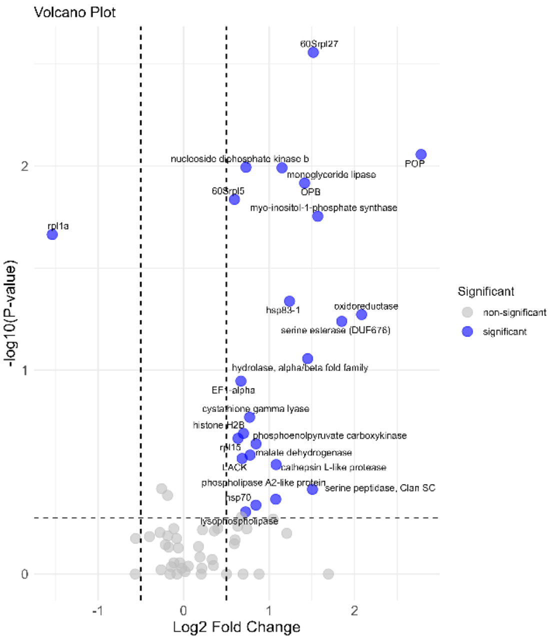

Figure S7 – Volcano plot of competitive ABPP with ZPP and probe 7. The effect of Z-pro-prolinal (ZPP) treatment on the SHs of *Leishmania* parasites was assessed. Following the described procedures (*vide infra* – see Material and Methods), SH activities were detected by probe **7** (10 μM) in untreated parasites (DMSO) and compared to parasites pre-treated with 100 μM ZPP for 2 hours at 26 °C. The volcano plot illustrates the variation in the activity of enzymes inhibited by ZPP treatment. Positive *L. mexicana* targets are depicted on the right-hand side as blue-filled circles, whilst grey-filled circles correspond to targets below the preestablished cut-off ranges. The curated SHs identified are listed in **Table 3**.

Supplementary Tables S1-S4

Table S1 - Docking scores of the top hit of each compound were calculated against each protein target using the ChemPLP scoring function in GOLD.[1,2] While higher ChemPLP scores often indicate stronger predicted binding affinities, reflecting more favourable protein-ligand interactions, higher level molecular dynamics simulations are usually required to establish a clear correlation between experimental and computational binding affinities.[3,4]

	Probes							
**Protein ID**	**2**	**7**	**12**	**20**	**21**	**22**	**25**	**30**
**E9AMS8**	86.9	98.1	75.3	82.6	90.8	98.9	91.5	98.0
**E9ANM9**	89.7	89.4	73.0	82.7	92.4	90.3	96.7	116.2
**E9APD8**	96.7	92.9	87.1	82.2	89.1	104.0	91.6	104.3
**E9AQU7**	39.6	31.9	20.2	26.9	37.6	53.5	50.6	59.5
**E9AR77**	92.0	97.8	74.4	88.2	94.4	103.3	95.0	110.4
**E9ARI2**	93.1	96.7	81.1	93.5	96.8	102.7	82.8	107.8
**E9AX29**	84.9	83.0	71.5	79.2	91.0	100.3	81.6	101.5
**E9AZX7**	99.8	105.4	79.3	89.7	98.8	114.1	115.8	111.9
**E9B1R1**	90.2	71.8	80.5	74.1	88.1	79.7	60.4	102.3
**E9B2L5**	83.3	88.9	70.6	76.0	79.9	99.8	96.6	103.8
**E9B6F9**	81.7	85.2	71.8	70.3	76.3	91.6	85.9	105.5
**E9B6I1**	98.4	98.0	83.3	91.6	102.8	115.9	110.7	120.1
**E9AT19**	79.8	83.7	77.0	66.4	72.3	95.8	73.3	98.9
**E9AUB0**	84.3	84.3	66.7	65.8	74.6	83.6	77.8	89.3

Table S2 – SHs labelled in RAW cells using probe 7. Curated list of SHs identified in murine macrophages (RAW cells) through treatment with Probe 7 (10 μM) following TMT LC-MS/MS analysis.

Gene	Enzyme	-Log_{10} *P*	Log_2 FC	Protein IDs	MW [kDa]
Tpp2	Tripeptidyl-peptidase 2	2.413	2.904	Q3TW28; Q3U4M7; Q3TB11; A0A087WRC0; Q05DJ9; Q3V451; Q3V3M8; A0A087WQR6; Q922K4; A0A087WP19	138.460
Prep	Prolyl endopeptidase	1.590	3.838	Q543B9; Q8C6A3; Q3TCS0; Q3TQ97	80.751
Apeh	Acylamino-acid-releasing enzyme (AARE)	1.887	2.975	A0A0R4J107; A0A0A6YXV0	79.704
Ctsa	Carboxypeptidase	1.507	2.627	Q9D2D1; Q544R6; G3X8T3; A2A5J9; A2A5J8	53.829
Lpl	Lipoprotein lipase	0.448	1.831	Q3U5U0; Q3UBE9; Q8C562; Q4FJQ8; Q3UCZ2; Q3UCH4;Q3UCD4;Q3UC81;Q3UC44;Q3UB98;Q3UB54;Q3U841;Q3U784;Q3U715;Q3UAX2;Q3U6S8;Q3U831;Q3U9P4	53.149

Prcp	Prolylcarboxypeptidase (angiotensinase C)	1.280	2.212	A0A140LHY2	51.064
Scpep1	Carboxypeptidase	0.768	3.273	Q99J29;Q3U5P4	50.958
Acot2	Acyl-CoA thioesterase 2	0.627	2.255	Q6P2K2;A0A1Y7VMZ4;Q91YQ6;B2RTE4;Q99L15;Q14DI6;Q53YL1	49.640
Tpp1	Peptidase S53 domain-containing protein	2.349	3.180	Q8BNF3;Q3U2Z6;Q3TDY6	34.522
Abhd12	Lysophosphatidylserine lipase ABHD12	1.118	1.887	D6RFU2;F7BHM8;D6RI21	33.335
Esd	S-formylglutathione hydrolase	1.694	3.241	H3BLJ9;H3BJL6;H3BKH6;H3BJP2;H3BK43;H3BJC6;H3BL99	28.553
Lypla1	Acyl-protein thioesterase 1	0.604	2.709	J3QP56;D3YUG4;Q4FK51;D3Z111;D3Z269	23.726
Pafah1b3	1-alkyl-2-acetylglycerophosphocholine esterase	0.830	2.272	D3Z7E6;Q3TJC2;D3Z2X5;Q8CA83	20.149
Pafah1b2	1-alkyl-2-acetylglycerophosphocholine esterase	0.788	2.301	Q8BTA2;A0A1L1SVK0;A0A1L1SRD0;A0A1L1SQ76;A0A1L1SQV7;A0A1L1SSQ5	16.614

Table S3 – Proteins enriched in competitive ABPP with ZPP and probe 7. List of proteins differentially enriched in *L. mexicana* parasites treated with ZPP (100 μM) and identified by TMT LC-MS/MS following the described procedures (*vide infra* – see Material and Methods) using probe **7** (10 μM).

**Protein IDs**	**Gene**	**Protein name**	**-log (P-value)**	**Log2 FC**
E9AUB0	LmxM.36.6750	POP	2.057	2.777
Q25222;E9ATK1;A1Y2C5;A1Y2C4	LmxM.36.4170	Oxidoreductase	1.272	2.082
E9B066	LmxM.28.2445	Serine esterase (DUF676)	1.239	1.850
Q95PT4;E9APY4;O00815	LmxM.14.1360	Myo-inositol-3-phosphate synthase	1.754	1.569
E9B350;E9B348	LmxM.31.2710, LmxM.31.2690	60S ribosomal protein L27 60Srpl27	2.557	1.517
E9AT19	LmxM.36.2420	Serine peptidase, Clan SC	0.415	1.507
E9AZX7	LmxM.28.1570	Hydrolase, alpha/beta fold family	1.056	1.451
E9AMS8;A7XAB0	LmxM.09.0770	OPB	1.917	1.415
E9B3L2	LmxM.32.0312/14/16	Heat shock protein hsp83-1	1.338	1.239
E9B1R1	LmxM.30.1140	Monoglyceride lipase	1.991	1.151
E8NHH6;Q95ZD6;P92185;E9AMH7;E8NHD8;E9AMH5;E8NHS3;Q7JMY2;E9AMH6;E8NHJ1;E8NHL3;Q25318;E8NHH7	LmxM.08.1080	Cathepsin L-like protease	0.537	1.082
E9B6I1	LmxM.34.3020	Phospholipase A2-like protein	0.367	1.077
E9AZ81;E9AZ83	LmxM.27.1805/10/07	Phosphoenolpyruvate carboxykinase	0.638	0.847
E9B099	LmxM.28.2770	Putative heat-shock protein hsp70	0.338	0.846
E9B4I1	LmxM.33.0140	Malate dehydrogenase	0.583	0.778
E9B6K2	LmxM.34.3230	Putative cystathione gamma lyase	0.769	0.772

E9B376;A0A1Y0DDB3	LmxM.31.2950/51	Nucleoside diphosphate kinase b	1.994	0.729
E9AX29	LmxM.24.1840	Lysophospholipase	0.305	0.725
E9AZI2	LmxM.28.0210	Histone H2B	0.689	0.700
E8NHN2	LmxM.28.2740	Guanine nucleotide-binding protein subunit beta-like protein LACK	0.567	0.684
I3V666;E9ARD0;E9ARD5	LmxM.17.0082/80/85	Elongation factor 1-alpha EF1-alpha	0.946	0.671
E9B1D9;E9B668	LmxM.29.3650, LmxM.34.1910	Ribosomal protein rpl15	0.665	0.637
E9B665	LmxM.34.1880/1890/1900	Putative 60S ribosomal protein 60Srpl5	1.836	0.595
E9ALW5;E9ALW4	LmxM.08_29.1070/90	Putative ribosomal protein L1a rpl1a	1.664	-1.536

Table S4 - ^{31}P and ^{19}F chemical shifts of synthesised FP ABPs.

Probe	Nature	Electronic effect	^{31}P (ppm)	^{19}F (ppm)	*J* coupling
**20**	Aryl	EDG (strong)	17.7	-63.5	1035
**21**	Aryl	EDG (strong)	18.3	-63.7	1033
**22**	Aryl	EDG (strong)	18.3	-63.7	1033
**25**	Aryl	EDG (weak)	17.6	-63.8	1040
**30**	Aryl	EDG (weak)	17.2	-63.9	1041
**35**	Aryl	EWG (strong)	15.6	-58.8	1045
**2**	Alkyl	-	28.1	-63.6	1070
**7**	Alkyl	-	31.5	-64.6	1069
**12**	Benzyl	EDG (moderate)	25.0	-64.5	1079

Materials and Methods

General Conditions

All solvents and reagents were purchased from commercial suppliers. NMR spectra were recorded on the following instruments: Bruker Neo 700 MHz spectrometer with operating frequencies of 699.73 MHz for ^{1}H, 175.95 MHz for ^{13}C, 658.41 MHz for ^{19}F, and 283.25 MHz for ^{31}P; Varian VNMRS-600 spectrometer with operating frequencies of 599.42 MHz for ^{1}H, 150.72 MHz for ^{13}C, 564.02 MHz for ^{19}F, 242.65 MHz for ^{31}P; Bruker Neo-400 spectrometer with operating frequencies of 400.20 MHz for ^{1}H, 100.63 MHz for ^{13}C, 376.57 MHz for ^{19}F, 162.00 MHz for ^{31}P; Bruker Avance III-HD-400 spectrometer with operating frequencies of 399.95 MHz for ^{1}H, 100.57 MHz for ^{13}C, 376.33 MHz for ^{19}F, 161.90 MHz for ^{31}P; Bruker Avance III-HD-400 spectrometer with operating frequencies of 400.07 MHz for ^{1}H, 100.60 MHz for ^{13}C, 376.45 MHz for ^{19}F, 161.95 MHz for ^{31}P. Spectra were referenced relative to $CDCl_3$ (δ_H 7.26 ppm, δ_C 77.16 ppm), DMSO-d6 (δ_H 2.50 ppm, δ_C 39.52 ppm) or CD_3OD (δ_H 4.87 ppm, δ_C 49.00 ppm), D_2O (δ_H 4.79 ppm) Chemical shifts are reported in parts per million (ppm), coupling constants (*J*) in hertz (Hz) and multiplicity as singlet (s), doublet (d), triplet (t), quartet (q), multiplet (m) or a combination thereof. All ^{1}H NMR and ^{13}C NMR spectral assignments were made with the aid of ^{1}H-^{1}H COSY, ^{1}H-^{13}C HSQC and ^{1}H-^{13}C HMBC NMR experiments. Infra-red spectra were recorded on a PerkinElmer Frontier FTIR spectrometer equipped with a Specac Quest ATR accessory with extended range diamond puck. IR assignments are reported in wavenumbers (cm^{-1}). Thin layer chromatography was performed using Merck F_{254} silica gel 60 aluminium sheets pre-coated with silica gel. High-resolution mass spectrometry (HRMS) and liquid chromatography mass spectrometry (LCMS) were recorded on a Waters TQD mass spectrometer ESI-LC water (0.1 % formic acid): MeCN, flow rate 0.6 mL min-1 with a UPLC BEH C18 1.7 µm (2.1 mm × 50 mm) column.

General Methods

General procedure A – Michaelis-Arbuzov reaction

The stated aryl/alkyl halide (1 eq) was added to triethylphosphite (10-20 eq) in a round bottom flask at room temperature. The reaction mixture was heated at 140-160 °C overnight under an Ar atmosphere. Subsequently, it was diluted with EtOAc and washed with H_2O and brine. The organic layer was dried ($MgSO_4$), and the volatiles were removed under high vacuum (24-72 h) to afford the desired phosphonate.

General procedure B - Aryl phosphonate synthesis (Hirao cross coupling reaction)

A round bottom flask charged with dry EtOH (volume adjusted to achieve 0.2 M limiting reactant), $Pd(OAc)_2$ (0.03 eq), and PPh_3 (0.09 eq) was degassed and stirred at 90 °C for 30 min. under N_2. Subsequently, aryl halide (1 eq), *N,N*-dicyclohexylmethylamine (1.5 eq) and diethyl phosphite (1.2 eq) were added via syringe, and the reaction mixture was stirred at 90 °C overnight. Upon completion, the volatiles were removed, and the remaining residue was then taken in EtOAc. The remaining residue was then washed with 1 M HCl, H_2O, and brine, dried ($MgSO_4$) and purified by flash chromatography to afford the desired phosphonate.

General procedure C – Williamson etherification

Anhydrous K_2CO_3 (2.0 eq) was added to a solution of the stated hydroxy aryl phosphonate (1.0 eq) in dry acetone (5 mL), and the resulting mixture was stirred for 0.5 h. Subsequently the tosylated alkyne (1.2 eq) and TBAI (10 mol %) were added to the solution, which was stirred for an additional 12-36 h at reflux temperature under N_2. The residue obtained after removing the volatiles was extracted with EtOAc and washed with water and brine. The organic layer was then dried ($MgSO_4$) and purified by flash chromatography with the stated solvent system to afford the desired ether.

General procedure D – Fluorophosphonate synthesis

NaOH (2-3 eq) was added to a solution of phosphonate intermediate (1 eq) in either H_2O or H_2O:EtOH (1:1) (0.05 – 0.2 M), and the mixture was stirred at 80-85 °C until no starting material was observed by TLC (2-72 h). It was then concentrated to remove the EtOH, diluted with water, acidified with 1N HCl, and extracted with EtOAc. The combined organic extracts were concentrated, and the resultant crude product was dissolved in DCM (0.05 – 0.2 M), cooled to −78 °C and DAST (2.5 eq) was added dropwise. The mixture was allowed to warm to room temperature over 2 h and stirred at this temperature for a further 1 h. It was then diluted with EtOAc and washed with H_2O and brine. The organic extract was then dried ($MgSO_4$) and concentrated. Purification by flash chromatography with the solvent system (Hex:EtOAc or DCM:MeOH gradients), stated for each individual reaction, afforded the desired fluorophosphonate.

General procedure E -Acyl chloride synthesis

To a stirred solution of carboxylic acid (1.0 eq) in DCM (0.2M), 2 drops of DMF, and oxalyl chloride (1.3 eq) was added dropwise. The mixture was then stirred at room temperature for 2 h under N_2, after which it was concentrated to proceed with the next synthetic step without further purification.

General procedure F - *N*-Boc deprotection

The *N*-Boc carbamate (1.0 eq) was treated with a solution of 4M HCl in dioxane (8.4 mL) under N_2 at room temperature for 1 h. Subsequently, the mixture was concentrated *in vacuo* to afford the deprotected amine hydrochloride salt. The product was used directly without further purification.

General procedure G – Amide coupling.

A round bottom flask charged with dry DCM (0.2 M), carboxylic acid (1.2 eq), EDC (1.5 eq), and DMAP (2.0 eq) was degassed and stirred at 50 °C for 30 min under N_2. Subsequently, the amine (1.0 eq) was added, and the reaction was heated under reflux for a further 19 h. The crude reaction mixture was then washed with 1N HCl and brine; dried (Mg_2SO_4) and the volatiles were removed under high vacuum to afford the desired phosphonate. When stated, purification by flash chromatography using the stated solvent system afforded the desired amide.

General procedure Gb – Amide coupling.

A solution of acyl chloride (1.0 eq) in DCM (0.1-0.2 M) was added dropwise to a round-bottom flask containing the coupling amine (1.2 eq) and Et_3N (1.1 eq) in dry DCM (0.1-0.2 M) under N_2. The mixture was left to react at room temperature overnight. Upon completion, it was washed with H_2O and saturated aqueous $NaHCO_3$ solution, dried ($MgSO_4$), and the volatiles removed under high vacuum to afford the desired amide**.** When stated, purification by flash chromatography using the stated solvent system afforded the desired amide.

Experimental Procedures

Diethyl (but-3-yn-1-yl)phosphonate (1)

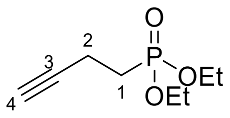

A mixture of $P(OEt_3)$ (1.0 mL, 0.006 mol, 1 eq) and 4-bromo-1-butyne (1.2 mL, 0.01 mol, 2.1 eq) was heated at 150 °C for 2.5 h. The reaction mixture was then cooled to room temperature and after the volatiles were removed, compound **1** (412 mg, 38%) was obtained as a yellow oil. ν_{max} (ATR) 3311 (C-H alkyne), 2244 (C≡C), 1242 (P=O) cm^{-1}. δ_H NMR (400 MHz, $CDCl_3$) 4.19 – 4.01 (4H, m, -OC***H*₂**CH₃), 2.47 (2H, m, 1-***H₂***), 2.05 – 1.93 (3H, m, 4-***H***, 2-***H₂***), 1.32 (6H, t, *J* 7.0, -OCH₂C***H₃***). δ_C NMR (101 MHz, $CDCl_3$) 82.8 (d, $^3J_{C-P}$ 20.6, ***C***-3), 69.2 (d, $^4J_{C-P}$ 1.8, ***C***-4), 61.9 (d, $^2J_{C-P}$ 6.5, -O***C***H₂CH₃), 25.34 (d, $^1J_{C-P}$ 142.0, ***C***-1),

16.6 (d, $^3J_{C-P}$ 6.0, -OCH$_2$**C**H$_3$), 12.7 (d, $^2J_{C-P}$ 3.6, **C**-2). δ_P NMR (162 MHz, CDCl$_3$) 28.9. m/z LCMS (ES$^+$) 191.2 [M+H]$^+$. All the data are in accordance with the literature.[5]

Ethyl (but-3-yn-1-yl)phosphonofluoridate (2)

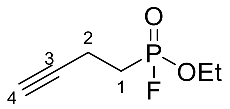

NaOH (64 mg, 1.6 mmol, 3 eq) was added to a solution of **2** (100 mg, 0.53 mmol, 1 eq) in 1:1 H_2O:EtOH (4.0 mL), and the mixture was left to react at 85 °C for 8 h. Subsequently, the mixture was concentrated to remove the EtOH, diluted with water (1 mL), acidified with 1N HCl (1 mL), and extracted with EtOAc (3 × 25 mL). The combined organic extracts were then washed with brine (1 × 5 mL), and concentrated. The resulting crude product was dissolved in dry DCM (4.0 mL), cooled to −78 °C, and DAST (0.146 mL, 1.05 mmol, 2 eq) was added dropwise. The mixture was stirred for 3 h at −78°C. After completion, it was quenched with water, the volatiles were removed under vacuum and the product was extracted with EtOAc (25.0 mL), washed with water (1 × 5 mL) and brine (1 × 5 mL), dried, and purified by flash chromatography (Hex:EtOAc 0-100% gradient) to afford the titled alkyl fluorophosphonate **32** (60 mg, 69%) as a yellow liquid. ν_{max} (ATR) 3305 (C-H alkyne), 2248 (C≡C), 1677 (C=O), 1272 (P=O), 1043 (C-O) cm^{-1}. δ_H NMR (599 MHz, CDCl$_3$) 4.35 – 4.23 (2H, m, -OC***H$_2$***CH$_3$), 2.60 – 2.51 (2H, m, 2-***H$_2$***), 2.22 – 2.12 (2H, m, 1-***H$_2$***), 2.05 (1H, t, *J* 2.7, 4-***H***), 1.39 (3H, t, *J* 7.1, -OCH$_2$C***H$_3$***). δ_C NMR (151 MHz, CDCl$_3$) 81.4 (d, $^3J_{C-P}$ 20.1, **C**-3), 70.0 (d, $^4J_{C-P}$ 1.8, **C**-4), 63.8 (d, $^2J_{C-P}$ 7.1, -O**C**H$_2$CH$_3$), 24.2 (dd, $^1J_{C-P}$ 145.8, $^1J_{C-F}$ 24.0, **C**-1), 16.5 (d, $^3J_{C-P}$ 5.6, -OCH$_2$**C**H$_3$), 12.1 (d, $^2J_{C-P}$ 3.5, **C**-2). δ_P NMR (162 MHz, CDCl$_3$) 28.1 (d, $^1J_{P-F}$ 1070). δ_F NMR (376 MHz, CDCl$_3$) -63.6 (d, $^1J_{F-P}$ 1070). HRMS (ES$^+$) found [M+H]$^+$ 165.0476; $C_6H_{10}FO_2P$ requires *M* 165.0481.

5-(Benzyloxy)pentan-1-ol (3)

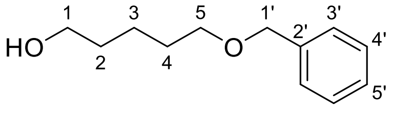

A stirred suspension of NaH (384 mg, 9.60 mmol, 2.00 eq) in dry THF (10 mL) was prepared, to which a solution of pentane-1,5-diol (500 mg, 4.80 mmol, 1.00 eq) in dry THF (10 mL) was added dropwise at room temperature. The reaction mixture was stirred for 30 minutes, followed by the addition of tetrabutylammonium iodide (Bu_4NI) (180 mg, 0.48 mmol, 0.10 eq) and benzyl bromide (903 mg, 5.28 mmol, 1.10 eq). The reaction mixture was stirred overnight at room temperature. After completion, the reaction was quenched by the addition of water (8 mL) and extracted with EtOAc (2 × 50 mL). The combined organic layers were washed successively with water (50 mL) and brine (50 mL), then dried over $MgSO_4$.

The solvent was removed under reduced pressure, and the crude product was purified using column chromatography with a gradient from 100% hexane to 100% EtOAc, affording 5-(benzyloxy)pentan-1-ol (**3**) as a colourless oil (570 mg, 61% yield). ν_{max} (ATR) 3391 (br, -OH) cm^{-1}. δ_H NMR (400 MHz, CDCl$_3$) 7.47–7.30 (5H, m, Ar-***H***), 4.53 (2H, s, -O-C***H***$_2$-Bn), 3.65 (2H, t, ^3J 6.5, 1-***H***$_2$), 3.51 (2H, t, ^3J 6.5, 5-***H***$_2$), 1.75–1.52 (4H, m, CH$_2$), 1.52–1.41 (2H, m, 4-***H***$_2$). δ_C NMR (101 MHz, CDCl$_3$) 138.6 (***C***-2'), 128.4 (***C***-4'), 127.7 (***C***-3'), 127.6 (***C***-5'), 73.0 (***C***-1'), 70.3 (***C***-5), 62.8 (***C***-1), 32.5 (***C***-2), 29.5 (***C***-4), 22.4 (***C***-3). m/z (LCMS ES$^+$) 195.6. All the data is in accordance with the literature.[6]

[(5-iodopentyl)oxy]methylbenzene (4)

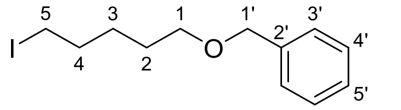

A mixture of 5-(benzyloxy)-1-pentanol (**3**) (300 mg, 1.54 mmol, 1.00 eq), triphenylphosphine (810.0 mg, 3.09 mmol, 2.00 eq), imidazole (420.5 mg, 6.18 mmol, 4.00 eq), and iodine (783.9 mg, 3.09 mmol, 2.00 eq) in toluene (10 mL) was stirred under reflux for 50 minutes. After cooling to room temperature, the solution was treated with aqueous NaHCO$_3$ (25 mL) and stirred at 20°C for 15 minutes. Subsequently, excess iodine was added, and the mixture was stirred for an additional 20 minutes at 20°C. A large excess of Na$_2$SO$_3$ was then added until the iodine colour disappeared. The toluene layer was separated, and the aqueous portion was further extracted with CH$_2$Cl$_2$ (4 × 30 mL), minimizing exposure to light throughout. The combined organic layers were washed with water (50 mL), dried over MgSO$_4$, and the solvents were removed under reduced pressure. The crude product was purified by column chromatography on silica gel. Elution with 0–20% CH$_2$Cl$_2$/Hexane yielded foreruns, followed by further elution with 20–33% CH$_2$Cl$_2$/hexane, which gave compound **4** as a colourless oil (328 mg, 70% yield). ν_{max} (ATR) 3035 (C-H), 2939 (C-H), 1101 (C-O-C) cm^{-1}. δ_H NMR (400 MHz, CDCl$_3$) 7.42–7.26 (5H, m, Ar-***H***), 4.53 (2H, s, O-C***H***$_2$-Ar), 3.51 (2H, t, ^3J 6.4, 1-***H***$_2$), 3.22 (2H, t, ^3J 7.0, 5-***H***$_2$), 1.88 (2H, q, ^3J 7.1 Hz, 2-***H***$_2$), 1.76–1.62 (2H, m, 2-***H***$_2$), 1.58–1.45 (2H, m, 3-***H***$_2$). δ_C NMR (101 MHz, CDCl$_3$) 138.5 (***C***-2'), 128.4 (***C***-4'), 127.7 (***C***-3'), 127.6 (***C***-5'), 73.0 (O-***C***-1'), 70.0 (O-***C***-1), 33.4 (***C***-4), 28.7 (***C***-3), 27.3 (***C***-2), 6.9 (I-***C***-5). HRMS (ES$^+$) found [M+H]$^+$ 305.0412; C$_{12}$H$_{18}$OI requires *M* 305.0397. All the data is in accordance with the literature.[7]

[5-(benzyloxy)pentyl]phosphonate (5)

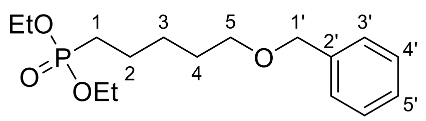

A solution of [5-(iodopentyl)oxy]methylbenzene (**4**) (100 mg, 0.328 mmol, 1.00 eq) in triethyl phosphite (500 µL, 3.00 eq) was heated at 150°C for 8 hours. After cooling to 20°C, the reaction mixture was quenched by the addition of water (8 mL). The resulting mixture was extracted with EtOAc (2 × 10 mL). The combined organic layers were washed successively with water (50 mL) and brine (50 mL), and dried over $MgSO_4$. The solvent was removed under reduced pressure, and the residue was purified by column chromatography with a gradient from 100% hexane to 100% EtOAc, affording diethyl [5-(benzyloxy)pentyl]phosphonate (**5**) as a colourless oil (76 mg, 74%). ν_{max} (ATR) 3033 (C-H), 1242 (P=O), 1095 (C-O-C) cm^{-1}. δ_H NMR (400 MHz, $CDCl_3$) 7.35-7.10 (5H, m, Ar-***H***), 4.52 (2H, s, O-C$\boldsymbol{H_2}$-Ar), 4.23–3.96 (4H, m, -OC$\boldsymbol{H_2}$$CH_3$), 3.49 (2H, t, ^3J 6.4, 5-$\boldsymbol{H_2}$), 1.85–1.69 (2H, m, 1-$\boldsymbol{H_2}$), 1.69–1.56 (4H, m), 1.56–1.42 (2H, m), 1.33 (6H, t, ^3J 7.1, -OCH_2C$\boldsymbol{H_3}$). δ_C NMR (101 MHz, $CDCl_3$) 138.6 (***C***-2'), 128.4 (***C***-4'), 127.6 (***C***-3'), 127.5 (***C***-5'), 72.9 (O-***C***-1'), 70.1 (O-***C***-5), 61.5 (d, $^2J_{C-P}$ 6.5, -O**C**H2CH3), 29.3 (***C***-4), 27.3 (d, $^2J_{C-P}$ 17.0, ***C***-2), 25.7 (d, $^1J_{C-P}$ 141.5, ***C***-1), 22.3 (d, $^4J_{C-P}$ 6.0, ***C***-3),16.5 (d, $^3J_{C-P}$ 6.0, -OCH_2**C**H_3). δ_P NMR (162 MHz, $CDCl_3$) 32.3. HRMS (ES$^+$) found $[M+H]^+$ 315.1708; $C_{16}H_{28}O_4P$ requires *M* 315.1720.

Diethyl [5-(prop-2-yn-1-yloxy)pentyl]phosphonate (6)

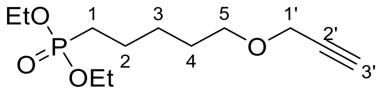

An oven-dried 50 mL flask with a stirrer was charged with diethyl (5-hydroxypentyl)phosphonate (**5**) (50 mg, 0.159 mmol, 1 eq) and palladium (10% w/w on carbon, 18 mg, 0.016 mmol). The flask was sealed with a septum, evacuated, and back-filled with nitrogen (x3). Methanol (2 mL) was added, and the reaction mixture was re-evacuated and back-filled with hydrogen gas (x5). The mixture was stirred under a balloon of hydrogen at room temperature for 2 hours, then purged with nitrogen gas and filtered through a plug of Celite (2 g), eluting with EtOAc (10 mL). The filtrate was concentrated in vacuo to give crude diethyl (5-hydroxypentyl)phosphonate (38 mg, quant.) as a colourless oil, which was used immediately without further purification. Next, 60% NaH dispersion in mineral oil (10 mg, 0.238 mmol, 1.5 equiv) was placed in a round-bottom flask under a nitrogen atmosphere at room temperature. Dried DMF (2 mL) was added, and the suspension was cooled to 0°C. A solution of (5-hydroxypentyl)phosphonate (38 mg, 27 µL, 0.159 mmol, 1 eq) in dried DMF (2 mL) and propargyl bromide (80% in toluene) (30 mg, 0.200 mmol, 1.25 eq) was added dropwise to the suspension at 0°C. The reaction mixture was allowed to warm to room temperature. The reaction progress was monitored by TLC. Upon completion, the mixture was quenched with cooled aqueous NH_4Cl (10 mL) and extracted with EtOAc (3 × 25 mL). The combined organic layers were washed with brine, dried over $MgSO_4$, and concentrated under reduced pressure. The residue was purified by column chromatography using a gradient from 100% hexane to 100% EtOAc, yielding diethyl (5-(prop-2-yn-1-yloxy)pentyl)phosphonate (**6**) as a colourless oil (36 mg, 86%). ν_{max} (ATR) 3303 (C-H

alkyne), 2252 (C≡C), 1239 (P=O) cm^{-1}. δ_H NMR (599 MHz, $CDCl_3$) 4.11 (2H, d, ^4J 2.2, 1'-$\boldsymbol{H_2}$), 4.10–4.02 (4H, m, -OC$\boldsymbol{H_2}$$CH_3$), 3.50 (2H, t, ^3J 6.4, 5-$\boldsymbol{H_2}$), 2.40 (1H, d, ^4J 2.3, 3'-***H***), 1.75–1.68 (2H, m, 1-$\boldsymbol{H_2}$), 1.65–1.58 (4H, m), 1.48–1.42 (2H, m), 1.31 (6H, t, ^3J 7.1, -$OCH_2C$$\boldsymbol{H_3}$). δ_C NMR (151 MHz, $CDCl_3$) 80.1 (O-***C***-2'), 74.3 (O-***C***-3'), 69.9 (O-***C***-5), 61.5 (d, $^2J_{C\text{-}P}$ 6.5, -O***C***H_2CH_3), 58.2 (O-***C***-1'), 29.2 (***C***-4), 27.3 (d, $^2J_{C\text{-}P}$ 17.3, ***C***-2), 25.7 (d, $^1J_{C\text{-}P}$ 140.7, ***C***-1), 22.4 (d, $^3J_{C\text{-}P}$ 5.1, ***C***-3), 16.6 (d, $^3J_{C\text{-}P}$ 6.0, -OCH_2***C***H_3). δ_P NMR (162 MHz, $CDCl_3$) 32.2. HRMS (ES^+) found $[M+H]^+$ 263.1422; $C_{12}H_{24}O_4P$ requires M 263.1407.

Ethyl [5-(prop-2-yn-1-yloxy)pentyl]phosphonofluoridate (7)

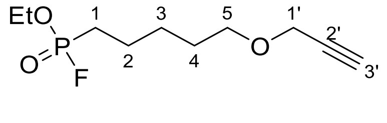

NaOH (14 mg, 0.344 mmol, 3 eq) was added to a solution of (5-(prop-2-yn-1-yloxy)pentyl)phosphonate (**6**) (30 mg, 0.115 mmol, 1 eq) in a 1:1 H_2O mixture (4.0 mL), and the reaction mixture was heated at 85°C for 8 hours. The mixture was then concentrated to remove EtOH, diluted with water (1 mL), acidified with 1N HCl (1 mL), and extracted with EtOAc (3 × 25 mL). The combined organic extracts were washed with brine (1 × 5 mL) and concentrated. The resulting crude product was dissolved in dry DCM (4.0 mL), cooled to −78°C, and DAST (37 mg, 30 µL, 0.230 mmol, 2 equiv) was added dropwise. The mixture was stirred for 3 hours at −78°C. Upon completion, the reaction was quenched with water, and the volatiles were removed under vacuum. The residue was extracted with EtOAc (25 mL), washed with water (1 × 5 mL) and brine (1 × 5 mL), dried, and purified by flash chromatography (Hexane:AcOEt, 0–100% gradient). This afforded the titled probe **7** (15 mg, 55%) as a colourless oil. ν_{max} (ATR) 3310 (C-H alkyne), 2245 (C≡C), 1261 (P=O) cm^{-1}. δ_H NMR (599 MHz, $CDCl_3$) 4.30–4.20 (2H, m, -OC$\boldsymbol{H_2}$$CH_3$), 4.12 (2H, d, ^4J 2.1, 1'-$\boldsymbol{H_2}$), 3.51 (2H, t, ^3J 6.3, 5-$\boldsymbol{H_2}$), 2.41 (1H, d, ^4J 2.1, 3'-***H***), 1.92–1.85 (2H, m, 1-$\boldsymbol{H_2}$), 1.72–1.65 (2H, m), 1.65–1.58 (2H, m), 1.52–1.46 (2H, m), 1.37 (3H, t, ^3J 7.1, -$OCH_2C$$\boldsymbol{H_3}$). δ_C NMR (151 MHz, $CDCl_3$) 80.0 (O-***C***-2'), 74.3 (O-***C***-3'), 69.7 (O-***C***-5), 63.2 (d, $^2J_{C\text{-}P}$ 7.2, -O***C***H_2CH_3), 58.2 (O-***C***-1'), 29.0 (***C***-4), 27.0 (d, $^2J_{C\text{-}P}$ 17.4, ***C***-2), 24.4 (dd, $^1J_{C\text{-}P}$ 143.6, $^1J_{C\text{-}F}$ 22.6, ***C***-1), 21.9 (d, $^3J_{C\text{-}P}$ 5.4, ***C***-3), 16.5 (d, $^3J_{C\text{-}P}$ 5.6, -OCH_2***C***H_3). δ_P NMR (162 MHz, $CDCl_3$) 31.5 (d, $^1J_{P\text{-}F}$ 1069). δ_F NMR (376 MHz, $CDCl_3$) -64.6 (d, $^1J_{F\text{-}P}$ 1069). HRMS (ES^+) found $[M+H]^+$ 237.1048; $C_{10}H_{19}FO_3P$ requires M 237.1050.

Diethyl [(4-nitrophenyl)methyl]phosphonate (8)

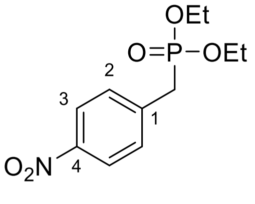

A solution of 4-nitrobenzyl bromide (2.0 g, 9 mmol, 1.0 eq) in $P(OEt_3)$ (32 mL, 0.18 mol, 20 eq) was heated for 1.75 h at 150 °C. The reaction mixture was then cooled to 20 °C and quenched with a solution of saturated $NaHCO_3$ solution (100 mL), and extracted with AcOEt (3 × 50 mL). The combined organic layers were washed with H_2O (2 × 50 mL) and brine (50 mL). The solvent was then removed *in vacuo*, and the residue was purified by flash chromatography (Hexane:EtOAc, 0-100% gradient) to afford the titled phosphonate **8** as a yellow oil (1.1 g, 44%). ν_{max} (ATR) 1522 (N-O stretching), 1352 (C-N stretching) cm^{-1}. δ_H (400 MHz, $CDCl_3$) 8.18 (1H, d, *J* 8.5, 3-***H***), 7.47 (1H, dd, *J* 8.5, $^3J_{H-P}$ 2.5, 2-***H***), 4.10 – 3.99 (4H, m, -OC***H₂***CH₃), 3.24 (2H, d, $^2J_{H-P}$ 22.4, Ar-C***H₂***P), 1.28 – 1.23 (6H, t, *J* 7.3, -OCH₂C***H₃***). δ_C (101 MHz, $CDCl_3$) 147.1 (d, $^5J_{C-P}$ 4.1, ***C***-4), 139.9 (d, $^4J_{C-P}$ 9.2, ***C***-1), 130.8 (d, J_{C-P} 6.4, ***C***-2), 123.8 (d, J_{C-P} 3.0, ***C***-3), 62.5 (d, $^2J_{C-P}$ 6.6, -O***C***H₂CH₃), 34.1 (d, $^1J_{C-P}$ 137.7, Ar-***C***H₂P), 16.5 (d, $^3J_{C-P}$ 6.0, -O**C**H₂***C***H₃). δ_P NMR (162 MHz, $CDCl_3$) 24.1. m/z LCMS (ES^+) 274.2 $[M+1]^+$, 316.4 $[M+1+MeCN]^+$. All the data are in accordance with the literature.[8]

Diethyl (4-aminophenyl)methylphosphonate (9)

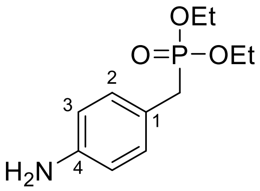

NH_4Cl (0.6 g, 0.01 mol, 3 eq) in water (0.2 M) and Zn (1.0 g, 16 mmol, 4 eq) were added to a solution of **8** (1.1 g, 4 mmol, 1 eq) in ethanol (20 mL) and the resulting mixture was stirred at reflux temperature for 2 h. Upon completion, the reaction mixture was filtered, and the filtrate was concentrated under vacuum, redissolved in water (20 mL), and extracted with EtOAc (3 × 50 mL). The organic layers were combined, dried, and concentrated under vacuum to afford the titled amine **9** (0.85 g, 86%) as a yellow oil. ν_{max} (ATR) 3349 (N-H stretching), 1628 (N-H bending) cm^{-1}. δ_H (400 MHz, $CDCl_3$) 7.13 – 7.08 (2H, m, 2-***H***), 6.68 – 6.64 (2H, m, 3-***H***), 4.07 – 3.95 (4H, *m*, -OC***H₂***CH₃), 3.64 (2H, s br, -N***H₂***), 3.05 (2H, d, $^2J_{H-P}$ 22.4, Ar-C***H₂***P), 1.28 – 1.18 (6H, t, *J* 7.2, -OCH₂C***H₃***). δ_C (101 MHz, $CDCl_3$) 145.2 (d, $^5J_{C-P}$ 4.1, ***C***-4), 130.8 (d, J_{C-P} 3.1, ***C***-2), 121.2 (d, J_{C-P} 6.4, ***C***-1), 115.5 (d, $^4J_{C-P}$ 3.0, ***C***-3), 62.3 (d, $^2J_{C-P}$ 6.6, -O***C***H₂CH₃), 32.8 (d, $^1J_{C-P}$ 137.7, Ar-***C***H₂P), 16.5 (d, $^3J_{C-P}$ 6.0, -O**C**H₂***C***H₃). δ_P NMR (162 MHz, $CDCl_3$) 27.3. m/z LCMS (ES^+) 244.3 $[M+H]^+$, 487. $[2M+H]^+$.

2,5-dioxopyrrolidin-1’-yl hex-5’-ynoate (10)

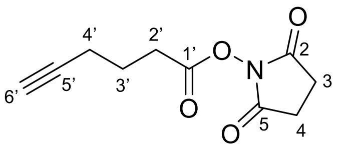

NHS (1.1 mg, 10 mmol, 1.1 eq) and EDC.HCl (1.9 mg, 10 mmol, 1.1 eq) were added to a solution of 5-hexynoic acid (1.0 mL, 9.2 mmol, 1.0 eq) in dry DCM (46 mL), and the reaction mixture was stirred at room temperature for 8 h. It was then washed with water (2 × 10 mL), brine (10 mL), dried over $MgSO_4$, filtered, and concentrated in vacuo. The residue was purified by flash chromatography (Hex:EtOAc gradient) to afford the titled NHS-ester **10** (956 mg, 50%) as a white solid. ν_{max} (ATR) 3288 (C≡C-H), 2260 (C≡C-H), 2262 (C≡C),1820 (C=O), 1786 (C=O), 1740 (C=O) cm^{-1}. δ_H (400 MHz, $CDCl_3$) 2.87 – 2.81 (4H, m, 2-***H***, 3-***H***), 2.77 (2H, t, *J* 7.1, 2'-***H***), 2.37 (2H, td, *J* 7.1, 2.7, 4'-***H***), 2.04 (1H, t, *J* 2.7, 6'-***H***), 2.00 (2H, p, *J* 7.1, 3'-***H***). δ_C (101 MHz, $CDCl_3$) 169.1 (***C***-2, ***C***-5), 168.2 (***C***-1'), 82.4 (***C***-5'), 69.8 (***C***-6'), 29.7 (***C***-2'), 25.6 (***C***-3, ***C***-4), 23.3 (***C***-3'), 17.6 (***C***-4'). m/z LCMS (ES^+) found 210.2 $[M+1]^+$. All data are in accordance with literature.[9]

Diethyl {[4-(hex-5-ynamido)phenyl]methyl}phosphonate (11)

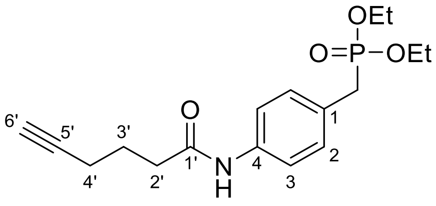

NHS-ester **10** (0.968 mL, 4.62 mmol, 1.25 eq) was dissolved in MeOH (37.0 mL) and stirred at room temperature for 24 h with **9** (0.900 mL, 3.70 mmol, 1 eq). Upon completion, the solvent was removed under reduced pressure, and the remaining crude was taken up in EtOAc (75 mL) and washed with brine (3 × 10 mL), dried and purified by flash chromatography (0-100% EtOAc:Hexane gradient) to afford the titled phosphonate **11** (1.6 g, 42 %) as a yellow solid. ν_{max} (ATR) 3302 (C≡C-H), 1673 (C=O), 1234 (P=O) cm^{-1}. δ_H (700 MHz, $CDCl_3$) 7.51 (1H, s br, -N***H***), 7.46 (2H, d, *J* 8.3, 3-***H***), 7.23 (2H, dd, *J* 8.3, $^3J_{H-P}$ 2.5, 2-***H***), 4.06 – 3.96 (4H, m, -OC$\boldsymbol{H_2}$$CH_3$), 3.11 (2H, d, $^2J_{H-P}$ 21.4, Ar-C$\boldsymbol{H_2}$P), 2.51 (2H, t, *J* 7.0, 2'-$\boldsymbol{H_2}$), 2.33 (2H, td, *J* 7.0, 2.6, 4'-$\boldsymbol{H_2}$), 2.00 (1H, t, *J* 2.6, 6'-***H***), 1.95 (2H, p, *J* 7.0 Hz, 3'-$\boldsymbol{H_2}$), 1.25 (6H, t, *J* 7.1, -OCH_2C$\boldsymbol{H_3}$). δ_C (176 MHz, $CDCl_3$) 170.6 (***C***-1'), 137,0 (***C***-4), 130.4 (d, $^3J_{C-P}$ 6.5, ***C***-2), 127.2 (d, $^2J_{C-P}$ 10.0, ***C***-1), 120.0 (d, $^4J_{C-P}$ 3.0 ***C***-3), 69.5 (***C***-6'), 62.3 (d, $^2J_{C-P}$ 7.0, -O***C***H_2CH_3), 36.1 (***C***-2'), 33.3 (d, $^1J_{C-P}$ 138.7, Ar-***C***H_2P), 24.1 (***C***-3'), 18.0 (***C***-4'), 16.55 (d, $^3J_{C-P}$ 5.8, -OCH_2***C***H_3). δ_P NMR (162 MHz, $CDCl_3$) 26.4. m/z LCMS (ES^+) 338.3 $[M+H]^+$, 675.5 $[2M+H]^+$.

Synthesis of ethyl {[4-(hex-5'-ynamido)phenyl]methyl}phosphonofluoridate (12)

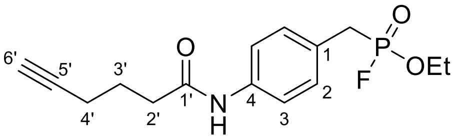

NaOH (11 mg, 0.3 mmol, 3 eq) was added to a solution of **11** (30 mg, 0.09 mmol, 1.0 eq) in 1:1 H_2O:EtOH (4.0 mL), and the mixture was allowed to react at 80 °C for 72 h. Subsequently, it was concentrated to remove the EtOH, diluted with H_2O (1 mL), acidified with 1N HCl (1 mL) and extracted with EtOAc (3 x 25 mL). The combined organic extracts were then washed with H_2O (1 × 10 mL) and concentrated. The resulting crude product was dissolved in dry DCM (4.0 mL), cooled to −78 °C and DAST (14 µL, 0.1 mmol, 1.1 eq) added dropwise. The mixture was stirred at −78 °C for 2 h, then quenched by addition to water at 0 °C. The volatiles were removed and the aqueous layer extracted with EtOAc (3 × 10 mL), concentrated, dried and purified by flash chromatography (Hex: EtOAc 0-100% gradient) to afford the benzylic fluorophosphonate **12** (10 mg, 37%) as a yellow oil. ν_{max} (ATR) 3311 (C≡C-H), 2250 (C≡C), 1677 (C=O) cm^{-1}. δ_H NMR (599 MHz, $CDCl_3$) 7.49 (2H, d, *J* 8.3, 3-***H₂***), 7.40 (1H, s, -N***H***), 7.24 (2H, dd, *J* 8.3, $^3J_{H-P}$ 2.7, 2-$\boldsymbol{H_2}$), 4.24 – 4.15 (2H, m, -OC$\boldsymbol{H_2}$$CH_3$), 3.32 – 3.21 (2H, m, Ar-C$\boldsymbol{H_2}$-P), 2.51 (2H, t, *J* 7.0, 2'-$\boldsymbol{H_2}$), 2.33 (2H, td, *J* 7.0, 2.7, 4'-$\boldsymbol{H_2}$), 2.01 (1H, t, *J* 2.7, 6'-$\boldsymbol{H_2}$), 1.95 (2H, p, *J* 7.0 Hz, 3'-$\boldsymbol{H_2}$), 1.30 (3H, t, *J* 7.1, -OCH_2C$\boldsymbol{H_3}$). δ_C NMR (151 MHz, $CDCl_3$) 170.5 (***C***-1'), 137.4 (d, $^5J_{C-P}$ 4.1, ***C***-4), 130.2 (d, $^3J_{C-P}$ 7.1, ***C***-2), 124.9 (d, $^2J_{C-P}$ 10.0, ***C***-1), 120.1 (d, $^4J_{C-P}$ 3.2, ***C***-3), 83.4 (***C***-5'), 69.4 (***C***-6'), 64.0 (d, $^2J_{C-P}$ 7.4, -O***C***H_2CH_3), 35.9 (***C***-2'), 31.6 (dd, $^1J_{C-P}$ 142.9, $^1J_{C-F}$ 24.5), 23.8 (***C***-3'), 17.8 (***C***-4'), 16.2 (d, $^3J_{C-P}$ 5.5, -OCH_2***C***H_3). δ_P NMR (162 MHz, $CDCl_3$) 25.0 (d, $^1J_{P-F}$ 1079). δ_F NMR (376 MHz, $CDCl_3$) -64.5 (d, $^1J_{P-F}$ 1079). HRMS (ES^+) found $[M+H]^+$ 12.1160; $C_{15}H_{20}NO_3FP$ requires *M* 312.1165.

Diethyl(4-hydroxyphenyl)phosphonate (13)

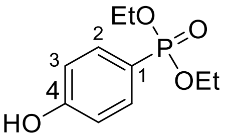

General procedure B was applied to *p*-bromophenol. Column eluent 0-5% MeOH in DCM. **13** (3572 mg, 90%) was obtained as a colourless oil. ν_{max} (ATR) 3122 (br, -OH), 1208 (P=O stretch), cm^{-1}. δ_H (400 MHz, $CDCl_3$) 10.00 (1H, s, -O***H***), 7.65 (2H, dd, $^3J_{H-P}$ 12.9, 8.6, 2-***H***), 7.03 (2H, dd, *J* 8.6, $^4J_{H-P}$ 3.7, 3-***H***), 4.22 – 3.97 (4H, m, -O-C$\boldsymbol{H_2}$-CH_3), 1.32 (6H , t, *J* 7.1, -CH_2-C$\boldsymbol{H_3}$). δ_C (101 MHz, $CDCl_3$) 161.9 (d, $^4J_{C-P}$ 3.4, ***C***-4), 133.8 (d, $^2J_{C-P}$ 11.7, ***C***-2), 116.1 (d, $^1J_{C-P}$ 197.0, ***C***-1), 116.0 (d, $^3J_{C-P}$ 16.4, ***C***-3), 62.3 (d, $^2J_{C-P}$ 5.4, -O-***C***H_2-CH_3), 16.2 (d, $^3J_{C-P}$ 6.6, -CH_2-***C***H_3). δ_P (162 MHz, $CDCl_3$) 21.1. *m/z* (LCMS ES^+) 231.1 $[M+H]^+$, 461.2 $[2M+H]^+$. All the data are in accordance with the literature.[10]

Hex-5-yn-1-yl 4-methylbenzene-1'-sulfonate (14)

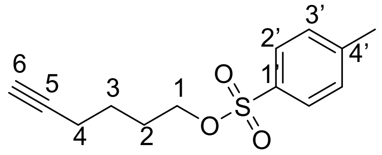

p-toluenesulfonyl chloride (3.27 g, 17.0 mmol, 1.7 eq) was added to a solution of 5-hexyn-1-ol (1.1 mL, 10 mmol, 1.0 eq) and pyridine (20 mL). The reaction mixture was stirred at 0 °C for 12 h, followed by an additional 12 h at room temperature under Ar. The resulting solution was washed with saturated $NaHCO_3$ solution (20 mL) and extracted with Et_2O (3 x 20 mL). Subsequently the organic layers were then washed with 2M HCl (3 x 20 mL) and water (20 mL). The combined organic layers were dried ($MgSO_4$) and concentrated. The residue was purified by flash chromatography (Hexane:EtOAc, 3:1) to afford the titled tosylate **14** (1.33 g, 53%) as a colourless oil. ν_{max} (ATR) 3298 (C-H stretching alkyne), 2123 (C≡C stretching), 1358 (S=O stretching) cm^{-1}. δ_H (599 MHz, $CDCl_3$) 7.77 (2H, d, *J* 8.1, 2'-***H***), 7.33 (2H, d, *J* 8.1, 3'-***H***), 4.05 (2H, t, *J* 6.3, 1-***H***$_2$), 2.43 (3H, s, 4'-C***H***$_3$), 2.14 (2H, td, *J* 6.9, 2.6, 4-***H***$_2$), 1.90 (1H, t, *J* 2.6, 6-***H***), 1.79 – 1.72 (2H, m, 2-***H***$_2$), 1.63 – 1.53 (2H, m, 3-***H***$_2$). δ_C (151 MHz, $CDCl_3$) 144.8 (***C***-4'), 133.1 (***C***-1'), 129.8 (***C***-3'), 127.9 (***C***-2'), 83.4 (***C***-5), 69.9 (***C***-1), 68.9 (***C***-6), 27.8 (***C***-2), 24.2 (***C***-3), 21.7 (-***C***H$_3$), 17.7 (***C***-4). *m/z* (LCMS ES$^+$) $[M+H]^+$ 253.3, $[2M+Na]^+$ 527.3. All the data are in accordance with the literature.[11]

Tert-butyl N-{6-[(4'-methylbenzenesulfonyl)oxy]hexyl}carbamate (15)

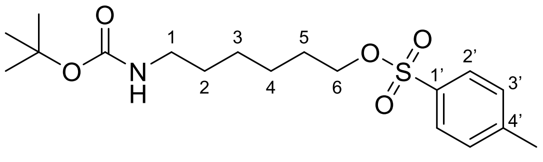

6-(Boc-amino)-1-hexanol **36** (1.0 g, 5 mmol, 1 eq) was dissolved in dry DCM (23 mL). Triethylamine (1.2 mL, 9 mmol, 2 eq), p-toluenesulfonyl chloride (1.5 g, 7.8 mmol, 1.7 eq), and a catalytic amount of DMAP (0.06 g, 0.5 mmol, 0.1 eq) were added. The solution was stirred at room temperature overnight. After this time, the volatiles were removed, and the residue was partitioned between ethyl acetate (150 ml) and 0.1 M HCl (150 ml). The organic layer was separated, and the aqueous phase was extracted with EtOAc (2 x 150 ml); the combined organic layers were washed with H_2O (2 x 150 ml), dried, filtered and concentrated under reduced pressure to afford the tosylated amino alcohol **15** (1.3 g, 78%) as a colourless oil. The product was used directly without further purification. δ_H NMR (400 MHz, $CDCl_3$) 7.78 (2H, d, *J* 8.0, Ar-***H***), 7.34 (2H, d, *J* 8.0, Ar-***H***), 4.01 (2H, t, *J* 6.4, 6-***H***$_2$), 3.05 (2H, t, *J* 7.1, 1-***H***$_2$), 2.45 (3H, s, Ph-C***H***$_3$), 1.63 (2H, m, 5-***H***$_2$), 1.43 (11H, s, -C***H***$_2$-, C(C***H***$_3$)$_3$), 1.28 (4H, m, 2 x -C***H***$_2$-). δ_C NMR (101 MHz, $CDCl_3$) 156.0 (C=O), 144.7 (***C***-1'), 133.1 (***C*** -4'), 129.8 (Ar- ***C***), 127.9 (Ar- ***C***), 79.3 (***C***(CH$_3$)$_3$), 70.5 (-O***C***H$_2$-), 40.4

(-N***C***H_2-), 29.9 (***C***H_2), 28.7 (***C***H_2), 28.4 (C(***C***H_3)$_3$), 26.1 (***C***H_2), 25.1 (***C***H_2), 21.7 (Ph-***C***H_3). m/z LCMS (ES^+) 316.3 $[M-101+46]^+$ (McLafferty rearrangement + H_2CO_2).

Tert-butyl N-{6-[4'-(diethoxyphosphoryl)phenoxy]hexyl}carbamate (16)

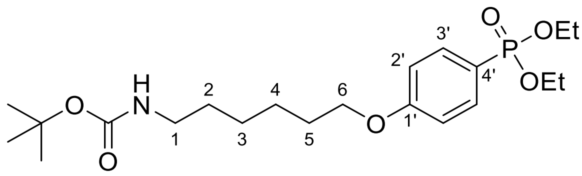

Phosphonate **13** and tosylated alcohol **15** were treated following the general procedure **C**. Column eluent 0-20% EtOH in DCM. **16** (400 mg, 72%) was obtained as a light-yellow oil. $\mathbf{v}_{max}$ (ATR) 1703 (C=O), 1245 (P=O) cm^{-1}. δ_H NMR (599 MHz, $CDCl_3$) 7.71 (2H, dd, $^3J_{H-P}$ 12.7, *J* 8.5, 3'-***H₂***), 6.93 (2H, dd, *J* 8.5, $^4J_{H-P}$ 3.3, 2'-$\boldsymbol{H_2}$), 4.51 (1H, s br, -N***H***), 4.11-4.03 (m, 4H, O-C$\boldsymbol{H_2}$-CH_3), 3.98 (2H, t, *J* 6.5, 6-$\boldsymbol{H_2}$), 3.11 (2H, t br, *J* 7.1, 1-$\boldsymbol{H_2}$), 1.78 (2H, p, *J* 6.5, 5-$\boldsymbol{H_2}$), 1.54 – 1.34 (13H, m, 4-$\boldsymbol{H_2}$, 3-$\boldsymbol{H_2}$, C(C$\boldsymbol{H_3}$)$_3$, 2-$\boldsymbol{H_2}$), 1.30 (6H, t, *J* 7.0, -CH_2-C$\boldsymbol{H_3}$). δ_C NMR (151 MHz, $CDCl_3$) 162.5 (d, $^4J_{C-P}$ 3.4, ***C***-1'), 156.1 (C=O), 133.9 (d, $^2J_{C-P}$ 11.3, ***C***-3'), 119.4 (d, $^1J_{C-P}$ 194.7, ***C***-4'), 114.6 (d, $^3J_{C-P}$ 16.0, ***C***-2'), 68.0 (***C***-6), 62.0 (d, $^2J_{C-P}$ 5.3, O-***C***H_2-CH_3), 60.5 (***C***(CH_3)$_3$), 40.6 (***C***-1), 30.2 (***C***-4), 29.1 (***C***-5), 28.5 (C(***C***H_3)$_3$), 26.6 (***C***-3), 25.8 (***C***-2), 16.4 (d, $^3J_{C-P}$ 6.5). δ_P NMR (162 MHz, $CDCl_3$) 19.82. HRMS (ES^+) found $[M+H]^+$ 430.2348, $C_{21}H_{37}NO_6P$ requires *M* 430.2059.

Diethyl [4-(prop-2'-yn-1'-yloxy)phenyl]phosphonate (17)

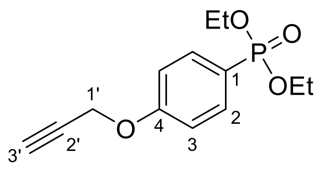

General procedure **C** was applied to diethyl(4-hydroxyphenyl)phosphonate (**13**). After the volatiles were removed, **17** (176 mg, 79%) was obtained as a brown oil. $\mathbf{v}_{max}$ (ATR) 3297 (C-H stretching alkyne), 2118 (C≡C stretching), 1228 (P=O stretching) cm^{-1}. δ_H (599 MHz, $CDCl_3$) 7.74 (2H, dd, $^3J_{H-P}$ 12.8, *J* 8.8, 2-***H***), 7.02 (2H, dd, *J 8.8*, $^4J_{H-P}$ 3.3, 3-***H***), 4.71 (2H, d, *J* 2.4, 1'-H_2), 4.14 – 3.99 (4H, m, O-C$\boldsymbol{H_2}$-CH_3), 2.53 (1H, t, *J* 2.4, 3'-***H***), 1.29 (6H, t, *J* 7.1, -C$\boldsymbol{H_3}$). δ_C (151 MHz, $CDCl_3$) 160.7 (d, $^4J_{C-P}$ 3.4, ***C***-4), 133.7 (d, $^2J_{C-P}$ 11.3, ***C***-2), 120.7 (d, $^1J_{C-P}$ 194.4, ***C***-1), 114.8 (d, $^3J_{C-P}$ 16.0, ***C***-3), 77.8 (***C***-2'), 76.1 (***C***-3'), 61.9 (d, $^2J_{C-P}$ 5.3, (CH_3-***C***H_2-O-), 55.7 (***C***-1'), 16.3 (d, $^3J_{C-P}$ 6.5, -***C***H_3). δ_P (162 MHz, $CDCl_3$), 19.2. m/z (LCMS ES^+) 269.2 $[M+H]^+$. HRMS (ES^+) found $[M+H]^+$ 269.0951; $C_{13}H_{18}O_4P$ requires *M* 269.0943.

Diethyl 4'-(hex-5-yn-1-yloxy)phenylphosphonate (18)

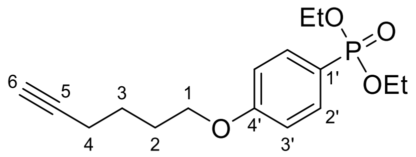

General procedure **C** was applied to diethyl-(4-hydroxyphenyl)phosphonate (**13**). Column eluent 0-100% EtOAc in hexanes. **18** (334 mg, 99%) was obtained as a colourless oil. ν_{max} (ATR) 3301 (C-H stretching alkyne), 2116 (C≡C stretching), 1603, 1242 (P=O stretching) cm^{-1}. δ_H (700 MHz, $CDCl_3$) 7.69 (2H, dd, $^3J_{H-P}$ 12.7, 8.7, 2'-***H***), 6.91 (2H, dd, *J* 8.7, $^4J_{H-P}$ 3.4, 3'-***H***), 4.11 – 3.96 (6H, m, -O-C$\boldsymbol{H_2}$-CH_3, 1-$\boldsymbol{H_2}$), 2.24 (2H, td, *J* 7.0, 2.7, 4-$\boldsymbol{H_2}$), 1.94 (1H, t, *J* 2.7, 6-***H***), 1.92 – 1.87 (2H, m, 2-$\boldsymbol{H_2}$), 1.71 – 1.65 (2H, m, 3-$\boldsymbol{H_2}$), 1.27 (6H, t, *J* 7.1, -O-CH_2-C$\boldsymbol{H_3}$). δ_C (176 MHz, $CDCl_3$) 162.2 (d, $^4J_{C-P}$ 3.4, ***C***-4'), 133.7 (d, $^3J_{C-P}$ 11.3, ***C***-3'), 119.4 (d, $^1J_{C-P}$ 194.8, ***C***-1'), 114.4 (d, $^2J_{C-P}$ 15.9, ***C***-2'), 83.8 (***C***-5), 68.7 (***C***-6), 67.3 (***C***-1), 61.8 (d, $^2J_{C-P}$ 5.3, -O-***C***H_2-CH_3), 28.0 (***C***-2), 24.9 (***C***-3), 18.1 (***C***-4), 16.3 (d, $^3J_{C-P}$ 6.5, -CH_2-***C***H_3). δ_P (162 MHz, $CDCl_3$) 19.8. m/z (LCMS ES^+) 311.3 $[M+H]^+$, 621.5 $[2M+H]^+$. HRMS (ES^+) found 311.1405 $[M+H]^+$; $C_{16}H_{24}O_4P$ requires *M* 311.1412.

Diethyl (4-{[6'-(hex-5''-ynamido)hexyl]oxy}phenyl)phosphonate (19)

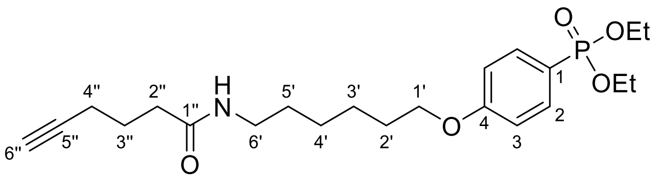

Boc protection was removed from **16** following general procedure **F** (m/z LCMS (ES^+) 330.4$[M+H]^+$). Subsequent amide coupling with 5-hexynoic acid following general procedure **G** afforded **19** (155 mg, 31%) as a yellow oil. Column eluent 0-30% EtOH in DCM. $\boldsymbol{\nu}_{max}$ (ATR) 3312 (C≡C-H), 2247 (C≡C), 1653 (C=O), 1237 (P=O) cm^{-1}. δ_H NMR (599 MHz, $CDCl_3$) 7.72 (2H, dd, $^3J_{H-P}$ 12.7, *J* 8.7, 2-***H***), 6.94 (2H, dd, *J* 8.7, $^4J_{H-P}$ 3.4, 3-***H***), 5.60 (1H, s, -N***H***-), 4.15 – 4.00 (4H, m, -O-C$\boldsymbol{H_2}$-CH_3), 3.99 (2H, t, *J* 6.4, 1'-$\boldsymbol{H_2}$), 3.26 (2H, td, *J* 7.2, 5.7, 6'-$\boldsymbol{H_2}$), 2.31 (2H, t, *J* 7.1, 2''-$\boldsymbol{H_2}$), 2.25 (2H, td, *J* 7.1, 2.7, 4''-$\boldsymbol{H_2}$), 1.97 (1H, t, *J* 2.7, ***6''-H***), 1.86 (2H, p, *J* 7.1, 3''-$\boldsymbol{H_2}$), 1.82 – 1.76 (2H, m, 2'-$\boldsymbol{H_2}$), 1.57 – 1.45 (4H, m, 5'-$\boldsymbol{H_2}$, 3'-$\boldsymbol{H_2}$), 1.43 – 1.36 (2H, m, 4'-$\boldsymbol{H_2}$), 1.31 (t, *J* 7.0, -O-CH_2-***C***H_3). δ_C NMR (151 MHz, $CDCl_3$) 172.3 (C=O), 162.5 (d, $^4J_{C-P}$ 3.4, ***C***-4), 133.9 (d, $^2J_{C-P}$ 11.3, ***C***-2), 119.4 (d, $^1J_{C-P}$ 194.8, ***C***-1), 114.6 (d, $^3J_{C-P}$ 16.0, ***C***-3), 83.7 (***C***-5''), 69.3 (***C***-6''), 68.0 (***C***-1'), 62.0 (d, $^2J_{C-P}$ 5.3, -O-***C***H_2-CH_3), 39.6 (***C***-6'), 35.2 (***C***-2''), 29.7 (***C***-5'), 29.1 (***C***-2'), 26.7 (***C***-4'), 25.8 (***C***-3'), 24.3 (***C***-3''), 17.9 (***C***-4''), 16.5 (d, $^3J_{C-P}$ 6.6, -O-CH_2-***C***H_3). δ_P NMR (162 MHz, $CDCl_3$) 19.8. HRMS (ES^+) found $[M+H]^+$ 424.2256; $C_{22}H_{35}NO_5P$ requires *M* 424.2253.

Ethyl [4-(prop-2'-yn-1'-yloxy)phenyl]phosphonofluoridate (20)

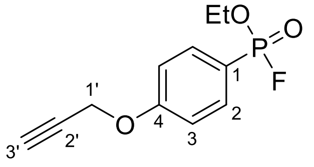

Phosphonate **17** was treated following the general procedure **D** using H_2O as solvent for the hydrolysis step and stirred during 13 h to afford the intermediate phosphinic acid. m/z LCMS (ES^+) 241.1 $[M+H]^+$, 481.1 $[2M+H]^+$. Subsequent fluorination afforded the title fluorophosphonate **20** (16 mg, 36%) as a brown oil. Column eluent 0-100% EtOAc in hexanes. ν_{max} (ATR) 3305 (H-C≡C), 2125 (C≡C), 1140 (P=O), 1036 (C-O) cm^{-1}. δ_H (599 MHz, $CDCl_3$) 7.80 (2H, dd, $^3J_{H-P}$ 13.8, 8.8, 2-***H***), 7.12 – 7.03 (2H, m, 3-***H***), 4.76 (2H, d, *J* 2.4, 1'-$\boldsymbol{H_2}$), 4.36 – 4.26 (2H, m, O-C$\boldsymbol{H_2}$-CH_3), 2.55 (1H, t, *J* 2.4, 3'-***H***), 1.41 (3H, t, *J* 7.1, -C$\boldsymbol{H_3}$). δ_C (151 MHz, $CDCl_3$) 161.6 (d, $^4J_{C-P}$ 3.8, ***C***-4), 133.8 (d, $^2J_{C-P}$ 12.5, ***C***-2), 116.8 (dd, $^1J_{C-P}$ 209.4, $^2J_{C-F}$ 30.9, ***C***-1), 115.1 (d, $^3J_{C-P}$ 17.5, ***C***-3), 77.5 (***C***-2'), 76.4 (***C***-3'), 63.6 (d, $^2J_{C-P}$ 6.1, -O-***C***H_2-CH_3), 55.8 (***C***-1'), 16.3 (d, $^3J_{C-P}$ 6.0, -***C***H_3). δ_P (162 MHz, $CDCl_3$), 17.7 (d, $^1J_{P-F}$1035.1). δ_F (376 MHz, $CDCl_3$) -63.5 (d, $^1J_{F-P}$1035). HRMS (ES+) found $[M+H]^+$ 243.0575; $C_{11}H_{13}O_3FP$ requires *M* 243.0586.

Ethyl [4-(hex-5´-yn-1´-yloxy)phenyl]phosphonofluoridate (21)

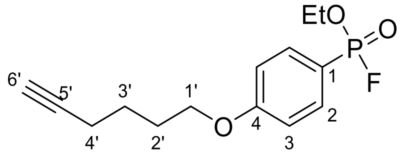

Phosphonate **18** was treated following the general procedure **D** using H_2O as solvent for the hydrolysis step and stirred for 6 h to afford the intermediate phosphinic acid. After the fluorination, **21** (62 mg, 68%) was obtained as a brown oil. Column eluent 0-100% EtOAc in hexanes. ν_{max} (ATR) 3309 (C≡C-H), 2247 (C≡C) cm^{-1}. δ_H (599 MHz, $CDCl_3$) 7.79 – 7.70 (2H, m, 2-***H***), 6.96 (2H, dd, *J* 8.3, $^4J_{H-P}$ 3.9, 3-***H***), 4.34 – 4.22 (2H, m, -O-C$\boldsymbol{H_2}$-CH_3), 4.03 (2H, t, *J* 6.3, *1´*-***H***), 2.27 (2H, td, *J* 7.0, 2.7, 4'-$\boldsymbol{H_2}$), 1.96 (1H t, *J* 2.7, 6'-$\boldsymbol{H_2}$), 1.95 – 1.90 (2H, m, 2'-$\boldsymbol{H_2}$), 1.74 – 1.68 (2H, m, 3'-$\boldsymbol{H_2}$), 1.39 (3H, t, *J* 7.1, -O-CH_2-C$\boldsymbol{H_3}$). δ_C (151 MHz, $CDCl_3$) 163.2 (d, $^4J_{C-P}$ 3.6, ***C***-4), 133.8 (d, $^2J_{C-P}$ 12.9, ***C***-2), 116.3 (dd, $^1J_{C-F}$ 209.9, $^1J_{C-P}$ 30.5, ***C***-1), 114.7 (d, $^3J_{C-P}$ 17.5, ***C***-3), 83.8 (***C***-5'), 68.8 (***C***-6'), 67.5 (***C***-1'), 63.5 (d, $^2J_{C-P}$ 6.1, -O-***C***H_2-CH_3), 28.0 (***C***-2'), 24.9 (***C***-3'), 18.1 (***C***-4'), 16.3 (d, $^2J_{C-P}$ 6.1, -O-***C***H_2-CH_3). δ_P (162 MHz, $CDCl_3$) 18.3 (d, $^1J_{P-F}$ 1033.5). δ_F (376 MHz, $CDCl_3$) -63.7 (d, $^1J_{P-F}$ 1033.5). HRMS (ES^+) found $[M+H]^+$ 285.1068, $C_{14}H_{18}FO_3P$ requires *M* 285.1056.

Ethyl (4-{[6-(hex-5-ynamido)hexyl]oxy}phenyl)phosphonofluoridate (22)

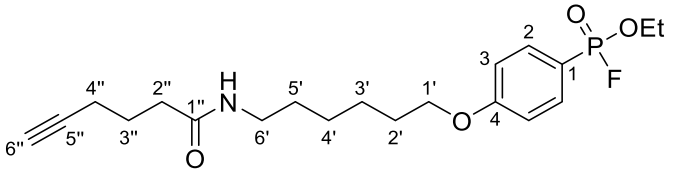

79

Phosphonate **19** was treated following the general procedure **D** using EtOH:H_2O (1:1) as solvent system for the hydrolysis step and stirred for 46 h. After the fluorination, **22** (42 mg, 60%) was obtained as a yellow oil. Column eluent 0-10% MeOH in DCM. ν_{max} (ATR) 3315 (C≡C-H), 1645 (C=O), 1263 (P=O) cm^{-1}. δ_H NMR (599 MHz, $CDCl_3$) 7.76 (2H, dd, $^3J_{H-P}$ 13.8, J 8.8, 2-***H***), 6.77 (2H, dd, J 8.8, $^4J_{H-P}$ 3.7, 3-***H***), 5.53 (1H, s br., -N***H***-), 4.36 – 4.25 (2H, m, -O-C$\boldsymbol{H_2}$-CH_3), 4.00 (2H, t, J 6.4, 1'-$\boldsymbol{H_2}$), 3.26 (2H, td, J 7.2, 5.8, 6'-$\boldsymbol{H_2}$), 2.31 (2H, t, J 7.1, 2''-$\boldsymbol{H_2}$), 2.25 (2H, td, J 7.1, 2.6, 4''-$\boldsymbol{H_2}$), 1.97 (1H, t, J 2.6, 6''-***H***), 1.86 (2H, p, J 7.1, 3''-$\boldsymbol{H_2}$), 1.83 – 1.76 (2H, m, 2'-$\boldsymbol{H_2}$), 1.58 – 1.45 (4H, m, 5'-$\boldsymbol{H_2}$, 3'-$\boldsymbol{H_2}$), 1.43 – 1.36 (6H, m, 4'-$\boldsymbol{H_2}$, -O-CH_2-***C***H_3). δ_C NMR (151 MHz, $CDCl_3$) 172.3 (C=O), 163.5 (d, $^4J_{C-P}$ 3.6, ***C***-4), 134.0 (d, $^2J_{C-P}$ 12.5, ***C***-2), 115.6 (d, $^1J_{C-P}$ 179.1, ***C***-1), 114.9 (d, $^3J_{C-P}$ 17.4, ***C***-3), 83.7 (***C***-5''), 69.3 (***C***-6''), 68.2 (***C***-1'), 63.7 (d, $^2J_{C-P}$ 6.0, -O-***C***H_2-CH_3), 39.6 (***C***-6'), 35.2 (***C***-2''), 29.8 (***C***-5'), 29.1 (***C***-2'), 26.7 (***C***-4'), 25.8 (***C***-3'), 24.3 (***C***-3''), 17.9 (***C***-4''), 16.5 (d, $^3J_{C-P}$ 6.1, -O-CH_2-***C***H_3). δ_P (162 MHz, $CDCl_3$) 18.3 (d, $^1J_{P-F}$ 1033). δ_F (376 MHz, $CDCl_3$) -63.7 (d, $^1J_{P-F}$ 1033). HRMS (ES^+) found $[M+H]^+$ 398.1880; $C_{20}H_{30}FNO_4P$ requires *M* 398.1897.

Diethyl 4-hydroxy-[1,1'-biphenyl]-4'-ylphosphonate (23)

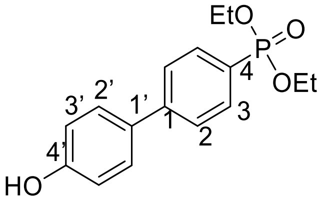

General procedure **B** was applied to 4-bromo-4'-hydroxybiphenyl. **24** (54 mg, 31%) was obtained as a yellow oil. Column eluent 0-100% EtOAc in Hexanes. ν_{max} (ATR) 3179 (br, -OH stretching), 1218 (P=O stretching) cm^{-1}. δ_H (700 MHz, $CDCl_3$) 8.13 (1H, s, -O***H***), 7.80 (2H, dd, $^3J_{H-P}$ 13.2, J 7.9, 3-***H***), 7.62-7.52 (2H, m, 2-***H***), 7.48-7.36 (2H, m, 2'-***H***), 6.97 (2H, d, J 8.4, 2'-***H***), 4.22 – 4.08 (4H, m, -O-C$\boldsymbol{H_2}$-CH_3), 1.33 (6H, t, J 7.1, -CH_2-C$\boldsymbol{H_3}$). δ_C (176 MHz, $CDCl_3$) 157.4 (***C***-4'), 145.4 (***C***-1'), 132.2 (d, $^2J_{C-P}$ 10.5, ***C***-3), 131.1 (***C***-1), 128.3 (***C***-2'), 126.6 (d, $^3J_{C-P}$ 15.6, ***C***-2), 124.8 (d, $^1J_{C-P}$ 205.0, ***C***-4), 116.13 (***C***-3'), 62.55 (m, -O-***C***H_2-CH_3), 16.25 (d, $^3J_{C-P}$ 6.6, O-CH_2-***C***H_3). δ_P (162 MHz, $CDCl_3$) 19.4. m/z (LCMS ES^+) 307.3 $[M+H]^+$, 613.4 $[2M+H]^+$. HRMS (ES^+) found $[M+H]^+$ 307.1096; $C_{16}H_{20}O_4P$ requires *M* 307.1099. All the data are in accordance with the literature.[12]

Diethyl [4'-(hex-5”-yn-1”-yloxy)-[1,1'-biphenyl]-4-yl]phosphonate (24)

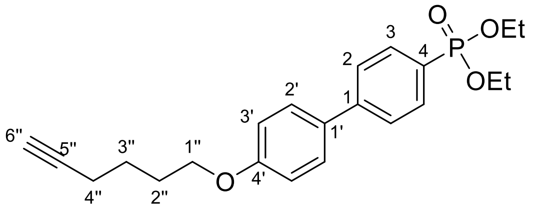

General procedure **C** was applied on diethyl 4'-hydroxy-[1,1'-biphenyl]-4-ylphosphonate (**23**). Column eluent 0-100% EtOAc in hexanes. **24** (182 mg, 85%) was obtained as a colourless oil. $\mathbf{v}_{max}$ (ATR) 3301 (H-C≡C stretching), 2121 (C≡C stretching), 1249 (P=O stretching) cm^{-1}. δ_H (700 MHz, $CDCl_3$) 7.83 (2H, dd, $^3J_{H-P}$ 13.0, 8.3, 3-***H***), 7.63 (2H, dd, *J* 8.3, $^4J_{H-P}$ 3.8, 2-***H***), 7.54 – 7.51 (2H, m, 2’-***H***), 6.98 – 6.95 (2H, m, 3’-***H***), 4.18 – 4.05 (4H, m, -O-C$\boldsymbol{H_2}$-CH_3), 4.02 (2H, t, *J* 6.3, 1´´-$\boldsymbol{H_2}$), 2.28 (2H, td, *J* 7.1, 2.6, 4´´-$\boldsymbol{H_2}$), 1.96 (1H, t, *J* 2.6, 6´´-***H***), 1.95 – 1.90 (2H, m, 2´´-$\boldsymbol{H_2}$), 1.76 – 1.70 (2H, m, 3´´-$\boldsymbol{H_2}$), 1.32 (6H, t, *J* 7.1, -O-CH_2-C$\boldsymbol{H_3}$). δ_C (176 MHz, $CDCl_3$) 159.2 (***C***-4’), 144.7 (d, $^4J_{C-P}$ 3.1, ***C***-1), 132.3 (d, $^2J_{C-P}$ 10.2, ***C***-3), 132.2 (d, $^5J_{C-P}$ 9.9, ***C***-1’), 128.3 (***C***-2’), 126.6 (d, $^3J_{C-P}$ 15.3, ***C***-2), 126.0 (d, $^1J_{C-P}$ 190.9, ***C***-4), 114.9 (***C***-3’), 84.0 (***C***-5”), 68.7 (***C***-6”), 67.4 (***C***-1”), 62.0 (d, $^3J_{C-P}$ 5.2, -O-***C***H_2-CH_3), 28.2 (***C***-2”), 25.0 (***C***-3”), 18.1 (***C***-4”),16.3 (d, $^3J_{C-P}$ 6.6, -CH_2-***C***H_3). δ_P (162 MHz, $CDCl_3$) 19.2. m/z (LCMS ES^+) 387.4 $[M+H]^+$, 774,6 $[2M+H]^+$. HRMS (ES^+) found $[M+H]^+$ 387.1734; $C_{22}H_{28}O_4P$ requires *M* 387.1725.

Ethyl [4'-(hex-5”-yn-1”-yloxy)-[1,1'-biphenyl]-4-yl]phosphonofluoridate (25)

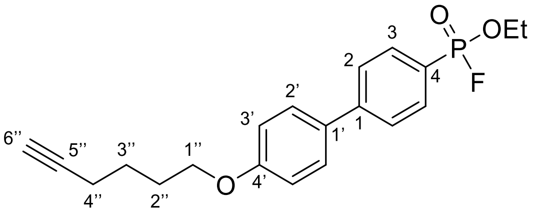

Phosphonate **24** was treated following the general procedure **D** using EtOH:H_2O (1:1) as solvent system for the hydrolysis step and stirred for 26 h. After the fluorination, **25** (11 mg, 54%) was obtained as a white oil. Column eluent 0-30% MeOH in DCM. $\mathbf{v}_{max}$ (ATR) 3311 (C≡C-H stretch), 1041 (C-O) cm^{-1}. δ_H (599 MHz, $CDCl_3$) 7.88 (2H, dd, $^3J_{H-P}$ 14.1, *J* 8.4, 3-***H***), 7.71-7.65 (2H, m, 2-***H***), 7.57 – 7.52 (2H, m, 2’-***H***), 7.01 – 6.97 (2H, m, 3’-***H***), 4.42 – 4.27 (2H, m, -O-C$\boldsymbol{H_2}$-CH_3), 4.05 (2H, t, *J* 6.3, 1´´-$\boldsymbol{H_2}$), 2.30 (2H, td, *J* 7.2, 2.6, 4´´-$\boldsymbol{H_2}$), 1.96 (1H, t, *J* 2.6, 6´´-***H***), 1.97 – 1.92 (2H, m, 2´´-$\boldsymbol{H_2}$), 1.78 – 1.72 (2H, p, *J* 7.2, 3´´-$\boldsymbol{H_2}$), 1.44 (3H, t, *J* 7.1, -O-CH_2-C$\boldsymbol{H_3}$). δ_C (151 MHz, $CDCl_3$) 159.7 (***C***-4’), 146.2 (d, $^4J_{C-P}$ 3.4, ***C***-1), 132.4 (d, $^2J_{C-P}$ 11.3, ***C***-3), 132.0

(***C***-1'), 128.6 (***C***-2'), 127.0 (d, $^3J_{C-P}$ 16.7, ***C***-2), 122.4 (dd, $^1J_{C-P}$ 204.1 $^2J_{C-F}$ 30.3, ***C***-4), 115.2 (***C***-3'), 84.2 (***C***-5''), 68.8 (***C***-6''), 67.6 (***C***-1''), 64.0 (d, $^3J_{C-P}$ 6.2, -O-***C***H2-CH3), 28.4 (***C***-2''), 25.2 (***C***-3''), 18.3 (***C***-4''),16.5 (d, $^3J_{C-P}$ 6.0, -CH2-***C***H3). δ_P (162 MHz, $CDCl_3$) 17.6 (d, $^1J_{P-F}$ 1039.5). δ_F (376 MHz, CDCl3) -63.8 (d, $^1J_{F-P}$ 1039.5). HRMS (ES^+) found $[M+H]^+$ 361.1349; $C_{20}H_{23}FO_3P$ requires *M* 361.1369.

5-(Boc-amino)-1-pentanol (26)

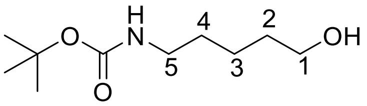

5-amino-1-pentanol (2.0 g, 18 mmol, 1.0 eq) was dissolved in dry DCM (45 mL) in a round-bottom flask equipped with a magnetic stirrer and a pressure equalising addition funnel. A solution of di-tert-butyl dicarbonate (4.0 g, 18 mmol, 1.0 eq) in dry DCM (45 mL) was added dropwise over 30 min. After stirring for 12 h under N_2, the reaction mixture was washed with saturated $NaHCO_3$ and brine, dried ($MgSO_4$), and concentrated to afford the protected amino alcohol **26** (3.8 g, 99%) as a light-yellow oil. **v**$_{max}$ (ATR) 3345 (-OH), 1685 (C=O), 1166 (C-O) cm^{-1}. δ_H (700 MHz, $CDCl_3$) 4.58 (1H, s, -N***H***-), 3.64 – 3.55 (2H, m,1-***H₂***), 3.13-3.07 (2H, m, 5-$\boldsymbol{H_2}$), 1.59 – 1.53 (2H, m, 2-$\boldsymbol{H_2}$), 1.51 – 1.45 (2H, m, 4-$\boldsymbol{H_2}$), 1.41 (9H, s, -C$\boldsymbol{H_3}$), 1.39-1.34 (2H, 3-$\boldsymbol{H_2}$). δ_C (176 MHz, $CDCl_3$) 156.0 (***C***=O), 79.1 (-***C***-$(CH_3)_3$), 62.6 (***C***-1), 40.4 (***C***-5), 32.2 (***C***-2), 29.8 (***C***-4), 28.4 (-***C***H3), 22.9 (***C***-3). m/z LCMS (ES^-) 248.2 $[M-H+46]^-$.

Tert-butyl N-{5-[(4'-bromophenyl)methoxy]pentyl}carbamate (27)

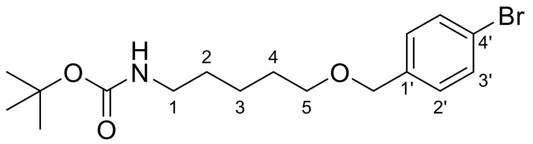

To a stirred solution of NaH (122 mg, 3.0 mmol, 1.3 eq) in dry THF (12 mL) under N_2, 5-(Boc-amino)-1-pentanol (**26**) (523 µL, 2.47 mmol, 1.05 eq) was added. After stirring at room temperature for 30 min, 4-bromobenzyl bromide (600 mg, 2.35 mmol, 1.0 eq), and TBAI (87 mg, 0.23 mmol, 0.1 eq) were added and the reaction was left until no starting material was observed by TLC. It was then concentrated, redissolved in EtOAc (20 mL), washed with water (2 x 10 mL), dried ($MgSO_4$) and purified by flash chromatography (Hex:EtOAc 0-100% gradient) to afford the titled carbamate **27** (600 mg, 69%) as a colourless oil. **v**$_{max}$ (ATR) 1695 (C=O), 1170 (C-O) cm^{-1}. δ_H (700 MHz, $CDCl_3$) 7.45 (2H, d, *J* 8.3, 3'-***H***), 7.19 (2H, d, *J* 8.3, 2'-***H***), 4.50 (1H, s, -N***H***-), 4.42 (2H, s, -O-C$\boldsymbol{H_2}$-Ar), 3.44 (2H, t, *J* 6.5, 5-$\boldsymbol{H_2}$), 3.10 (2H, d, *J* 9.0, 1-$\boldsymbol{H_2}$), 1.63 – 1.58 (2H, m, 4-$\boldsymbol{H_2}$), 1.48 (2H, p, *J* 7.0, 2-$\boldsymbol{H_2}$), 1.42 (9H, s, -C$\boldsymbol{H_3}$), 1.41 – 1.35 (2H, m, 3-$\boldsymbol{H_2}$). δ_C (176 MHz, $CDCl_3$) 155.9 (C=O), 137.6 (***C***-1'), 131.4 (***C***-3'), 129.2 (***C***-2'), 121.3 (***C***-4'), 79.0 -***C***$(CH_3)_3$, 72.1 (-O-

CH_2-Ar), 70.3 (**C**-5), 40.5 (**C**-1), 29.9 (**C**-2), 29.3 (**C**-4), 28.4 (-**C**H_3), 23.5 (**C**-3). m/z (ES^-) [M (^{79}Br)-H+46]$^-$ 416.1; [(^{81}Br)-H+46]$^-$ 418.1. HRMS (ES^+) found $[M+H]^+$ 372.1181; $C_{17}H_{27}NO_3{}^{79}Br$ requires *M* 372.1174.

Tert-butyl N-(5-{[4'-(diethoxyphosphoryl)phenyl]methoxy}pentyl)carbamate (28)

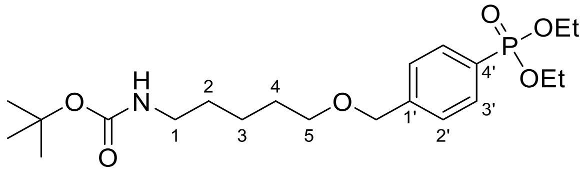

General procedure **B** was applied to **27**. Column eluent 0-100% H_2O in MeCN, using a C18 reverse phase column. **28** (992 mg, 61%) was obtained as a colourless oil. **v**$_{max}$ (ATR) 3318 (N-H), 1708 (C=O), 1243 (P=O), 1019 (C-O) cm^{-1}. δ_H (599 MHz, $CDCl_3$) 7.77 (2H, dd, $^3J_{H-P}$ 13.1, *J* 7.8, 3'-***H***), 7.41 (2H, dd, *J* 7.8, $^4J_{H-P}$ 3.9, 2'-***H***), 4.55 (1H, s, -N***H***-), 4.51 (2H, s, -O-C***H₂***-Ar), 4.16 – 3.99 (4H, m, -O-C***H₂***-CH_3), 3.47 (2H, t, *J* 6.6, 5-***H₂***), 3.13-3.07 (2H, m, 1-***H₂***), 1.62 (2H, dt, *J* 14.4, 6.6, 4-***H₂***), 1.48 (2H, p, *J* 7.2, 2-***H₂***), 1.44 – 1.35 (11H, m, -C(C***H₃***)₃, 3-***H₂***), 1.30 (6H, t, *J* 7.1, -O-CH_2-C***H₃***). δ_C (151 MHz, $CDCl_3$) 155.9 (C=O), 143.4 (d, $^4J_{C-P}$ 3.1, **C**-1'), 131.9 (d, $^2J_{C-P}$ 10.3, **C**-3'), 127.3 (d, $^1J_{C-P}$ 188.5, **C**-4'), 127.2 (d, $^3J_{C-P}$ 15.2, **C**-2'), 79.0 (-**C**(CH_3)₃), 72.2 (-O-**C**H_2-Ar), 70.6 (**C**-5), 62.0 (d, $^2J_{C-P}$ 5.3, -O-**C**H_2-CH_3), 40.5 (**C**-1), 29.9 (**C**-2), 29.3 (**C**-4), 28.4 (-C(**C**H_3)₃), 23.4 (**C**-3), 16.3 (d, $^3J_{C-P}$ 6.5, -O-CH_2-**C**H_3). δ_P (162 MHz, $CDCl_3$) 18.9. HRMS (ES^+) found $[M+H]^+$ 430.2368; $C_{21}H_{37}NO_6P$ requires *M* 430.2359.

Diethyl [4-({[5´-(hex-5´´-ynamido)pentyl]oxy}methyl) phenyl] phosphonate (29)

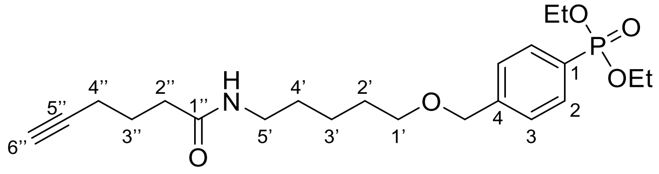

Compound **28** was deprotected following general procedure **F**. Amine intermediate was obtained as a yellow oil and used directly without further purification. After the volatiles were removed, general procedure **G** was applied to the free amine. After workup, **29** (402 mg, 65%) was obtained as a white solid. The product was used directly without further purification. **v**$_{max}$ (ATR) 3468 (N-H), 3299 (C≡C-H), 1646 (C=O), 1236 (P=O), 1022 (C-O) cm^{-1}. δ_H (599 MHz, $CDCl_3$) 7.71 (2H, dd, $^3J_{H-P}$ 13.1, 8.1, 2-***H***), 7.42 (2H, dd, *J* 8.1, $^4J_{H-P}$ 3.9, 3-***H***), 5.56 (1H, m, -N***H***-), 4.52 (2H, s, -O-C***H₂***-Ar), 4.17 – 4.00 (4H, m, -O-C***H₂***-CH_3), 3.48 (2H, t, *J* 6.4, 1'-***H₂***), 3.24 (2H, td, *J* 7.2, 5.8, 5'-***H₂***), 2.28 (2H, t, *J* 7.1, 2''-***H₂***), 2.24 (2H, td, *J* 7.1, 2.6, 4''-***H₂***), 1.96 (1H, t, *J* 2.6, 6''-***H***), 1.84 (2H, p, *J* 7.1, 3''-***H₂***), 1.64 (2H, dt, *J* 14.9, 6.4, 2'-***H₂***), 1.52 (2H, p, *J* 7.2, 4'-***H₂***), 1.44 – 1.38 (2H, m, 3'-***H₂***), 1.31 (2H, t, *J* 7.0, -CH_2-C***H₃***). δ_C (599 MHz, $CDCl_3$) 172,1 (C=O), 143.3 (d, $^4J_{C-P}$ 3.1, **C**-4), 131.9 (d, $^2J_{C-P}$ 10.2, **C**-2), 127.4 (d, $^1J_{C-P}$ 189.2, **C**-1), 127.2 (d, $^3J_{C-P}$ 15.3, **C**-3), 83.5 (**C**-5''), 72.3 (-O-**C**H_2-Ar), 70.6 (**C**-1'), 69.1 (**C**-6''), 62.0 (d, $^2J_{C-P}$ 5.4, -O-**C**H_2-CH_3), 39.4 (**C**-5'), 35.1 (**C**-2''),

29.5 (**C**-4'), 29.3 (**C**-2'), 24.2 (**C**-3''), 23.6 (**C**-3'), 17.8 (**C**-4''), 16.3 (d, $^{3}J_{C-P}$ 6.4, -O-CH$_2$-**C**H$_3$). δ_P (162 MHz, CDCl$_3$) 18.8. HRMS (ES$^+$) found [M+H]$^+$ 424.2256; $C_{22}H_{34}NO_4P$ requires *M* 424.2253.

Ethyl [4-({[5´-(hex-5´´-ynamido)pentyl]oxy}methyl)phenyl] phosphonofluoridate (30)

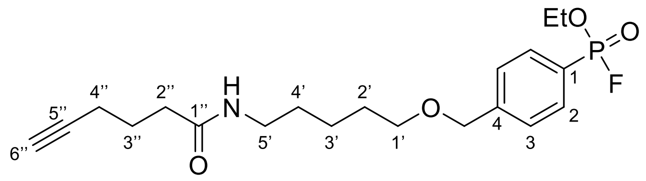

Phosphonate **29** was treated following the general procedure **D** using H_2O as solvent for the hydrolysis step and stirred for 6.5 h. After fluorination, **30** (51 mg, 64%) was obtained as a brown oil. Column eluent 0-100% EtOAc in hexanes. **ν**$_{max}$ (ATR) 3513 (N-H), 3310 (C≡C-H), 1652 (C=O), 1279 (P=O) 1039 (C-O) cm^{-1}. δ_H (700 MHz, CDCl$_3$) 7.81 (2H, dd, $^{3}J_{H-P}$ 14.1, 8.0, 2-***H***), 7.46 (2H, dd, *J* 8.0, $^{4}J_{H-P}$ 4.6, 3-***H***), 5.52 (1H, s, -N***H***-), 4.55 (2H, s, -O-C***H₂***-Ar), 4.37 – 4.27 (2H, m, -O-C***H₂***-CH$_3$), 3.49 (2H, t, *J* 6.5, 1'-***H₂***), 3.25 (2H, dt, *J* 7.2, 6.7, 5'-***H₂***), 2.29 (2H, t, *J* 7.2, 2''-***H₂***), 2.24 (2H, td, *J* 7.2, 2.7, 4''-***H₂***), 1.96 (1H, t, *J* 2.7, 6''-***H***), 1.84 (2H, p, *J* 7.2, 3''-***H₂***), 1.64 (2H, dt, *J* 14.8, 6.5, 2'-***H₂***), 1.52 (2H, p, *J* 7.3, 4'-***H₂***), 1.41 (5H, m, 3'-***H₂***, -O-CH$_2$-C***H₃***). δ_C (176 MHz, CDCl$_3$) 172,1 (C=O), 144.9 (d, $^{4}J_{C-P}$ 3.4, **C**-4), 131.8 (d, $^{2}J_{C-P}$ 11.3, **C**-2), 127.3 (d, $^{3}J_{C-P}$ 16.6, **C**-3), 123.6 (d, $^{1}J_{C-P}$ 202.9, $^{1}J_{C-P}$ 30.4, **C**-1), 83.5 (**C**-5''), 72.1 (-O-**C**H$_2$-Ar), 70.6 (**C**-1'), 69.1 (**C**-6''), 63.8 (d, $^{2}J_{C-P}$ 6.1, -O-**C**H$_2$-CH$_3$), 39.4 (**C**-5'), 35.1 (**C**-2''), 29.5 (**C**-4'), 29.3 (**C**-2'), 24.2 (**C**-3''), 23.6 (**C**-3'), 17.8 (**C**-4''), 16.3 (d, $^{3}J_{C-P}$ 6.0, -O-CH$_2$-**C**H$_3$). δ_P (162 MHz, CDCl$_3$) 17.2 (d, $^{1}J_{P-F}$ 1041). δ_F (376 MHz, CDCl$_3$) -63.9 (d, $^{1}J_{P-F}$ 1041). m/z LCMS (ES$^+$) 398.4 [M+H]$^+$. HRMS (ES$^+$) found [M+H]$^+$ 398.1878, $C_{20}H_{30}FNO_4P$ requires *M* 398.1897. Phosphinic acid intermediate m/z LCMS (ES$^+$) 396.4 [M+H]$^+$.

4-(diethoxyphosphoryl)benzoic acid (31)

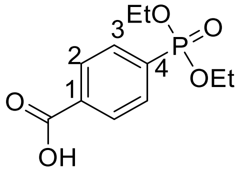

General procedure **B** was applied to 4-iodobenzoic acid. After workup, **31** (960 mg, 94%) was obtained as a dark brown oil. **ν**$_{max}$ (ATR) 3414 (-OH), 1718 (C=O), 1211 (P=O), 1014 (C-O). cm^{-1}. δ_H (400 MHz, CDCl$_3$) 10.62 (1H, s, -O***H***), 8.22 (2H, dd, *J* 8.3, $^{4}J_{H-P}$ 3.8, 2-***H***), 7.96 (2H, dd, $^{3}J_{H-P}$ 13.1, *J* 8.3, 3-***H***), 4.30 – 4.09 (4H, m, -O-C***H₂***-CH$_3$), 1.36 (6H, t, *J* 7.1, -O-CH$_2$-C***H₃***). δ_C (101 MHz, CDCl$_3$) 169.5 (**C**=O), 133.5 (d, $^{4}J_{C-P}$ 3.0, **C**-1), 133.2 (d, $^{1}J_{C-P}$ 186.5, **C**-4), 131.9 (d, $^{2}J_{C-P}$ 10.2, **C**-2), 130,0 (d, $^{3}J_{C-P}$ 15.1, **C**-2), 62.8 (d, $^{2}J_{C-P}$ 5.6, -O-**C**H$_2$-CH$_3$), 16.3 (d, $^{3}J_{C-P}$ 6.5, -O-CH$_2$-**C**H$_3$). δ_P (162 MHz, CDCl$_3$) 17.1. m/z LCMS (ES$^+$) 259.1 [M+H]$^+$; 517.2 [2M+H]$^+$ and m/z LCMS (ES$^-$) 257.2 [M-H]$^-$; 515.3 [2M-H]$^-$.

Diethyl [4-(carbonochloridoyl)phenyl]phosphonate (32)

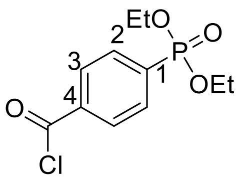

General procedure **E** was applied to **31**. After the volatiles were removed, **32** was obtained as a yellow oil. ν_{max} (ATR) 1719 (C=O), 737 (C-Cl) cm^{-1}. δ_H (400 MHz, CDCl$_3$) 8.22 (2H, dd, *J* 8.6, $^4J_{H-P}$ 3.6, 3-***H***), 8.02 – 7.94 (2H, dd, $^3J_{H-P}$ 12.9, *J* 8.6, 2-***H***), 4.29 – 4.07 (4H, m, -O-C***H₂***-CH$_3$), 1.36 (6H, t, *J* 7.1, -O-CH$_2$-C***H₃***). δ_C (101 MHz, CDCl$_3$) 132.3 (d, $^2J_{C-P}$ 10.0, ***C***-2), 131.0 (d, $^3J_{C-P}$ 14.9, ***C***-3), 62.7 (d, $^2J_{C-P}$, -O-***C***H$_2$-CH$_3$), 16.4 (d, $^3J_{C-P}$ 6.5, -O-CH$_2$-***C***H$_3$). δ_P (162 MHz, CDCl$_3$) 15.6.

Tert-butyl N-(2-{[4´-(diethoxyphosphoryl)phenyl]formamido}ethyl)carbamate (33)

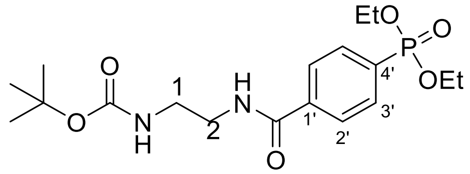

General procedure **Gb** was applied to **32** and *tert*-butyl (2-aminoethyl)carbamate. After workup, **33nn** (676 mg, 87%) was obtained as a yellow oil. **v**$_{max}$ (ATR) 3328 (N-H), 1701 (C=O), 1651 (C=O), 1241 (P=O) cm^{-1}. δ_H (700 MHz, CDCl$_3$) 7.89 – 7.83 (3H, m, 2'-***H***, -N***H***-), 7.72 (2H, dd, $^3J_{H-P}$ *1*3.0, *J* 8.0, 3'-***H***), 5.53 (1H, t, *J* 6.1, -N***H***-), 4.10 – 3.96 (4H, m, -C***H₂***-CH$_3$), 3.52 - 3.47 (2H, m, 2-***H***), 3.38 – 3.32 (2H, m, 1-***H***), 1.35 (9H, s, -C-(C***H₃***)$_3$), 1.25 (6H, t, *J* 7.1, -CH$_2$-C***H₃***). δ_C (176 MHz, CDCl$_3$) 166.9 (-NH-***C***(O)-Ar), 157.4 (-***C***(O)-NH-CH$_2$-), 131.8 (d, $^2J_{C-P}$ 10.2, ***C***-3'), 131,0 (d, $^1J_{C-P}$ 184.2, ***C***-4')128.7 (d, $^4J_{C-P}$ 12.3, ***C***-1'), 127.1 (d, $^3J_{C-P}$ 15.0, ***C***-2'), 79.7 (-***C***(CH$_3$)$_3$), 62.4 (d, $^2J_{C-P}$ 5.6, -***C***H$_2$-CH$_3$), 42.0 (***C***-2), 39.9 (***C***-1), 28.3 (-C(***C***H$_3$)$_3$), 16.2 (d, $^3J_{C-P}$ 6.3, -CH$_2$-***C***H$_3$). δ_P (162 MHz, CDCl$_3$) 17.4. HRMS (ES$^+$) found [M+H]$^+$ 401.1838; $C_{18}H_{30}N_2O_6P$ requires *M* 401.1842.

Diethyl (4-{[2'-(hex-5''-ynamido)ethyl]carbamoyl}phenyl)phosphonate (34)

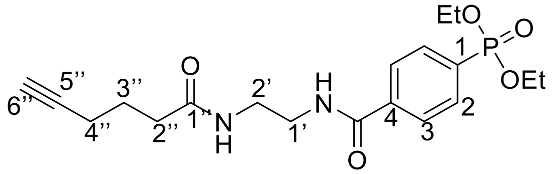

General procedure **F** was applied to **36**. After the volatiles were removed, deprotected amine (478 mg, 94%) was obtained as a yellow oil. **v**$_{max}$ (ATR) 3406 (N-H), 3277 (N-H), 1652 (C=O), 1228 (P=O) cm^{-1}. δ_H (700 MHz, CDCl$_3$) 8.82 (1H, s, -N***H***-C(O)-), 8.40 – 8.20 (2H, m, -N***H₂***), 8.00 (2H, dd, *J* 8.2, $^4J_{H-P}$ 3.7, 3-***H***), 7.71 (2H, dd, $^3J_{H-P}$ 13.0, *J* 8.2, 2-***H***), 4.07 – 3.93 (4H, m, -O-C***H₂***-CH$_3$), 3.75 – 3.65 (2H, m, 1´-***H₂***), 3.27 – 3.15 (2H, m, 2´-***H₂***), 1.22 (6H, t, *J* 7.1, -O-CH$_2$-C***H₃***). δ_C (176 MHz, CDCl$_3$) 167.4 (C=O), 137.2 (d, $^4J_{C-P}$

2.85, ***C***-4), 131.5 (d, $^2J_{C\text{-}P}$ 10.3, ***C***-2), 130.6 (d, $^1J_{C\text{-}P}$ 187.6, ***C***-1), 127.9 (d, $^3J_{C\text{-}P}$ 14.8, ***C***-3), 62.6 (d, $^2J_{C\text{-}P}$ 5.8, -O-***C***H_2-CH_3), 39.9 (***C***-1′), 37.7 (***C***-2′), 16.2 (d, $^3J_{C\text{-}P}$ 6.4, -O-CH_2-***C***H_3). □$_P$ (162 MHz, $CDCl_3$) 11.79. HRMS (ES$^+$) found $[M+H]^+$ 301.1327; $C_{13}H_{22}N_2O_4P$ requires *M* 301.1317. Subsequently, general procedure Ga was applied. After workup, **34** (208 mg, 54%) was obtained as a dark yellow solid. ν_{max} (ATR) 3481 (N-H), 3305 (≡C-H), 2260 (C≡C), 1650 (C=O), 1232 (P=O) cm^{-1}. δ_H (700 MHz, $CDCl_3$) 7.88 (2H, dd, *J* 8.2, $^4J_{H\text{-}P}$ 3.8, 3-***H***), 7.83 (2H, dd, $^3J_{H\text{-}P}$ 12.9, *J* 8.2, 2-***H***), 7.68 (1H, t, *J* 4.9, -1′-N***H***), 6.47 (1H, d, *J* 6.3, -2′-N***H***), 4.17 – 4.02 (4H, m, -O-C***H₂***-CH_3), 3.59 – 3.55 (2H, m, 2’-***H₂***), 3.51 (2H, m, 1’-***H₂***), 2.34 (2H, t, *J* 7.4, 2’’-***H₂***), 2.21 (2H, td, *J* 6.9, 2.7, 4’’-***H₂***), 1.93 (1H, t, *J* 2.7, 6’’-***H***), 1.83 (2H, p, *J* 6.9, 3’’-***H₂***), 1.31 (6H, t, *J* 7.1, -O-CH_2-C***H₃***). δ_C (176 MHz, $CDCl_3$) 174.3 (-CH_2-***C***(O)-NH-), 167.1 (-NH-***C***(O)-Ar), 137.6 (d, $^4J_{C\text{-}P}$ 3.3, ***C***-4), 132.0 (d, $^2J_{C\text{-}P}$ 10.0, ***C***-2), 131.6 (d, $^1J_{C\text{-}P}$ 188.3, ***C***-1), 127.0 (d, $^3J_{C\text{-}P}$ 15.1, ***C***-3), 83.2 (***C***-5’’), 69.3 (***C***-6’’), 62.4 (d, $^2J_{C\text{-}P}$ 5.5, -O-***C***H_2-CH_3), 41.8 (***C***-2’), 39.6 (***C***-1’), 34.9 (***C***-2’’), 24.1 (***C***-3’’), 17.8 (***C***-4’’), 16.3 (d, $^3J_{C\text{-}P}$ 6.4, -O-CH_2-***C***H_3). δ_P (162 MHz, $CDCl_3$) 17.2. HRMS (ES$^+$) found $[M+H]^+$ 395.1737; $C_{19}H_{28}N_2O_5P$ requires *M* 395.1736.

Ethyl (4-{[2’-(hex-5’’-ynamido)ethyl]carbamoyl}phenyl)phosphonofluoridate (35)

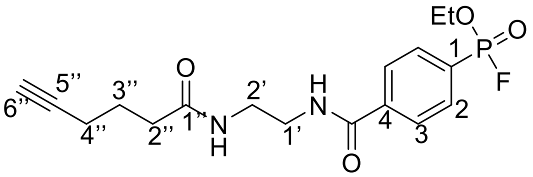

Phosphonate **34** was treated following the general procedure **D** using H_2O as solvent for the hydrolysis step and stirred for 3 h. After the fluorination, **35** (10 mg, 45%) was obtained as a brown oil. Column eluent 0-30% MeOH in DCM. ν_{max} (ATR) 3319 (C≡C-H), 1656 (C=O), 1554 (C=O), 1275 (P=O), 1042 (C-O) cm^{-1}. δ_H (599 MHz, $CDCl_3$) 7.99-7.86 (4H, m, 3-***H***, 2-***H***), 7.67 (1H, s, 1’-N***H***), 6.24 (1H, d, 2’-N***H***) 4.42 – 4.28 (2H, m, -O-C***H₂***-CH_3), 3.62 – 3.58 (2H, m, 1’-***H₂***), 3.58-3.51 (2H, m, 2’-***H₂***), 2.37 (2H, t, *J* 7.1, 2’’-***H₂***), 2.23 (2H, td, *J* 7.1, 2.7, 4’’-***H₂***), 1.96 (1H, t, *J* 2.7, 6’’-***H***), 1.83 (2H, p, *J* 7.1, 3’’-***H₂***), 1.43 (3H, t, *J* 7.0, -O-CH_2-C***H₃***). δ_C (151 MHz, $CDCl_3$) 174.9 (***C’’***-1), 166.6 (-NH-***C***(O)-Ar), 138.8 (d, $^4J_{C\text{-}P}$ 3.3, ***C***-4), 132.2 (d, $^2J_{C\text{-}P}$ 11.2, ***C***-2), 127.8 (dd, $^1J_{C\text{-}P}$ 200.7 $^1J_{C\text{-}F}$ 32.1, ***C***-1), 127.4 (d, $^3J_{C\text{-}P}$ 16.6, ***C***-3), 83.3 (***C***-5’’), 69.9 (***C***-6’’), 64.4 (d, $^2J_{C\text{-}P}$ 6.3, -O-***C***H_2-CH_3), 42.4 (***C***-1’), 39.7 (***C***-2’), 35.0 (***C***-2’’), 24.2 (***C***-3’’), 17.9 (***C***-4’’), 16.5 (d, $^3J_{C\text{-}P}$ 5.9, -O-CH_2-***C***H_3). δ_P (162 MHz, $CDCl_3$) 15.6 (d, $^1J_{P\text{-}F}$ 1045). δ_F (376 MHz, CDCl3) -58.8 (d, $^1J_{P\text{-}F}$ 1045). HRMS (ES$^+$) found $[M+H]^+$ 369.1367; $C_{17}H_{23}N_2O_4PF$ requires *M* 369.1379.

6-(Boc-amino)-1-hexanol (36)

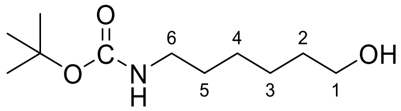

6-Amino-1-hexanol (2.0 g, 17 mmol, 1 eq) was dissolved in dry DCM (43 mL) in a round bottom flask equipped with a magnetic stirrer and a pressure equalising addition funnel. Subsequently, a solution of Boc_2O (3.8 g, 17 mmol, 1 eq) in dry DCM (43 mL) was added dropwise over 30 min. After stirring for 12 h under N_2, the reaction mixture was washed with saturated $NaHCO_3$ (2 x 25 mL) and H_2O (1 x 25 mL), dried, and concentrated to afford the protected amino alcohol **36** (3.4 g, 91%) as a light-yellow solid. $\mathbf{v}_{max}$ (ATR) 3352 (OH), 1695 (C=O) cm^{-1}. δ_H NMR (599 MHz, $CDCl_3$) 4.48 (1H, s, -O***H***), 3.62 (2H, t, *J* 6.6, 1-***H₂***), 3.10 (2H, t, *J* 7.1, 6-$\boldsymbol{H_2}$), 1.59 – 1.53 (2H, m, 2-$\boldsymbol{H_2}$), 1.51 – 1.45 (2H, m, 5-$\boldsymbol{H_2}$), 1.43 (9H, s, -C(C$\boldsymbol{H_3}$)$_3$), 1.41 – 1.30 (4H, m, 3-$\boldsymbol{H_2}$,4-$\boldsymbol{H_2}$). δ_C NMR (151 MHz, $CDCl_3$) 156.1 (C=O), 82.1 (-***C***(CH_3)$_3$), 62.7 (***C***-1), 40.4 (***C***-6), 32.6 (***C***-2), 30.1 (***C***-5), 28.4 (-C(***C***H_3)$_3$), 26.4 (***C***-4), 25.2(***C***-3). m/z LCMS (ES^+) 240.4 $[M+Na]^+$; 118.2 $[M\text{-}Boc]^+$. All the data are in accordance with the literature.[13]

Leishmania mexicana **cell culture**

L. mexicana promastigotes (MNYC/BZ/62/M379) were maintained at 26 °C in Schneider's Drosophila medium supplemented with 15% foetal bovine serum (FBS) and 1% Penicillin/Streptomycin. *Leishmania* promastigotes in the late log phase were then transferred into Schneider's Insect medium supplemented with 20 % FBS (pH 5.5) at a density of 5×10^5 parasites per mL. Metacyclic promastigotes were retrieved after 6 days of incubation at 26 °C.

RAW 264.7 cell culture

Briefly, RAW 264.7 (TIB-71, ATCC) were routinely cultured in T-25s flasks with Dulbecco's Modified Eagle's Medium (DMEM) supplemented with 10% foetal bovine serum (FBS) and 1% Penicillin/Streptomycin at 37°Cin a humidified incubator with 5 % CO_2.

L. mexicana **– RAW cell interactome assays**

RAW cell cultures were inoculated with *L. mexicana* metacyclic promastigotes (in Schneider's Drosophila medium (pH 5.5) supplemented with 20% FBS and 1% Pen/Strep) at a multiplicity of infection (MOI) of 10:1 and incubated at 37 °C for 4 hours in a humidified incubator with 5 % CO_2. Subsequently, the infected cells were treated with 10 μM of probe **7** for 1 hour at 26 °C. The infected cells were then gently washed 3 times with warm PBS to remove remaining parasites, cells were scraped, and centrifuged at 500 x g for 5 minutes at room temperature. Cells were resuspended in ice-cold lysis buffer (25 mM Tris–HCl (pH 7.4), 150 mM NaCl, 1% Triton X-100, and 5% glycerol) supplemented with a cOmplete Mini, EDTA-free

protease inhibitor cocktail (Roche 1183170001) and agitated in microcentrifuge tubes for 30 min at 4 °C. The samples were then centrifuged at 16,000 x g for 20 min at 4 °C, and the supernatant was collected in fresh tubes and used in downstream assays.

Protein extraction from *Leishmania* parasites

Cells were harvested by centrifugation (1000 × g, 5 min, 4 °C), washed three times with cold Dulbecco's phosphate-buffered saline (PBS, pH 7.4), and lysed with lysis buffer (25 mM Tris–HCl (pH 7.4), 150 mM NaCl, 1% Triton X-100, and 5% glycerol). For lysate labelling, cysteine protease inhibitor E-64 (10 μM) was added, while for whole-parasite labelling, a cOmplete Mini, EDTA-free protease inhibitor cocktail (Roche 1183170001) was used. The resulting lysates were centrifuged (13,000 × g, 10 min, 4 °C) to remove insoluble material. Protein concentration in each sample was determined using Pierce Rapid Gold BCA Protein Assay Kit (ThermoFisher) following the manufacturers' protocol, and homogenates were adjusted to the stated protein concentration (1-2 mg/mL).

Lysate ABP labelling

L. mexicana lysates (1.5 mg/mL) were incubated with the stated ABPs (15 μM) for 30 min at room temperature, followed by lysis using the previously described procedure.

Live parasites ABP labelling

Stationary-phase *L. mexicana* promastigotes were incubated with the stated ABPs (15 μM) for 30 minutes at 26 °C, followed by lysis using the previously described procedure. The lysis buffer included cOmplete Mini, EDTA-free protease inhibitor cocktail (Roche 1183170001).

Bioorthogonal Cu-Catalysed cycloaddition

Rh-N_3/biotin- N_3 (50 μM), $CuSO_4$ (1 mM), TBTA (0.1 mM), and sodium ascorbate (1 mM) were added to the ABP-labelled tissue homogenate (1.5 mg/mL). The mixture was incubated at room temperature for 1 h, with periodic mixing. Upon completion, the reaction was stopped by adding 4X LDS sample buffer (200 mM Tris–HCl, pH 6.8, 400 mM DTT, 8% LDS, 0.04% bromophenol blue, and 40% glycerol) and incubated at 95 °C for 5 minutes. Proteins were resolved on a 12.5% (w/v) SDS-PAGE, and fluorescent bands were

detected using a Typhoon 9400 Variable Mode Imager. Emission filter: 580 BP 30 Cy3, TAMRA, AlexaFluor546; Laser: green (532 nm). Gels were subsequently stained with Coomassie Brilliant Blue R-250 (CBB), and the images were documented. ABPs and reporter tag stocks were prepared in DMSO (Sigma Aldrich).

L. mexicana infectivity assay

The efficacy of the Z-Pro-Prolinal (ZPP) inhibitor was assessed as previously described with modifications.[14] Briefly, RAW cells were harvested, washed with PBS 1X, and seeded in 96-well plates at 5×10^4 cells per well, then incubated overnight at 37 °C, 5% CO_2 in a humidified incubator. *L. mexicana* metacyclic promastigotes were treated for 2 hours with 100 µM of ZPP (Sigma-Aldrich) at 26 °C before being inoculated at densities of 25 x 10^4, 12.5 x 10^4 and 62.5 x 10^3 per well, respectively. Plates were incubated for another 4 hours at 37°C, 5 % CO_2 in a humidified incubator. Subsequently, *L. mexicana* infected RAW cells were washed five times with DMEM (2% FBS; 1% Penicillin/Streptomycin) to remove remaining *Leishmania* parasites. Cells were then treated with 0.05% (v/v) SDS in Schneider's media for 45 seconds before the addition of 180 µL of Schneider's Insect medium (pH 7, 15% FBS). Plates were sealed with parafilm and incubated for 48 hours at 26 °C. Afterward, 10 µL of resazurin (125 µg/mL) was added to each well, followed by a 4 h incubation at 26 °C. Parasite viability was assessed using a fluorescent plate reader (Biotek; 560EX nm/ 600 EM nm). All experiments described above were carried out in triplicate.

L. mexicana competitive ABPP with ZPP coupled with TMT-LC-MS/MS

Live parasite competition assays were conducted by incubating *L. mexicana* metacyclic promastigotes with 100 µM ZPP or DMSO for 2 hours at 26 °C, followed by addition of probe **7** (10 µM) and further incubation for 1 hour. After this period, and following previously described procedures, the samples were lysed, and biotin-N_3 was attached via click chemistry. Subsequently, the labelled proteomes were enriched, reduced, alkylated, digested, and TMT tagged (*vide infra*). Proteins were then identified with LC-MS/MS.

Protein precipitation

After biotin attachment by biorthogonal click chemistry, proteins were precipitated by the addition of 9 volumes of ice-cold MeOH and stored overnight at -80 °C. Subsequently, lysates were centrifuge at 10,000 x g for 10 min at 4 °C and washed with ice-cold MeOH (2X) to remove unreacted ABPs, biotin-N_3, and click chemistry reagents. Finally, pellets were air-dried for 30 min.

Affinity enrichment

Precipitated proteins were redissolved in a minimal volume of 2% SDS (in PBS) and diluted to a final concentration of 0.1% SDS. NeutrAvidin-Agarose beads (50 µL per sample), freshly washed three times with 4 volumes of 0.1% SDS (in PBS), were added to each of the samples, and the mixture were rotated on an end-over-end rotating shaker for 2 h at room temperature. The beads were then washed three times with 0.5% SDS in PBS, three times with 6M urea in PBS, three times with PBS, and once with 50 mM TEAB buffer. Each washing was performed with 500 µL of the washing solution, and centrifugation was carried out at 1,500 × g for 2 min at room temperature.

On-bead reduction, alkylation, and tryptic digestion

Washed beads from the previous affinity enrichment step were treated with 200 µL 10 mM TCEP in 50 mM TEAB buffer for 45 min at 30 °C. After this period, the samples were washed by the addition of 400 µL of 50 mM TEAB buffer, centrifuged at 1500 x g for 2 min, and the supernatant removed. Subsequently, the beads were resuspended in 200 µL of 15 mM α-iodoacetamide in 50 mM TEAB buffer and incubated in the dark for 45 min. The beads were again washed with 50 mM TEAB buffer, resuspended in 200 µL of fresh 100 mM TEAB buffer, and treated with 4 µg of sequencing-grade modified trypsin at 37 °C for 16 h. The samples were centrifuged at 5000 x g for 5 minutes, and the supernatant collected. The remaining beads were washed twice with 50 µL of 50% ACN containing 0.1% FA, centrifuged at 1500 x g for 2 min, and the supernatants were mixed. The collected tryptic peptides were acidified to pH = 3 using formic acid and evaporated to dryness. The peptides were then redissolved in 0.1% (v/v) formic acid solution in water and desalted using Pierce™ Peptide Desalting Spin Columns (Thermo Scientific; CN: 89851) following manufacturer's instructions. The remaining peptides were finally evaporated to complete dryness under a vacuum.

TMT labelling

Dried and desalted tryptic peptides were resuspended in 100 µL of 100 mM TEAB. Subsequently, 41 µL of the previously equilibrated TMT10plex Mass Tag Labelling reagents (Thermo Scientific, CN: 90110) were added to each sample and incubated for 1 h at room temperature, followed by the addition of 8 µL of 5% hydroxylamine to each sample and incubated for further 15 min to quench the reaction. Upon completion, equal amounts of each sample were combined in a new microcentrifuge tube and evaporated

to dryness. Finally, the peptides were desalted using Pierce™ Peptide Desalting Spin Columns (Thermo Scientific; CN: 89851) following manufacturer's instructions and evaporated to dryness.

Nano LC-MS/MS data acquisition.

The LC-MS/MS analyses of TMT-labelled peptides were performed by the Proteomics Core Facility of The Institute of Cancer Research, London, UK, on an Orbitrap Ascent Mass Spectrometer (Thermo Fisher Scientific) coupled with a Thermo Scientific Ultimate 3000 RSLCnano UHPLC system (Thermo Fisher Scientific). Desalted and TMT-labelled tryptic peptides dissolved in 0.1% formic acid (FA) were first loaded onto an Acclaim PepMap 100 C18 trap column (5 µm particle size, 100 µm ID X 20 mm, TF164564) heated to 45 °C using 0.1% FA/H_2O with a flow rate of 10 µL/min, then separated on a Waters nanoEase M/Z Peptide BEH C18 Column (1.7 µm particle size, 130Å, 75 µm ID X 250 mm, 186008795) with a 5% to 35% ACN gradient in 0.1% FA over 150 min at a flow rate of 300 nL/min. The full MS spectra (*m/z* 375 to 1,500) were acquired in Orbitrap at 120,000 resolutions with an AGC target value of $4e^5$ for a maximum injection time of 251 ms. High-resolution HCD MS2 spectra were generated in positive ion mode using a normalised collision energy of 38% within a 0.7 *m/z* isolation window using quadrupole isolation. The AGC target value was set to $10e^4$, and the dynamic exclusion was set to 45 s. The MS2 spectra were acquired in Orbitrap with a maximum injection time of 80 ms at a resolution of 45,000 with an instrument determined scan range beginning at *m/z* 100. To ensure quality peptide fragmentation a number of filters were utilised, including peptide monoisotopic precursor selection, minimum intensity exclusion of 50000 and exclusion of precursor ions with unassigned charge state as well as charge state of +1 or superior to +7 from fragmentation selection. To prevent repeat sampling, a dynamic exclusion with exclusion count of 1, exclusion duration of 45 s, mass tolerance window of +/- 7 ppm and isotope exclusion were used.

Proteomics MS data processing (Statistical Analysis)

All raw LC-MS/MS data were processed using MaxQuant software[15] version 1.6.3.4 with integrated Andromeda database search engine.[16] The MS/MS spectra were queried against *L. mexicana* sequences from UniProt KB (8,559 sequences). The following search parameters were used: reporter ion MS2 with multiplicity 10-plex TMT, trypsin digestion with maximum 2 missed cleavages, carbamidomethylation of cysteine as a fixed modification, oxidation of methionine, acetylation of protein N-termini as variable modifications, minimum peptide length of 6, a maximum number of modifications per peptide set at 5, and protein false discovery rate (FDR) 0.01. Appropriate correction factors for the individual TMT channels, accounting for both lysine side-chain labelling and peptide N-terminal labelling, as per the TMT-10plex kits used (Thermo Fisher Scientific) were configured into the database search. The proteinGroups.txt files from

the MaxQuant search outputs were processed using Perseus software version 1.6.10.50.[17] Sequences only identified by site, reverse sequences, and potential contaminants were filtered out. The reporter intensities were normalised by Z-score and transformed to $\log_2$ scale. A modified *t*-test with permutation-based FDR statistics (250 permutations) was applied to compare the probe treatments versus control groups and volcano plots were generated in R 64-bit version 4.2.3 using R packages ggplot2 (version 3.4.2) and EnhancedVolcano (version 1.16.0).

Protein Structure Prediction and validation

The protein structures were predicted using the latest AlphaFold3 tool.[18] Serine hydrolases folds are generally highly conserved, so the catalytic domains were predicted with high confidence scores. In all cases, the active sites for each of the serine hydrolases were identified using known homologous hydrolases and confirmed by visually identifying the putative catalytic triad using interactive computer graphics (PyMOL).[19]

Covalent Docking

GOLD, developed by the Cambridge Crystallographic Data Centre,[1,2] was used to dock and score all probes using a genetic algorithm (GA) into each protein's active sites. In order to covalently dock the ligands, the probe structures were modified by replacing the leaving fluoride with an oxygen atom, which, in the docking protocol, represents the oxygen atom of the active serine's side chain covalently bound to the phosphoester. ChemPLP was used as the scoring function. ChemPLP is an empirical scoring function optimised for pose prediction using piecewise linear potentials. It includes hydrogen bonding and Van der Waals terms to model geometric complementarity between the protein and docked ligands efficiently. Multiple binding poses for each probe generated by the GA were visually inspected to ensure chemically reasonable interactions. The highest scoring poses were taken forward for further evaluation.

Appendix

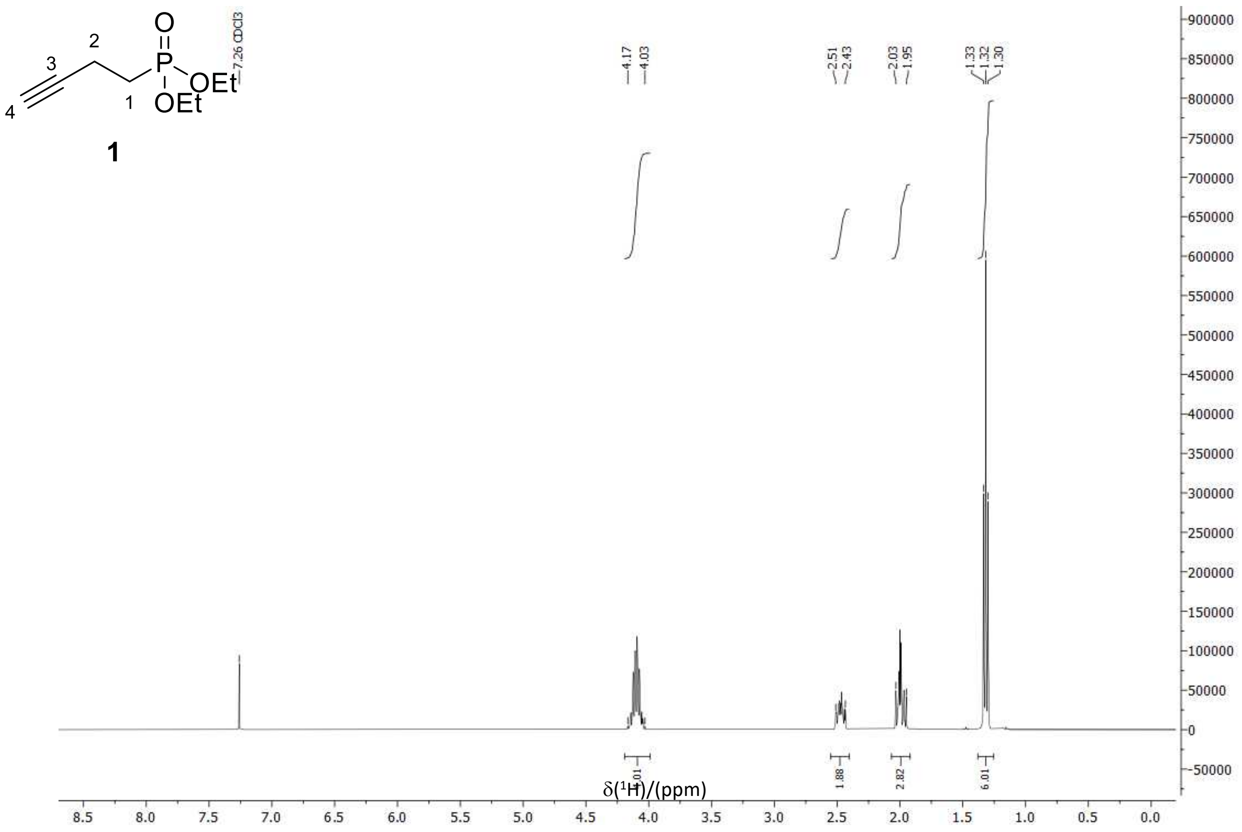

O
P
OEt
OEt
1
2
3
4
1
7.26 CDCl3
4.17
4.03
2.51
2.43
2.03
1.95
1.33
1.32
1.30
2.01
1.88
2.82
6.01
δ(¹H)/(ppm)

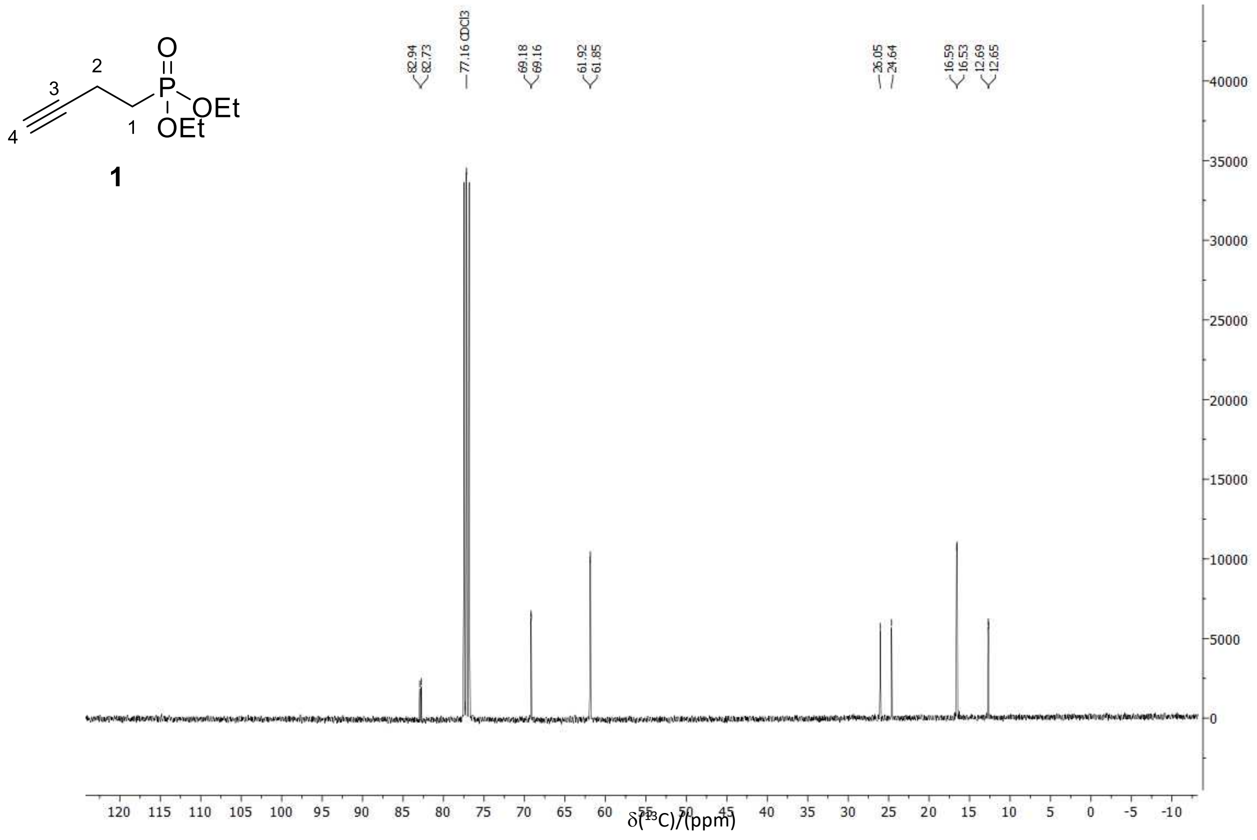

O
P
OEt
OEt
1
2
3
4
1
82.94
82.73
77.16 CDCl3
69.18
69.16
61.92
61.85
26.05
24.64
16.59
16.53
12.69
12.65
δ(¹³C)/(ppm)

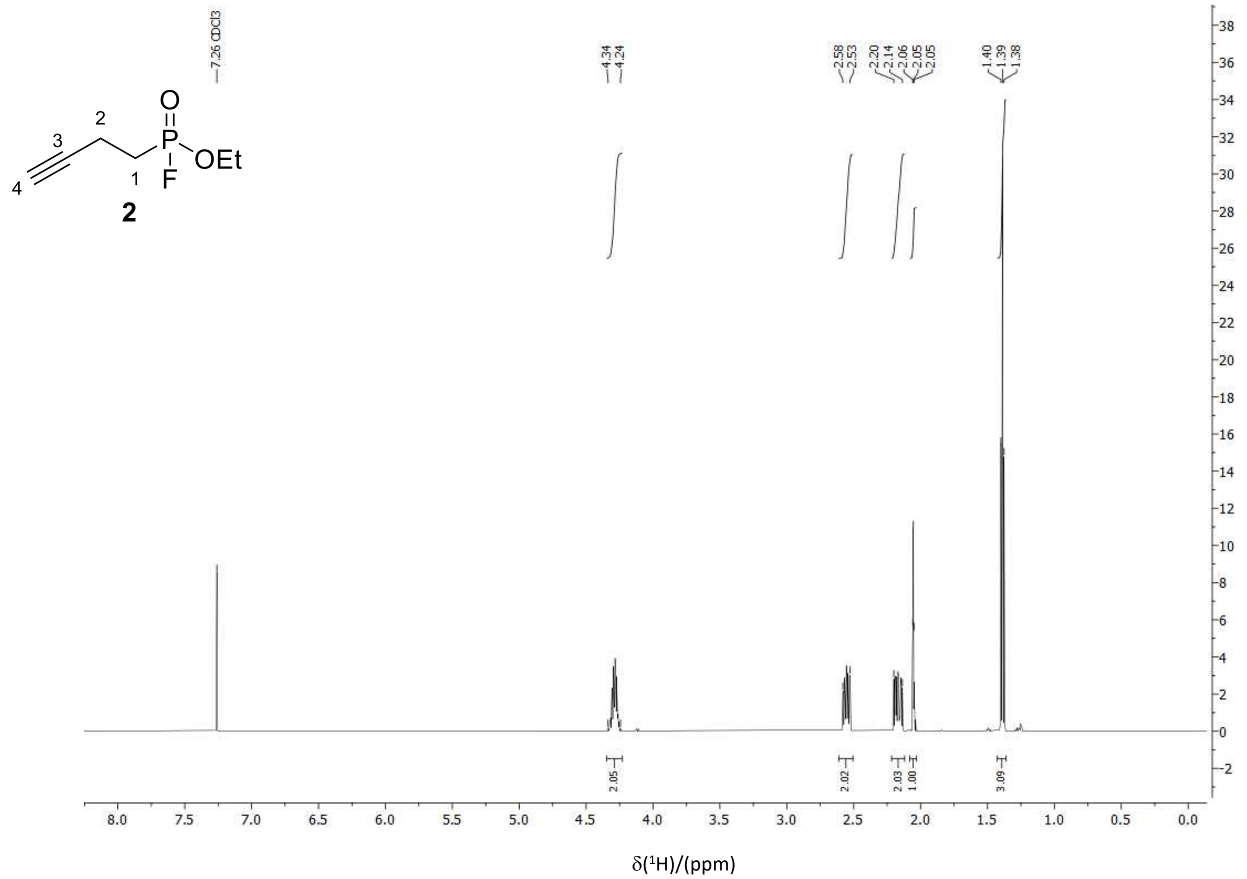
O
P
OEt
F
1
2
3
4
2
7.26 CDCl3
4.34
4.24
2.58
2.53
2.20
2.14
2.06
2.05
2.05
2.05
1.40
1.39
1.38
2.05
2.02
2.03
1.00
3.09
δ(1H)/(ppm)

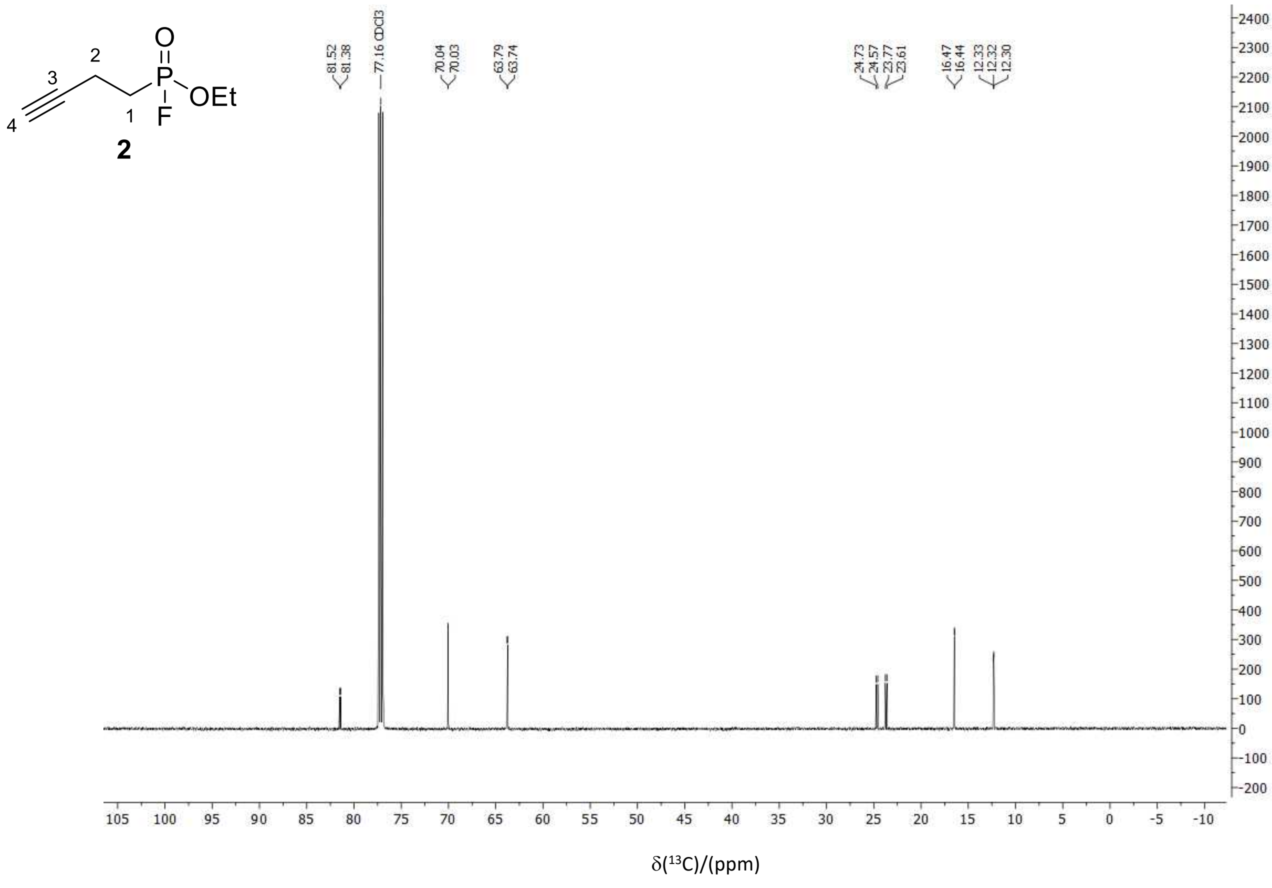
O
P
OEt
F
1
2
3
4
2
81.52
81.38
77.16 CDCl3
70.04
70.03
63.79
63.74
24.73
24.57
23.77
23.61
16.47
16.44
12.33
12.32
12.30
δ(13C)/(ppm)

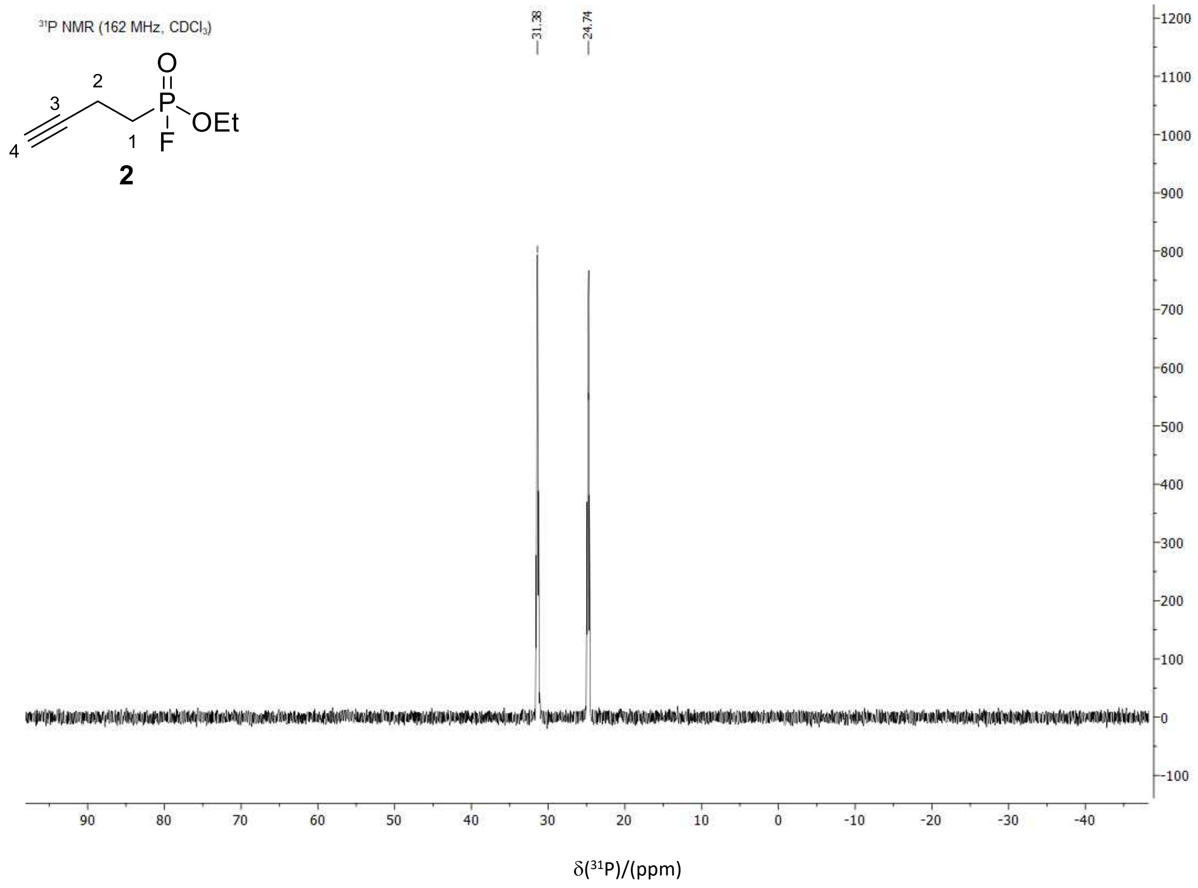

31P NMR (162 MHz, CDCl3)
O
2
3
P
OEt
1
F
4
2
31.38
24.74
δ(31P)/(ppm)

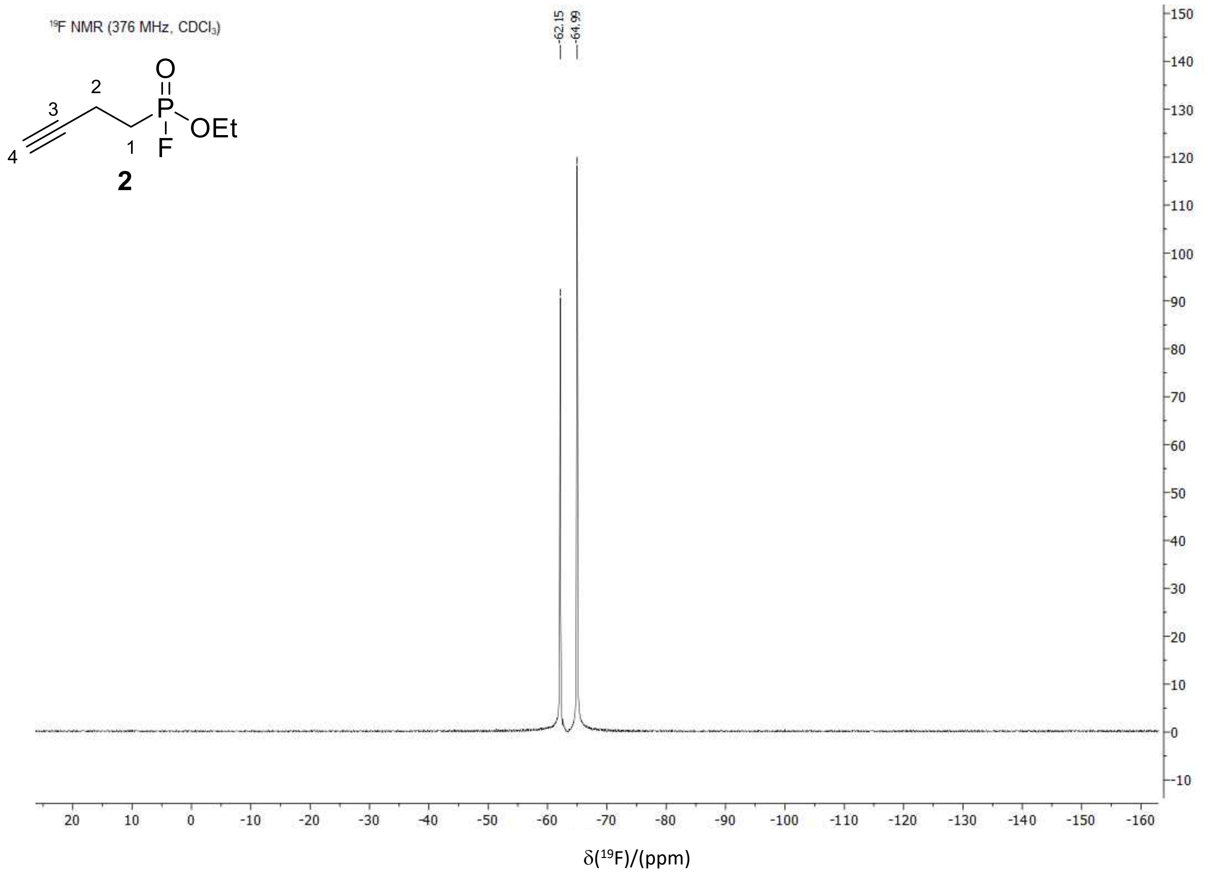

19F NMR (376 MHz, CDCl3)
O
2
3
P
OEt
1
F
4
2
-62.15
-64.99
δ(19F)/(ppm)

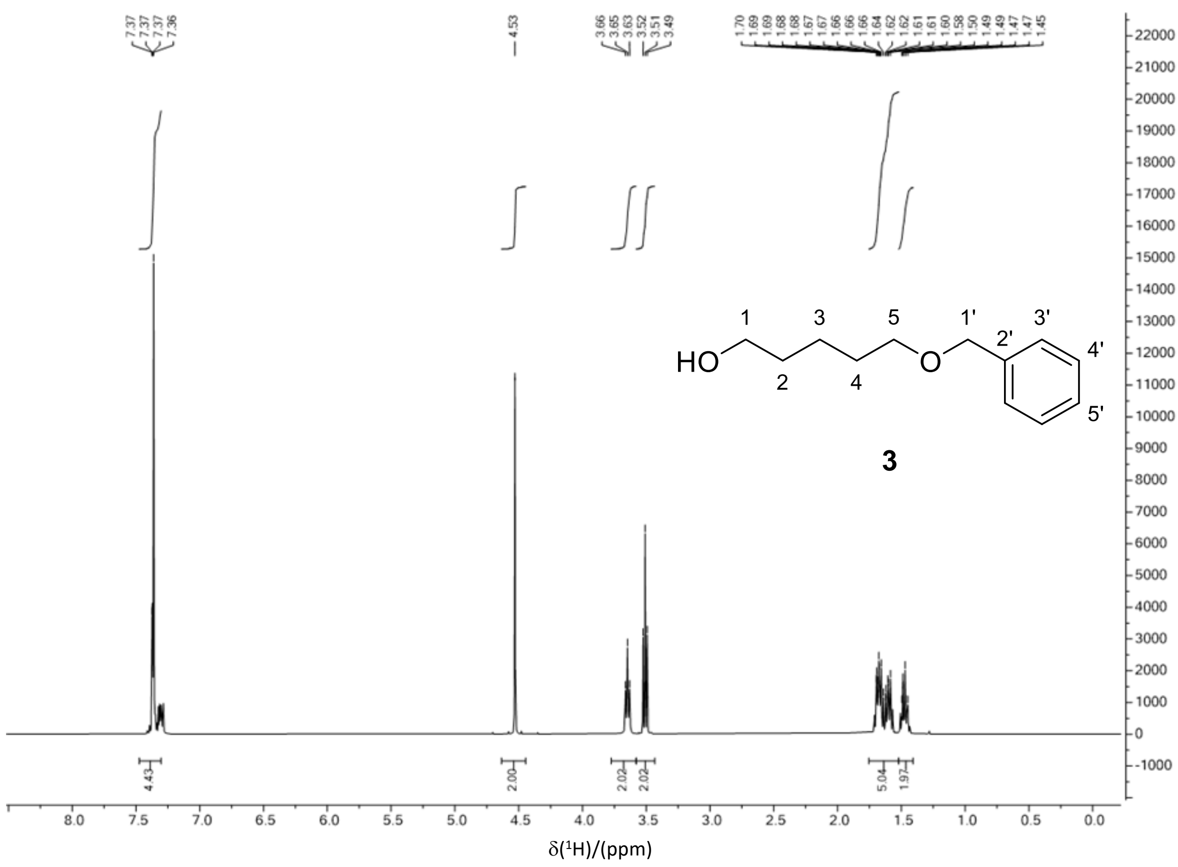
7.37
7.37
7.37
7.36
4.53
3.66
3.65
3.63
3.52
3.51
3.49
1.70
1.69
1.69
1.68
1.68
1.67
1.67
1.66
1.66
1.66
1.64
1.62
1.62
1.62
1.61
1.61
1.60
1.58
1.50
1.49
1.49
1.47
1.47
1.45
HO
O
1
2
3
4
5
1'
2'
3'
4'
5'
3
4.43
2.00
2.02
2.02
5.04
1.97
δ(¹H)/(ppm)

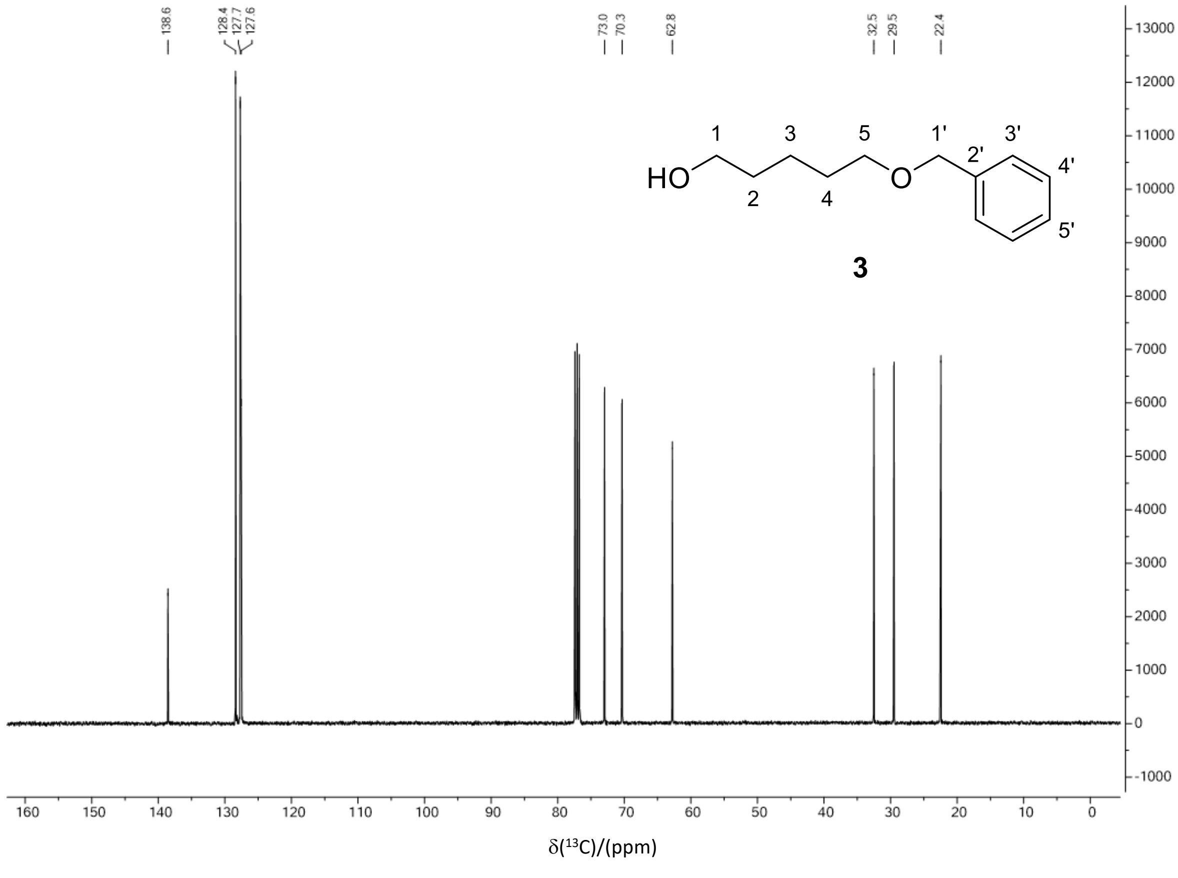
138.6
128.4
127.7
127.6
73.0
70.3
62.8
32.5
29.5
22.4
HO
O
1
2
3
4
5
1'
2'
3'
4'
5'
3
δ(¹³C)/(ppm)

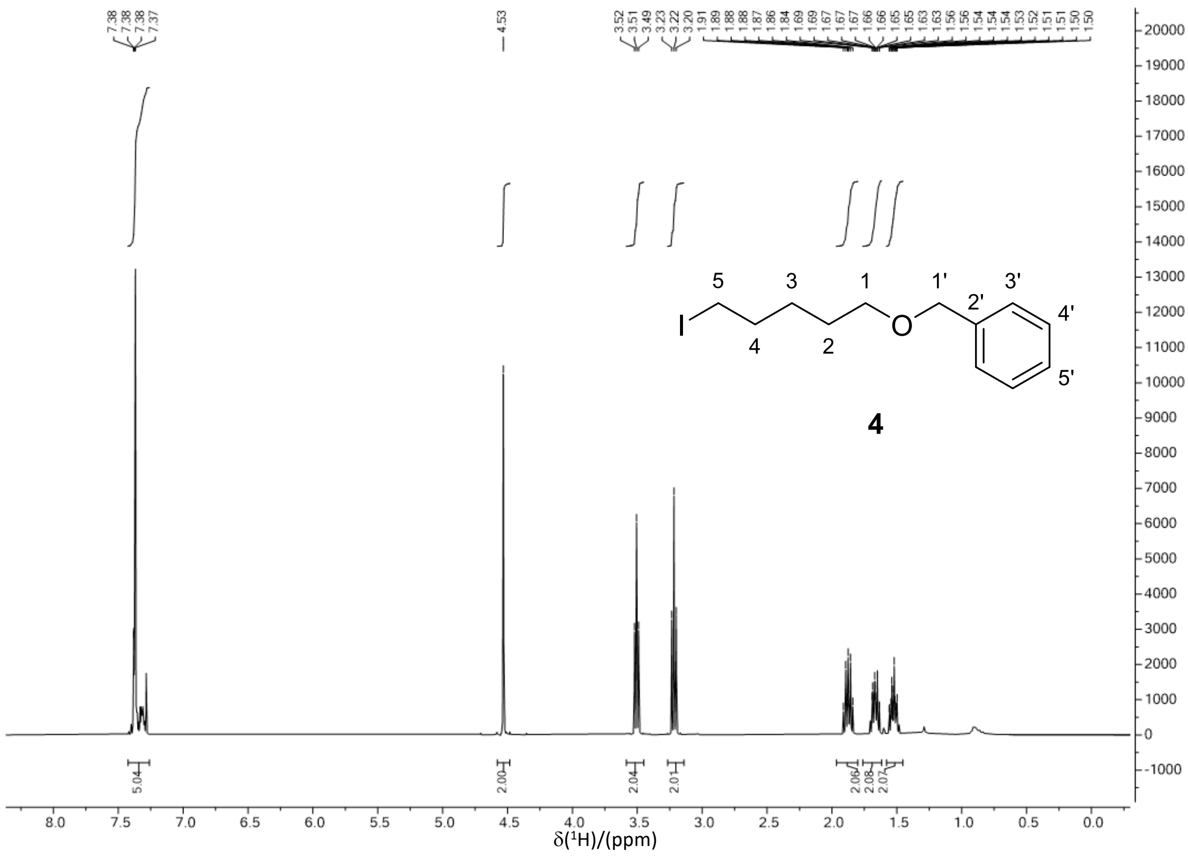
δ(¹H)/(ppm)
5 3 1 1' 3'
2' 4'
I O
4 2 5'
4

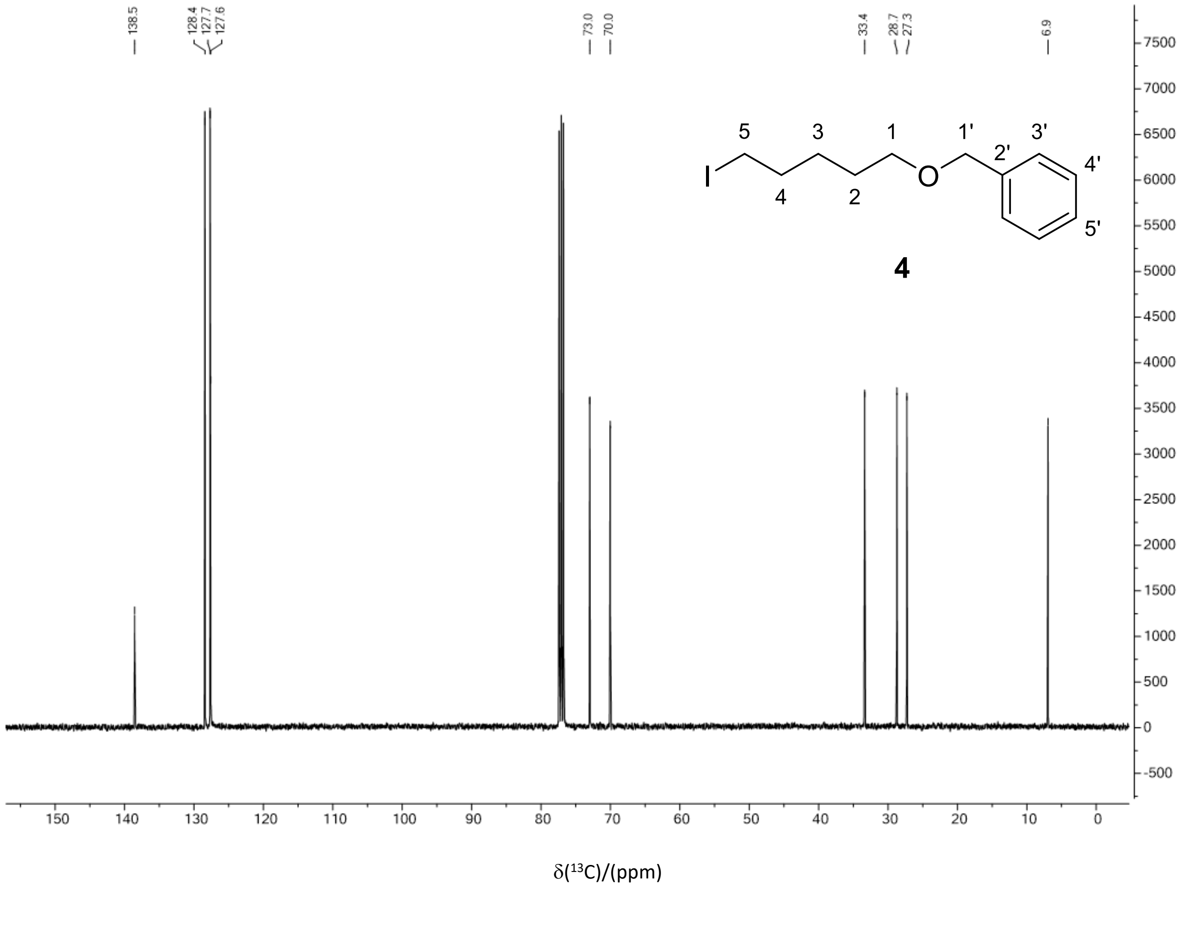
δ(¹³C)/(ppm)
5 3 1 1' 3'
2' 4'
I O
4 2 5'
4

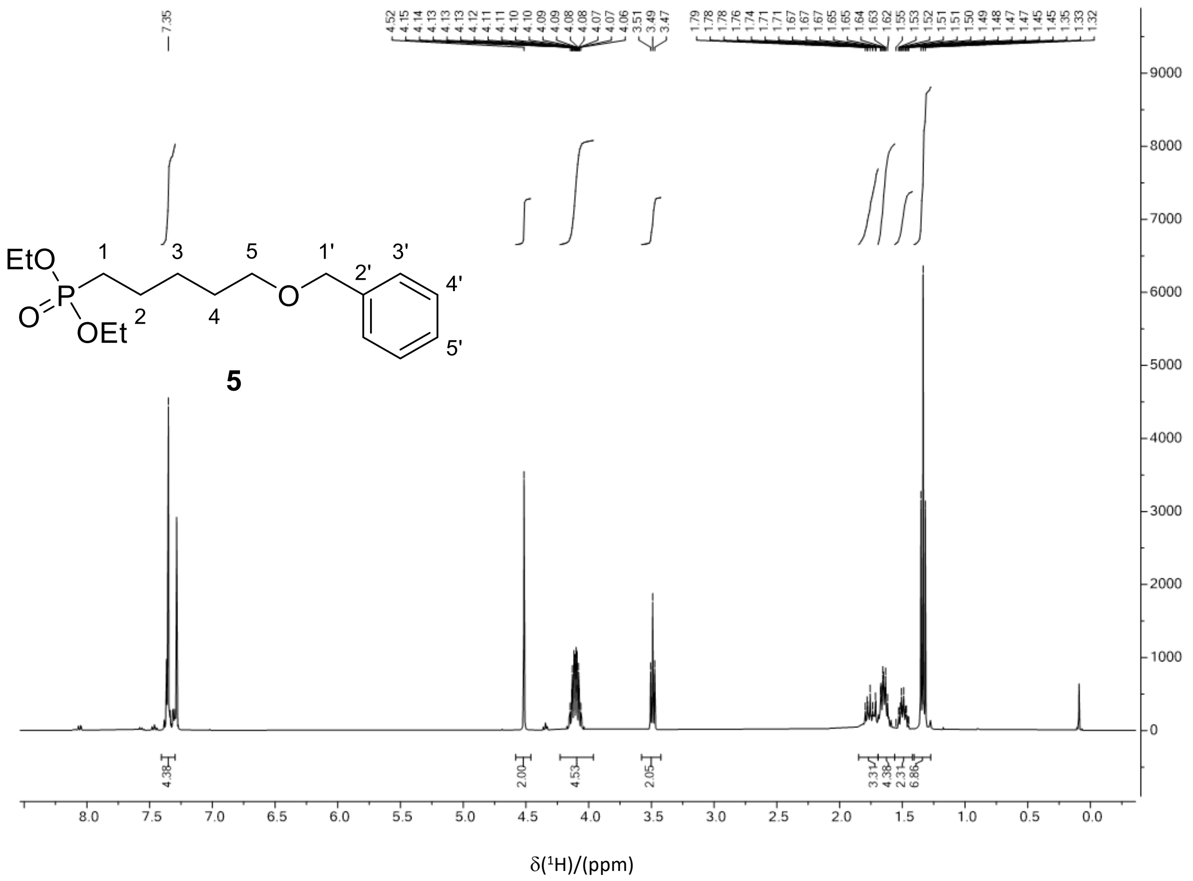

EtO
O=P
OEt
1
2
3
4
5
O
1'
2'
3'
4'
5'
5
δ(¹H)/(ppm)

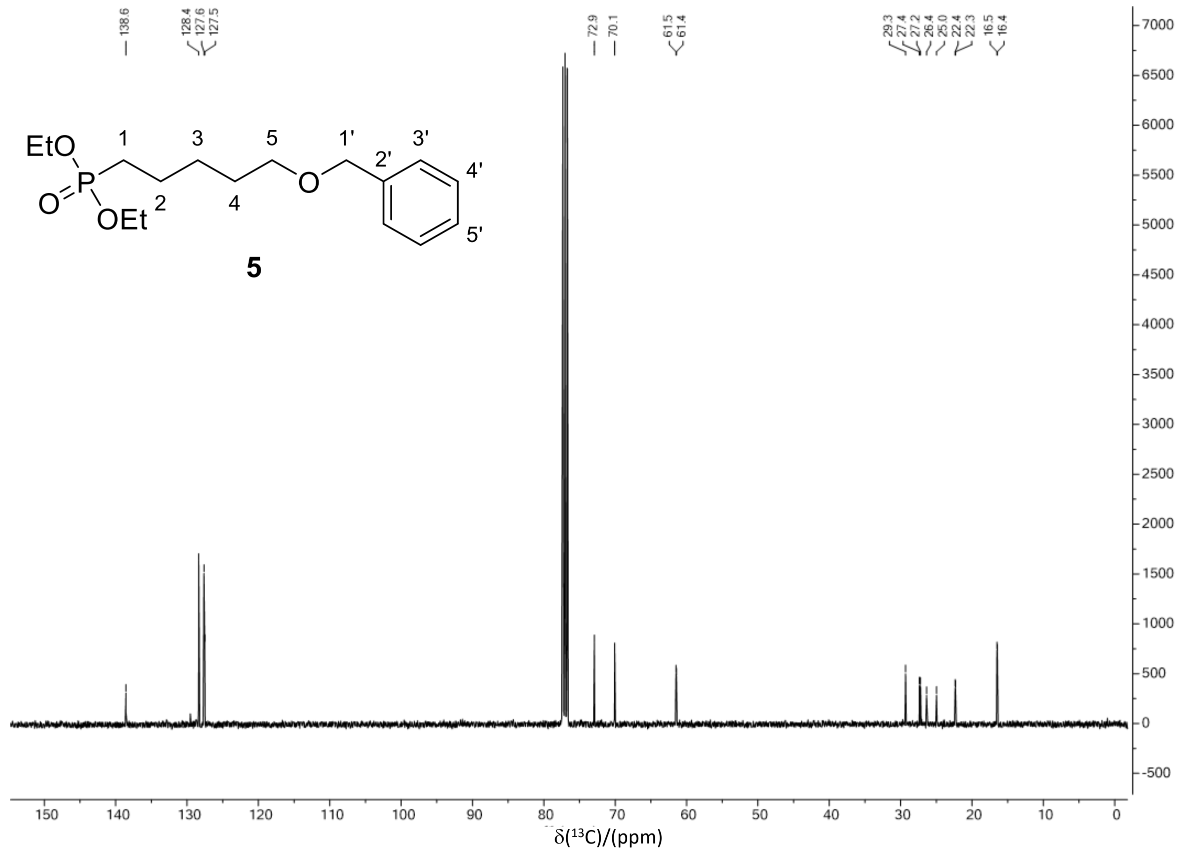

EtO
O=P
OEt
1
2
3
4
5
O
1'
2'
3'
4'
5'
5
δ(¹³C)/(ppm)

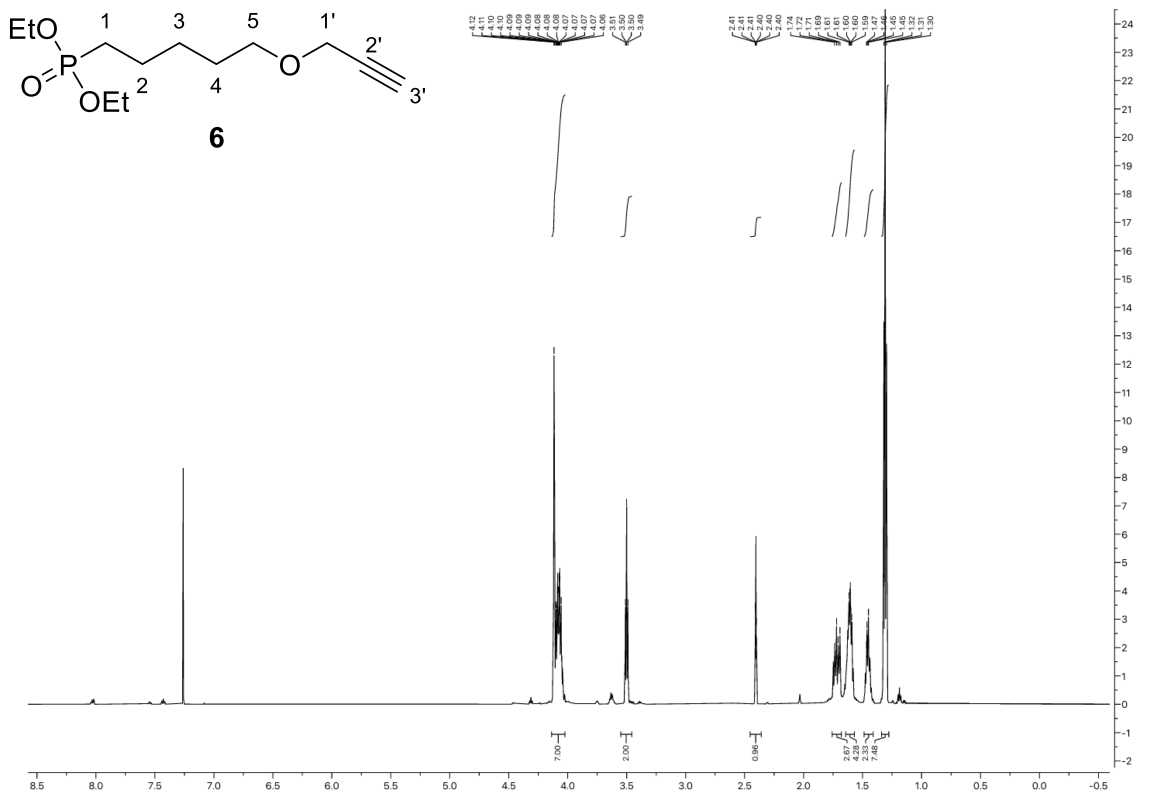

δ(^{1}H)/(ppm)

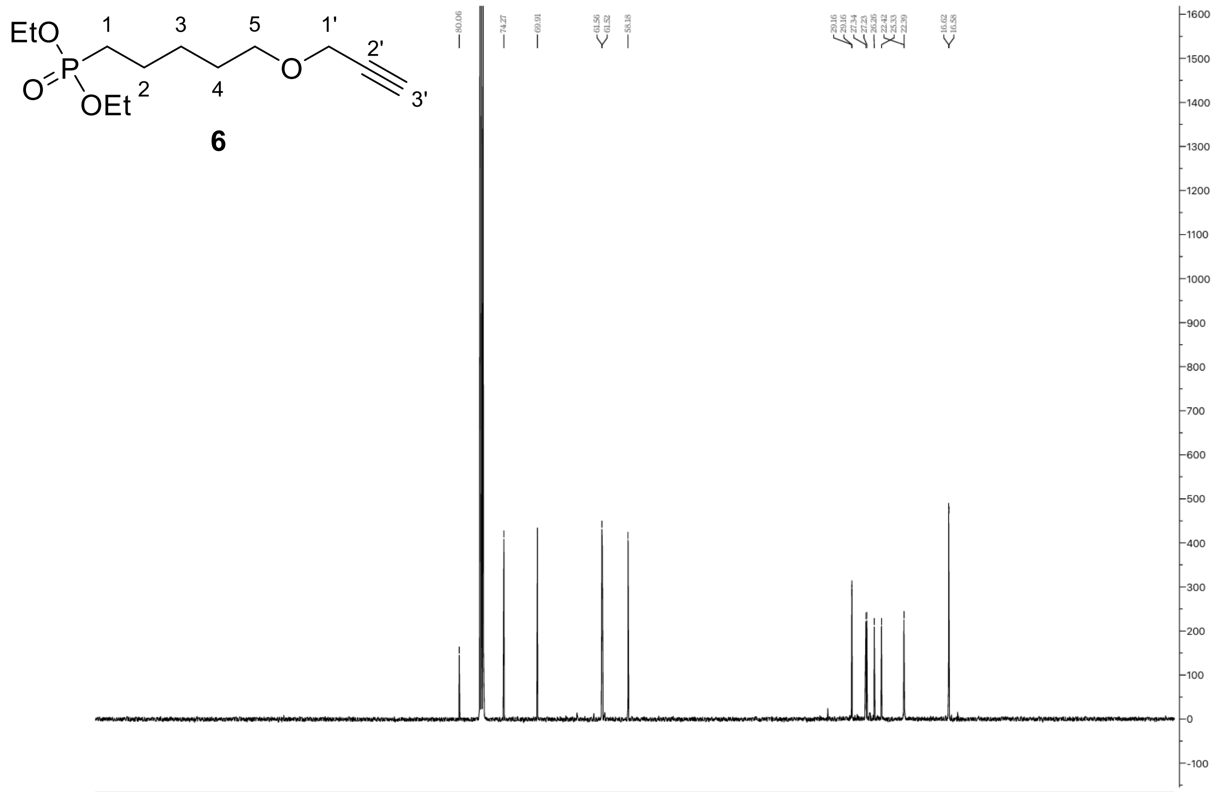

δ(^{13}C)/(ppm)

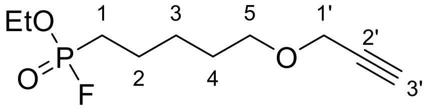

7

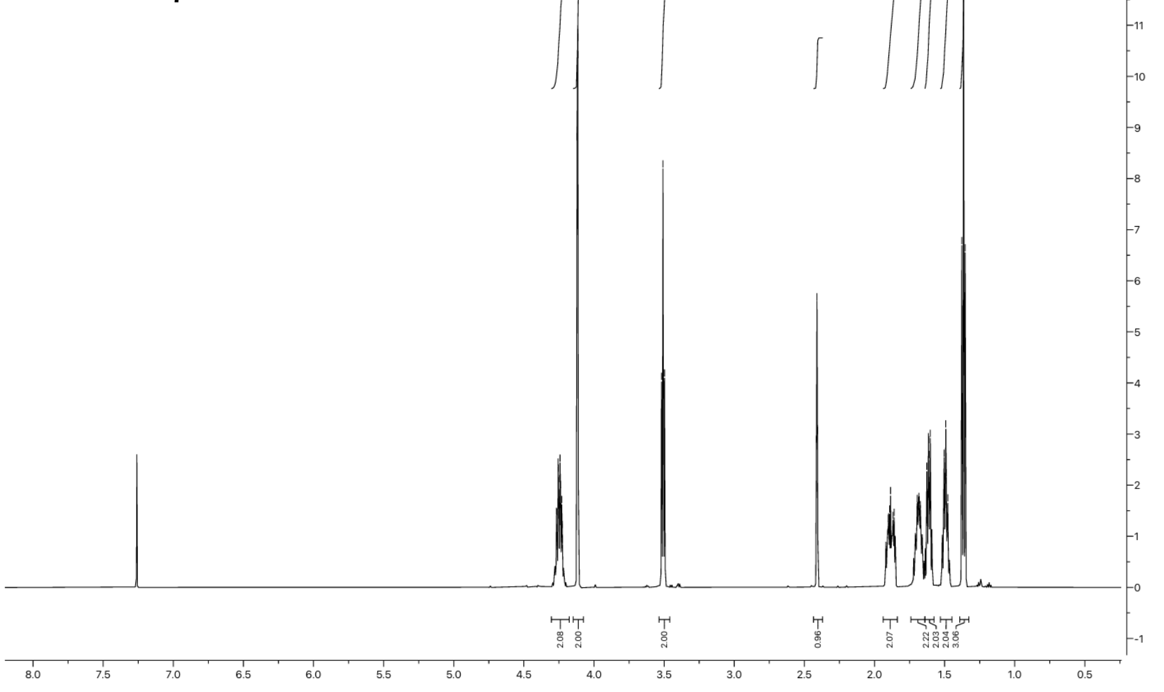

δ(¹H)/(ppm)

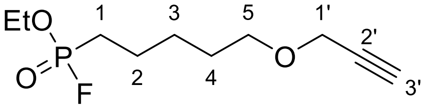

7

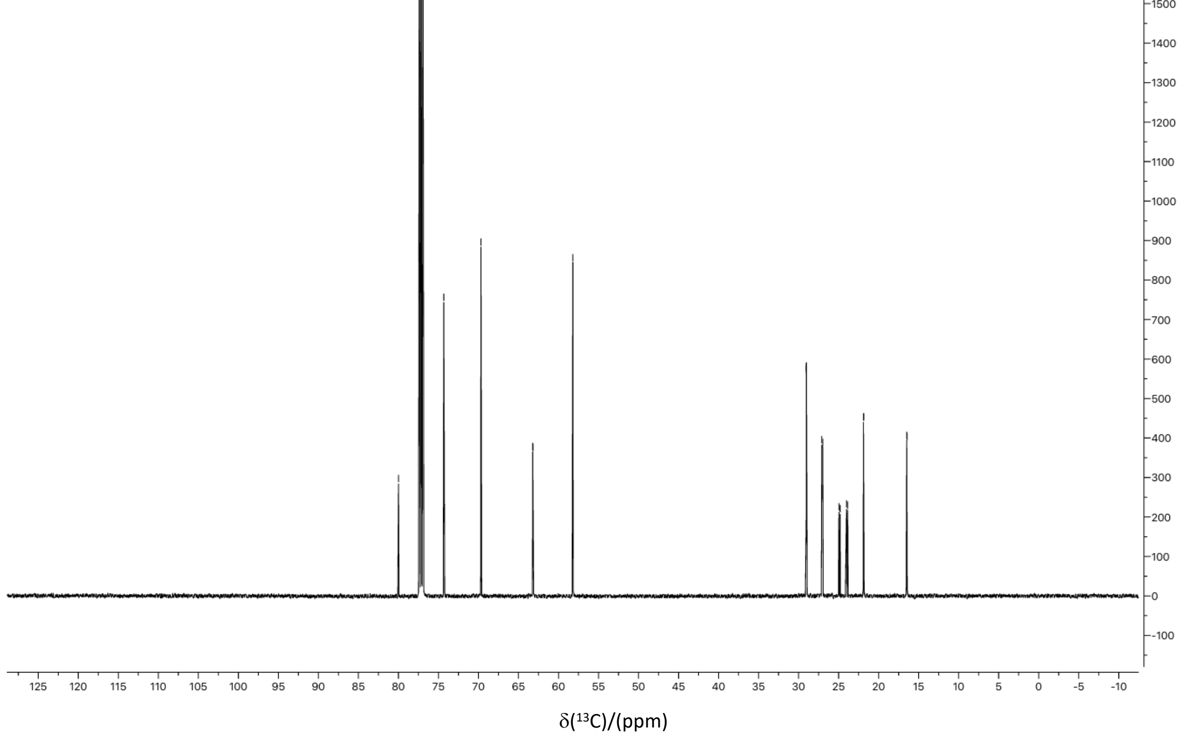

δ(¹³C)/(ppm)

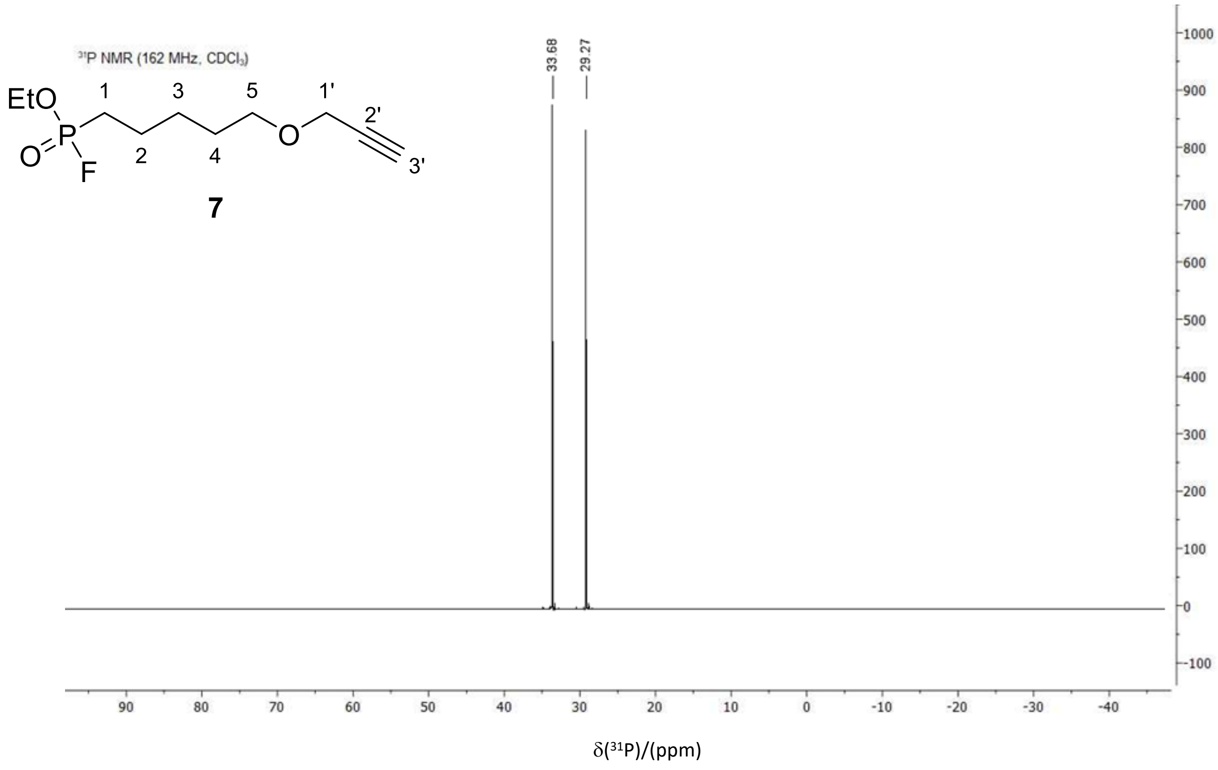

31P NMR (162 MHz, CDCl3)
33.68
29.27
EtO
O
P
F
1
2
3
4
5
1'
2'
3'
7
δ(31P)/(ppm)

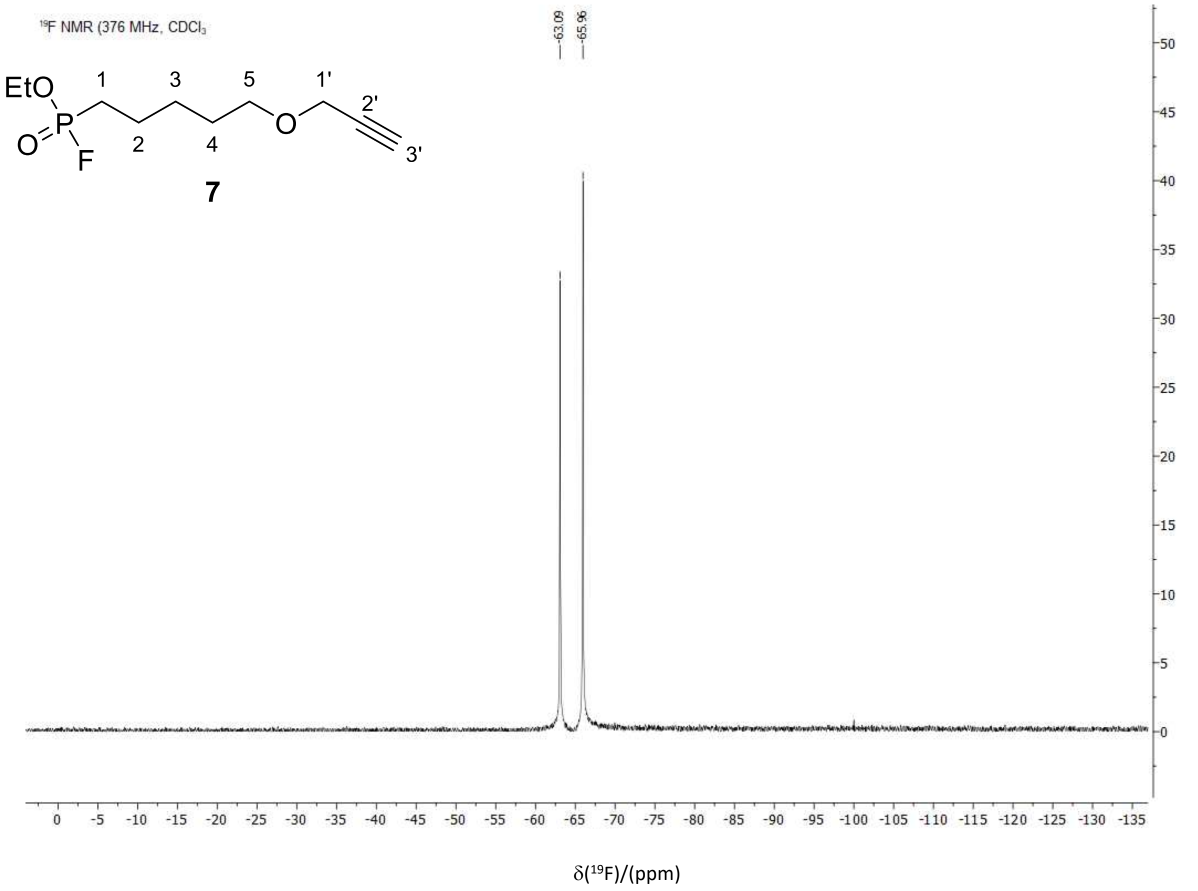

19F NMR (376 MHz, CDCl3
-63.09
-65.96
EtO
O
P
F
1
2
3
4
5
1'
2'
3'
7
δ(19F)/(ppm)

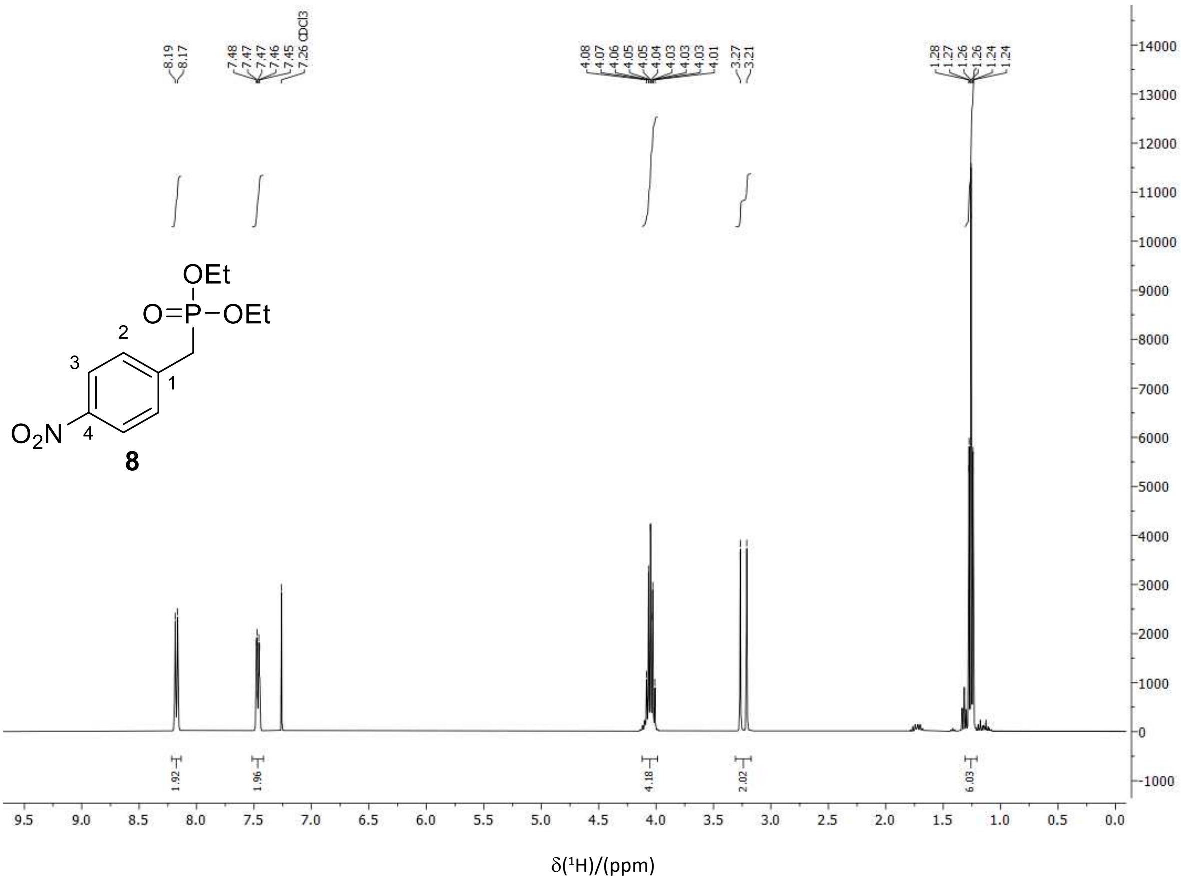

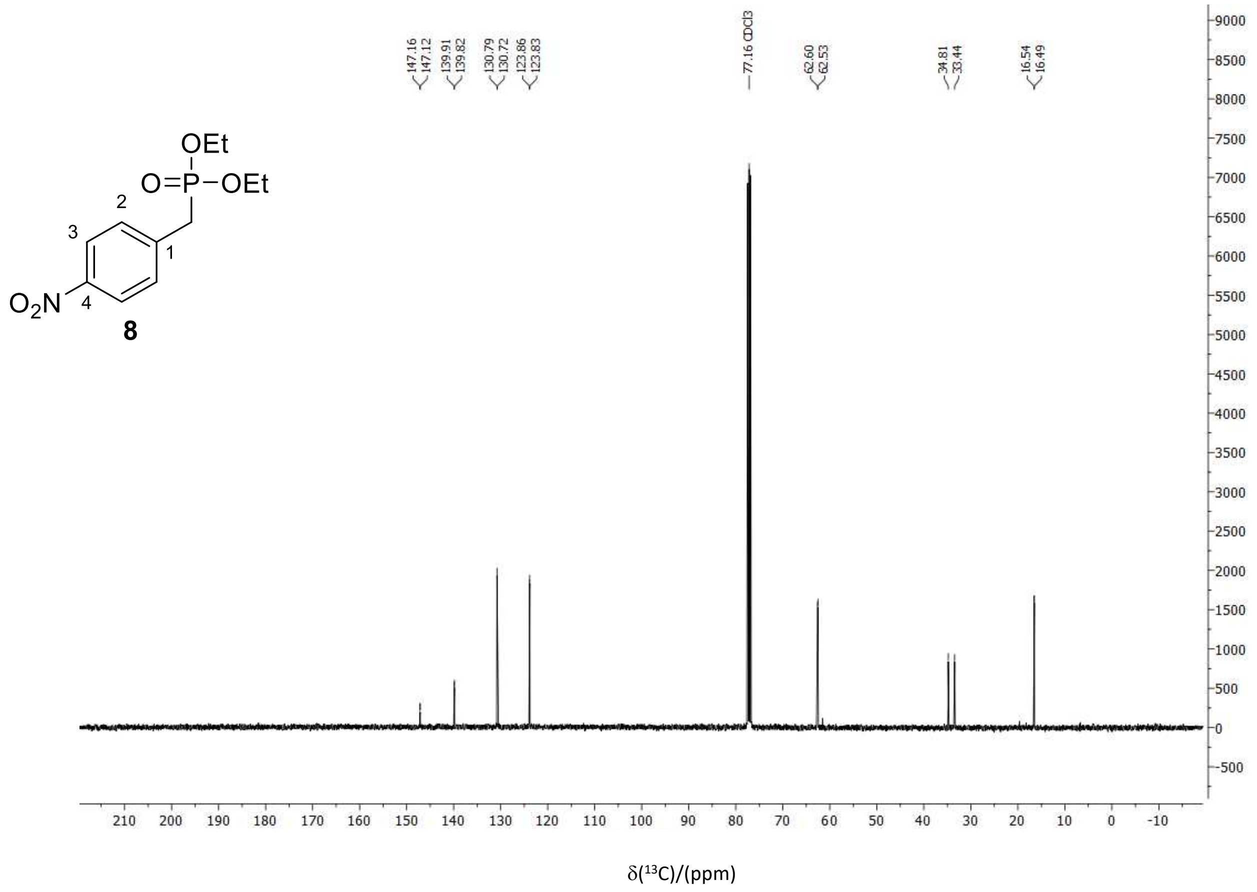

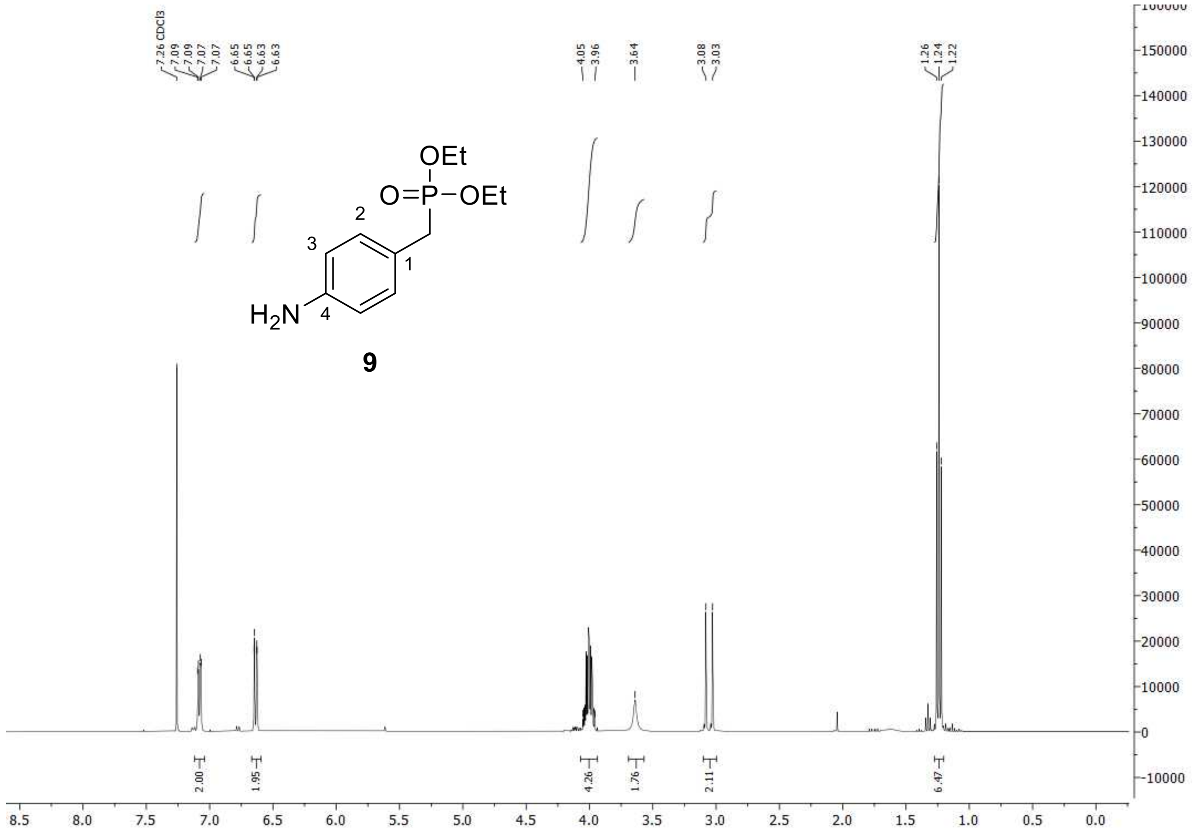

δ(¹H)/(ppm)

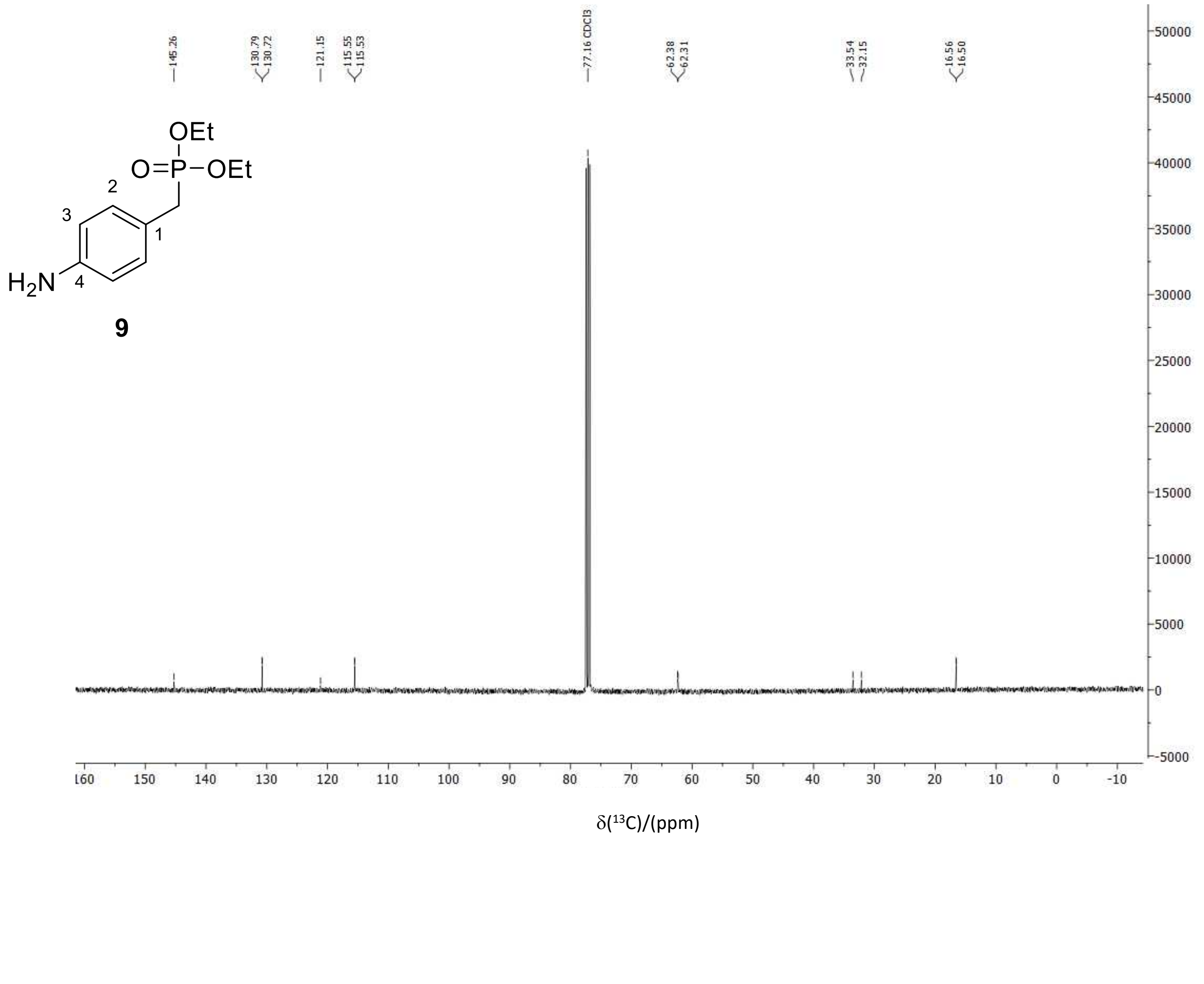

δ(¹³C)/(ppm)

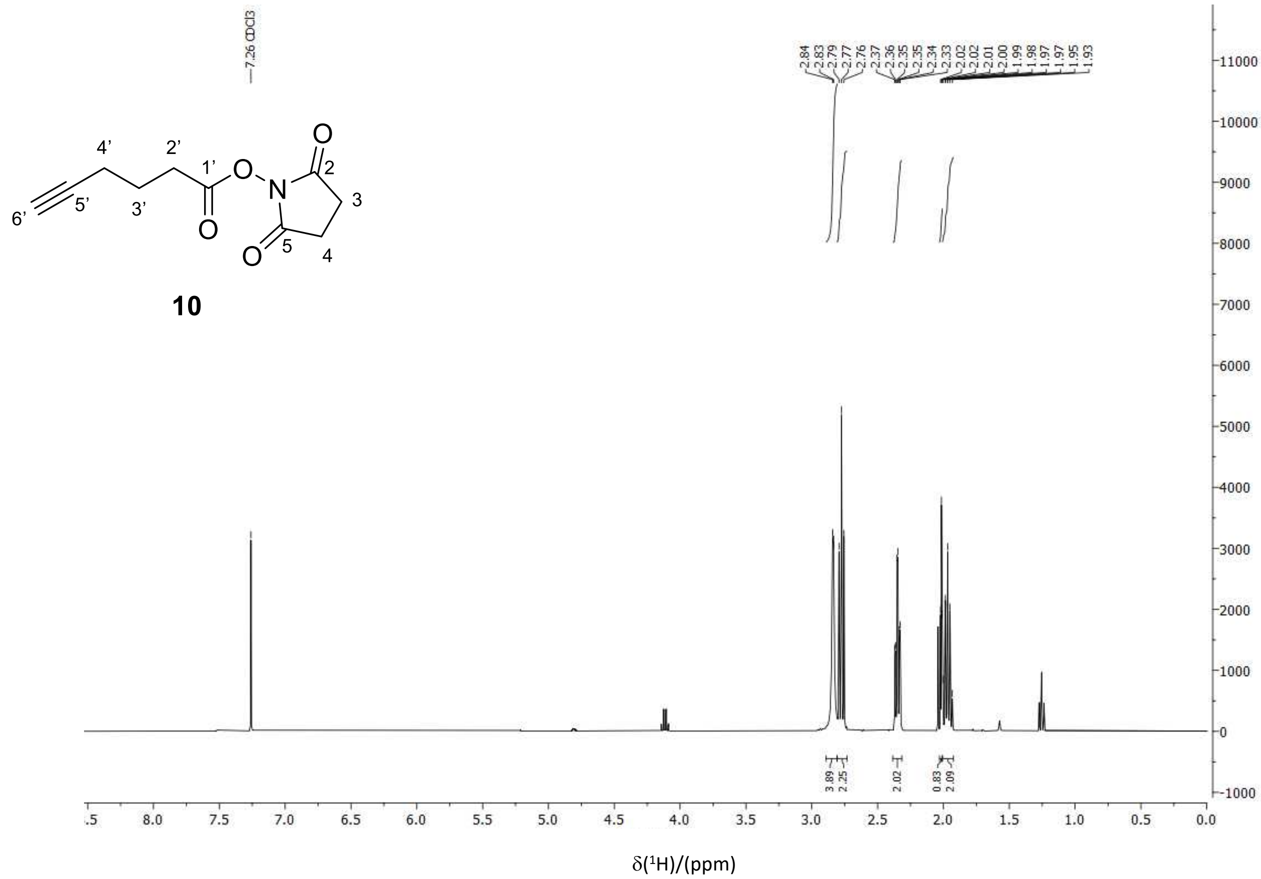
4'
2'
1'
O
N
2
3
6'
5'
3'
O
O
5
4
7.26 CDCl3
2.84
2.83
2.79
2.77
2.76
2.37
2.36
2.35
2.35
2.34
2.33
2.02
2.02
2.01
2.00
1.99
1.98
1.97
1.97
1.95
1.93
3.89
2.25
2.02
0.83
2.09
δ(1H)/(ppm)

10

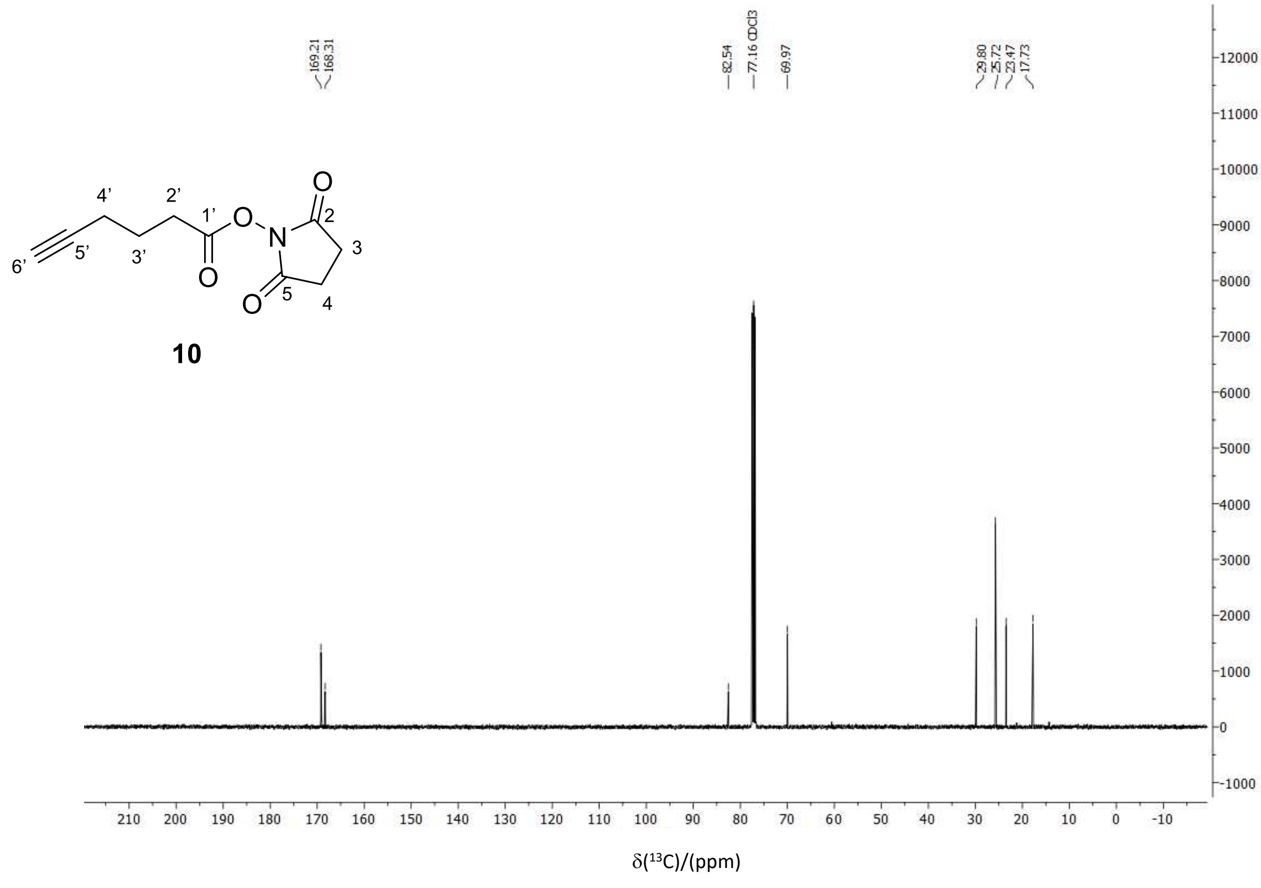
4'
2'
1'
O
N
2
3
6'
5'
3'
O
O
5
4
169.21
168.31
82.54
77.16 CDCl3
69.97
29.80
25.72
23.47
17.73
δ(13C)/(ppm)

10

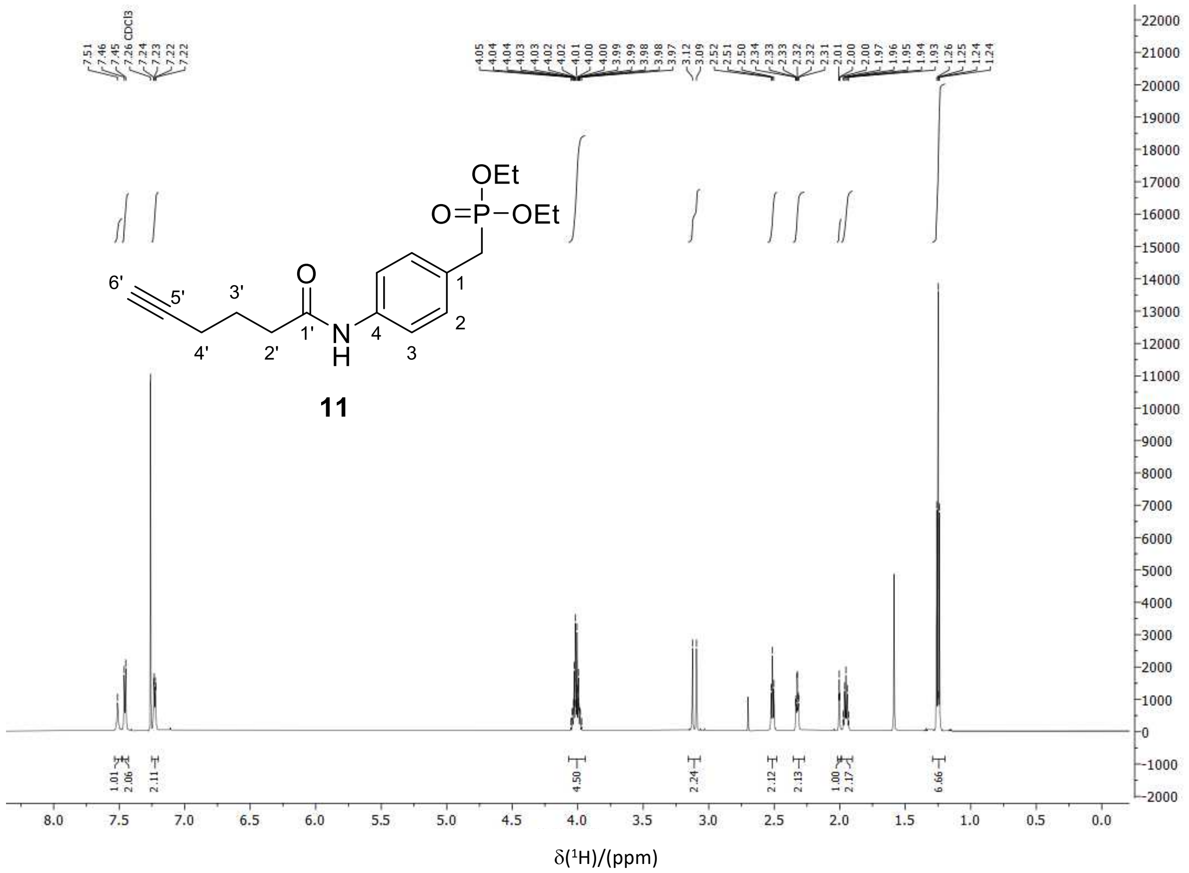

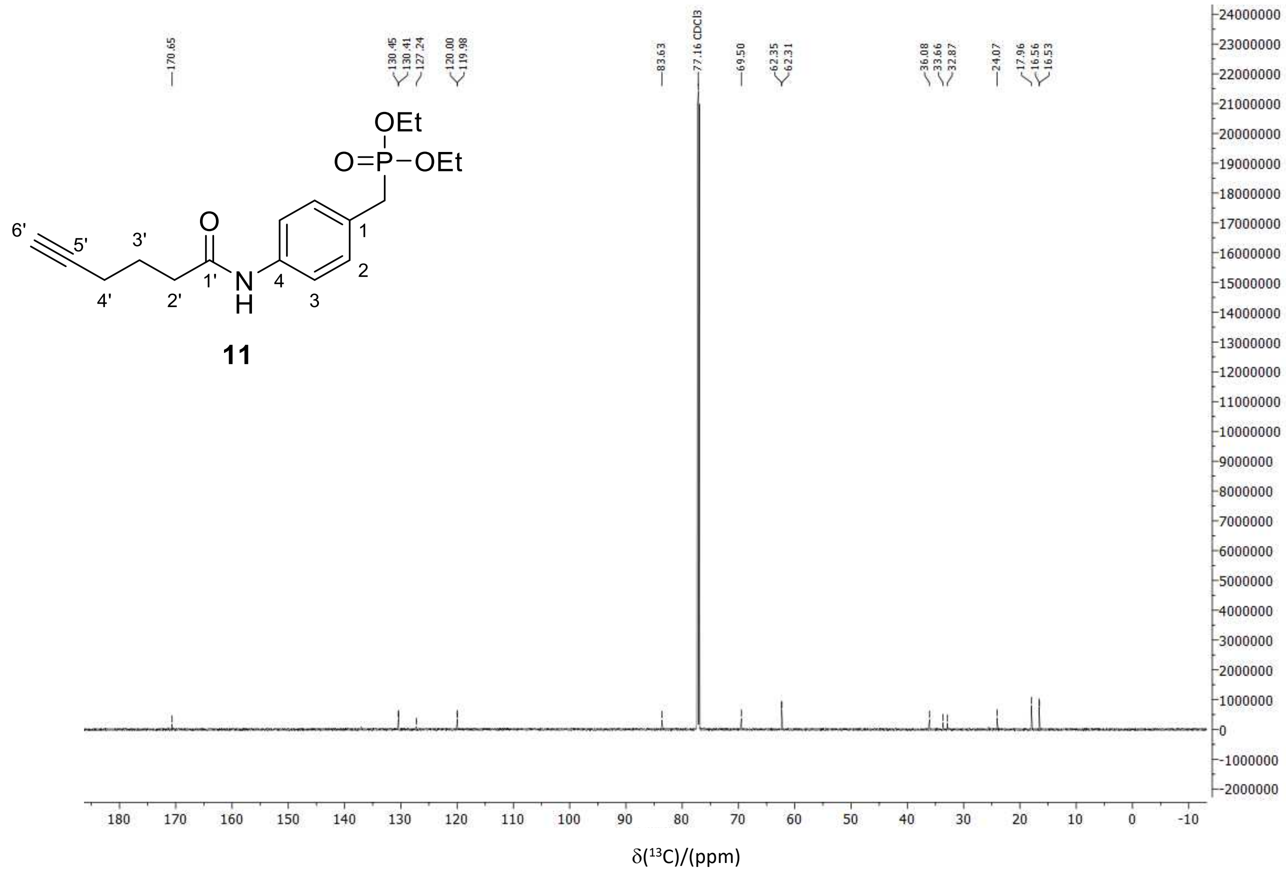

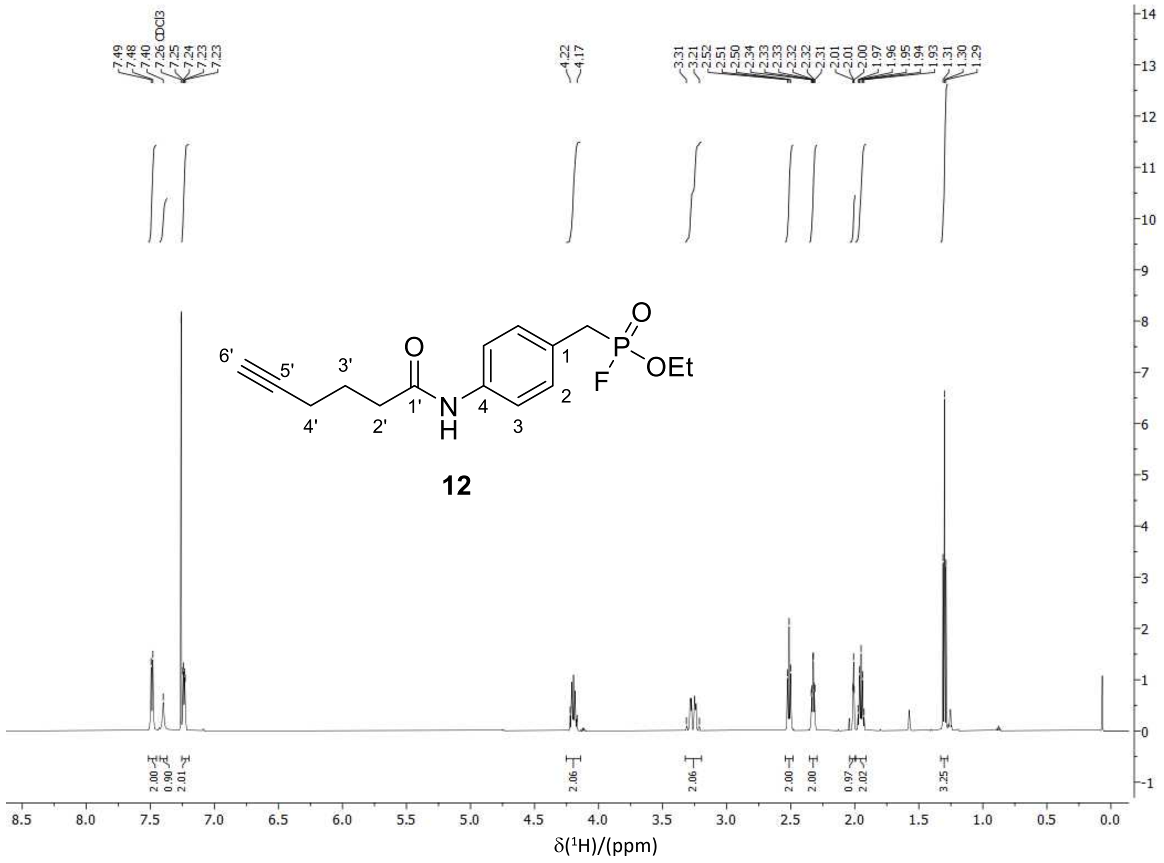
7.49 7.48 7.40 7.26 CDCl3 7.25 7.24 7.23 7.23
4.22 4.17
3.31 3.21 2.52 2.51 2.50 2.34 2.33 2.33 2.32 2.32 2.32 2.31 2.01 2.01 2.00 1.97 1.96 1.95 1.94 1.93 1.31 1.30 1.29
6'
5'
3'
O
1'
N
H
4
4'
2'
3
2
1
P
O
OEt
F
12
2.00 0.90 2.01
2.06
2.06
2.00 2.00 0.97 2.02
3.25
δ(¹H)/(ppm)

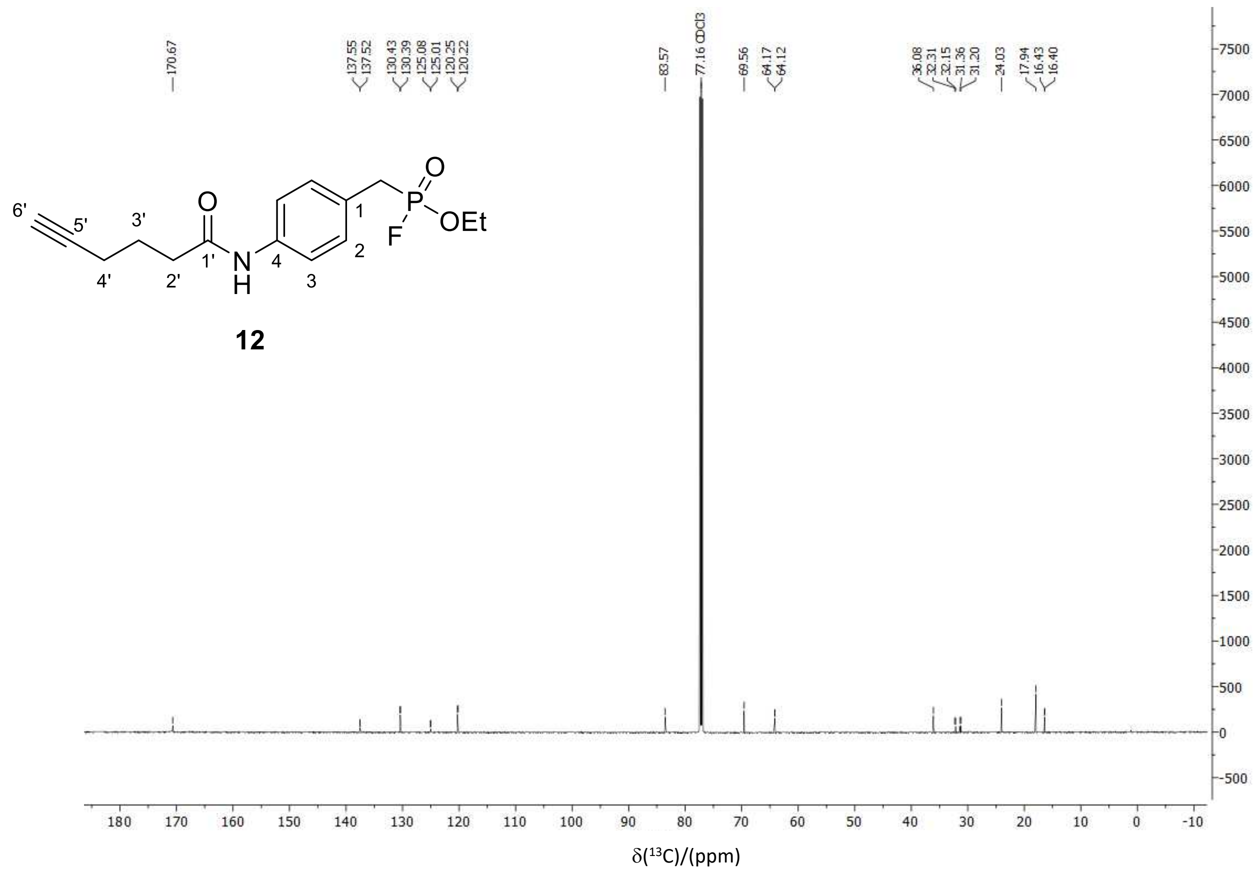
170.67
137.55 137.52 130.43 130.39 125.08 125.01 120.25 120.22
83.57
77.16 CDCl3
69.56 64.17 64.12
36.08 32.31 32.15 31.36 31.20 24.03 17.94 16.43 16.40
6'
5'
3'
O
1'
N
H
4
4'
2'
3
2
1
P
O
OEt
F
12
δ(¹³C)/(ppm)

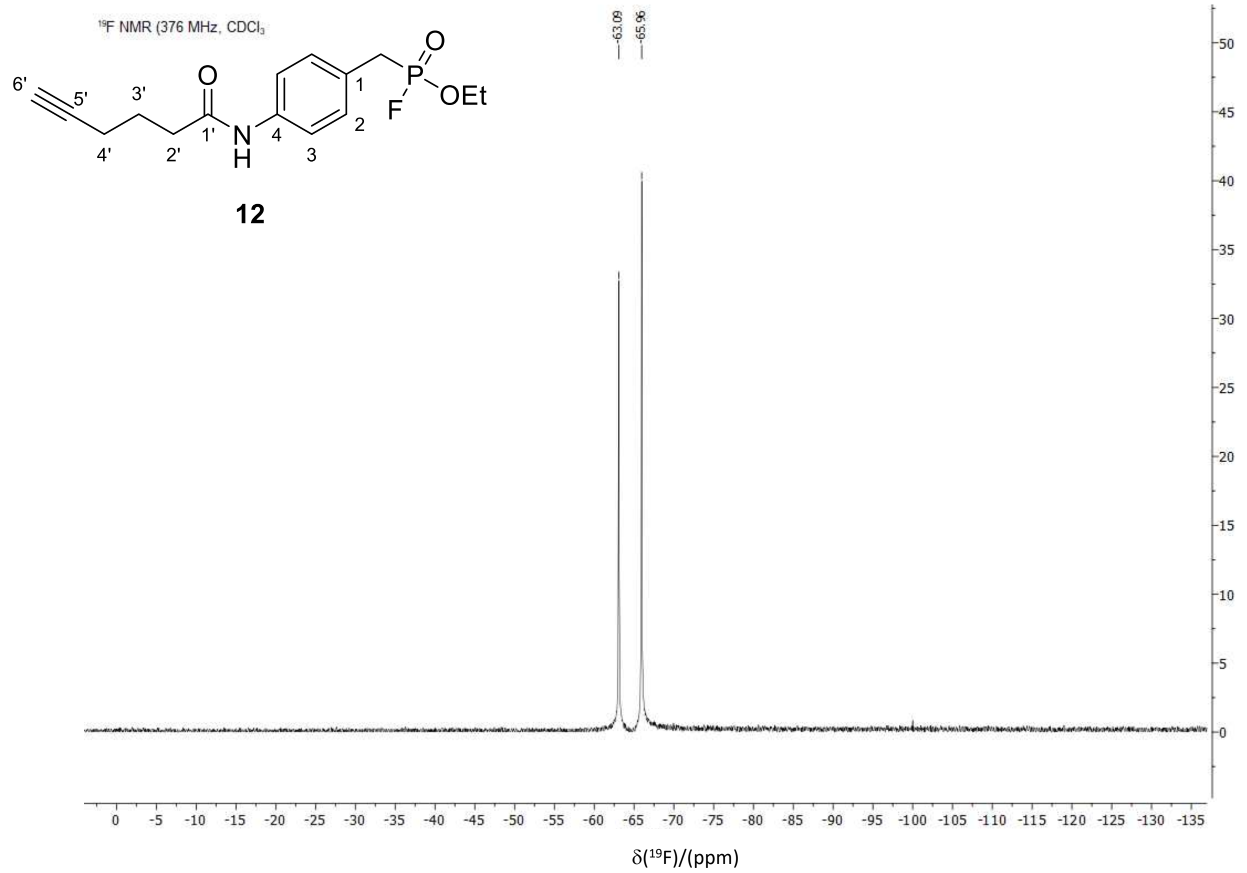
19F NMR (376 MHz, CDCl3
12
-63.09
-65.96
δ(19F)/(ppm)

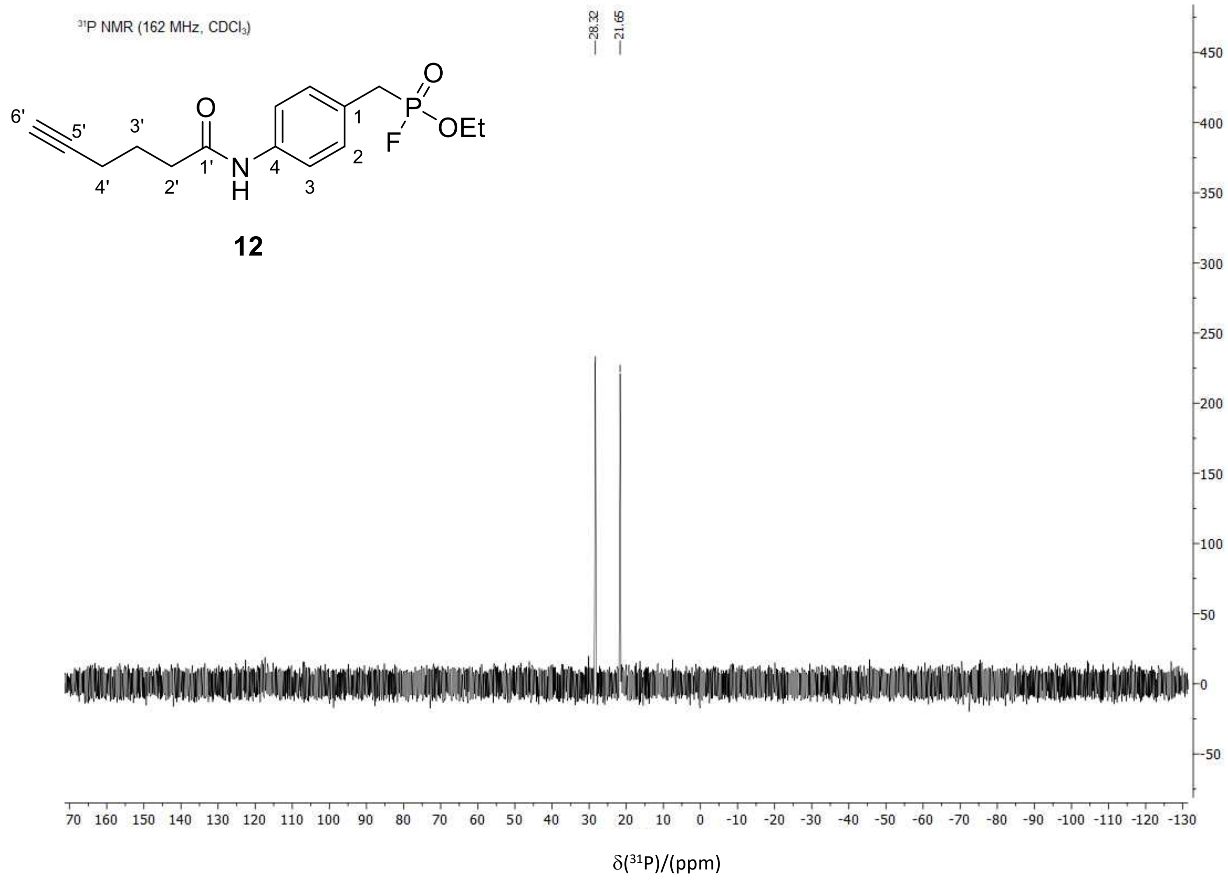
31P NMR (162 MHz, CDCl3)
12
28.32
21.65
δ(31P)/(ppm)

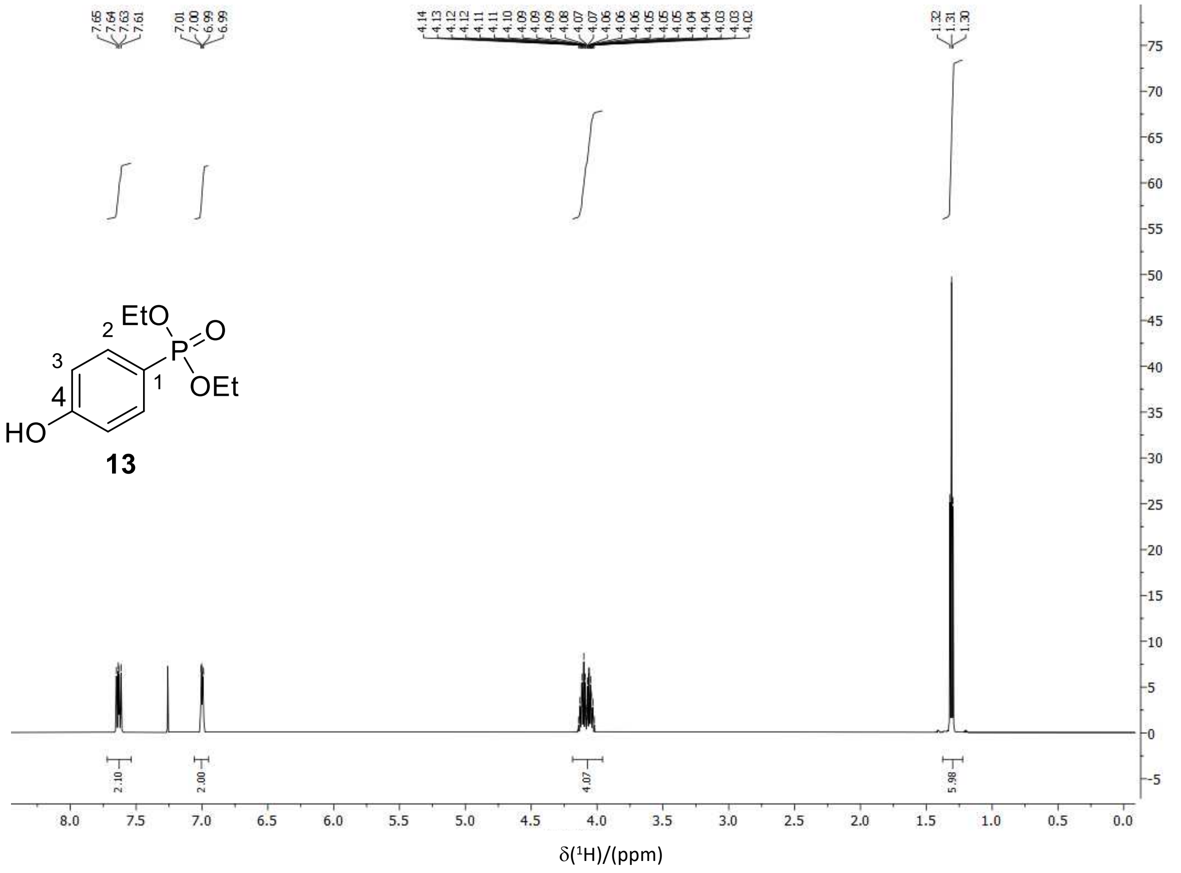
7.65 7.64 7.63 7.61
7.01 7.00 6.99 6.99
4.14 4.13 4.12 4.12 4.11 4.10 4.09 4.09 4.09 4.08 4.07 4.07 4.06 4.06 4.05 4.05 4.05 4.04 4.04 4.03 4.03 4.02
1.32 1.31 1.30
EtO
P
O
OEt
HO
1
2
3
4
13
2.10
2.00
4.07
5.98
δ(¹H)/(ppm)

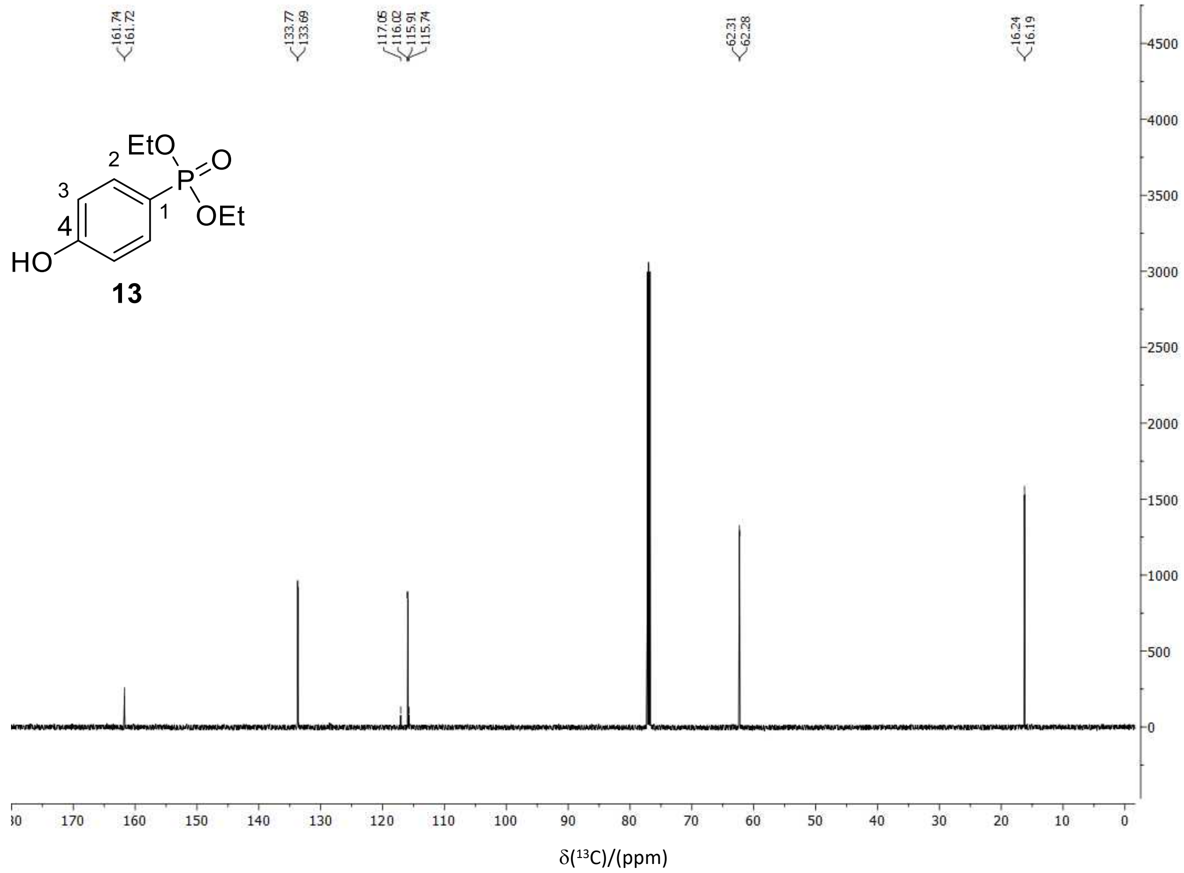
161.74 161.72
133.77 133.69
117.05 116.02 115.91 115.74
62.31 62.28
16.24 16.19
EtO
P
O
OEt
HO
1
2
3
4
13
δ(¹³C)/(ppm)

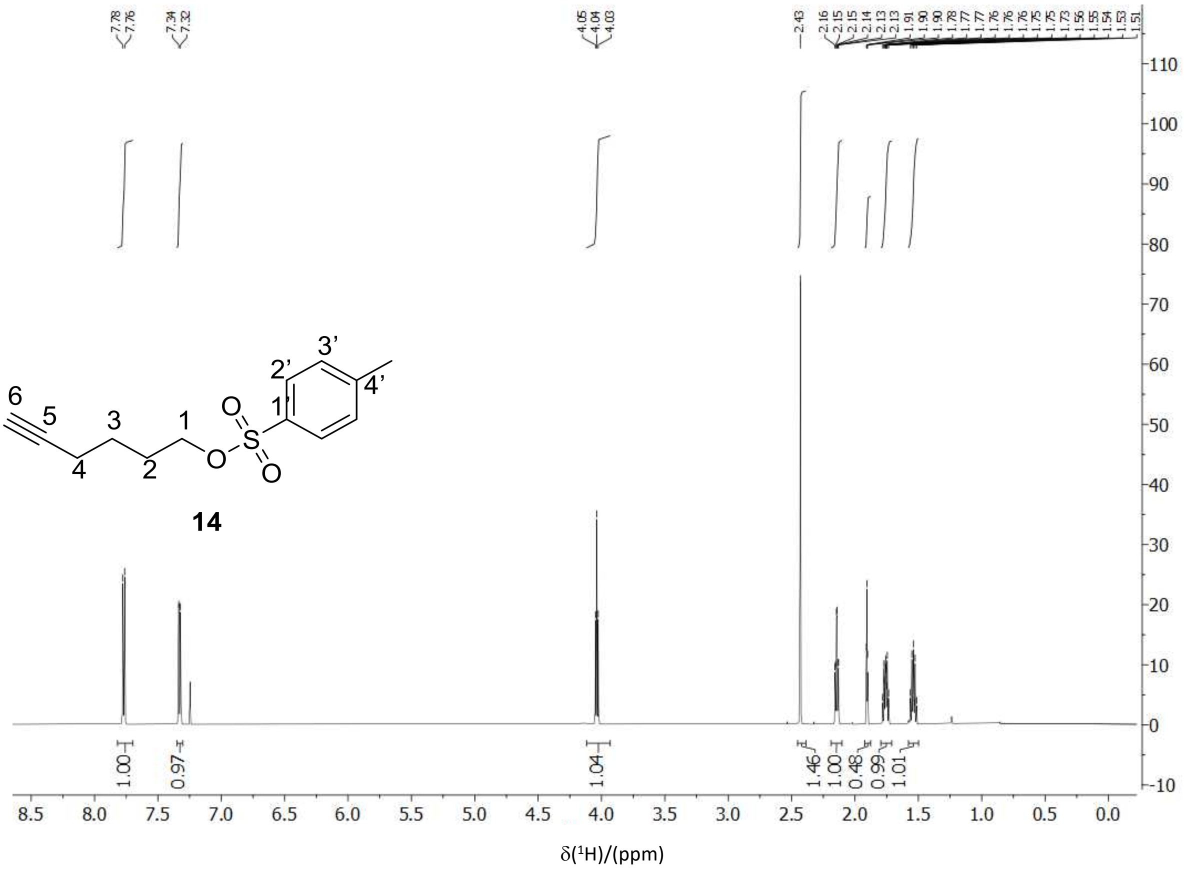
7.78
7.76
7.34
7.32
4.05
4.04
4.03
2.43
2.16
2.15
2.15
2.14
2.13
2.13
1.91
1.90
1.90
1.78
1.77
1.77
1.76
1.76
1.75
1.75
1.73
1.56
1.55
1.54
1.53
1.51
6
5
3
1
2'
3'
4'
1'
4
2
14
1.00
0.97
1.04
1.46
1.00
0.48
0.99
1.01
δ(1H)/(ppm)

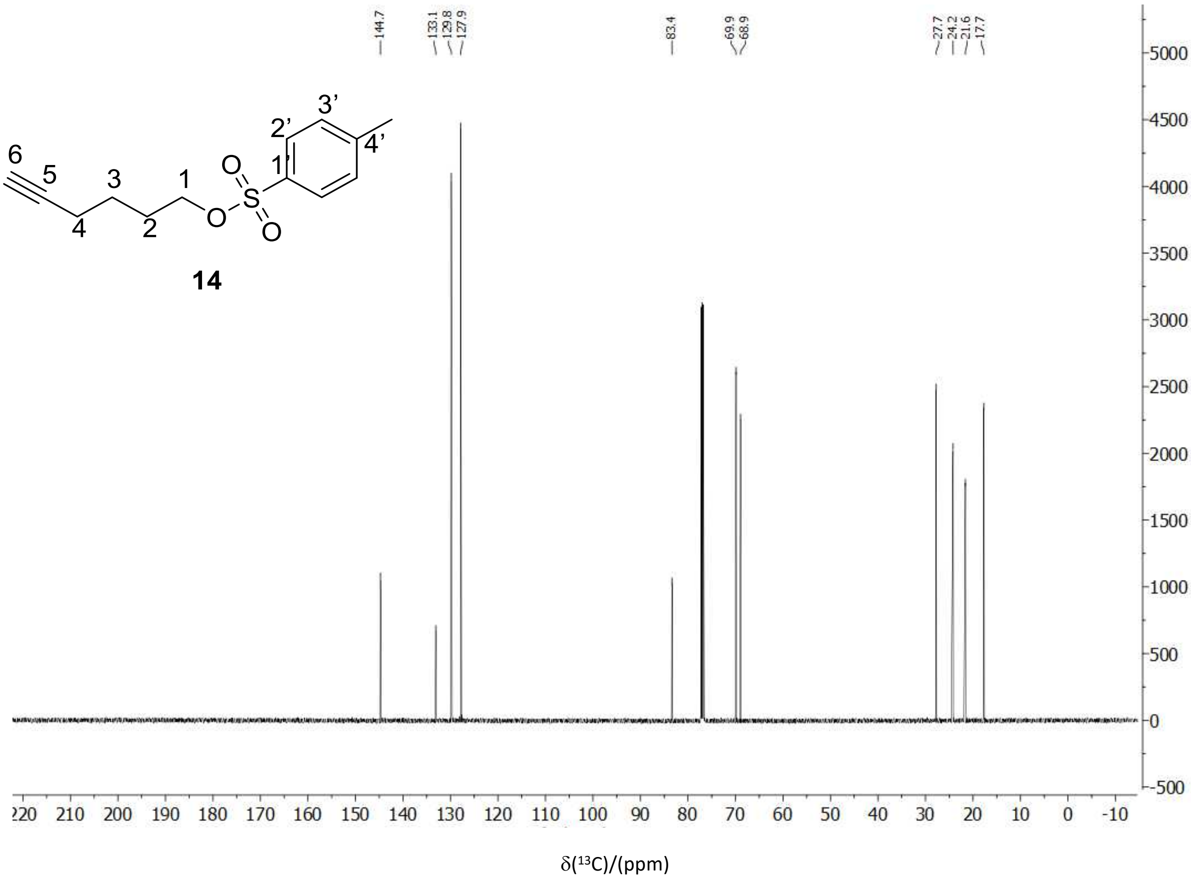
144.7
133.1
129.8
127.9
83.4
69.9
68.9
27.7
24.2
21.6
17.7
6
5
3
1
2'
3'
4'
1'
4
2
14
δ(13C)/(ppm)

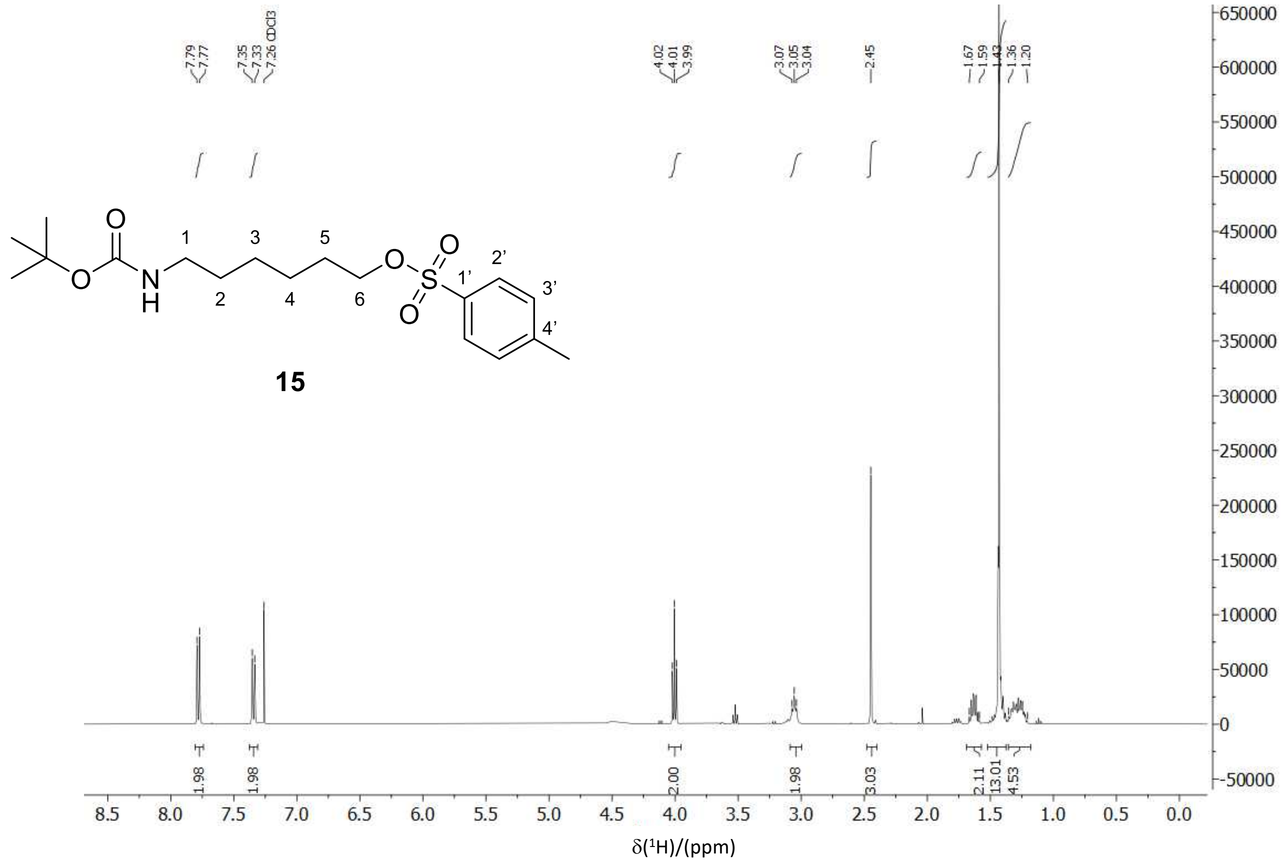

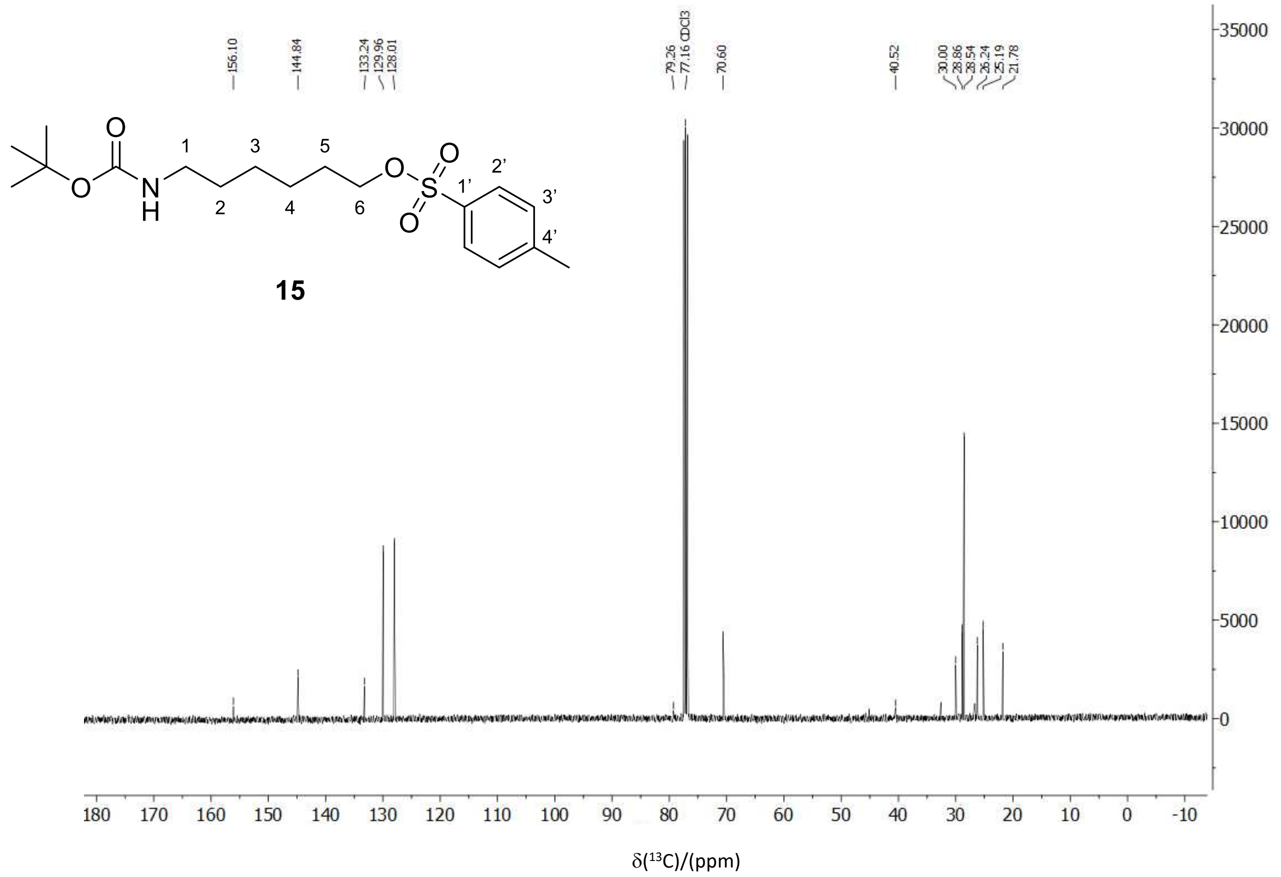

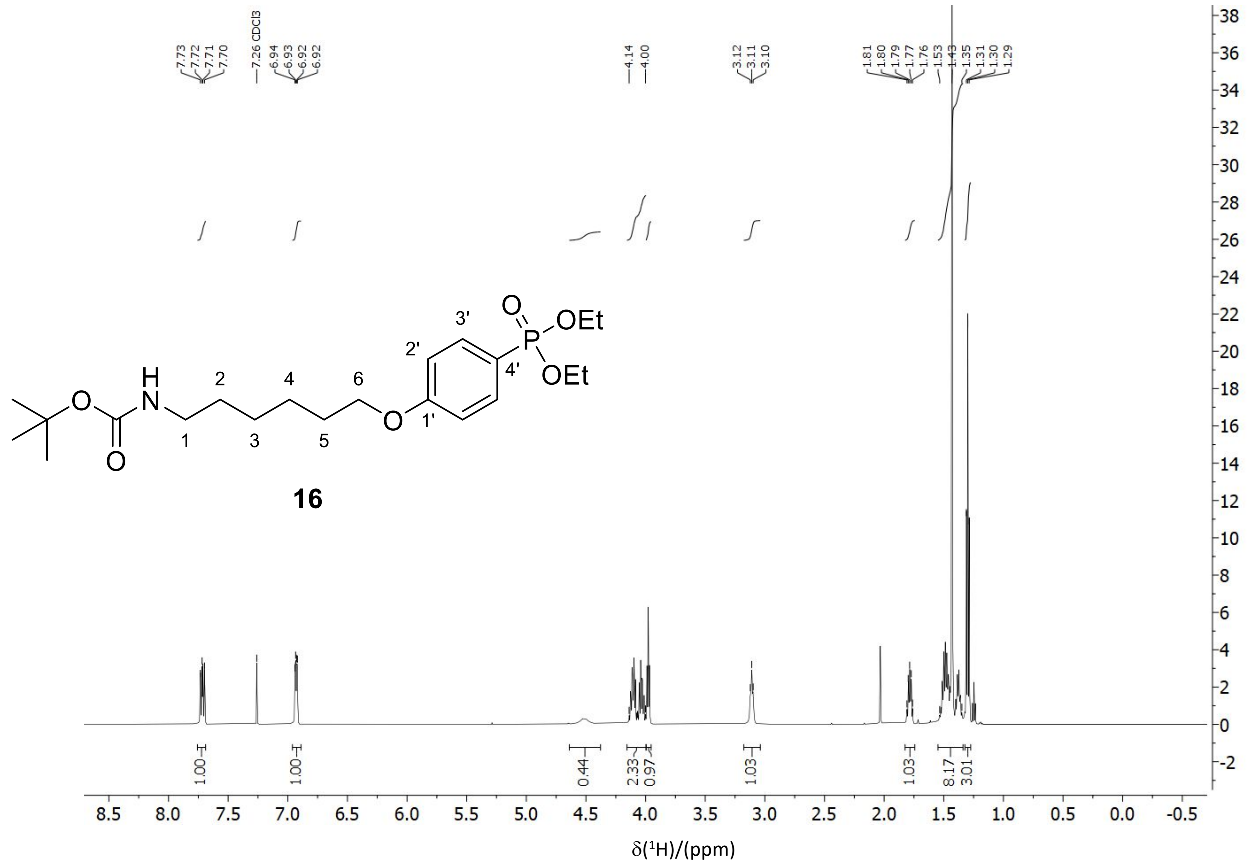
7.73
7.72
7.71
7.70
7.26 CDCl3
6.94
6.93
6.92
6.92
4.14
4.00
3.12
3.11
3.10
1.81
1.80
1.79
1.77
1.76
1.53
1.43
1.35
1.31
1.30
1.29
1.00
1.00
0.44
2.33
0.97
1.03
1.03
8.17
3.01
3'
2'
4'
1'
OEt
OEt
H
N
O
1
2
3
4
5
6
16
δ(¹H)/(ppm)

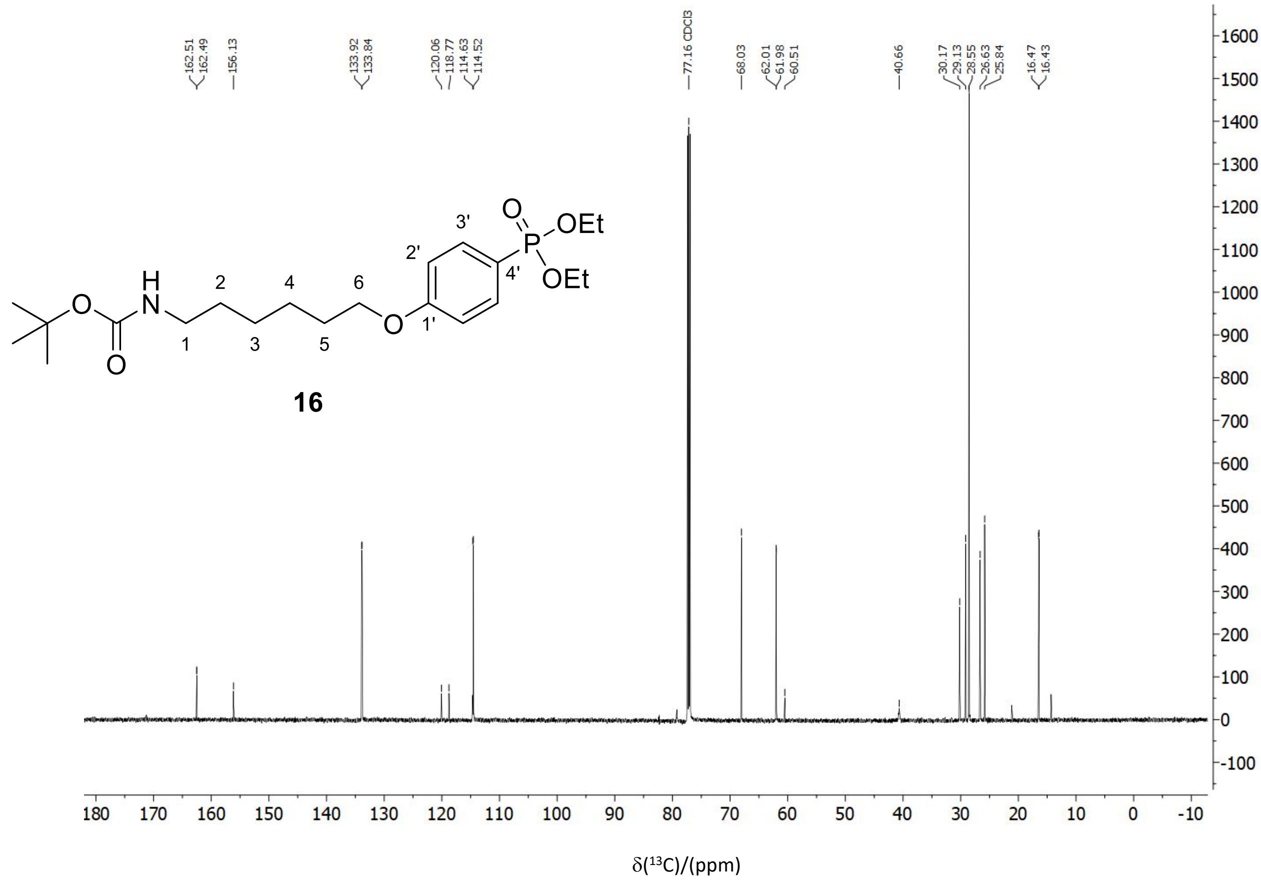
162.51
162.49
156.13
133.92
133.84
120.06
118.77
114.63
114.52
77.16 CDCl3
68.03
62.01
61.98
60.51
40.66
30.17
29.13
28.55
26.63
25.84
16.47
16.43
3'
2'
4'
1'
OEt
OEt
H
N
O
1
2
3
4
5
6
16
δ(¹³C)/(ppm)

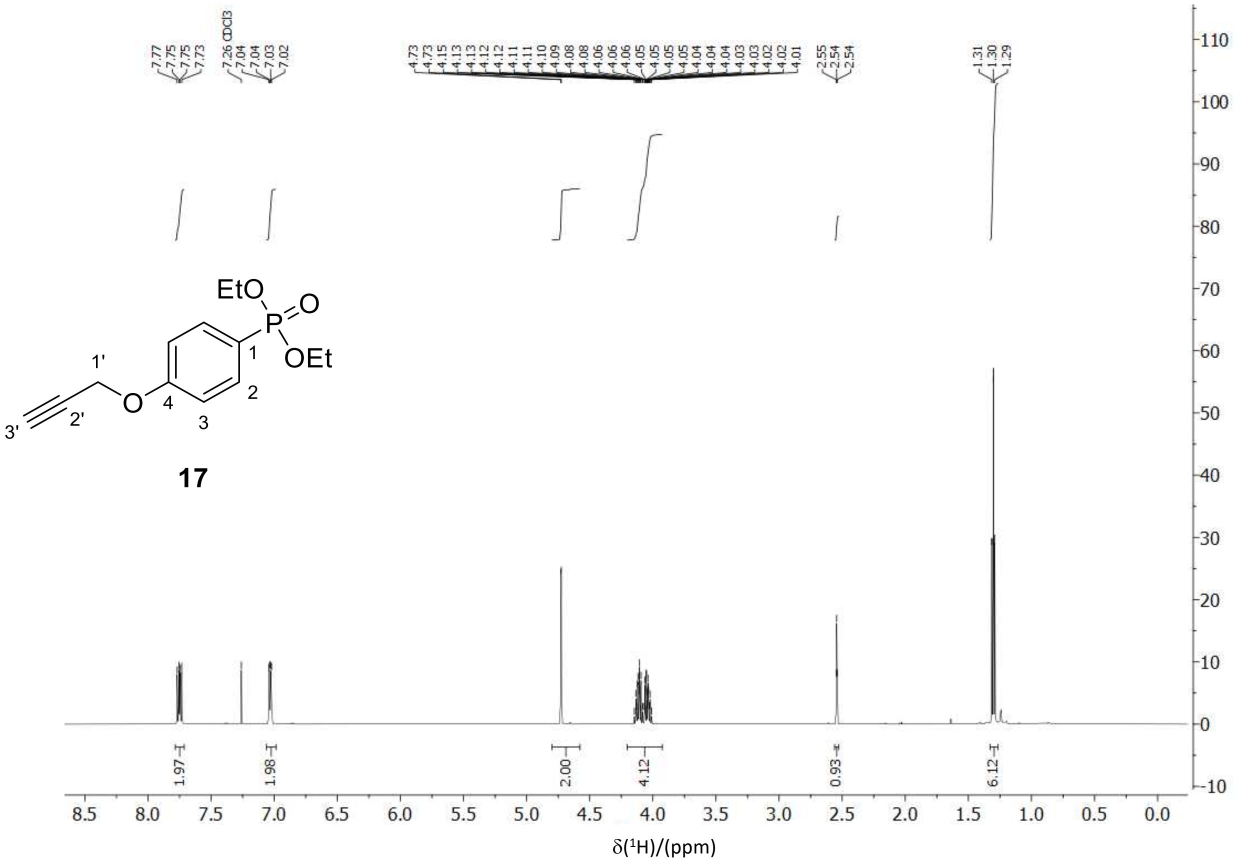
7.77 7.75 7.75 7.73
7.26 CDCl3 7.04 7.04 7.03 7.02
4.73 4.73 4.15 4.13 4.13 4.12 4.12 4.11 4.11 4.10 4.09 4.08 4.08 4.06 4.06 4.06 4.05 4.05 4.05 4.05 4.04 4.04 4.04 4.03 4.03 4.02 4.02 4.01
2.55 2.54 2.54
1.31 1.30 1.29
EtO
P
O
OEt
1'
1
2
3
4
O
2'
3'
17
1.97
1.98
2.00
4.12
0.93
6.12
δ(¹H)/(ppm)

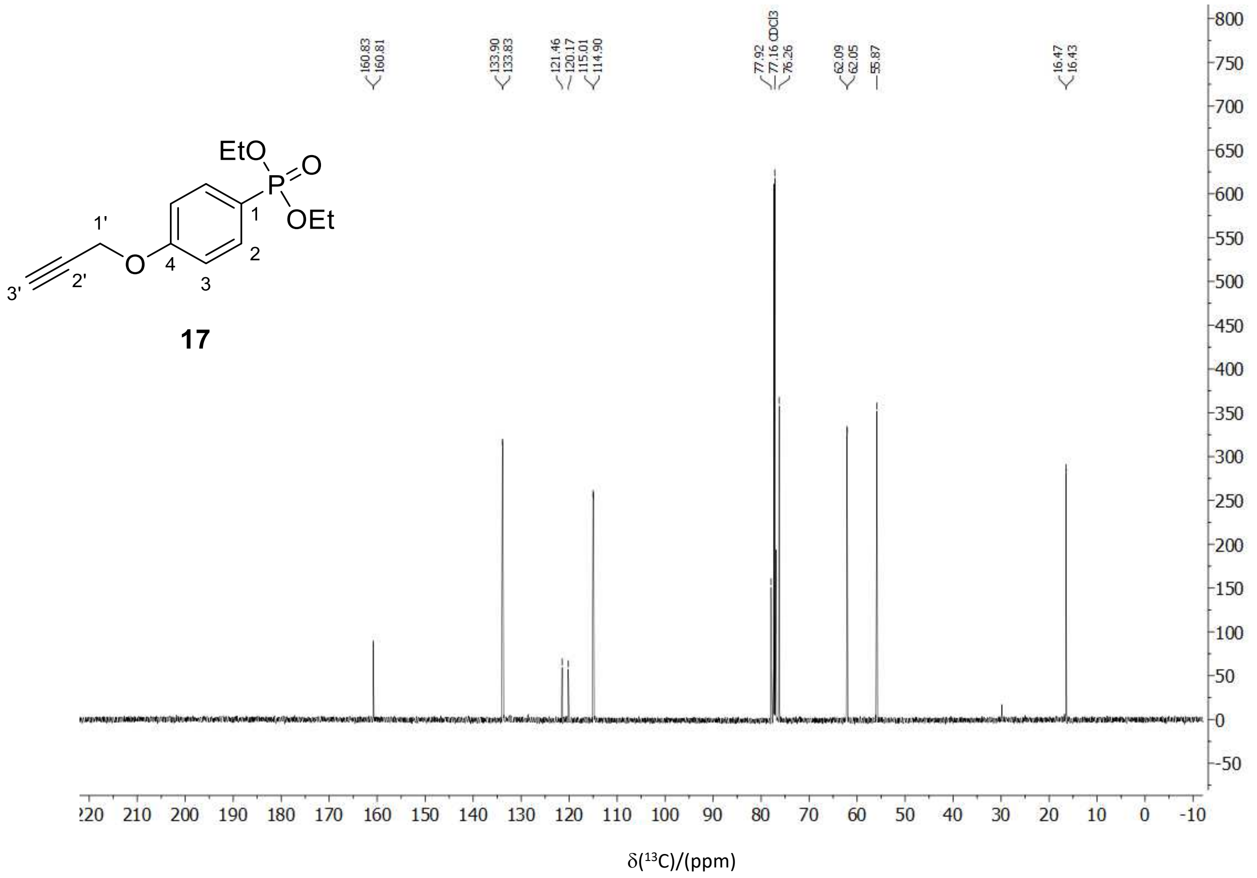
160.83 160.81
133.90 133.83
121.46 120.17 115.01 114.90
77.92 77.16 CDCl3 76.26
62.09 62.05
55.87
16.47 16.43
EtO
P
O
OEt
1'
1
2
3
4
O
2'
3'
17
δ(¹³C)/(ppm)

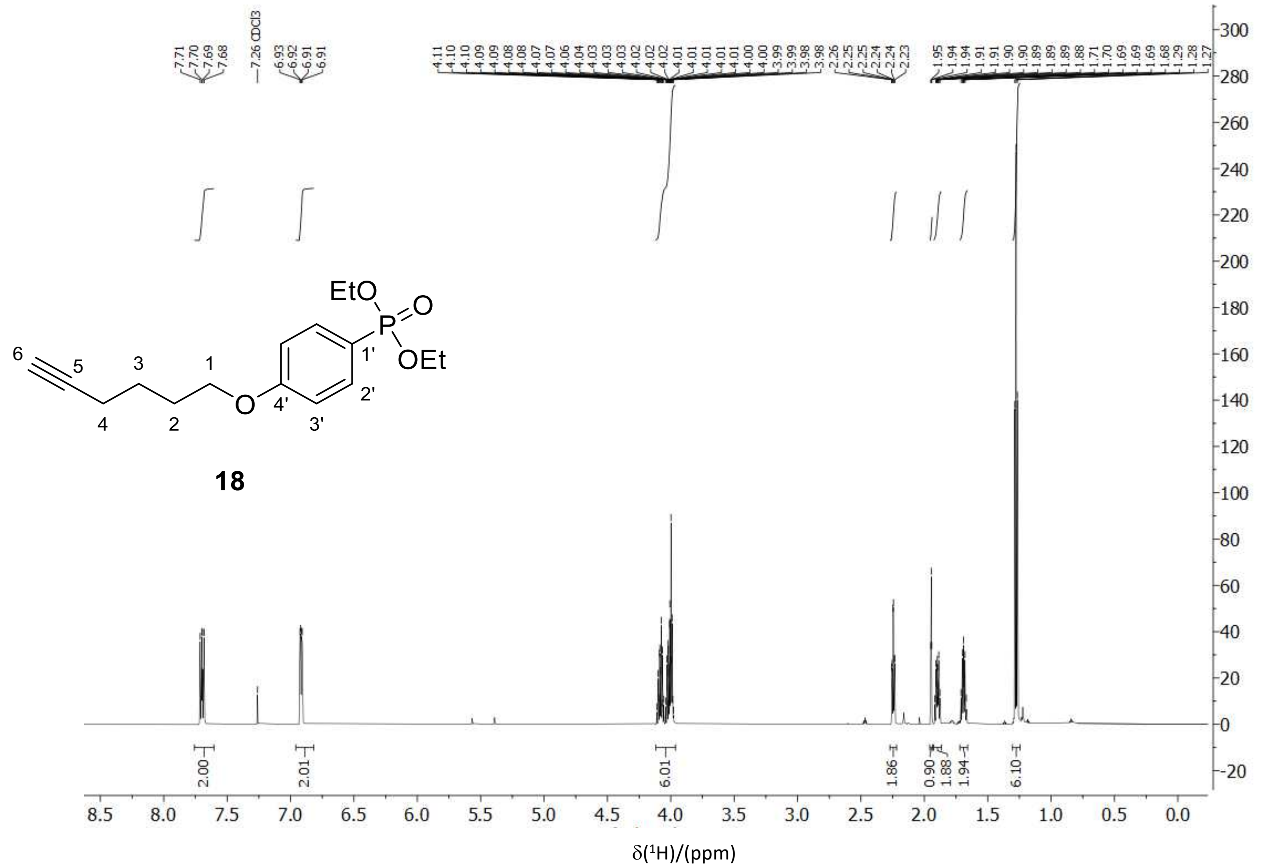
7.71
7.70
7.69
7.68
7.26 CDCl3
6.93
6.92
6.91
6.91
4.11
4.10
4.10
4.09
4.09
4.08
4.08
4.07
4.07
4.06
4.04
4.04
4.03
4.03
4.03
4.02
4.02
4.02
4.01
4.01
4.01
4.01
4.00
4.00
3.99
3.99
3.98
3.98
2.26
2.25
2.25
2.24
2.24
2.23
1.95
1.94
1.94
1.91
1.91
1.90
1.90
1.89
1.89
1.89
1.88
1.71
1.70
1.69
1.69
1.68
1.29
1.28
1.27
EtO
P
O
OEt
1'
2'
3'
4'
O
1
2
3
4
5
6
2.00
2.01
6.01
1.86
0.90
1.88
1.94
6.10
δ(1H)/(ppm)

18

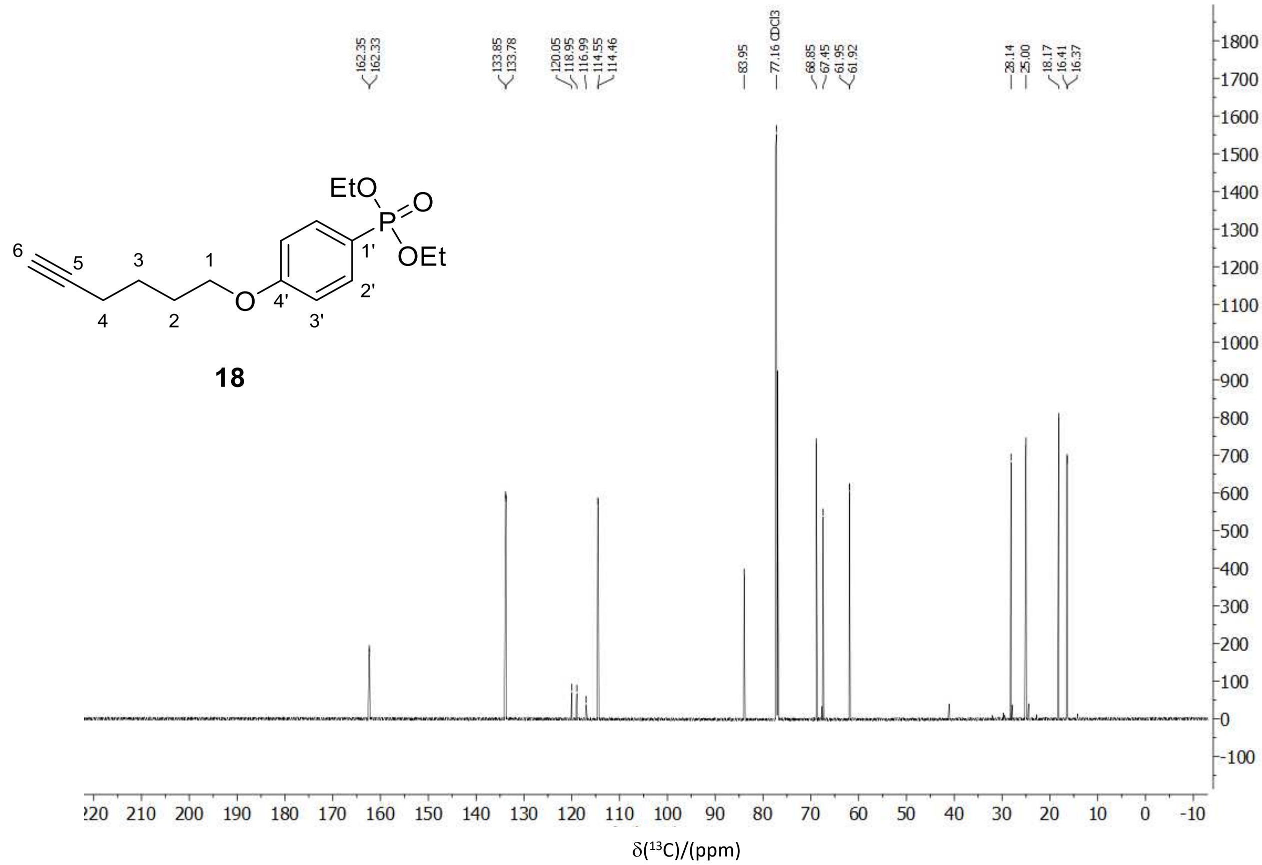
162.35
162.33
133.85
133.78
120.05
118.95
116.99
114.55
114.46
83.95
77.16 CDCl3
68.85
67.45
61.95
61.92
28.14
25.00
18.17
16.41
16.37
EtO
P
O
OEt
1'
2'
3'
4'
O
1
2
3
4
5
6
δ(13C)/(ppm)

18

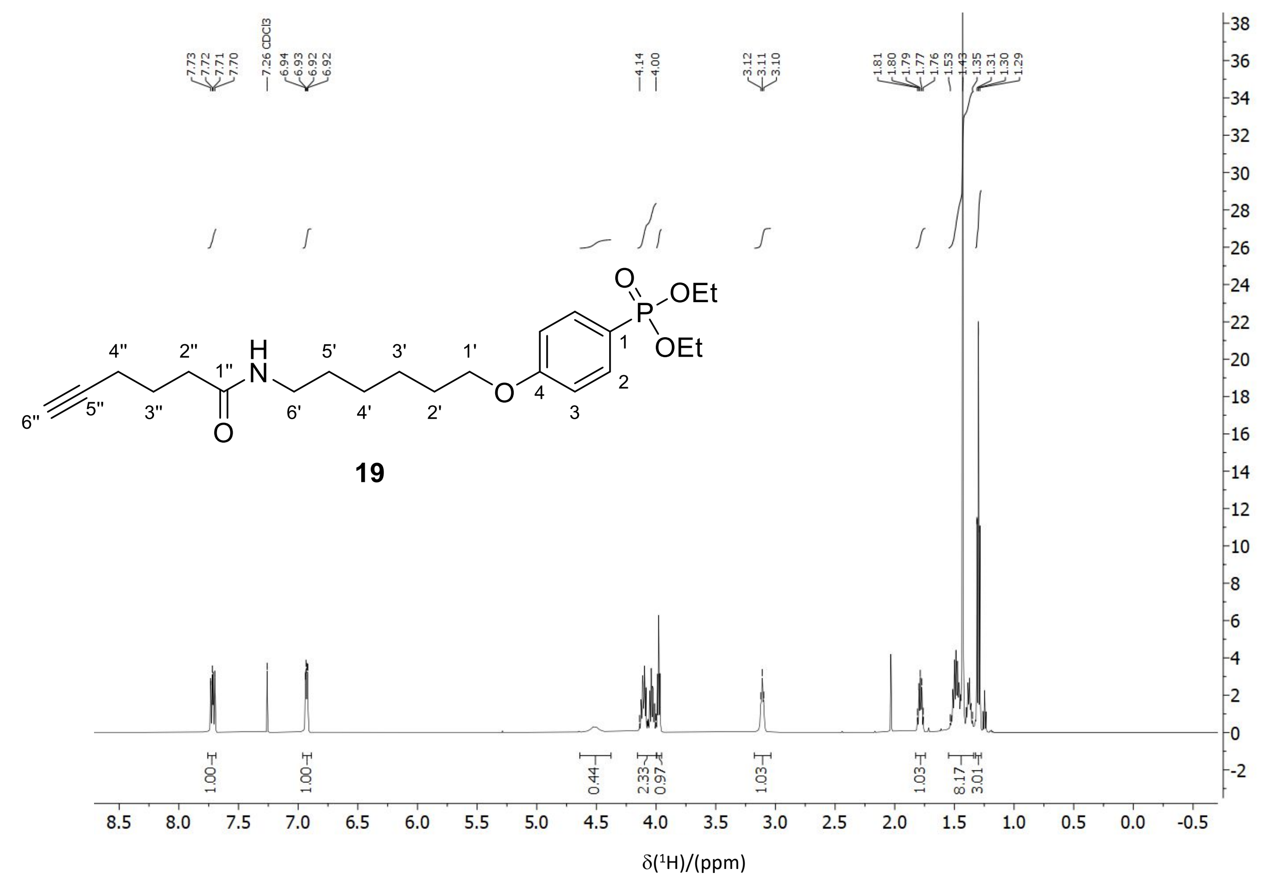
7.73 7.72 7.71 7.70
7.26 CDCl3
6.94 6.93 6.92 6.92
4.14 4.00
3.12 3.11 3.10
1.81 1.80 1.79 1.77 1.76 1.53 1.43 1.35 1.31 1.30 1.29
1.00 1.00 0.44 2.33 0.97 1.03 1.03 8.17 3.01
OEt
OEt
19
δ(¹H)/(ppm)

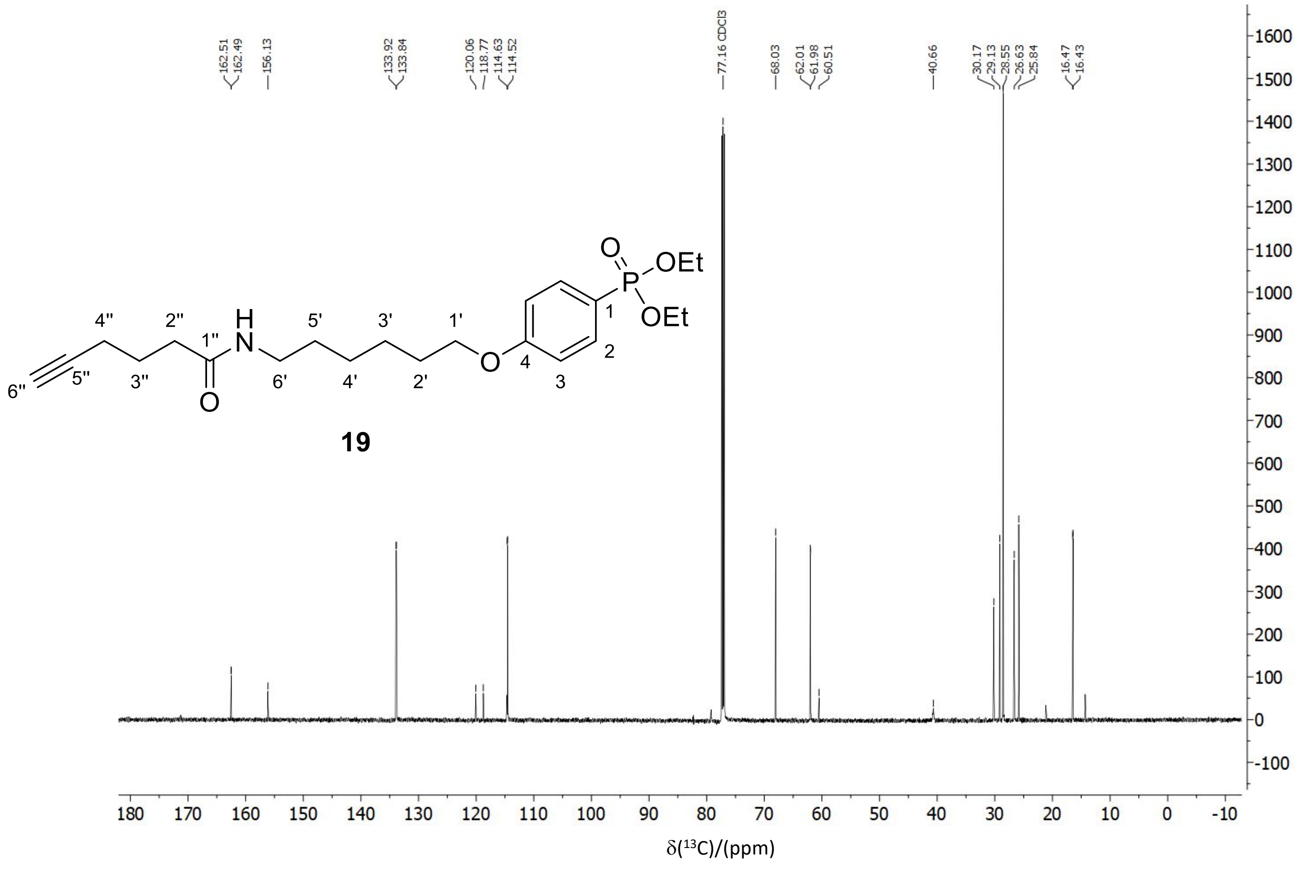
162.51 162.49 156.13 133.92 133.84 120.06 118.77 114.63 114.52
77.16 CDCl3
68.03 62.01 61.98 60.51 40.66 30.17 29.13 28.55 26.63 25.84 16.47 16.43
OEt
OEt
19
δ(¹³C)/(ppm)

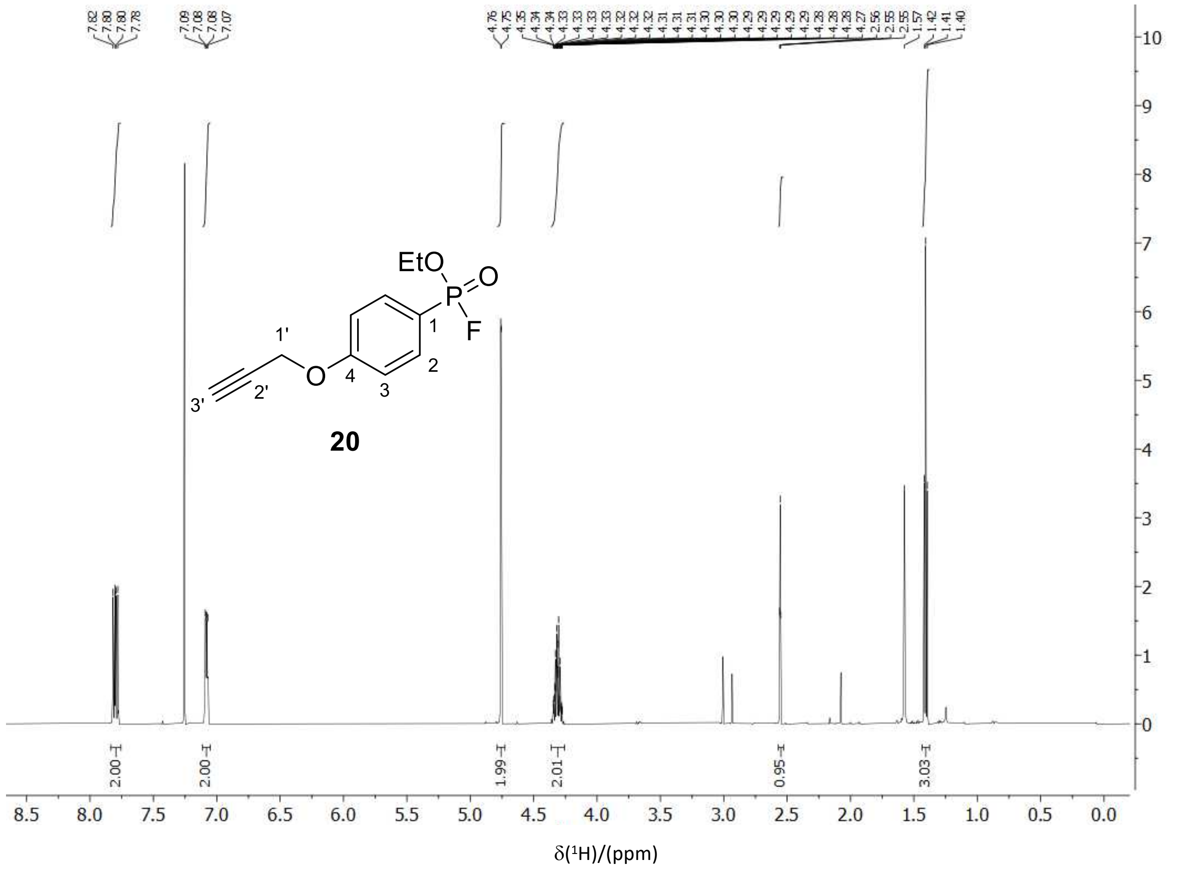
EtO
P
O
F
1'
1
2
3
4
O
2'
3'
20
δ(¹H)/(ppm)

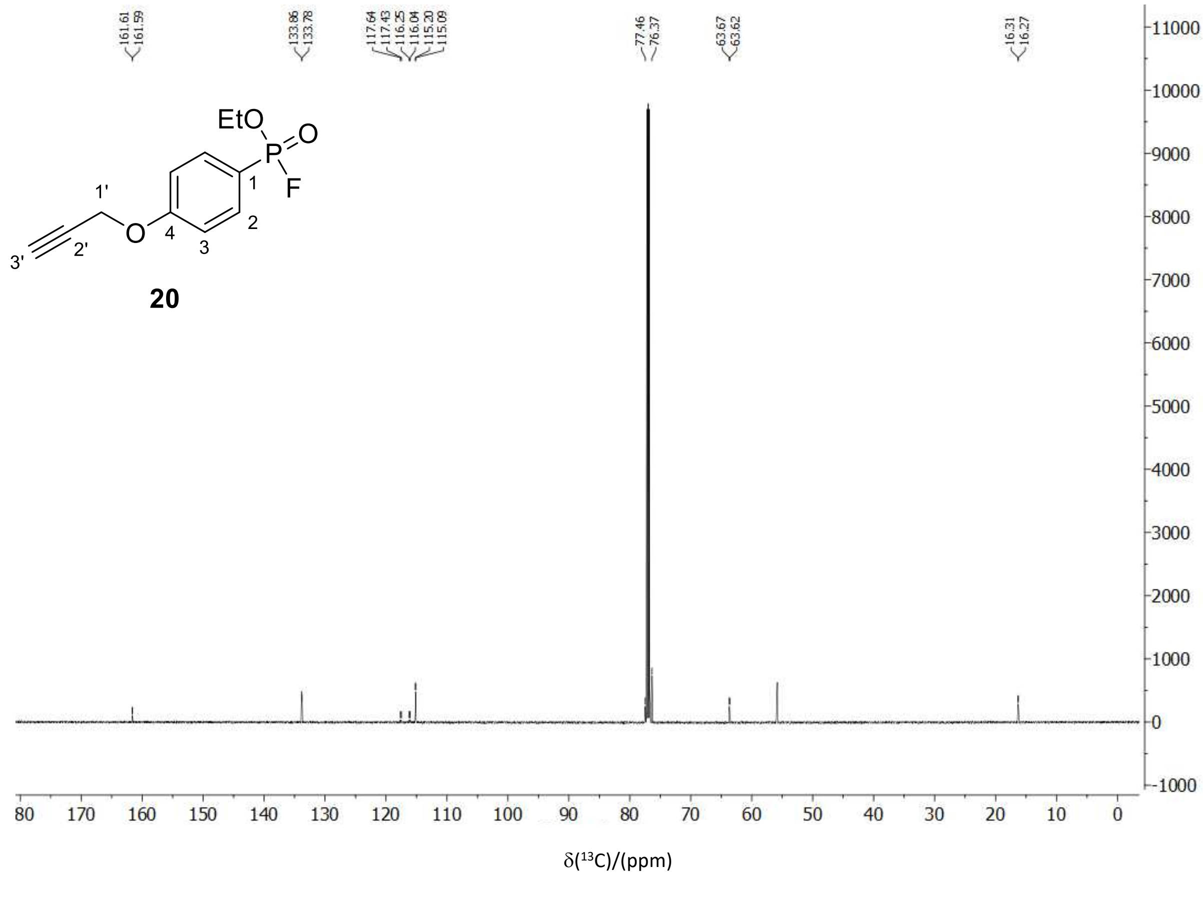
EtO
P
O
F
1'
1
2
3
4
O
2'
3'
20
δ(¹³C)/(ppm)

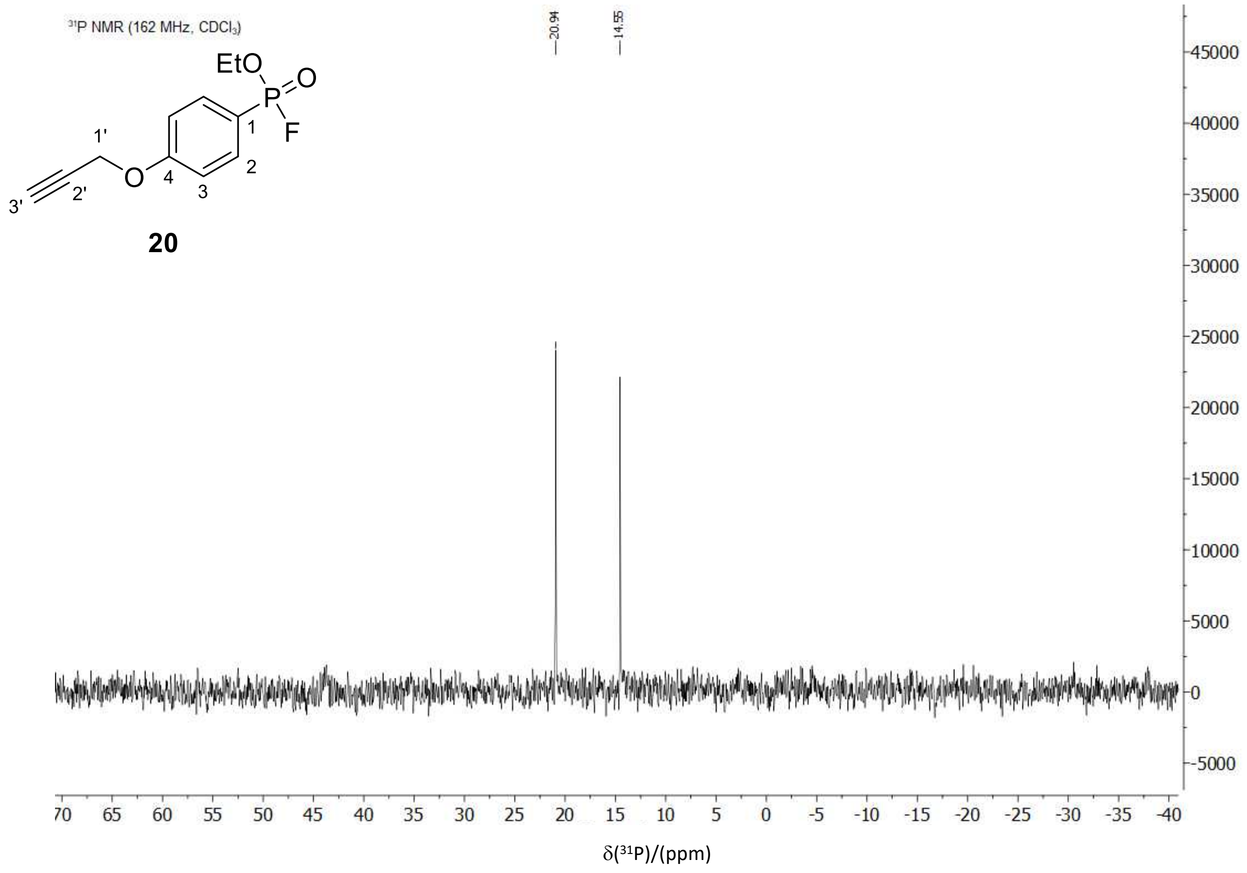

³¹P NMR (162 MHz, CDCl₃)
20.94
14.55
EtO
P
O
F
1
2
3
4
1'
2'
3'
O
20
45000
40000
35000
30000
25000
20000
15000
10000
5000
0
-5000
70 65 60 55 50 45 40 35 30 25 20 15 10 5 0 -5 -10 -15 -20 -25 -30 -35 -40
δ(³¹P)/(ppm)

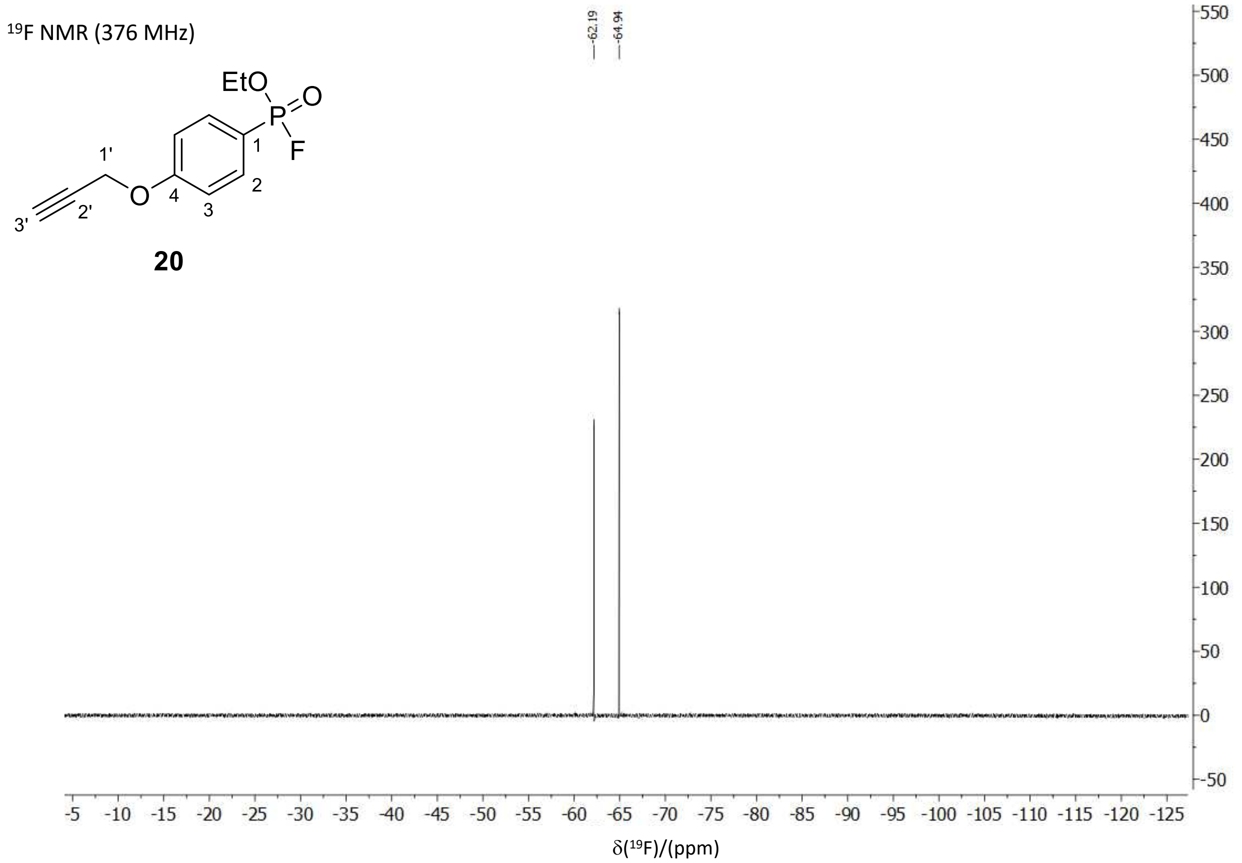

¹⁹F NMR (376 MHz)
-62.19
-64.94
EtO
P
O
F
1
2
3
4
1'
2'
3'
O
20
550
500
450
400
350
300
250
200
150
100
50
0
-50
-5 -10 -15 -20 -25 -30 -35 -40 -45 -50 -55 -60 -65 -70 -75 -80 -85 -90 -95 -100 -105 -110 -115 -120 -125
δ(¹⁹F)/(ppm)

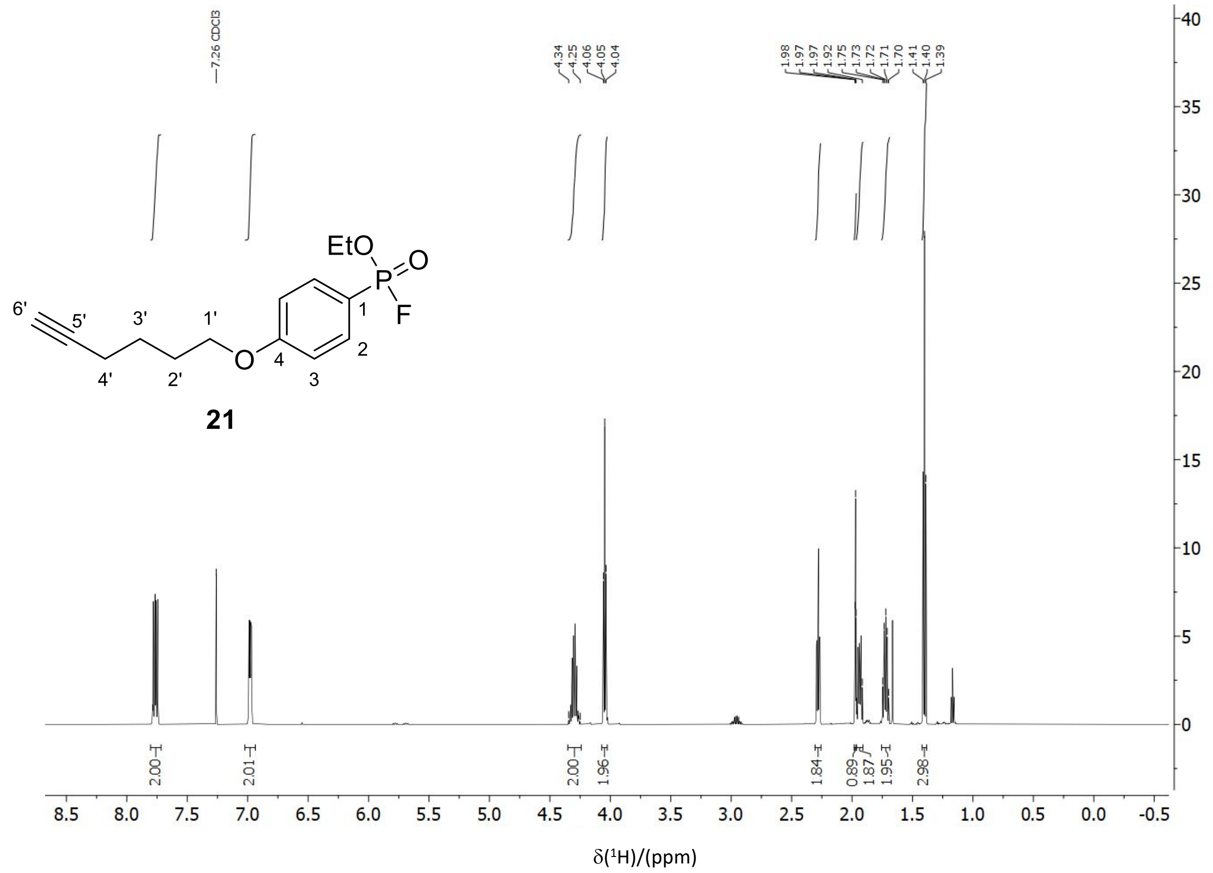
7.26 CDCl3
4.34
4.25
4.06
4.05
4.04
1.98
1.97
1.97
1.92
1.75
1.73
1.72
1.71
1.70
1.41
1.40
1.39
EtO
P
O
F
6'
5'
3'
1'
1
4
2
3
O
4'
2'
21
2.00
2.01
2.00
1.96
1.84
0.89
1.87
1.95
2.98
δ(¹H)/(ppm)

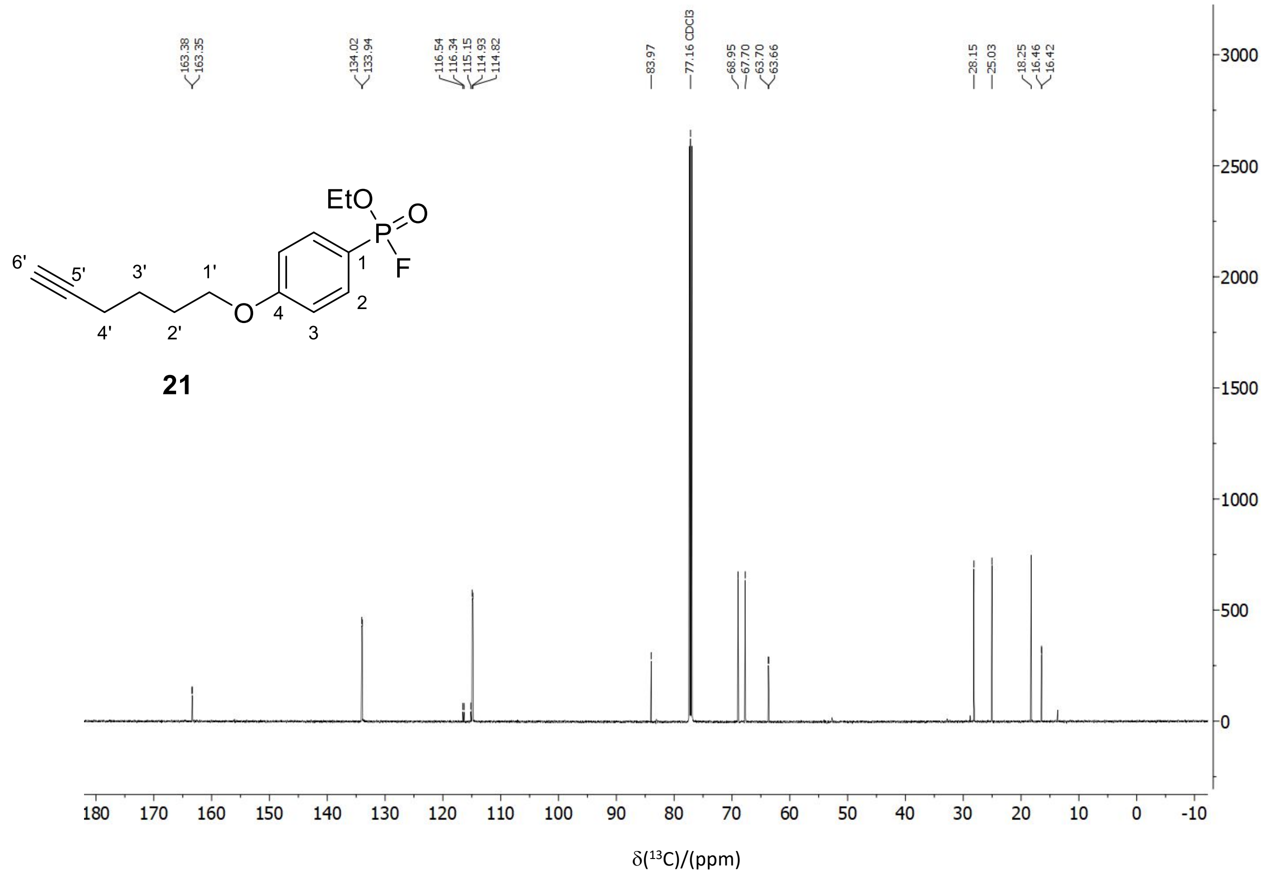
163.38
163.35
134.02
133.94
116.54
116.34
115.15
114.93
114.82
83.97
77.16 CDCl3
68.95
67.70
63.70
63.66
28.15
25.03
18.25
16.46
16.42
EtO
P
O
F
6'
5'
3'
1'
1
4
2
3
O
4'
2'
21
δ(¹³C)/(ppm)

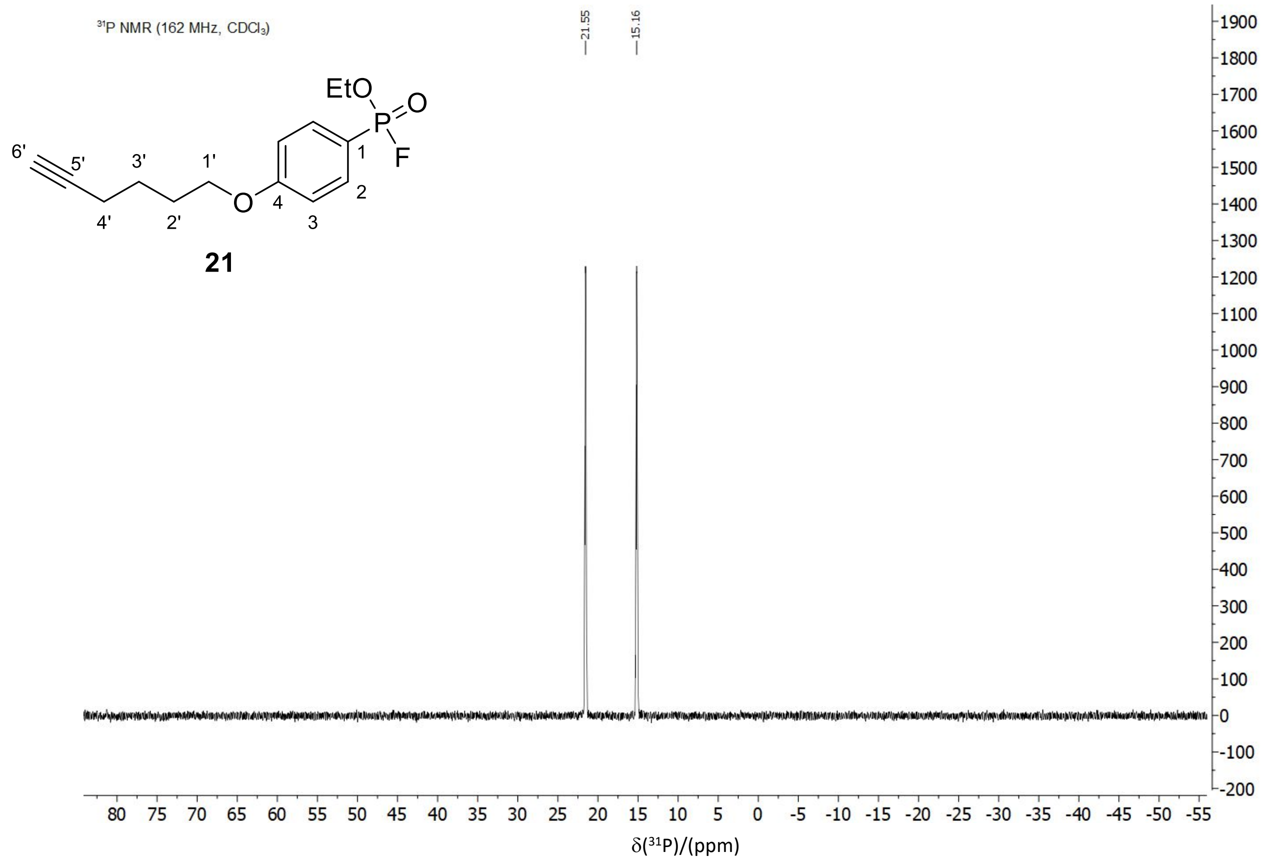

31P NMR (162 MHz, CDCl3)
21.55
15.16
EtO
P
O
F
1
2
3
4
O
1'
2'
3'
4'
5'
6'
21
δ(31P)/(ppm)

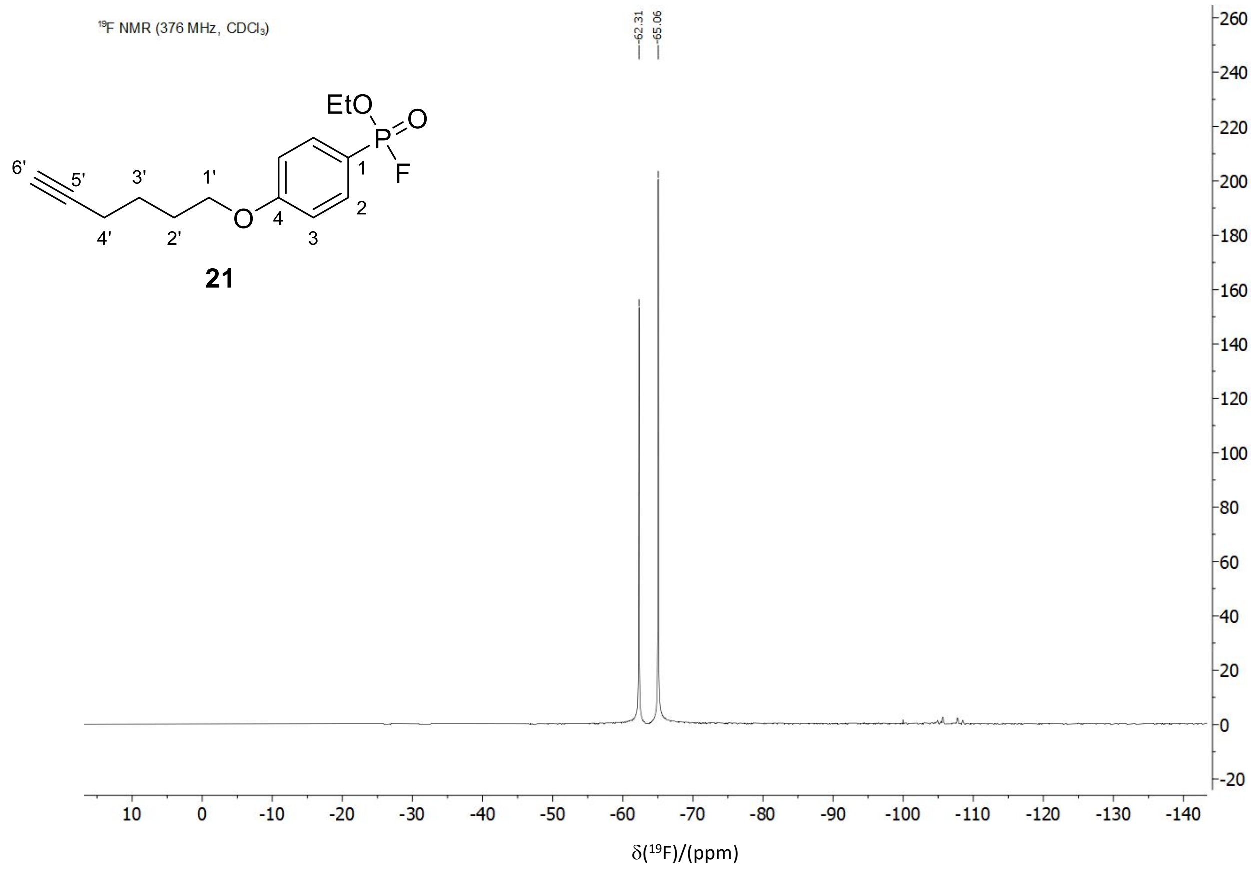

19F NMR (376 MHz, CDCl3)
-62.31
-65.06
EtO
P
O
F
1
2
3
4
O
1'
2'
3'
4'
5'
6'
21
δ(19F)/(ppm)

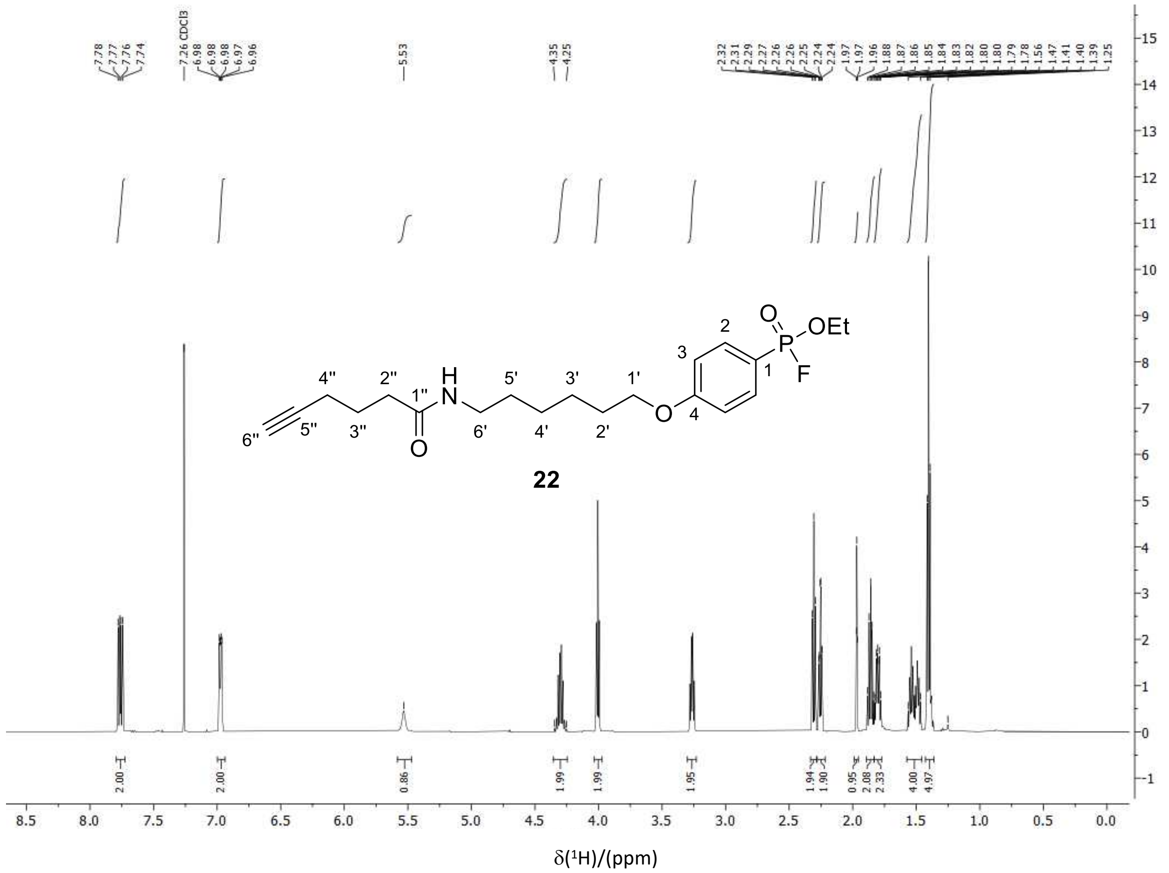
22
δ(¹H)/(ppm)

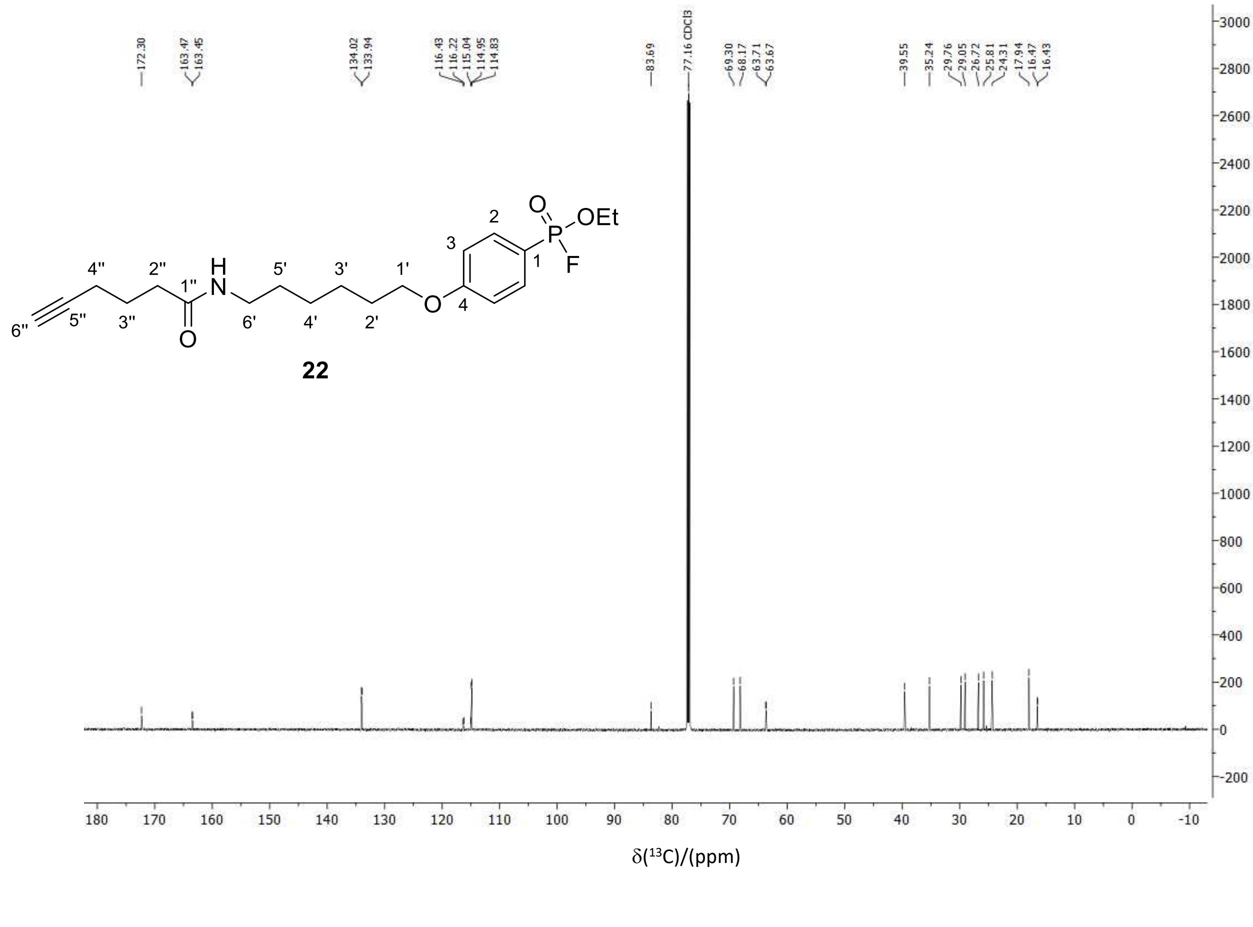
22
δ(¹³C)/(ppm)

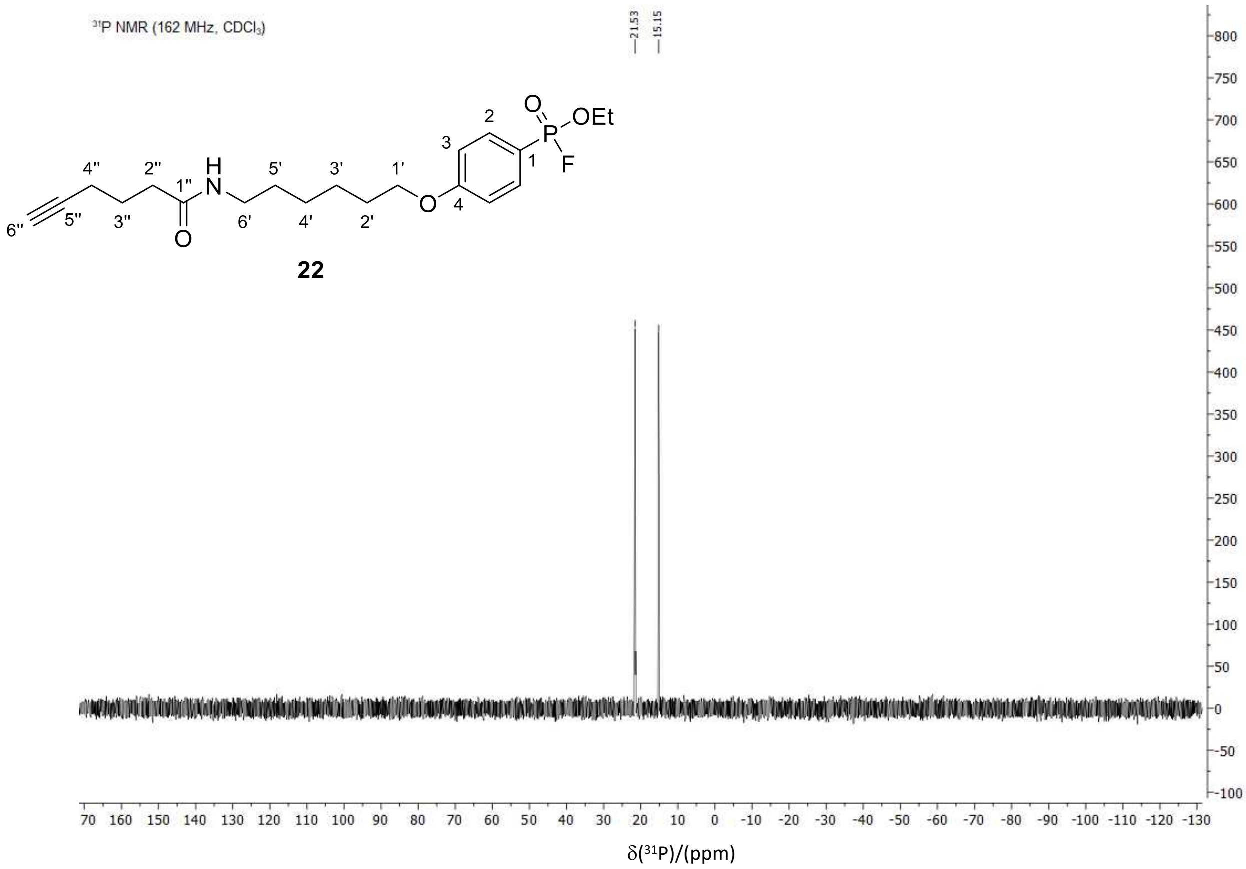
31P NMR (162 MHz, CDCl3)
21.53
15.15
OEt
F
22
δ(31P)/(ppm)

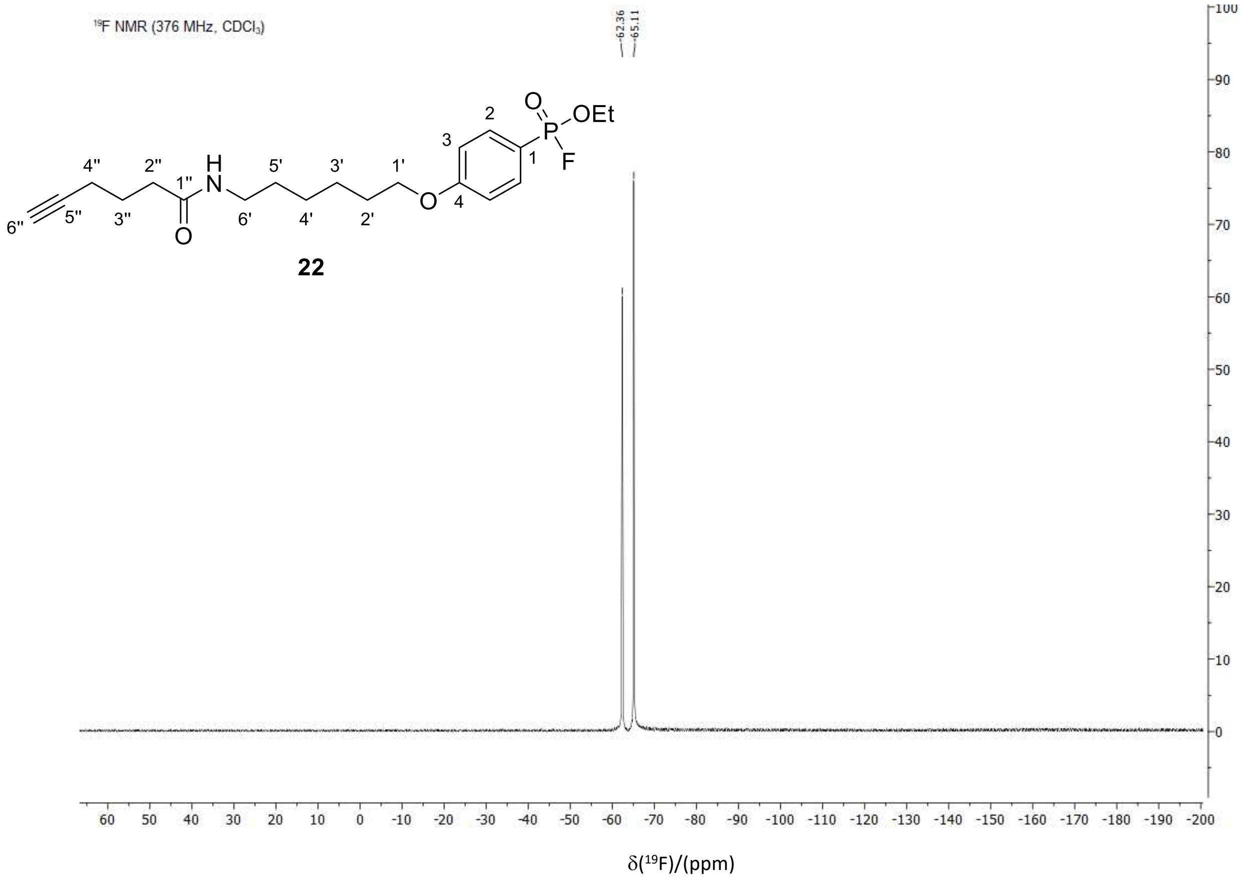
19F NMR (376 MHz, CDCl3)
-62.36
-65.11
OEt
F
22
δ(19F)/(ppm)

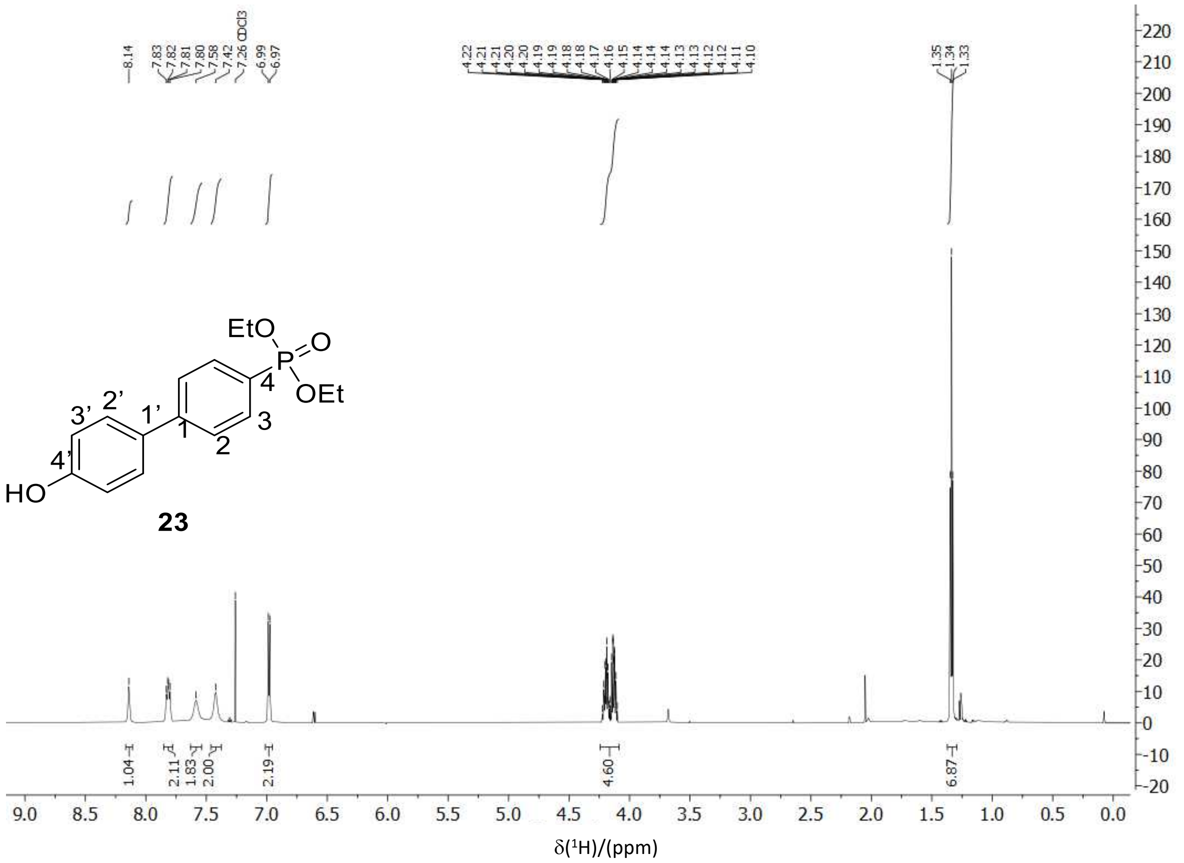
8.14
7.83
7.82
7.81
7.80
7.58
7.42
7.26 CDCl3
6.99
6.97
4.22
4.21
4.21
4.20
4.20
4.19
4.19
4.18
4.18
4.17
4.16
4.15
4.14
4.14
4.14
4.13
4.13
4.13
4.12
4.12
4.11
4.10
1.35
1.34
1.33
EtO
P
O
4
OEt
3' 2' 1'
1
3
2
4'
HO
23
1.04
2.11
1.83
2.00
2.19
4.60
6.87
δ(¹H)/(ppm)

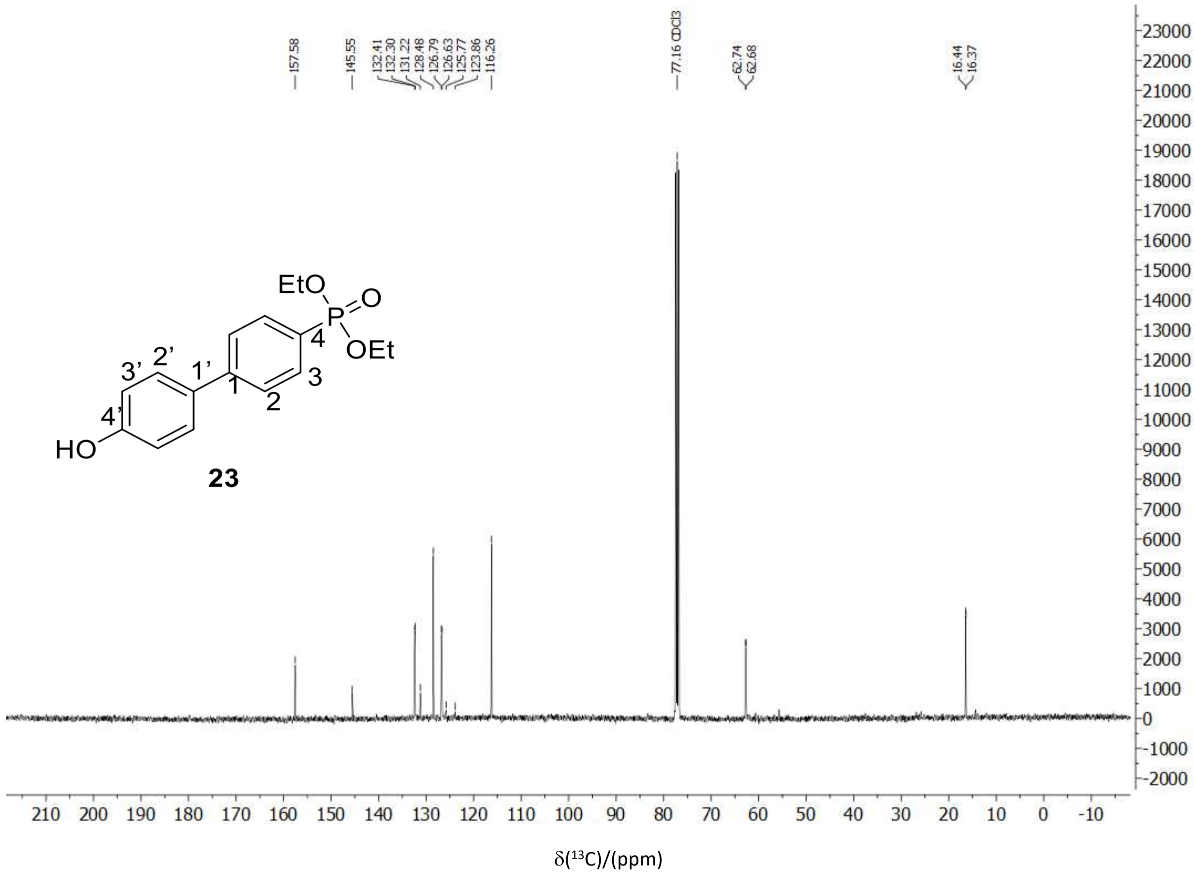
157.58
145.55
132.41
132.30
131.22
128.48
126.79
126.63
125.77
123.86
116.26
77.16 CDCl3
62.74
62.68
16.44
16.37
EtO
P
O
4
OEt
3' 2' 1'
1
3
2
4'
HO
23
δ(¹³C)/(ppm)

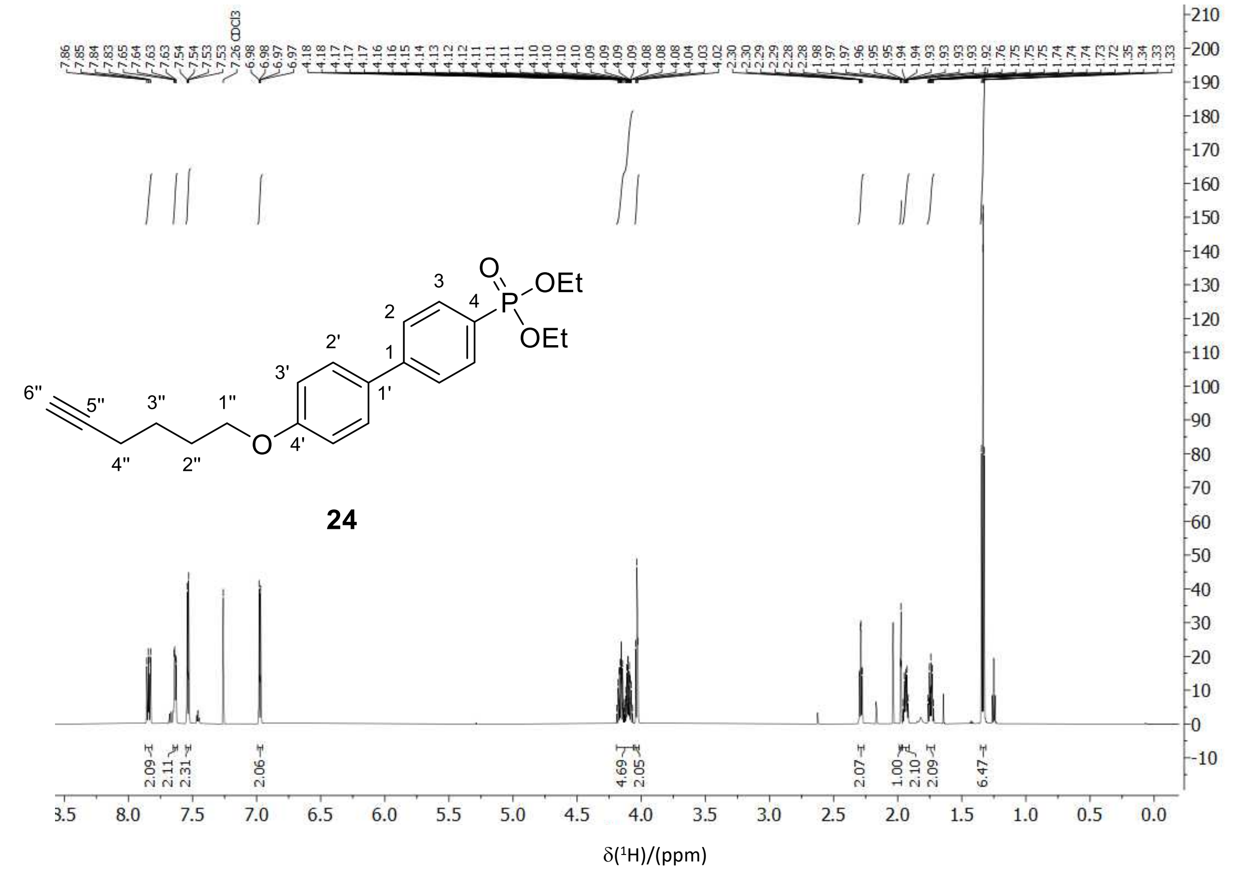

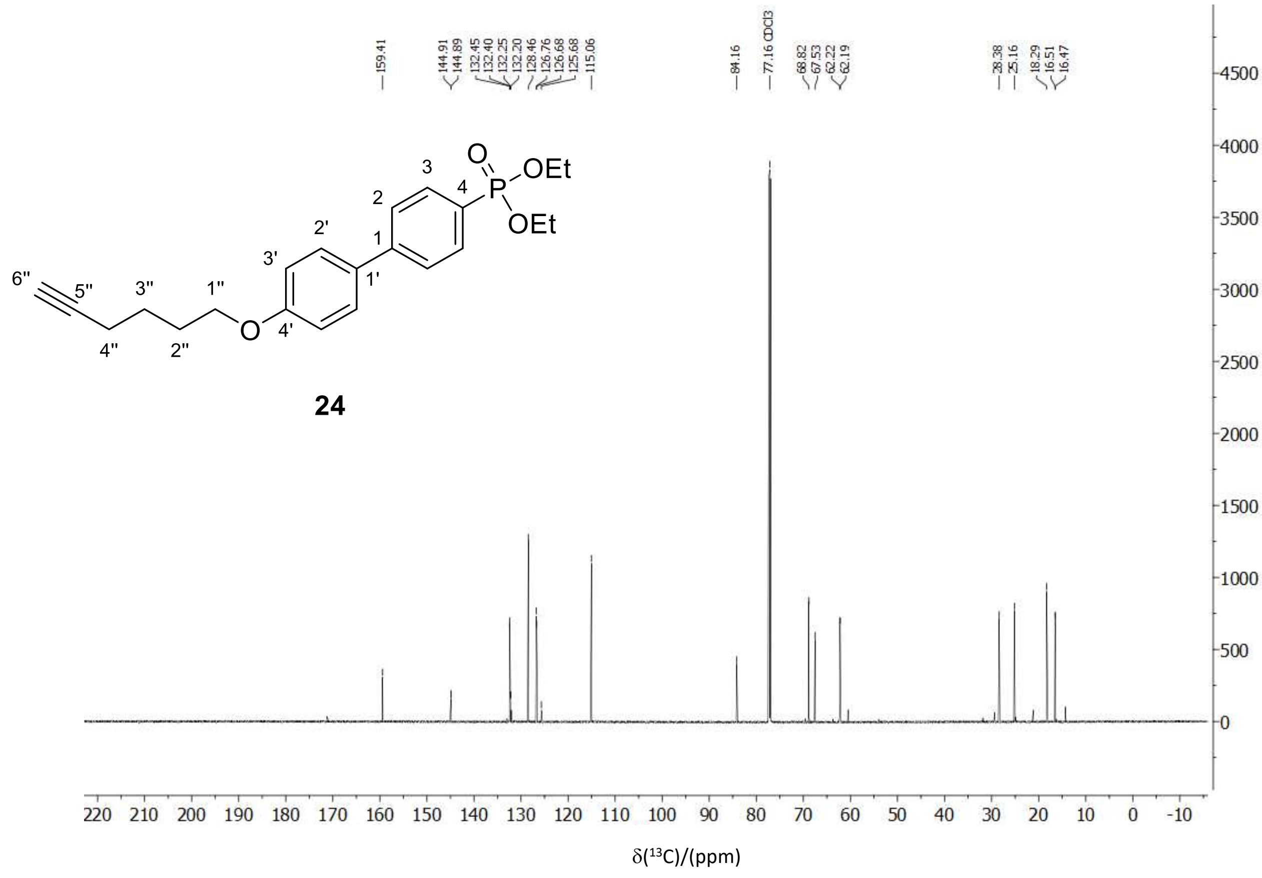

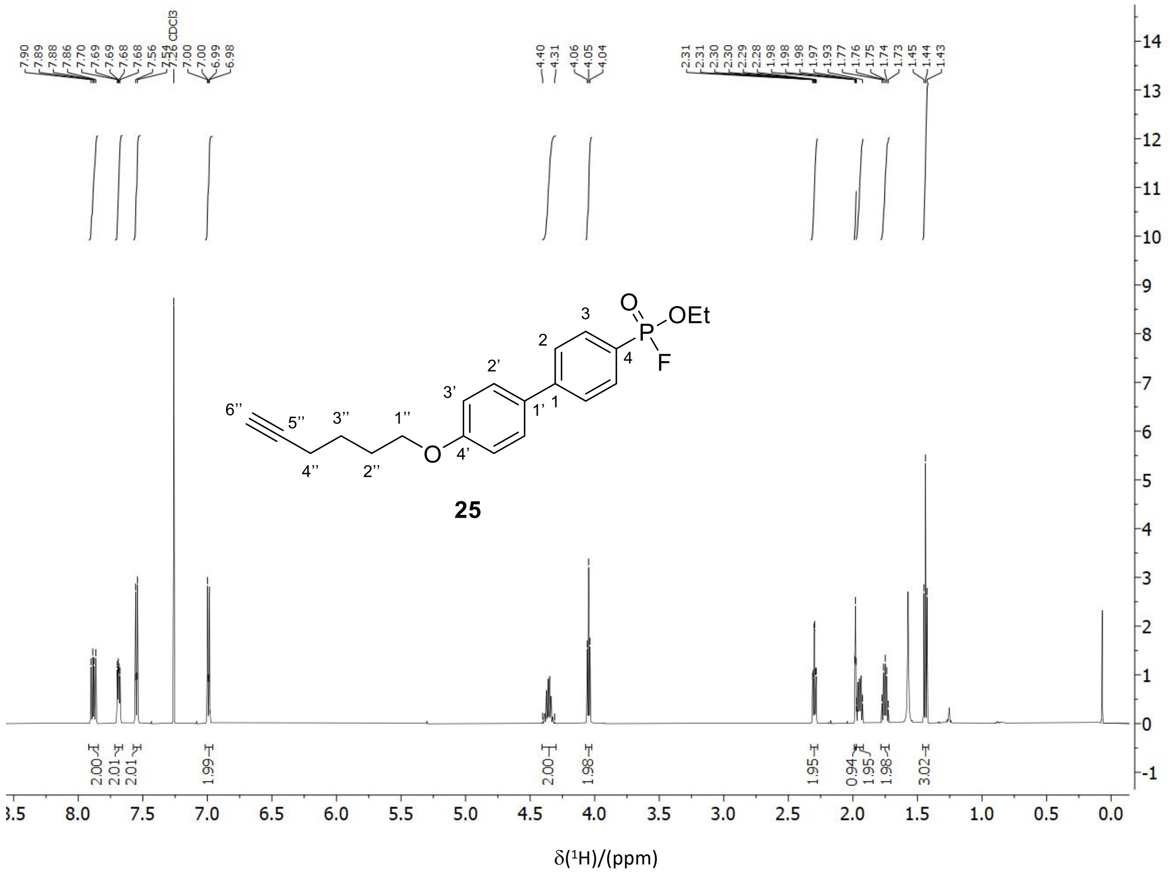
δ(¹H)/(ppm)
25

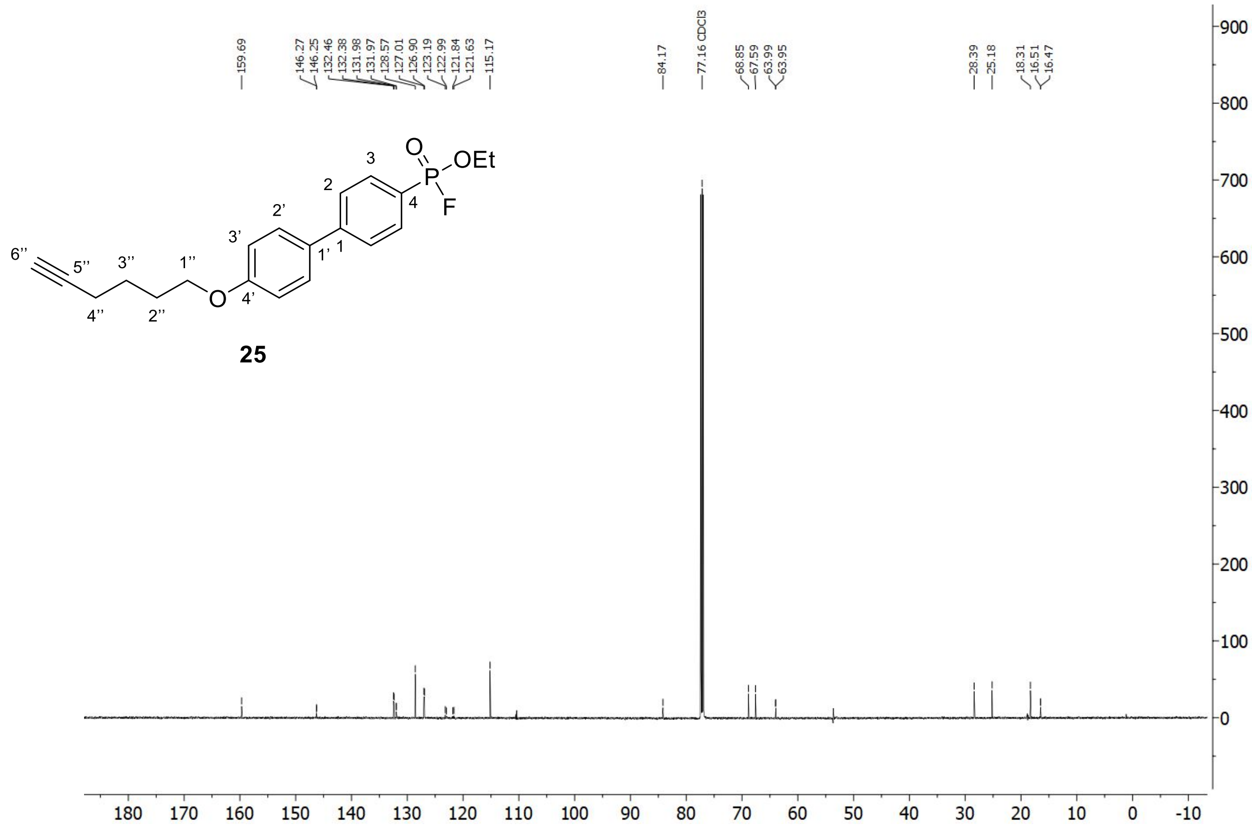
δ(¹³C)/(ppm)
25

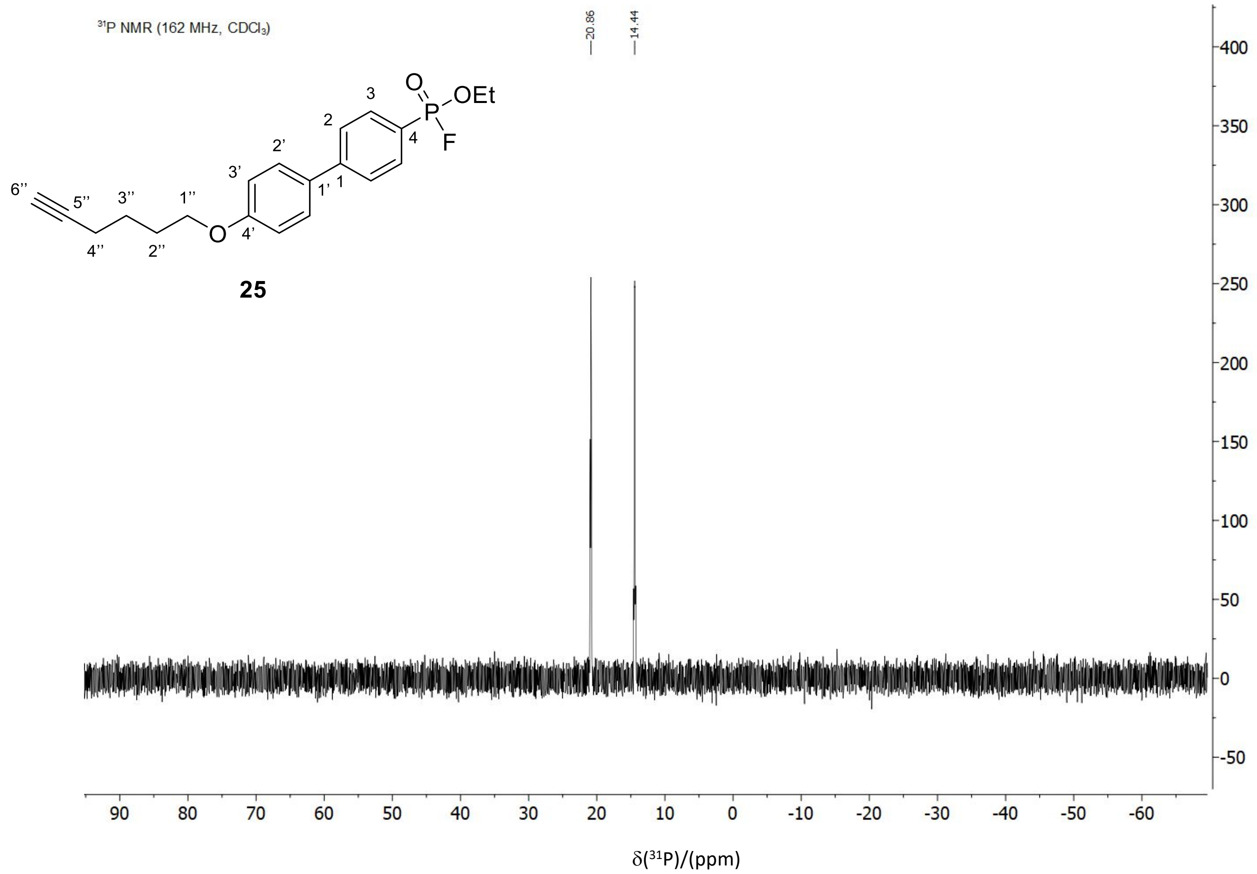

^{31}P NMR (162 MHz, CDCl$_3$)
—20.86
—14.44
OEt
F
25
δ(^{31}P)/(ppm)

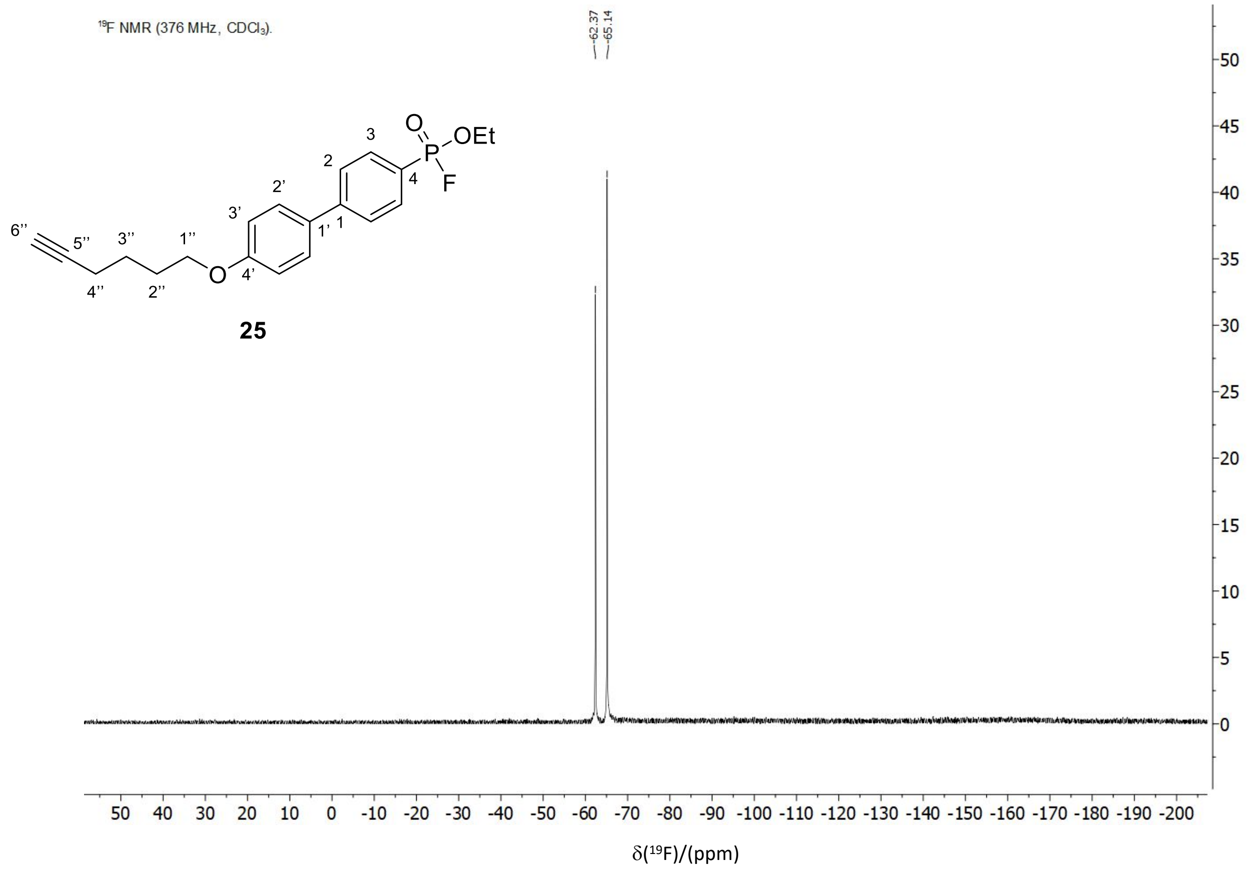

^{19}F NMR (376 MHz, CDCl$_3$).
—-62.37
—-65.14
OEt
F
25
δ(^{19}F)/(ppm)

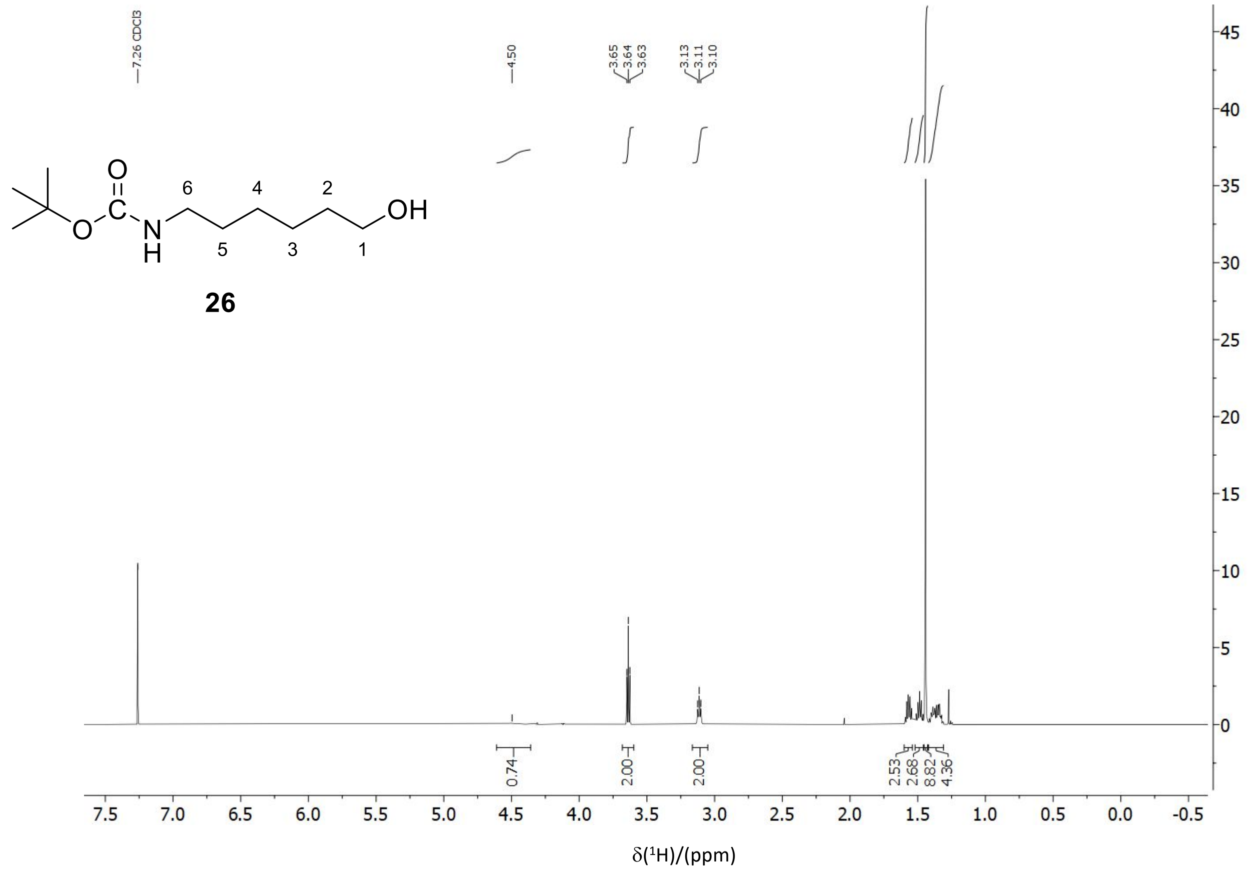
7.26 CDCl3
4.50
3.65
3.64
3.63
3.13
3.11
3.10
26
0.74
2.00
2.00
2.53
2.68
8.82
4.36
δ(¹H)/(ppm)

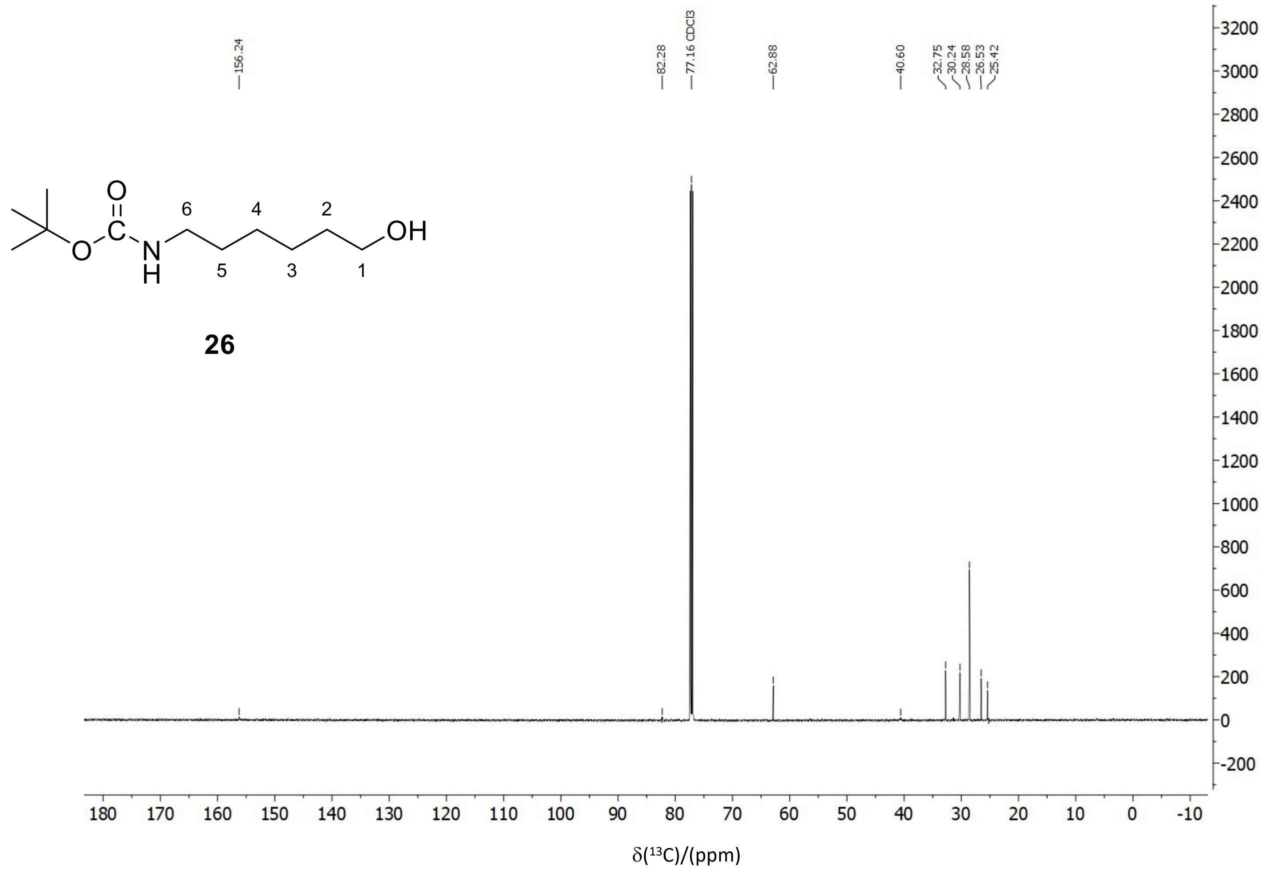
156.24
82.28
77.16 CDCl3
62.88
40.60
32.75
30.24
28.58
26.53
25.42
26
δ(¹³C)/(ppm)

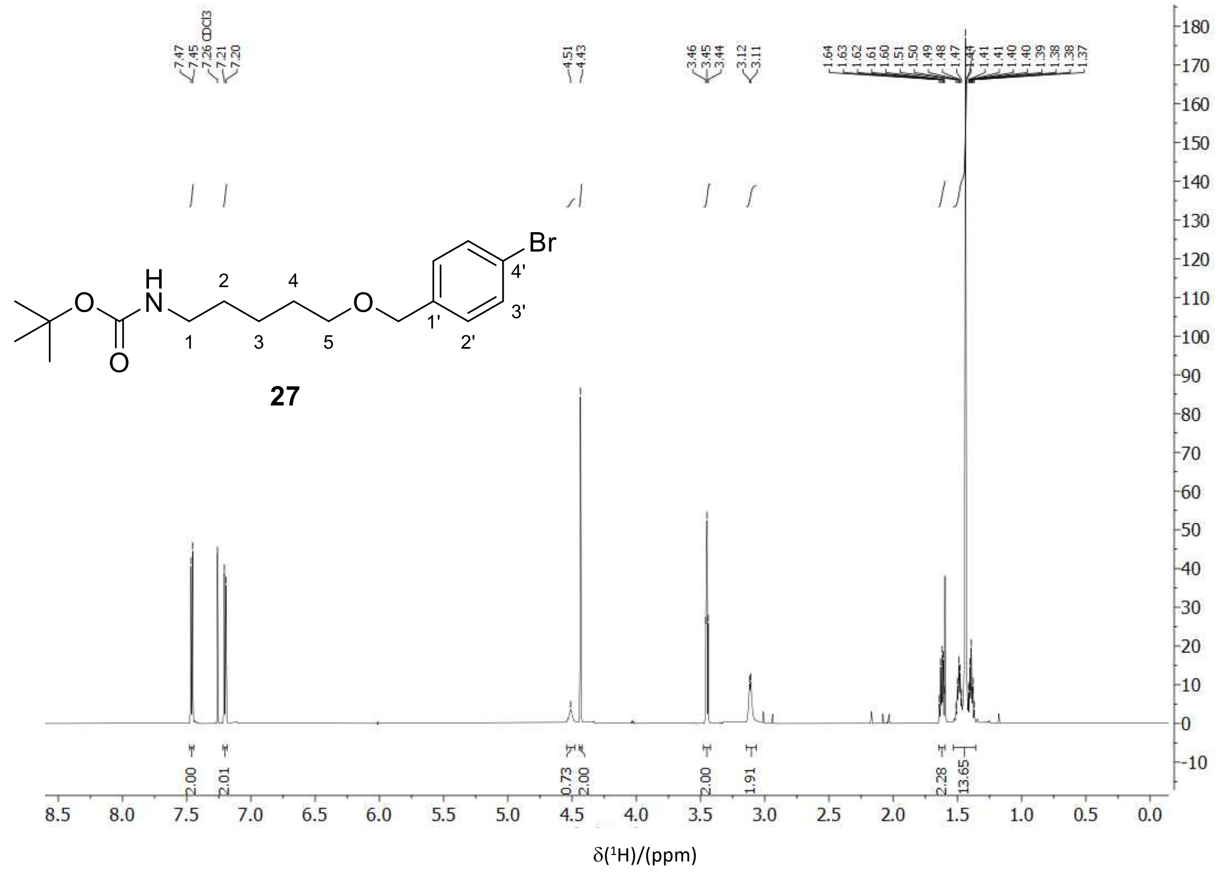

27
δ(¹H)/(ppm)

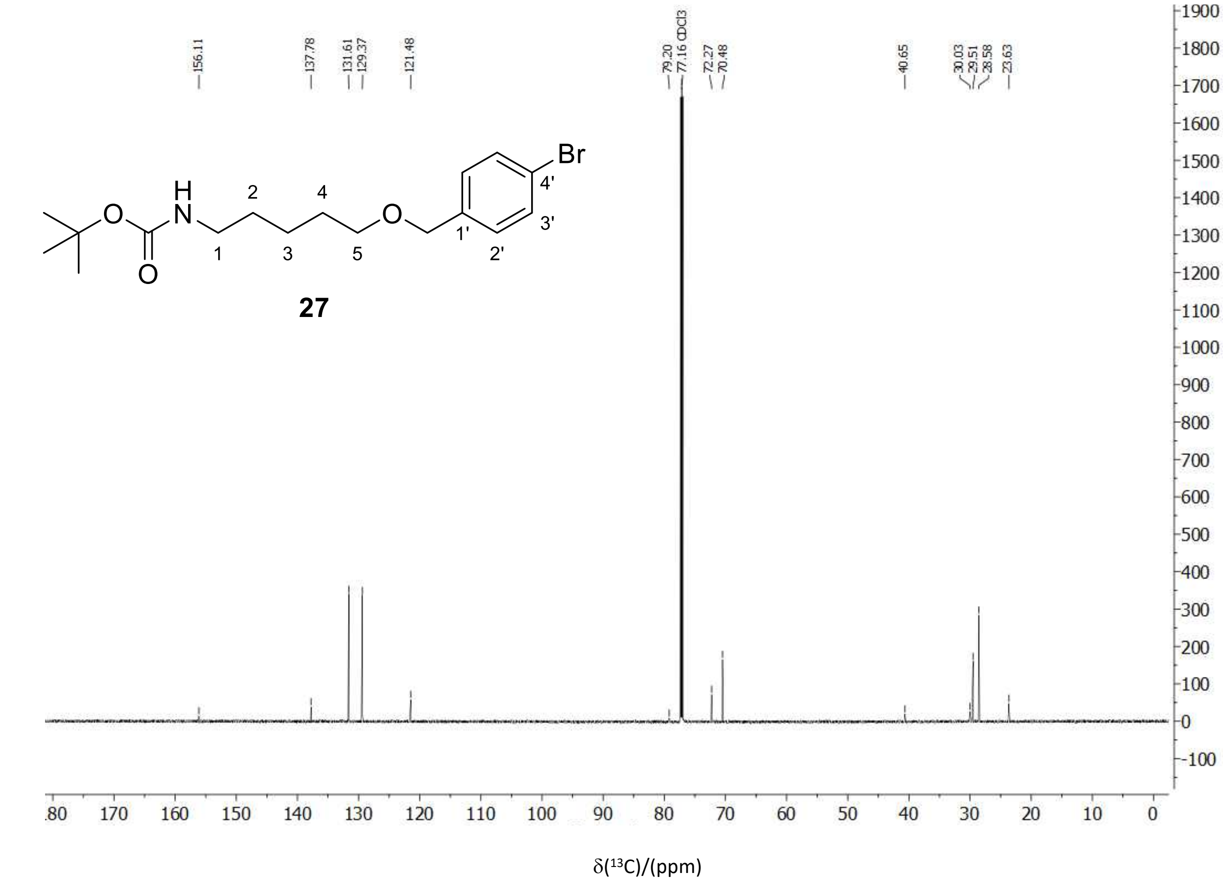

27
δ(¹³C)/(ppm)

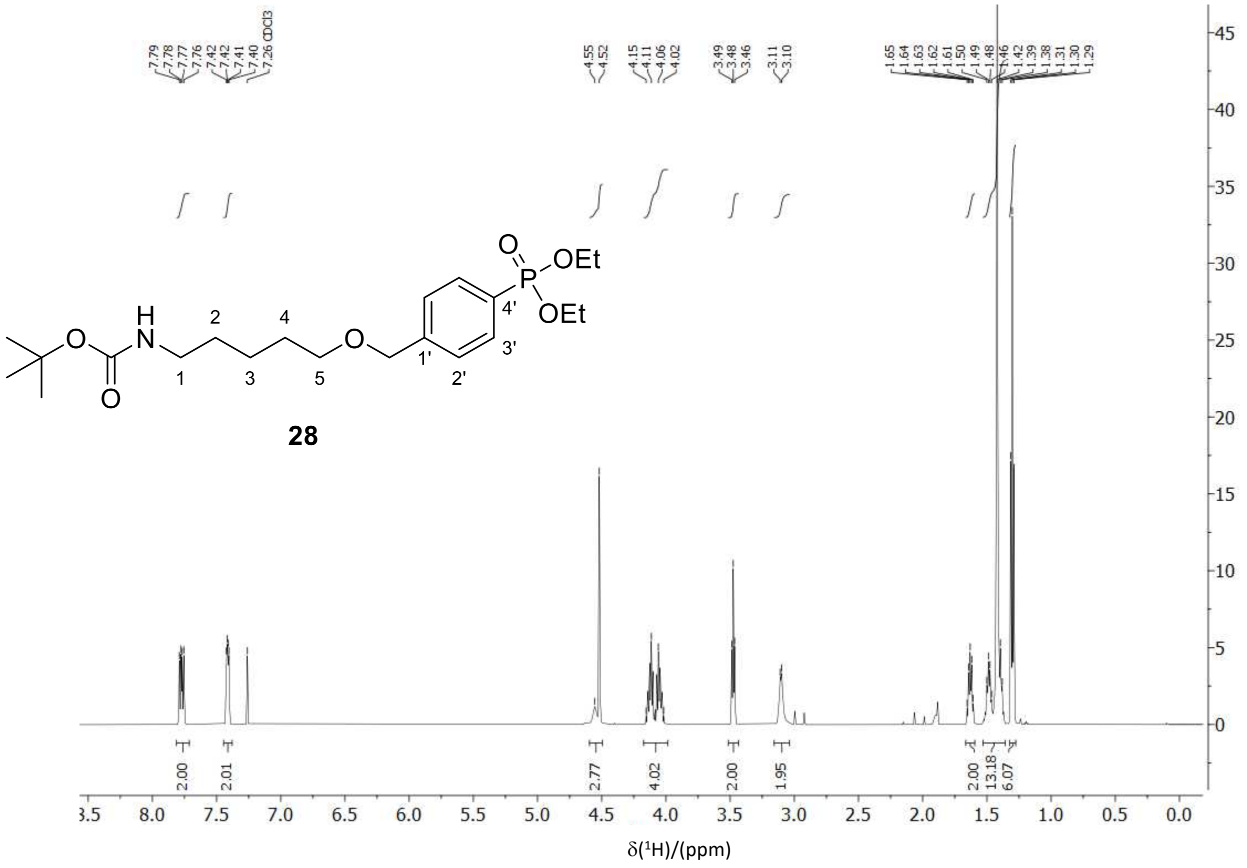

O
OEt
P
4'
OEt
H
N
O
O
O
2
4
1
3
5
1'
2'
3'
28
δ(¹H)/(ppm)

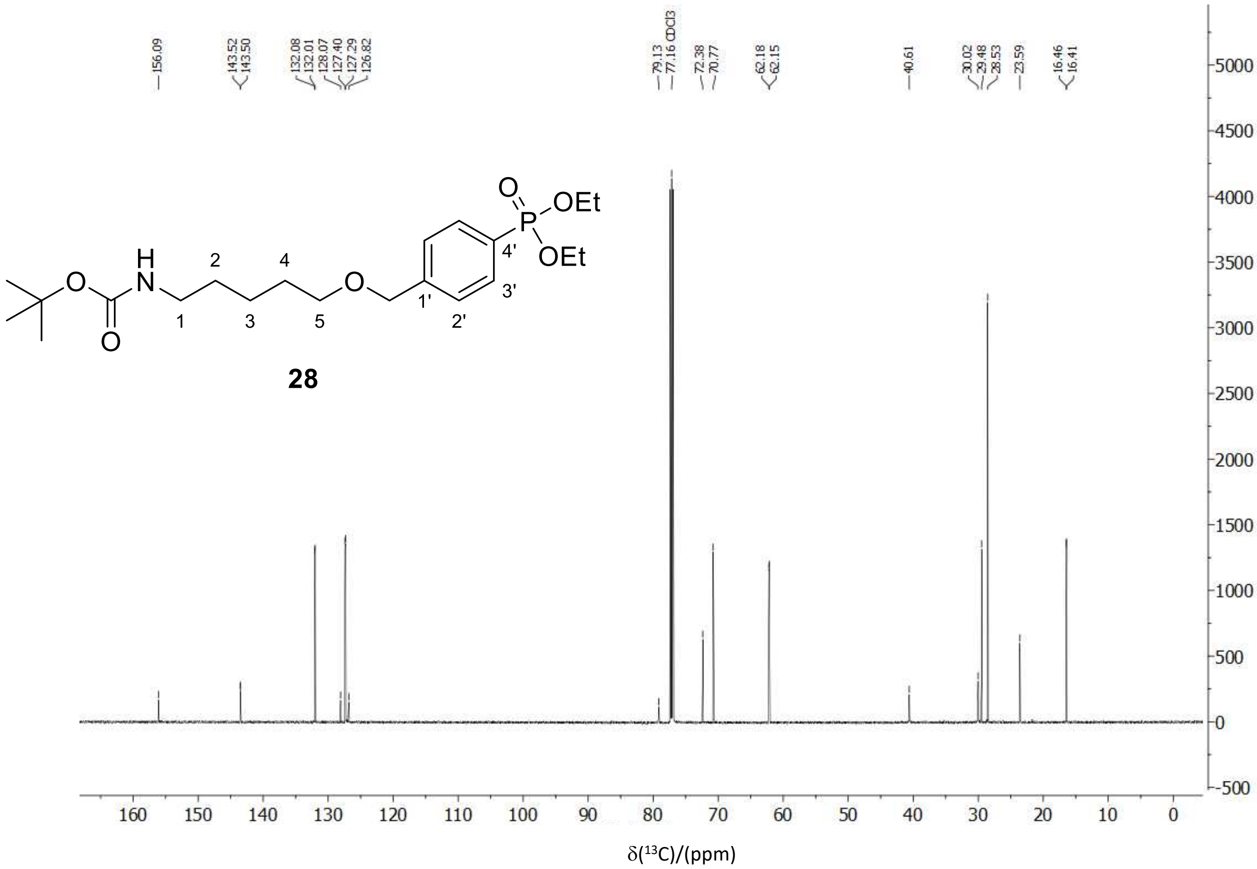

O
OEt
P
4'
OEt
H
N
O
O
O
2
4
1
3
5
1'
2'
3'
28
δ(¹³C)/(ppm)

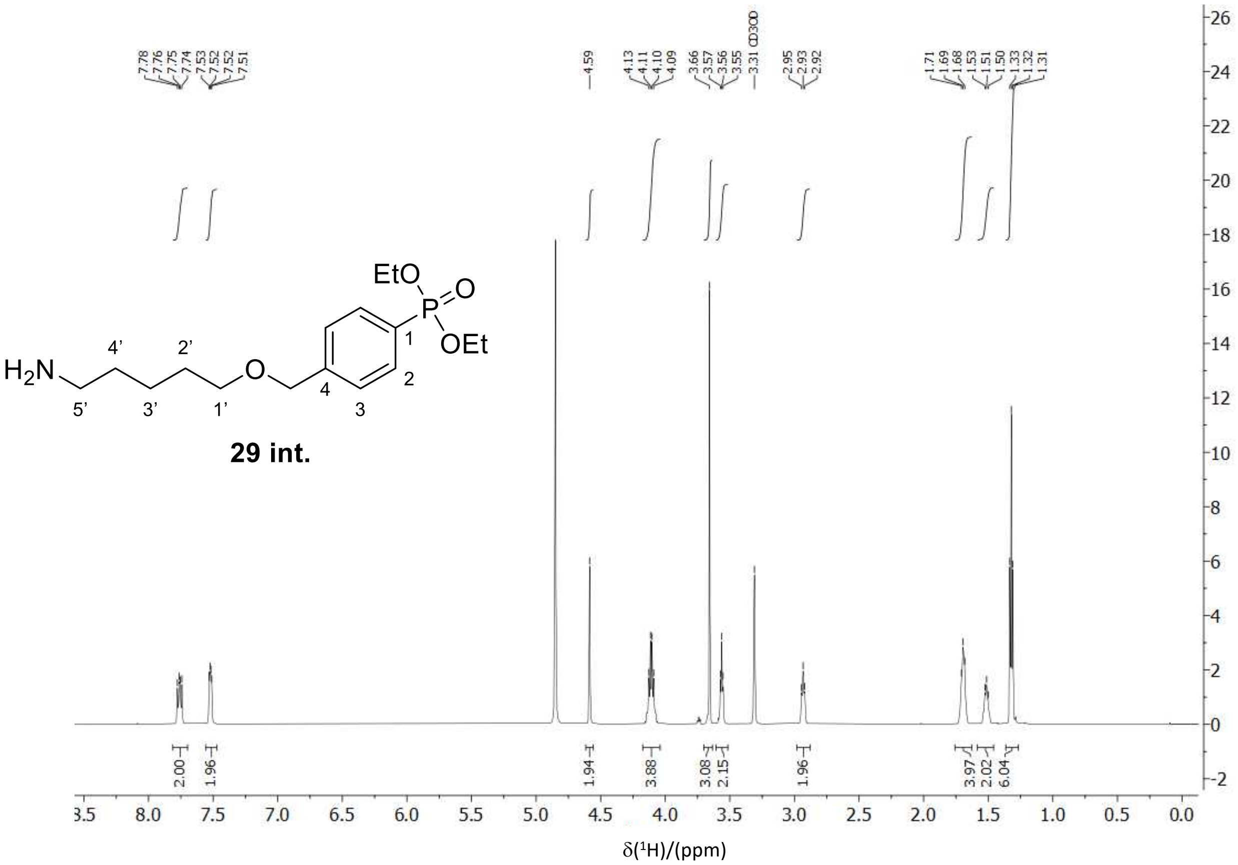
29 int.
δ(¹H)/(ppm)

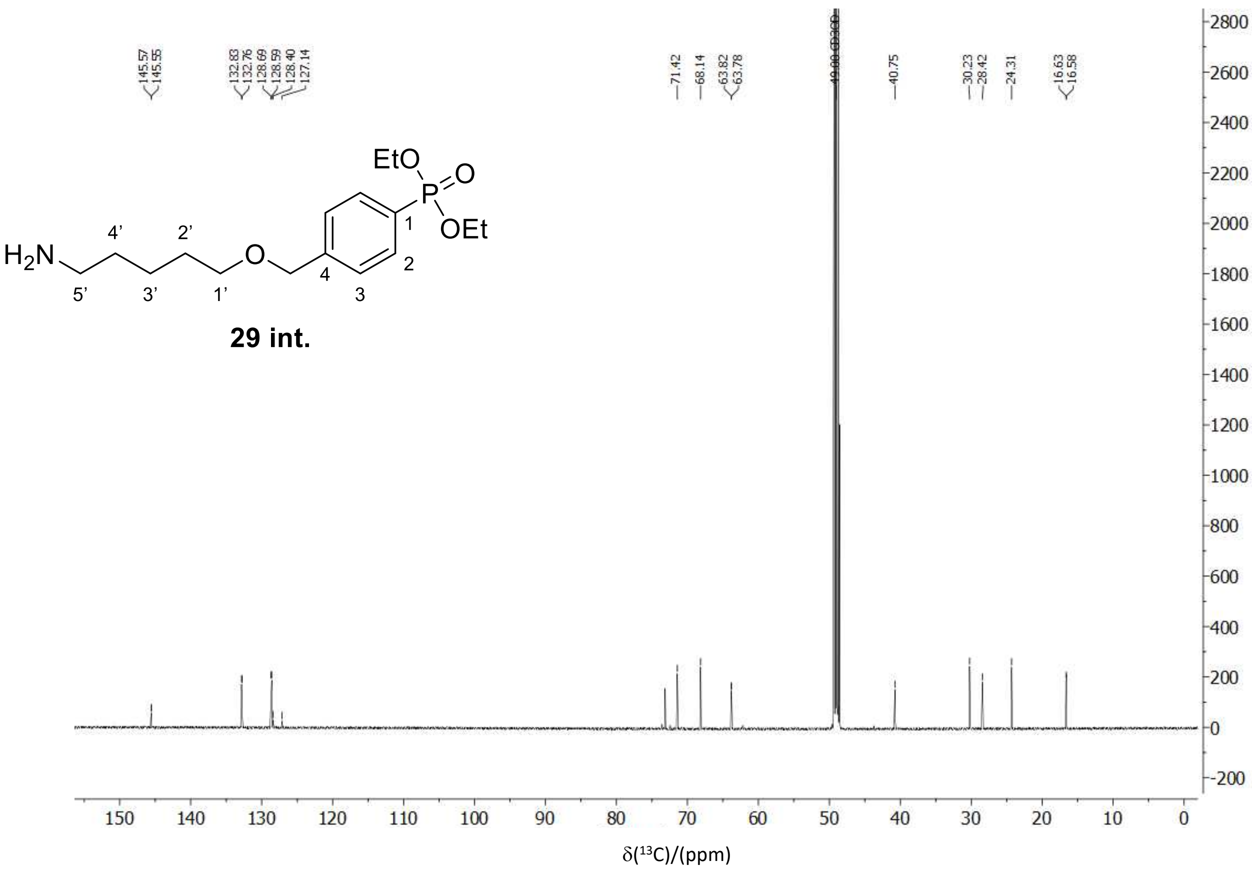
29 int.
δ(¹³C)/(ppm)

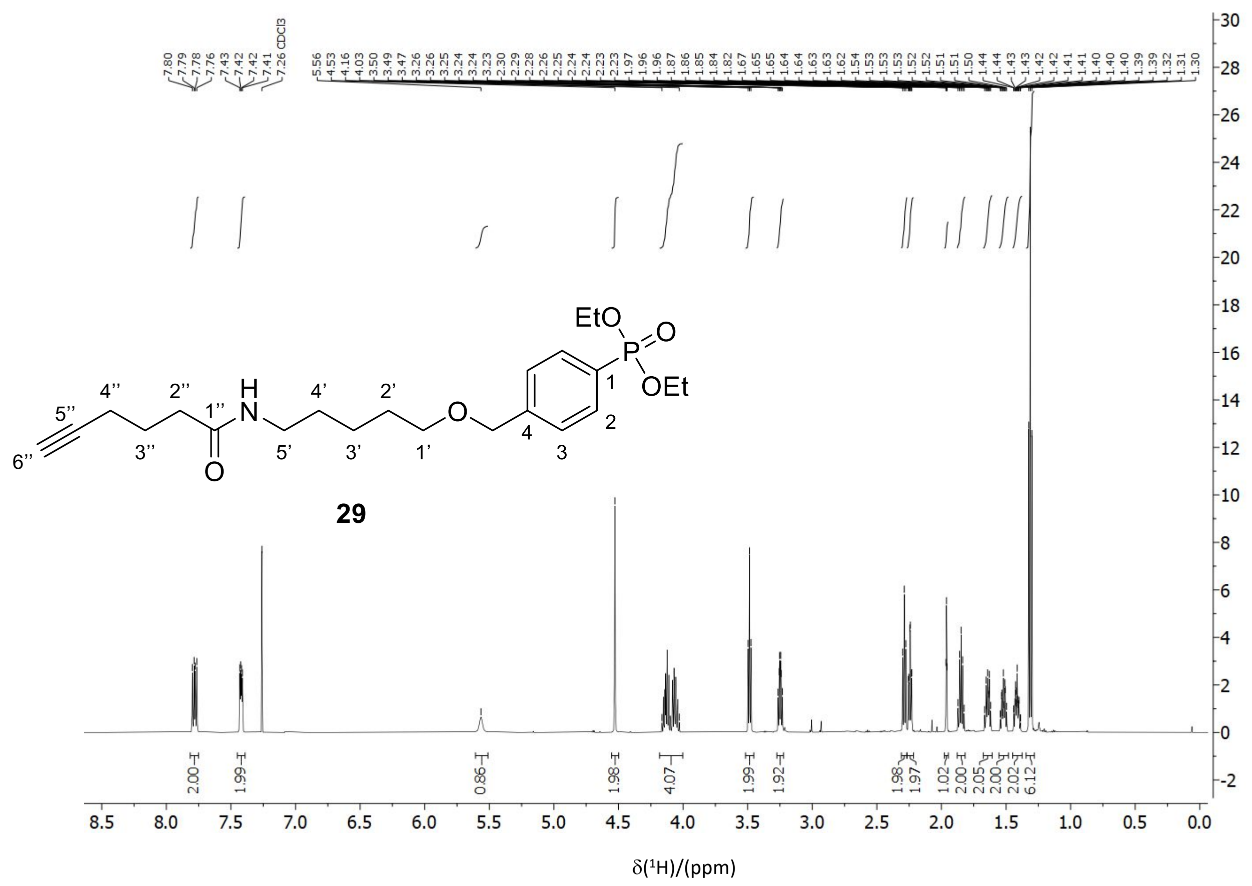

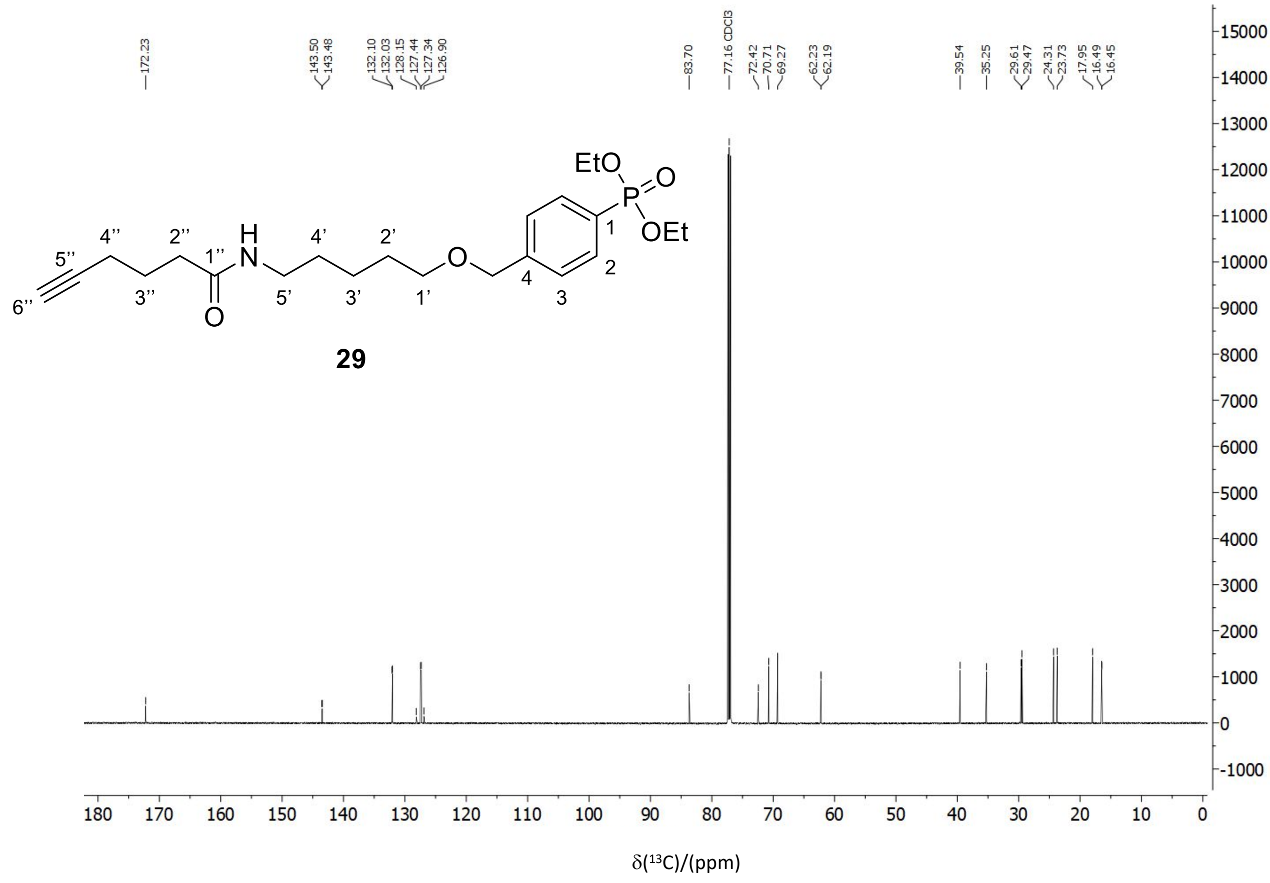

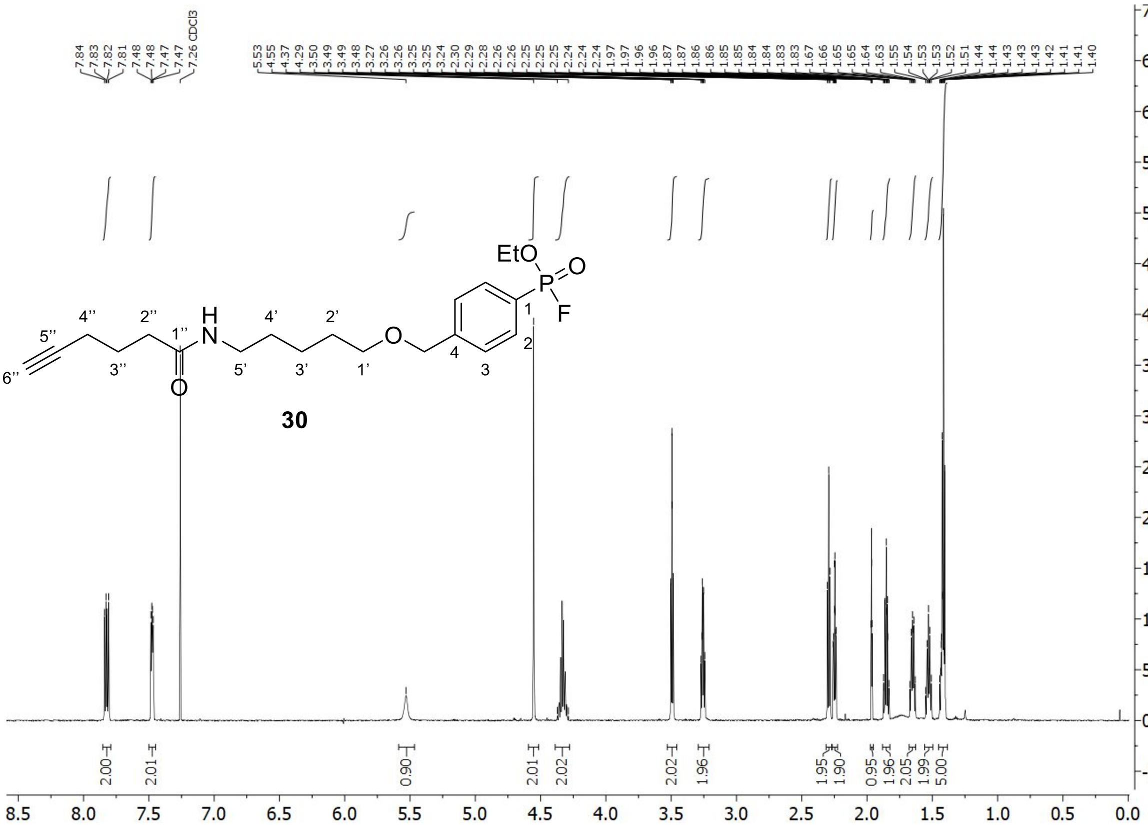

30
δ(¹H)/(ppm)

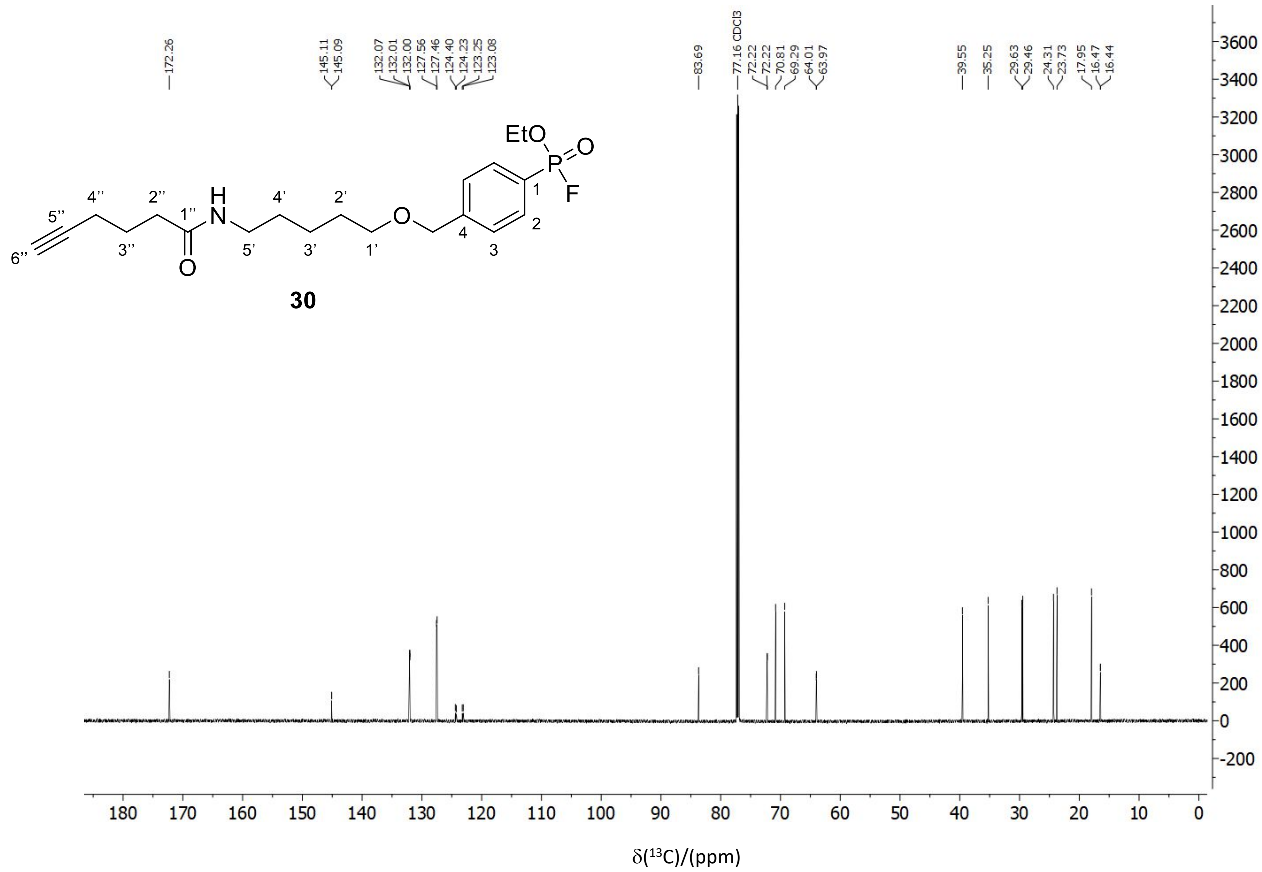

30
δ(¹³C)/(ppm)

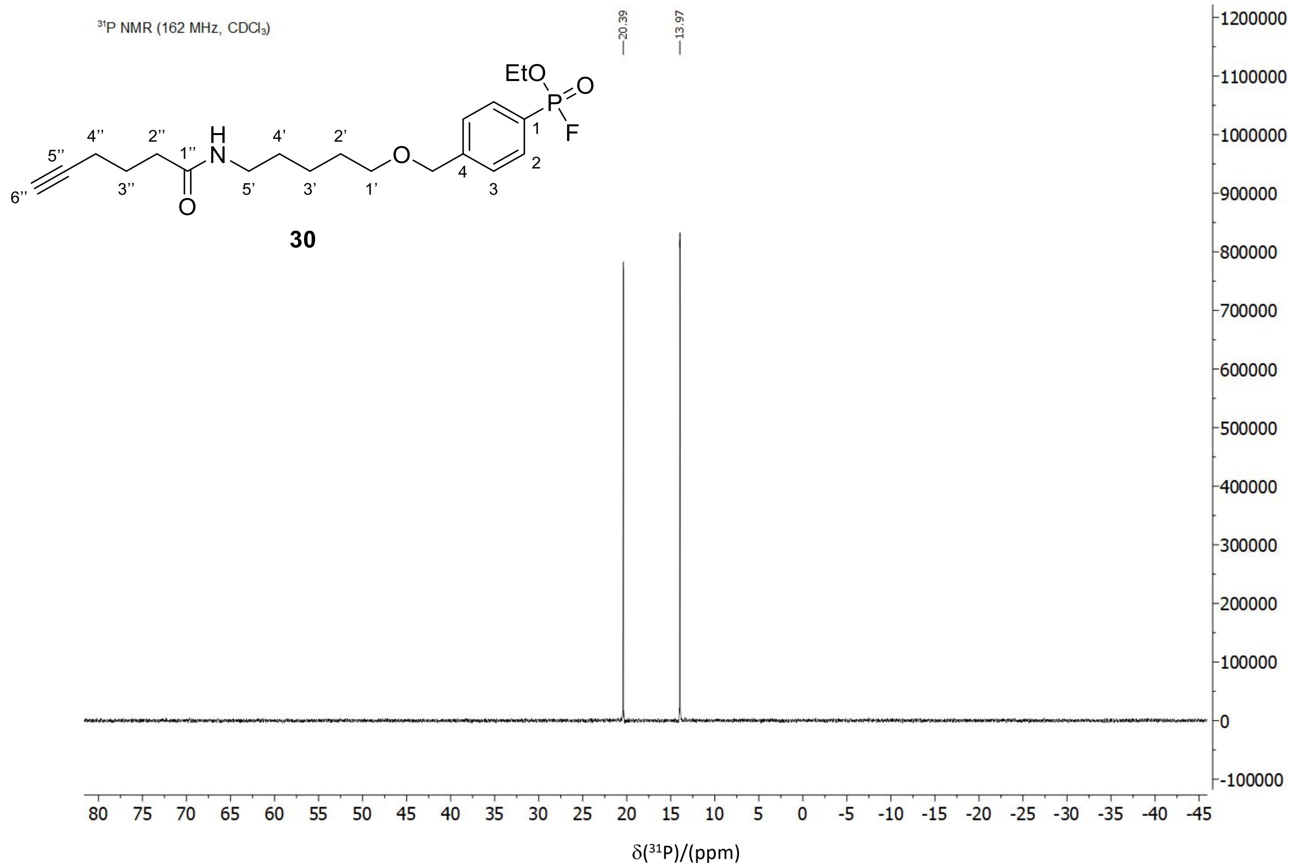

31P NMR (162 MHz, CDCl3)
20.39
13.97
EtO
P
O
F
1
2
3
4
1''
2''
3''
4''
5''
6''
1'
2'
3'
4'
5'
N
H
O
30
δ(31P)/(ppm)

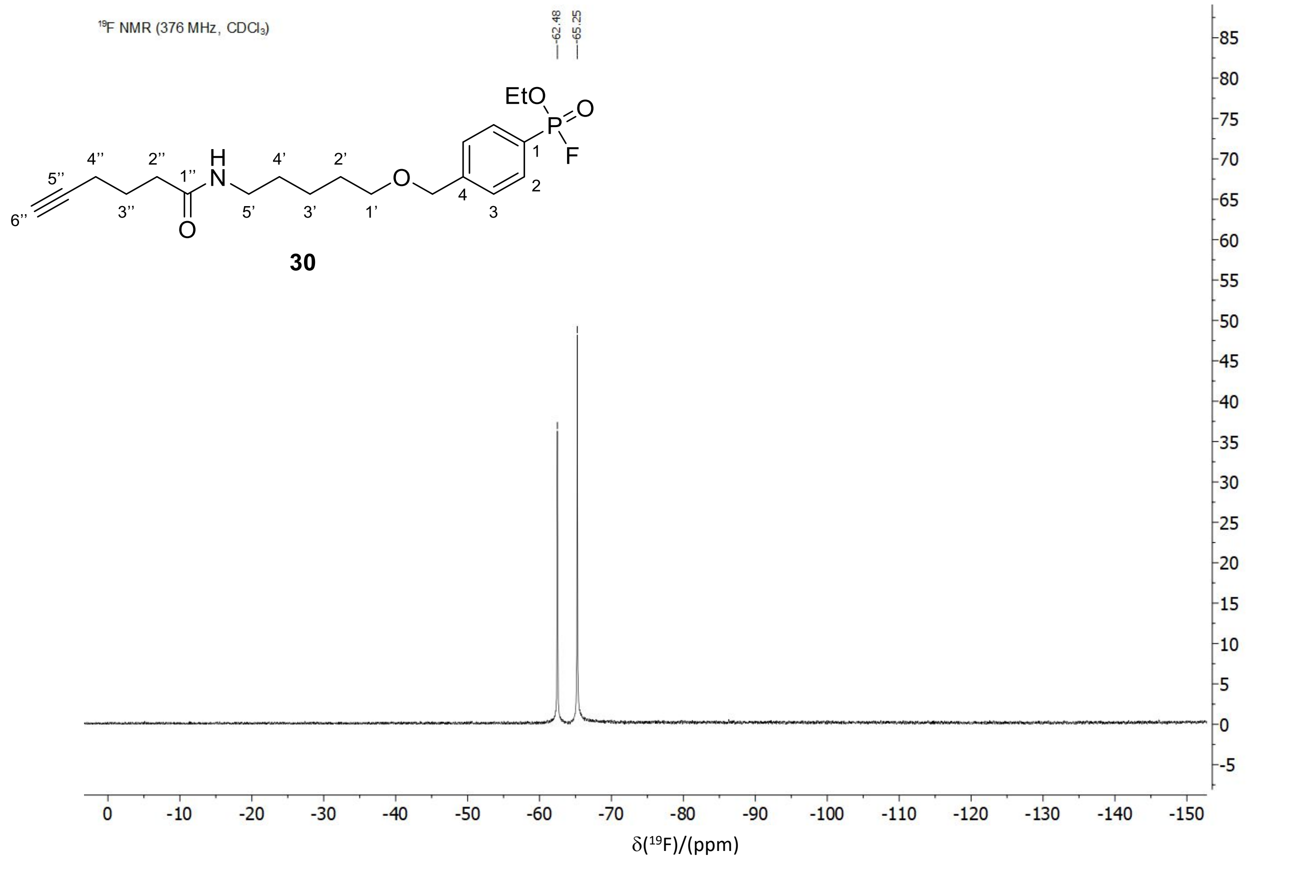

19F NMR (376 MHz, CDCl3)
-62.48
-65.25
EtO
P
O
F
1
2
3
4
1''
2''
3''
4''
5''
6''
1'
2'
3'
4'
5'
N
H
O
30
δ(19F)/(ppm)

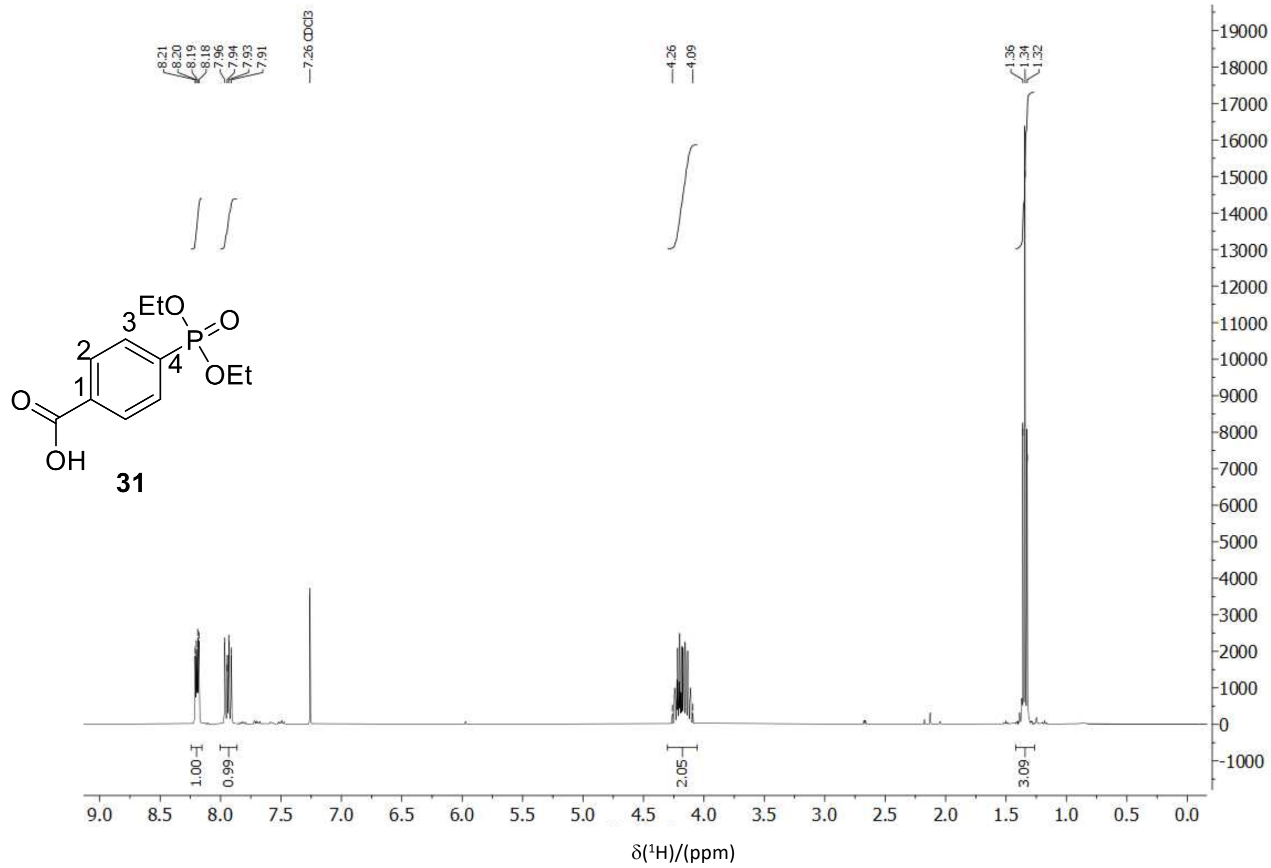
8.21
8.20
8.19
8.18
7.96
7.94
7.93
7.91
7.26 CDCl3
4.26
4.09
1.36
1.34
1.32
EtO
P
O
OEt
O
OH
1
2
3
4
31
1.00
0.99
2.05
3.09
δ(1H)/(ppm)

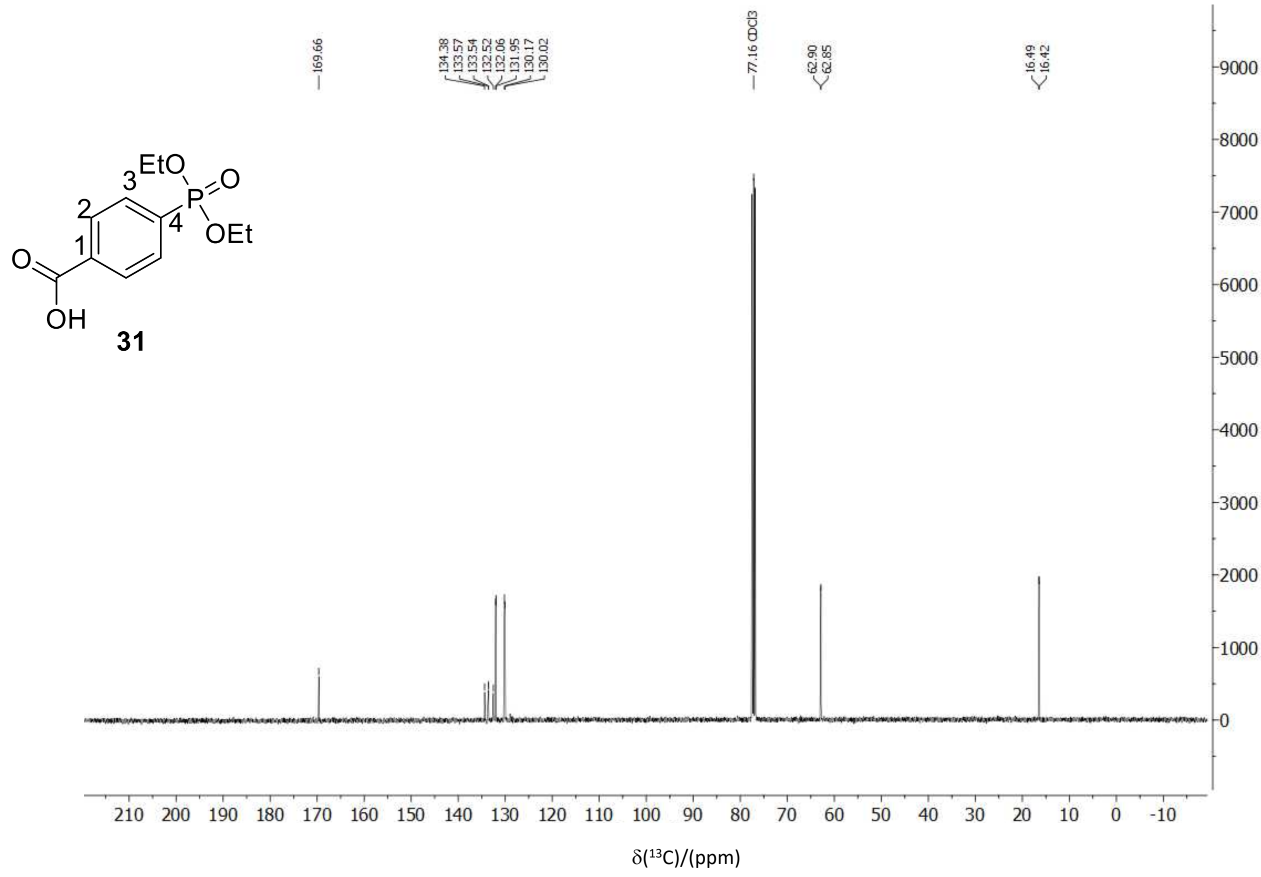
169.66
134.38
133.57
133.54
132.52
132.06
131.95
130.17
130.02
77.16 CDCl3
62.90
62.85
16.49
16.42
EtO
P
O
OEt
O
OH
1
2
3
4
31
δ(13C)/(ppm)

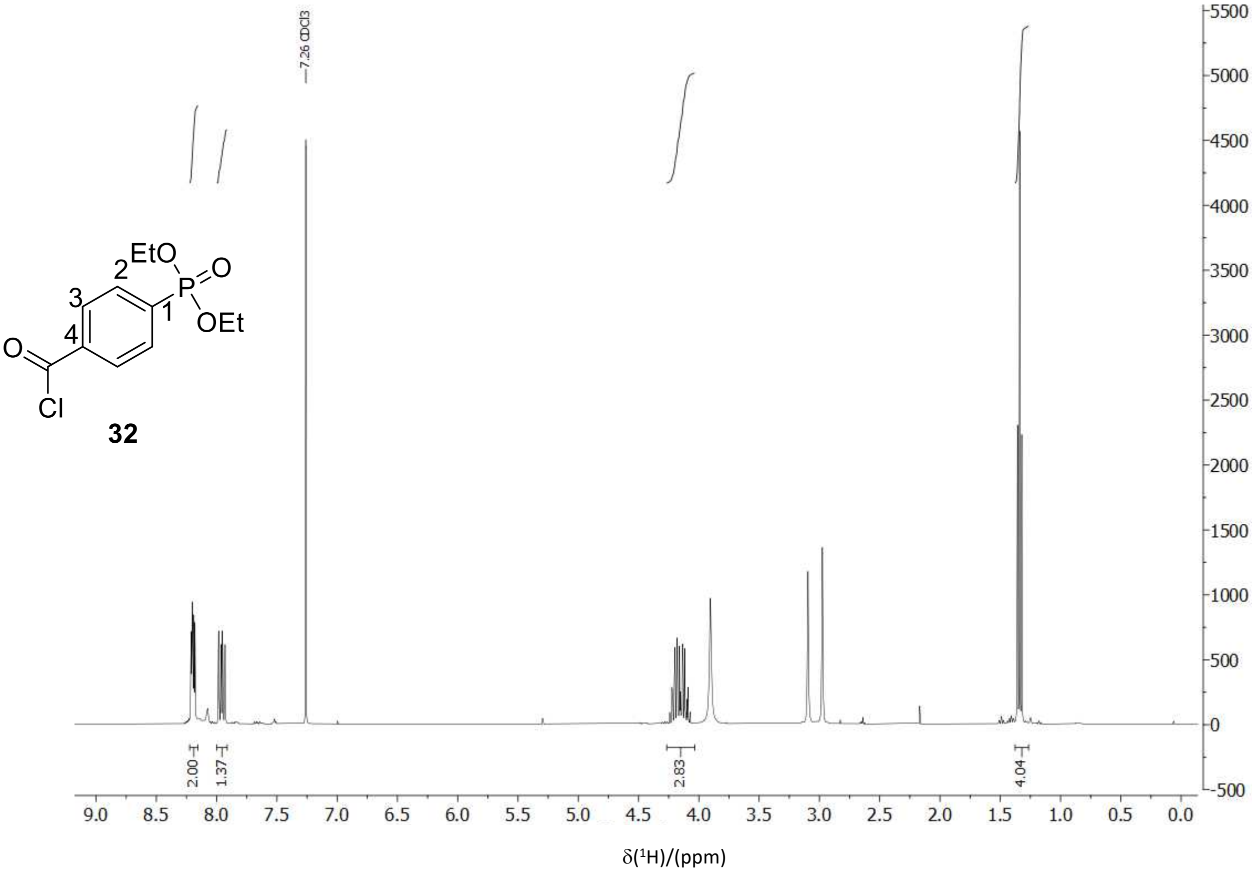
7.26 CDCl3
EtO
O
P
OEt
O
Cl
1
2
3
4
32
2.00
1.37
2.83
4.04
9.0 8.5 8.0 7.5 7.0 6.5 6.0 5.5 5.0 4.5 4.0 3.5 3.0 2.5 2.0 1.5 1.0 0.5 0.0
δ(¹H)/(ppm)
5500 5000 4500 4000 3500 3000 2500 2000 1500 1000 500 0 -500

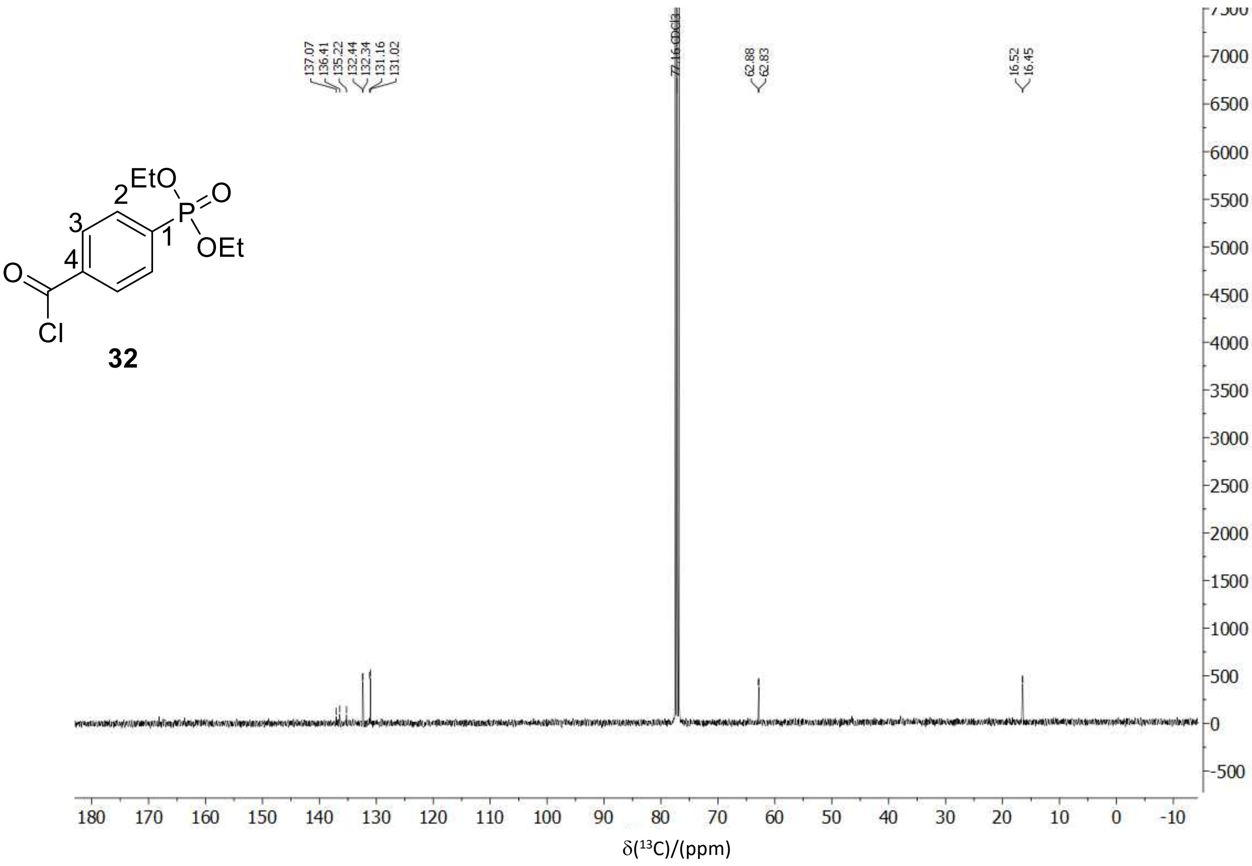
137.07
136.41
135.22
132.44
132.34
131.16
131.02
77.16 CDCl3
62.88
62.83
16.52
16.45
EtO
O
P
OEt
O
Cl
1
2
3
4
32
180 170 160 150 140 130 120 110 100 90 80 70 60 50 40 30 20 10 0 -10
δ(¹³C)/(ppm)
7000 6500 6000 5500 5000 4500 4000 3500 3000 2500 2000 1500 1000 500 0 -500

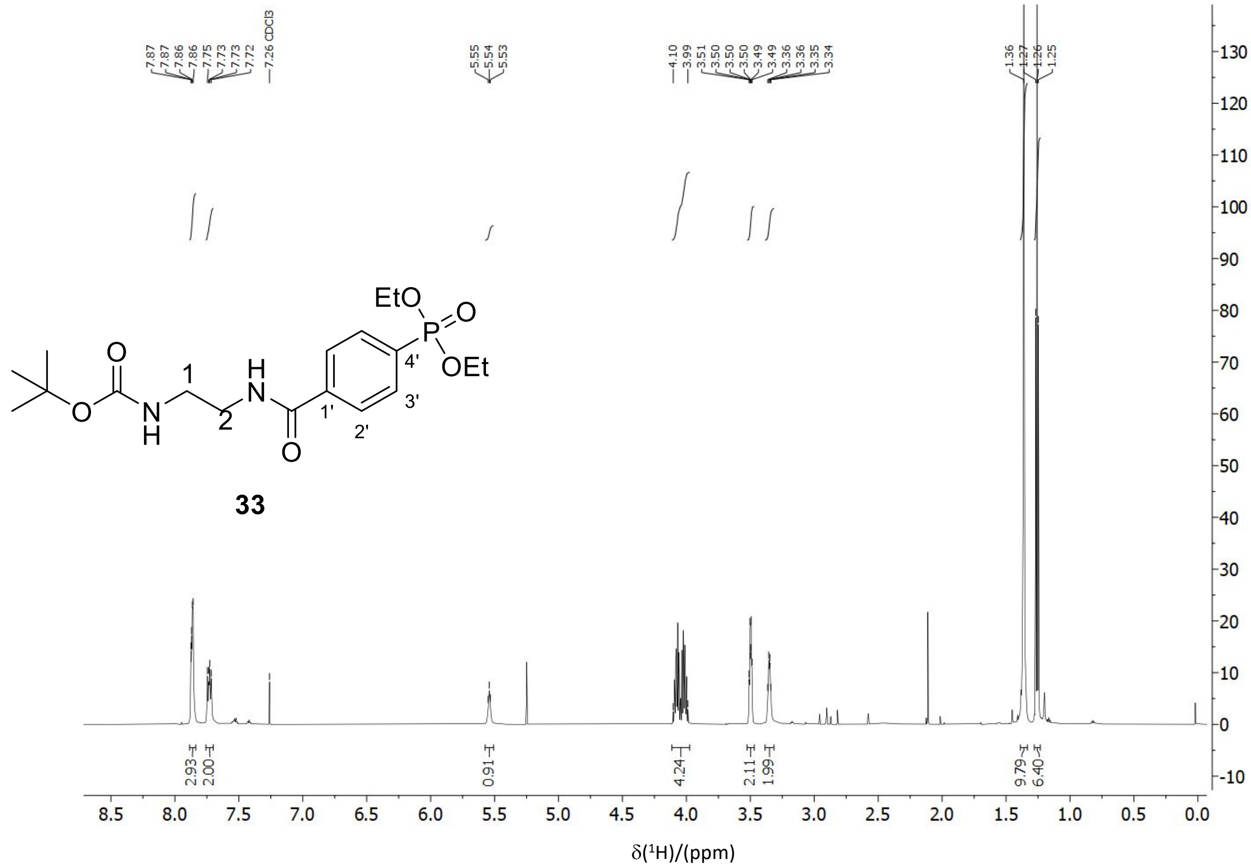

7.87
7.87
7.86
7.75
7.73
7.73
7.72
7.26 CDCl3
5.55
5.54
5.53
4.10
3.99
3.51
3.50
3.50
3.50
3.49
3.49
3.36
3.36
3.35
3.34
1.36
1.27
1.26
1.25
EtO
O
P
OEt
4'
3'
2'
1'
O
1
2
H
N
N
H
O
O
33
2.93
2.00
0.91
4.24
2.11
1.99
9.79
6.40
δ(1H)/(ppm)

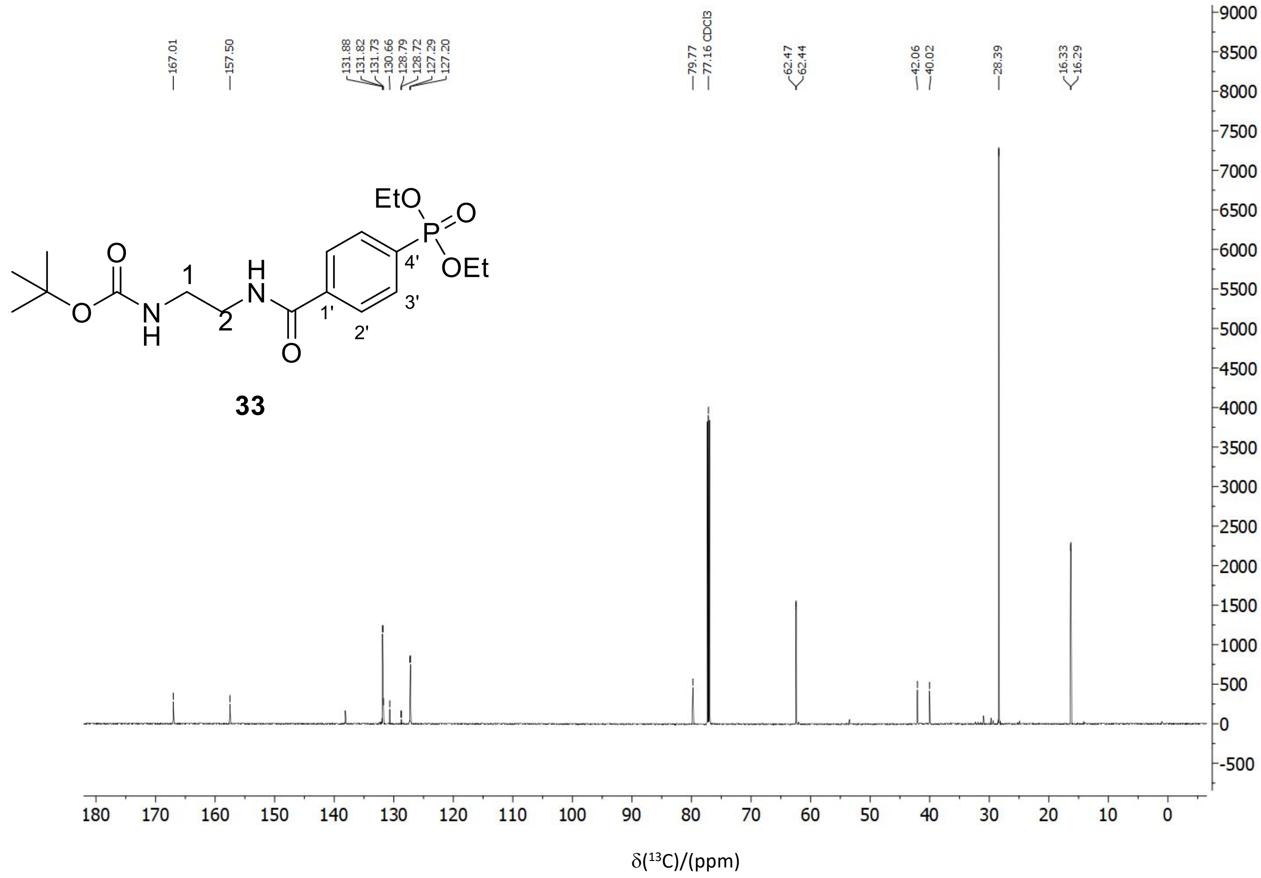

167.01
157.50
131.88
131.82
131.73
130.66
128.79
128.72
127.29
127.20
79.77
77.16 CDCl3
62.47
62.44
42.06
40.02
28.39
16.33
16.29
EtO
O
P
OEt
4'
3'
2'
1'
33
δ(13C)/(ppm)

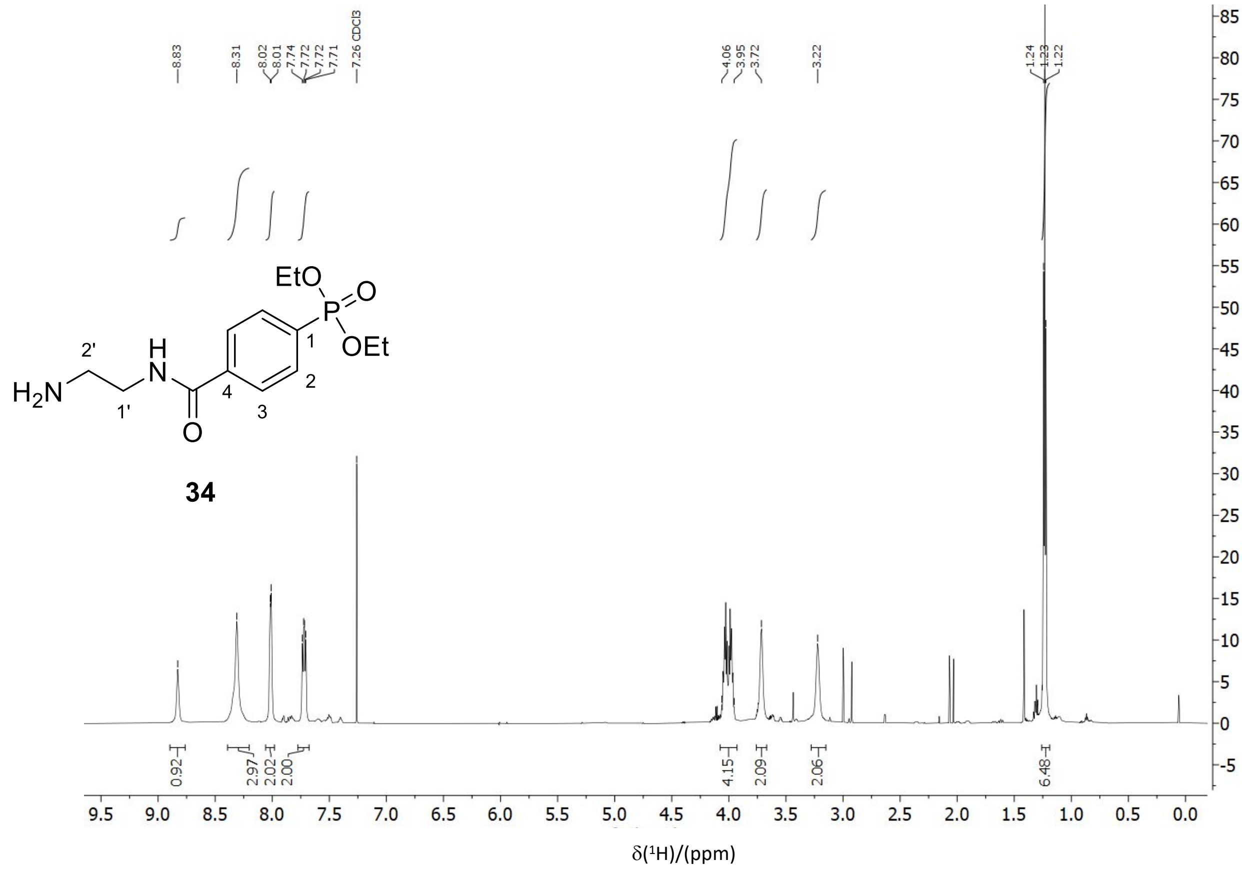
8.83
8.31
8.02
8.01
7.74
7.72
7.72
7.71
7.26 CDCl3
4.06
3.95
3.72
3.22
1.24
1.23
1.22
EtO
P
O
OEt
H
N
H_2N
2'
1'
1
2
3
4
O
34
0.92
2.97
2.02
2.00
4.15
2.09
2.06
6.48
δ(¹H)/(ppm)

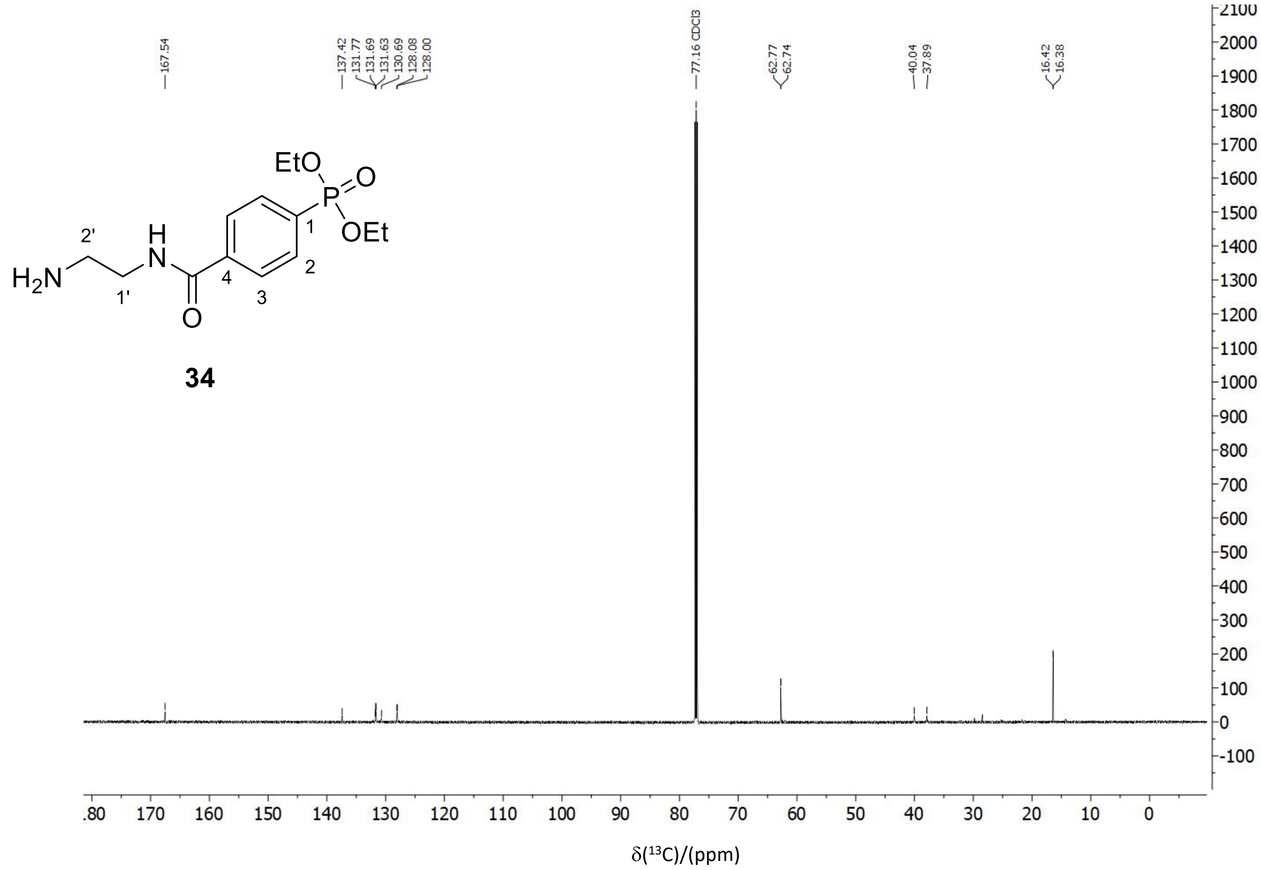
167.54
137.42
131.77
131.69
131.63
130.69
128.08
128.00
77.16 CDCl3
62.77
62.74
40.04
37.89
16.42
16.38
EtO
P
O
OEt
H
N
H_2N
2'
1'
1
2
3
4
O
34
δ(¹³C)/(ppm)

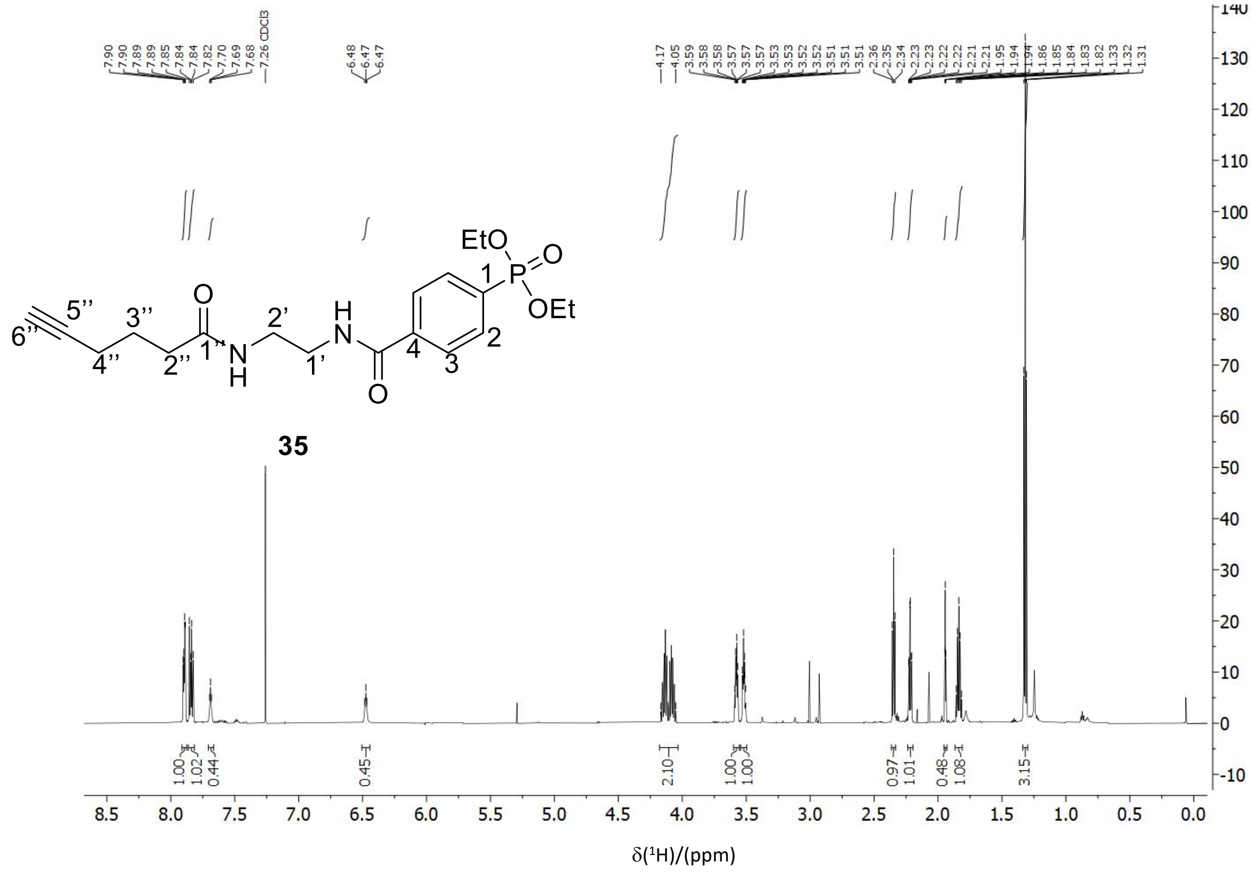
7.90 7.90 7.89 7.85 7.84 7.84 7.82 7.70 7.69 7.68 7.26 CDCl3
6.48 6.47 6.47
4.17 4.05 3.59 3.58 3.58 3.57 3.57 3.57 3.53 3.53 3.52 3.52 3.51 3.51 3.51 2.36 2.35 2.34 2.23 2.23 2.22 2.22 2.21 2.21 1.95 1.94 1.94 1.94 1.86 1.85 1.84 1.83 1.82 1.33 1.32 1.31
EtO
O
OEt
P
1
2
3
4
5''
6''
4''
3''
2''
1''
2'
1'
N
H
O
35
1.00 1.02 0.44 0.45 2.10 1.00 1.00 0.97 1.01 0.48 1.08 3.15
δ(¹H)/(ppm)

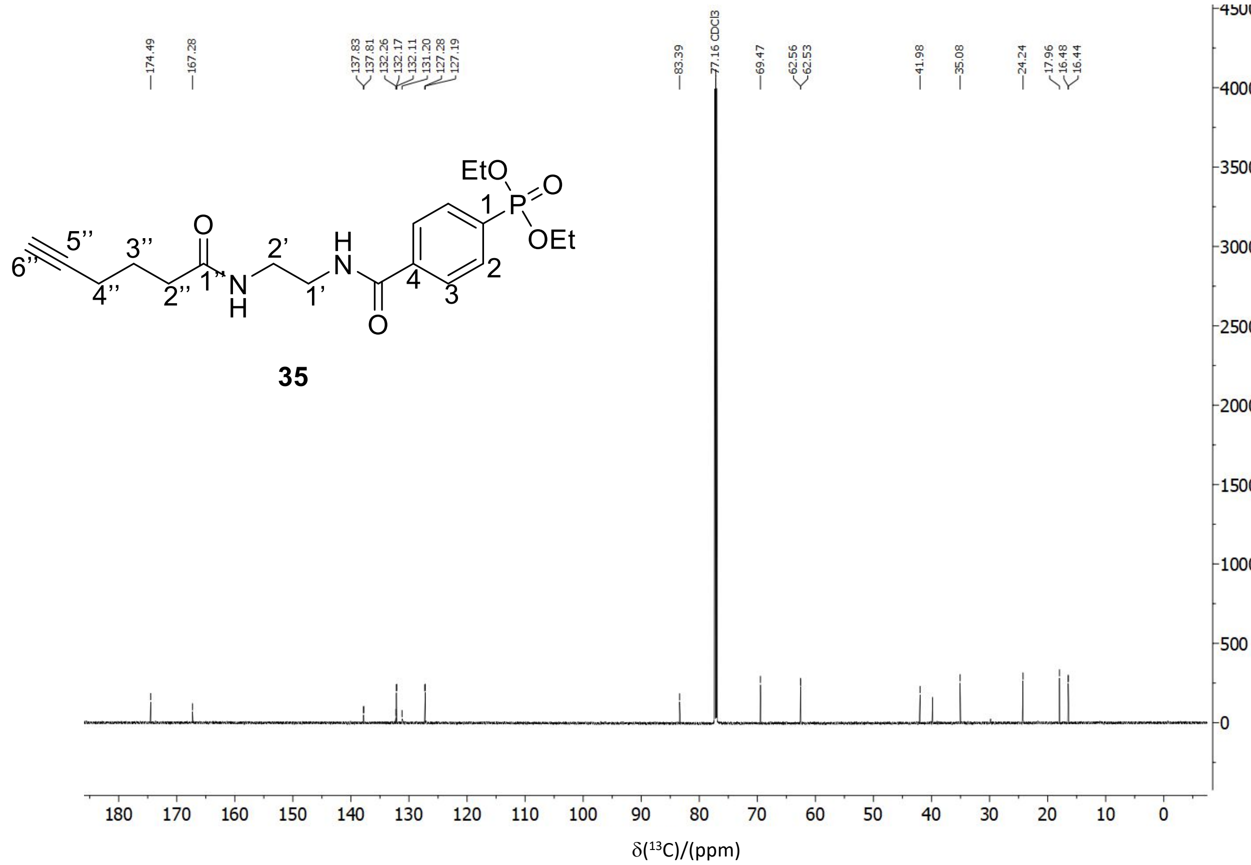
174.49
167.28
137.83 137.81 132.26 132.17 132.11 131.20 127.28 127.19
83.39
77.16 CDCl3
69.47
62.56 62.53
41.98
35.08
24.24
17.96 16.48 16.44
EtO
O
OEt
P
1
2
3
4
5''
6''
4''
3''
2''
1''
2'
1'
N
H
O
35
δ(¹³C)/(ppm)

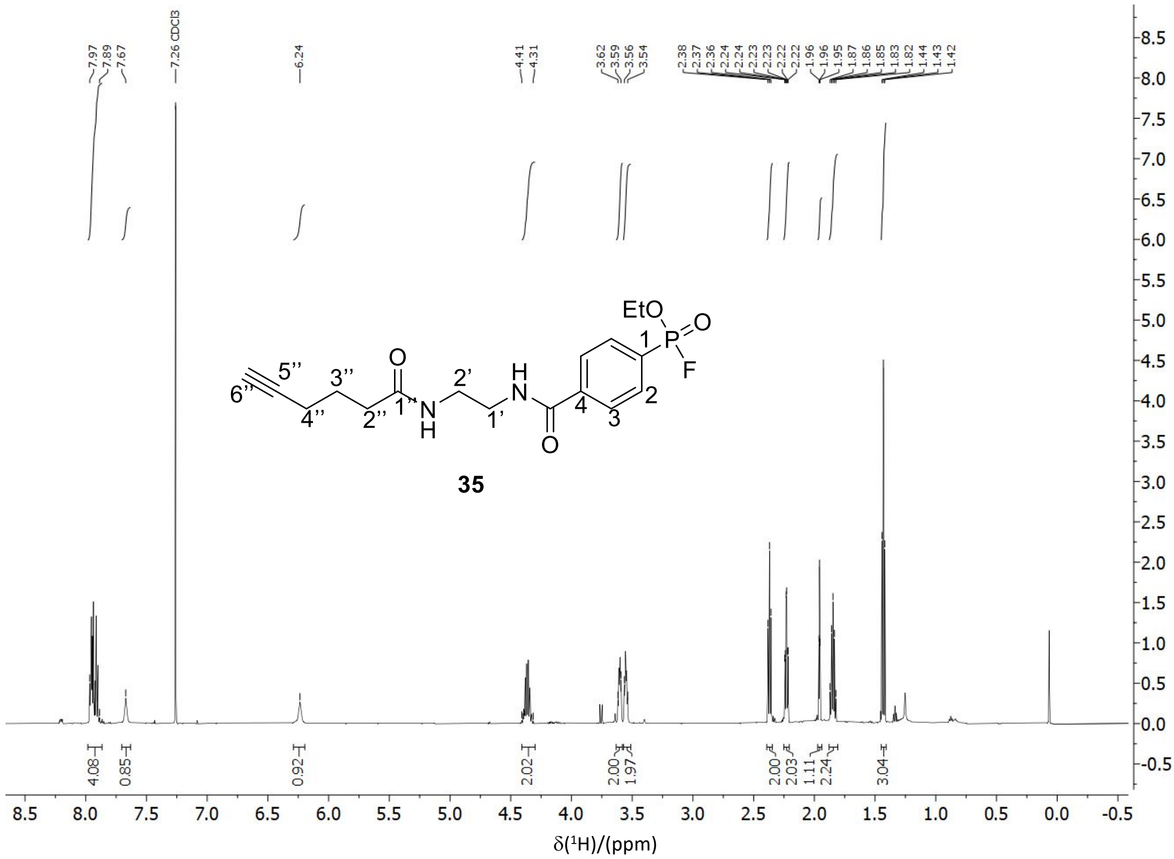
35
δ(¹H)/(ppm)

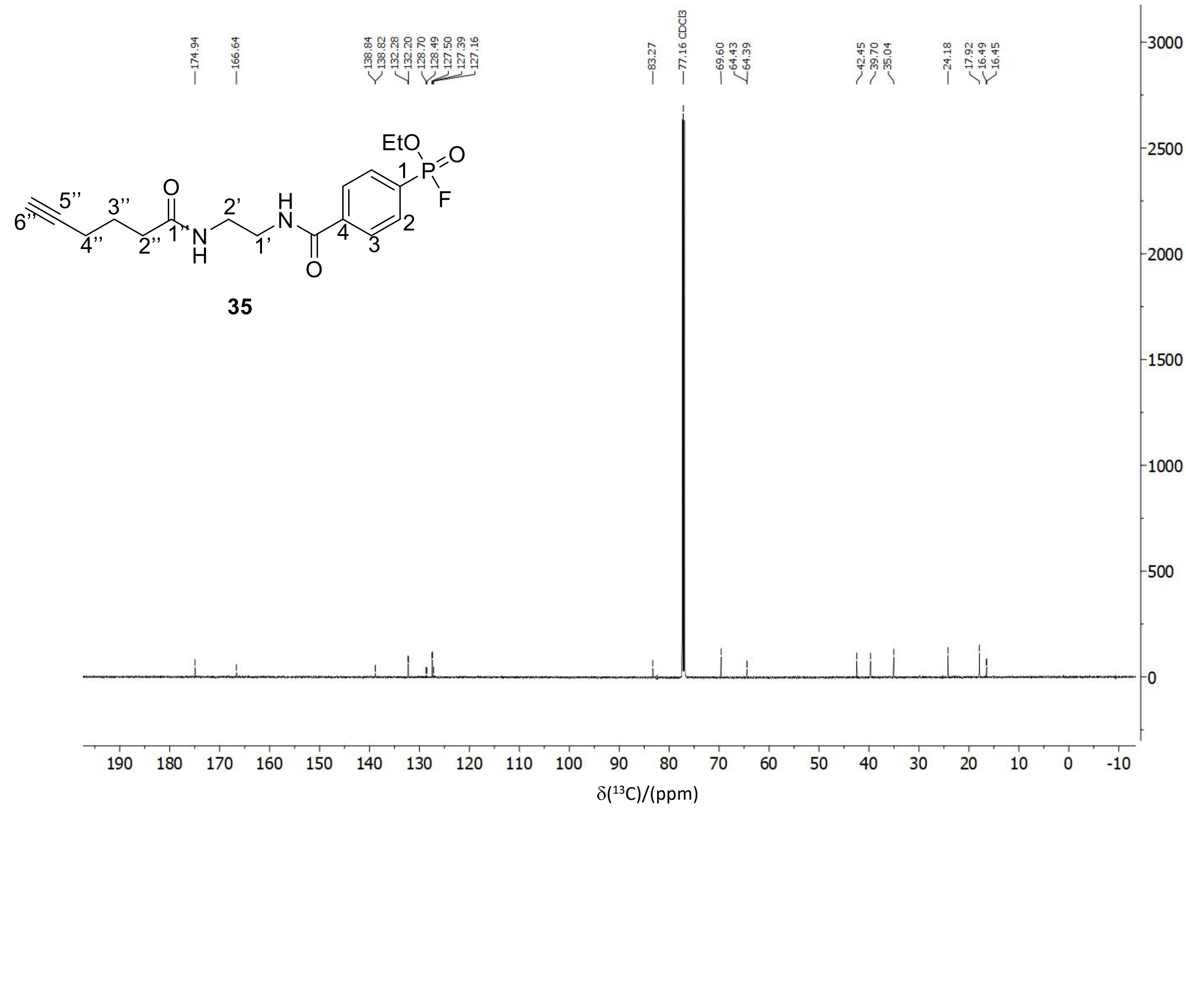
35
δ(¹³C)/(ppm)

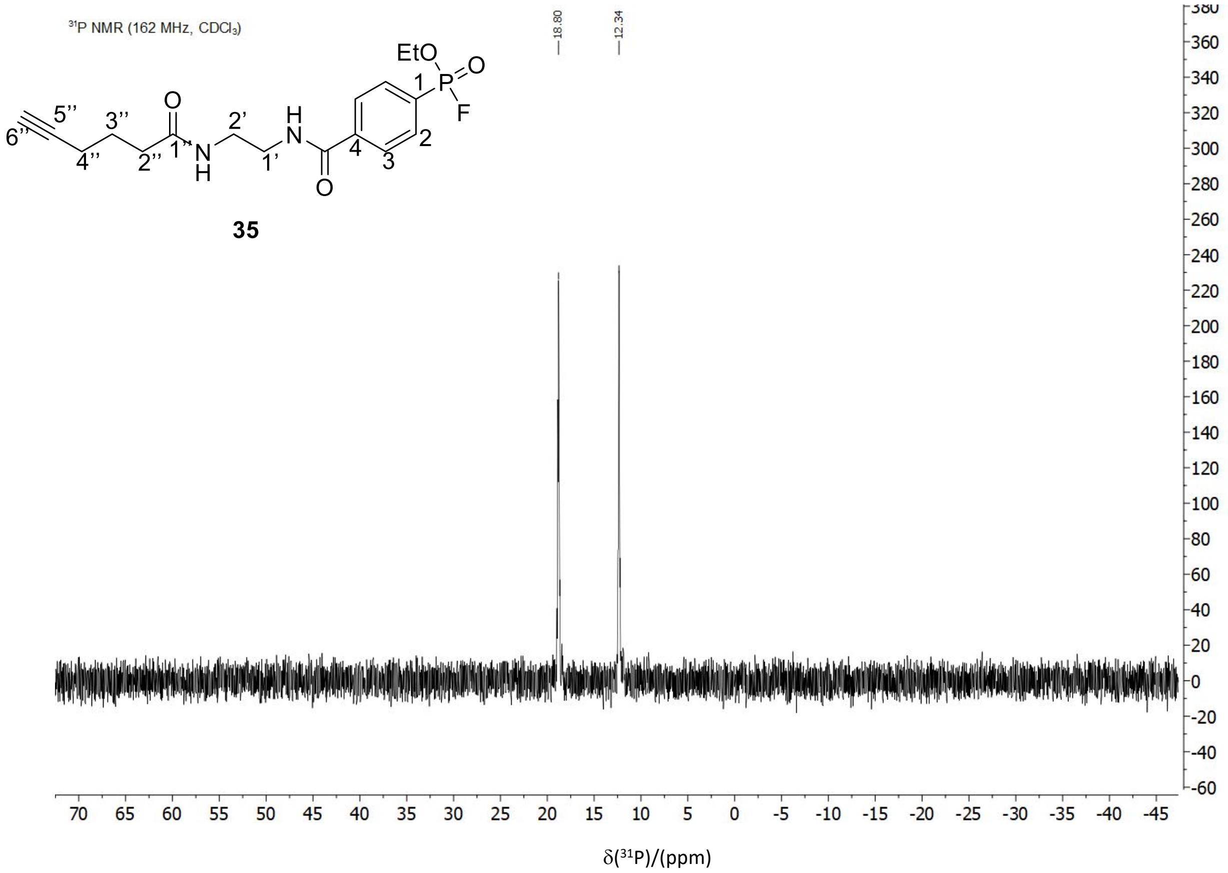
31P NMR (162 MHz, CDCl3)
18.80
12.34
EtO
F
35
δ(31P)/(ppm)

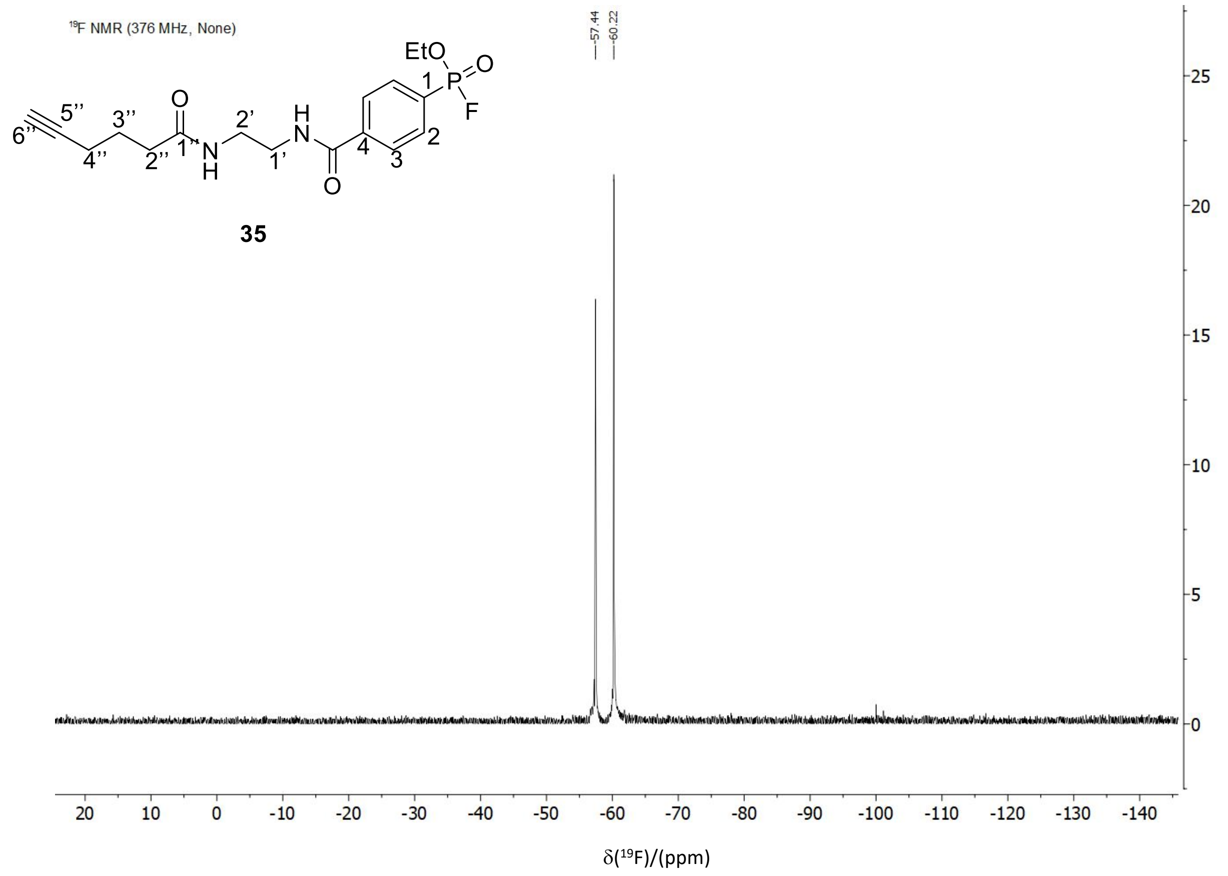
19F NMR (376 MHz, None)
-57.44
-60.22
EtO
F
35
δ(19F)/(ppm)

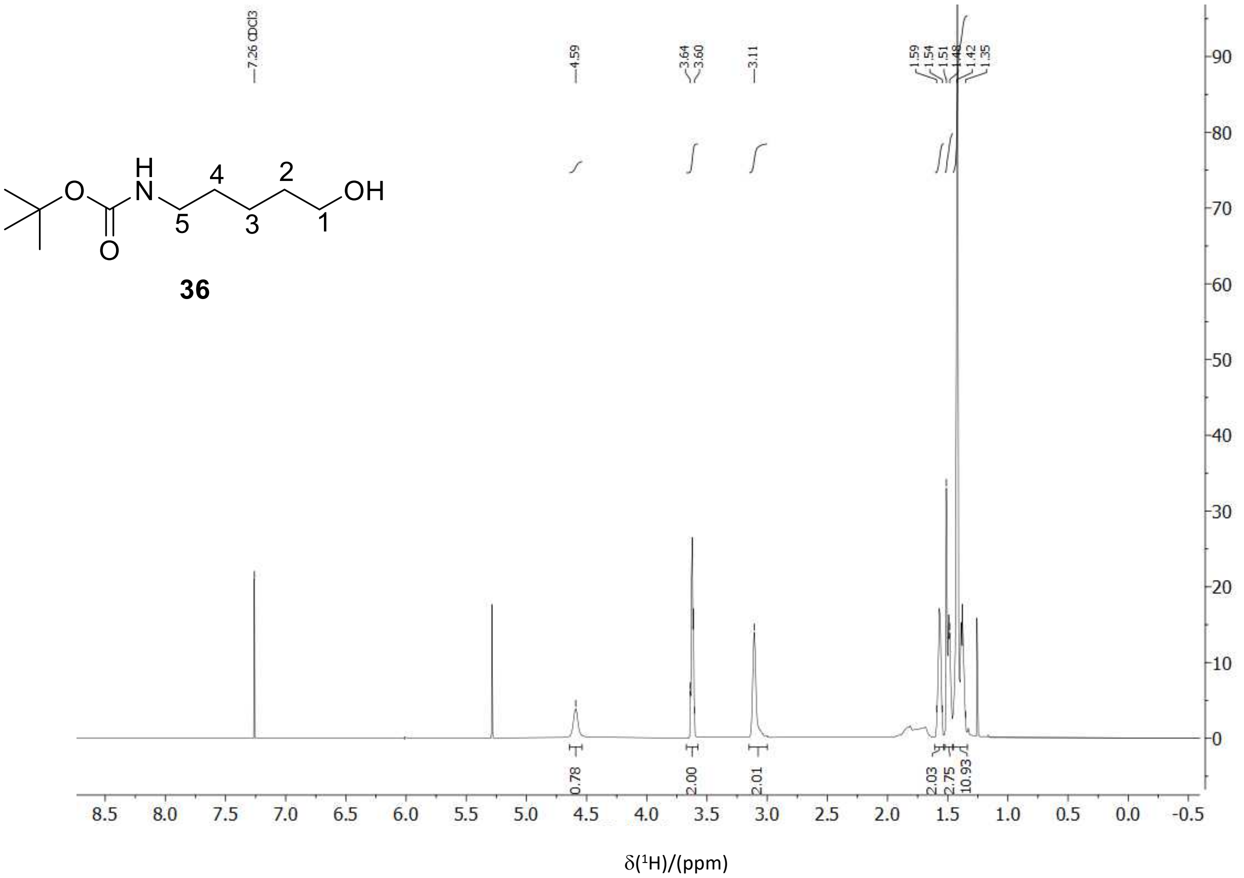
H
4
2
O
N
OH
5
3
1
O
7.26 CDCl3
4.59
3.64
3.60
3.11
1.59
1.54
1.51
1.48
1.42
1.35
0.78
2.00
2.01
2.03
2.75
10.93
δ(¹H)/(ppm)

36

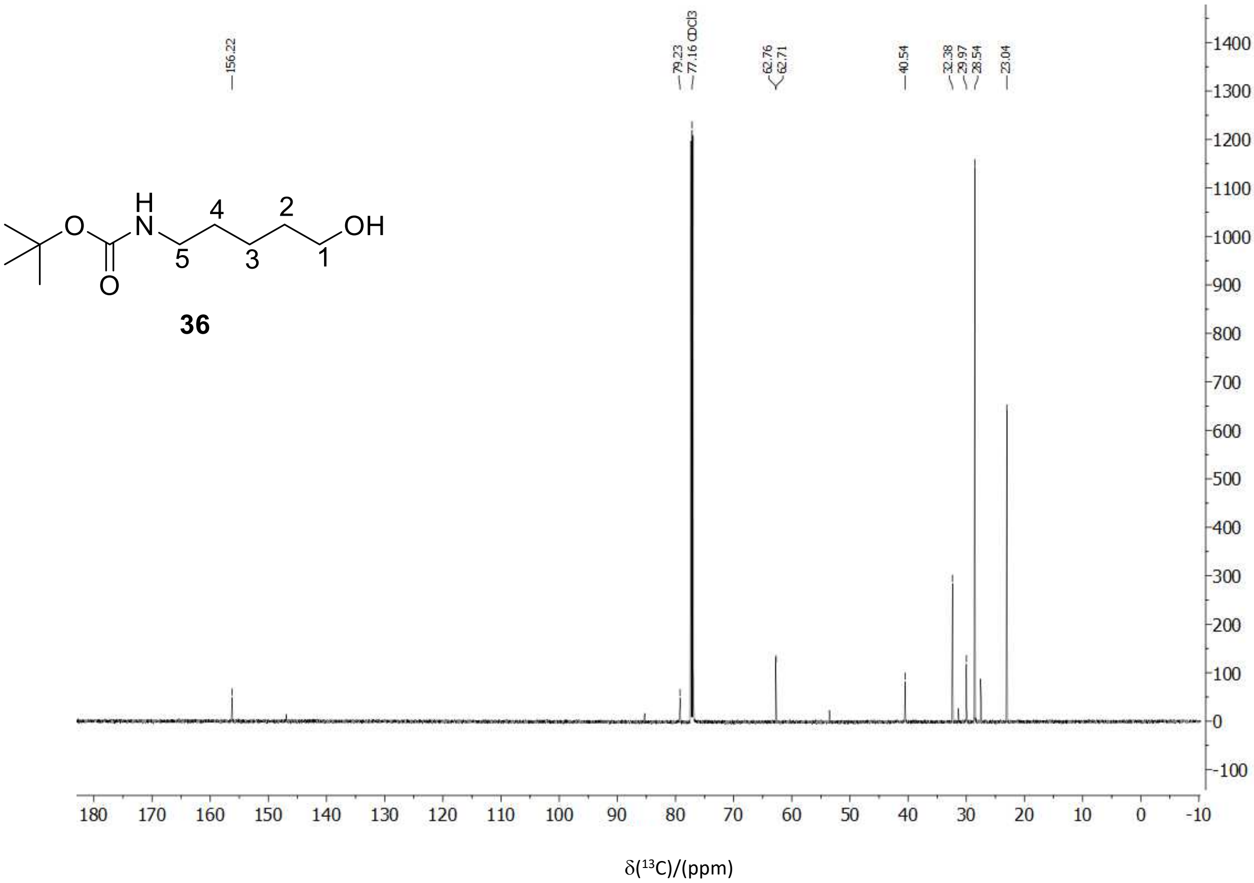
H
4
2
O
N
OH
5
3
1
O
156.22
79.23
77.16 CDCl3
62.76
62.71
40.54
32.38
29.97
28.54
23.04
δ(¹³C)/(ppm)

36

References

(1) Jones, G.; Willett, P.; Glen, R. C.; Leach, A. R.; Taylor, R. Development and Validation of a Genetic Algorithm for Flexible Docking. *J Mol Biol* **1997**, *267* (3), 727–748. https://doi.org/10.1006/jmbi.1996.0897.

(2) Verdonk, M. L.; Cole, J. C.; Hartshorn, M. J.; Murray, C. W.; Taylor, R. D. Improved Protein-Ligand Docking Using GOLD. *Proteins* **2003**, *52* (4), 609–623. https://doi.org/10.1002/prot.10465.

(3) Tatum, N. J.; Duarte, F.; Kamerlin, S. C. L.; Pohl, E. Relative Binding Energies Predict Crystallographic Binding Modes of Ethionamide Booster Lead Compounds. *J Phys Chem Lett* **2019**, *10* (9), 2244–2249. https://doi.org/10.1021/acs.jpclett.9b00741.

(4) Wan, S.; Bhati, A. P.; Zasada, S. J.; Coveney, P. V. Rapid, Accurate, Precise and Reproducible Ligand-Protein Binding Free Energy Prediction. *Interface Focus* **2020**, *10* (6), 20200007. https://doi.org/10.1098/rsfs.2020.0007.

(5) Suzuki, I.; Kiyokawa, K.; Yasuda, M.; Baba, A. Indium(III) Halide-Catalyzed UV-Irradiated Radical Coupling of Iodomethylphosphorus Compounds with Various Organostannanes. *Org Lett* **2013**, *15* (7), 1728–1731. https://doi.org/10.1021/ol4005257.

(6) Sudhakar, G.; Reddy, K. J.; Nanubolu, J. B. Total Synthesis of Palmyrolide A and Its 5,7-*Epi* Isomers. *Tetrahedron* **2013**, *69* (11), 2419–2429. https://doi.org/10.1016/j.tet.2013.01.048.

(7) Thompson, A. M.; Delaney, A. M.; Hamby, J. M.; Schroeder, M. C.; Spoon, T. A.; Crean, S. M.; Showalter, H. D. H.; Denny, W. A. Synthesis and Structure-Activity Relationships of Soluble 7-Substituted 3-(3,5-Dimethoxyphenyl)-1,6-Naphthyridin-2-Amines and Related Ureas as Dual Inhibitors of the Fibroblast Growth Factor Receptor-1 and Vascular Endothelial Growth Factor Receptor-2 Tyrosine Kinases. *J Med Chem* **2005**, *48* (14), 4628–4653. https://doi.org/10.1021/jm0500931.

(8) Anthony, N. G.; Breen, D.; Clarke, J.; Donoghue, G.; Drummond, A. J.; Ellis, E. M.; Gemmell, C. G.; Helesbeux, J.-J.; Hunter, I. S.; Khalaf, A. I.; Mackay, S. P.; Parkinson, J. A.; Suckling, C. J.; Waigh, R. D. Antimicrobial Lexitropsins Containing Amide, Amidine, and Alkene Linking Groups. *J Med Chem* **2007**, *50* (24), 6116–6125. https://doi.org/10.1021/jm070831g.

(9) Eirich, J.; Orth, R.; Sieber, S. A. Unraveling the Protein Targets of Vancomycin in Living S. Aureus and E. Faecalis Cells. *J Am Chem Soc* **2011**, *133* (31), 12144–12153. https://doi.org/10.1021/ja2039979.

(10) Kohler, M. C.; Sokol, J. G.; Stockland, R. A. Development of a Room Temperature Hirao Reaction. *Tetrahedron Letters* **2009**, *50* (4), 457–459. https://doi.org/10.1016/j.tetlet.2008.11.040.

(11) Sharma, S.; Oehlschlager, A. C. Scope and Mechanism of Stannylalumination of 1-Alkynes. *J. Org. Chem.* **1989**, *54* (21), 5064–5073. https://doi.org/10.1021/jo00282a021.

(12) Bergkamp, J. J.; Sherman, B. D.; Mariño-Ochoa, E.; Palacios, R. E.; Cosa, G.; Moore, T.; Gust, D.; Moore, A. Synthesis and Characterization of Silicon Phthalocyanines Bearing Axial Phenoxyl Groups for Attachment to Semiconducting Metal Oxides. *Journal of Porphyrins and Phthalocyanines* **2011**, *15* (9–10), 943–950. https://doi.org/10.1142/S1088424611003847.

(13) Fisher, B. F.; Gellman, S. H. Impact of γ-Amino Acid Residue Preorganization on α/γ-Peptide Foldamer Helicity in Aqueous Solution. *J Am Chem Soc* **2016**, *138* (34), 10766–10769. https://doi.org/10.1021/jacs.6b06177.

(14) Lasse, C.; Azevedo, C. S.; de Araújo, C. N.; Motta, F. N.; Andrade, M. A.; Rocha, A. P.; Sampaio, I.; Charneau, S.; Gèze, M.; Grellier, P.; Santana, J. M.; Bastos, I. M. D. Prolyl Oligopeptidase From Leishmania Infantum: Biochemical Characterization and Involvement in Macrophage Infection. *Front Microbiol* **2020**, *11*, 1060. https://doi.org/10.3389/fmicb.2020.01060.

(15) Cox, J.; Mann, M. MaxQuant Enables High Peptide Identification Rates, Individualized p.p.b.-Range Mass Accuracies and Proteome-Wide Protein Quantification. *Nat Biotechnol* **2008**, *26* (12), 1367–1372. https://doi.org/10.1038/nbt.1511.

(16) Cox, J.; Neuhauser, N.; Michalski, A.; Scheltema, R. A.; Olsen, J. V.; Mann, M. Andromeda: A Peptide Search Engine Integrated into the MaxQuant Environment. *J Proteome Res* **2011**, *10* (4), 1794–1805. https://doi.org/10.1021/pr101065j.

(17) Tyanova, S.; Temu, T.; Sinitcyn, P.; Carlson, A.; Hein, M. Y.; Geiger, T.; Mann, M.; Cox, J. The Perseus Computational Platform for Comprehensive Analysis of (Prote)Omics Data. *Nat Methods* **2016**, *13* (9), 731–740. https://doi.org/10.1038/nmeth.3901.

(18) Jumper, J.; Evans, R.; Pritzel, A.; Green, T.; Figurnov, M.; Ronneberger, O.; Tunyasuvunakool, K.; Bates, R.; Žídek, A.; Potapenko, A.; Bridgland, A.; Meyer, C.; Kohl, S. A. A.; Ballard, A. J.; Cowie, A.; Romera-Paredes, B.; Nikolov, S.; Jain, R.; Adler, J.; Back, T.; Petersen, S.; Reiman, D.; Clancy, E.; Zielinski, M.; Steinegger, M.; Pacholska, M.; Berghammer, T.; Bodenstein, S.; Silver, D.; Vinyals, O.; Senior, A. W.; Kavukcuoglu, K.; Kohli, P.; Hassabis, D. Highly Accurate Protein Structure Prediction with AlphaFold. *Nature* **2021**, *596* (7873), 583–589. https://doi.org/10.1038/s41586-021-03819-2.

(19) The PyMOL Molecular Graphics System, Version 3.0 Schrödinger, LLC.
